# MnO_2_-Mediated Oxidative Cyclization of “Formal” Schiff’s Bases: Easy Access to Diverse Naphthofuro-Annulated Triazines

**DOI:** 10.3390/molecules27207105

**Published:** 2022-10-21

**Authors:** Ramil F. Fatykhov, Igor A. Khalymbadzha, Ainur D. Sharapov, Anastasia P. Potapova, Nataliya N. Mochulskaya, Anton N. Tsmokalyuk, Alexandra V. Ivoilova, Polina N. Mozharovskaia, Sougata Santra, Oleg N. Chupakhin

**Affiliations:** 1 Department of Organic and Biomolecular Chemistry, Chemical Engineering Institute, Ural Federal University, 19 Mira Str., 620002 Ekaterinburg, Russia; 2 Postovsky Institute of Organic Synthesis, Ural Branch of the Russian Academy of Sciences, 22 S. Kovalevskaya Str., 620990 Ekaterinburg, Russia

**Keywords:** oxidative cyclization, manganese(IV) oxide, 1,2,4-triazine, phenols, cross-coupling

## Abstract

A different type of MnO_2_-induced oxidative cyclization of dihydrotriazines has been developed. These dihydrotriazines are considered as a “formal” Schiff’s base. This method provided easy access to naphthofuro-fused triazine via the C-C/C-O oxidative coupling reaction. The reaction sequence comprised the nucleophilic addition of 2-naphthol or phenol to 1,2,4-triazine, followed by oxidative cyclization. The scope and limitations of this novel coupling reaction have been investigated. Further application of the synthesized compound has been demonstrated by synthesizing carbazole-substituted benzofuro-fused triazines. The scalability of the reaction was demonstrated at a 40 mmol load. The mechanistic study strongly suggests that this reaction proceeds through the formation of an O-coordinated manganese complex.

## 1. Introduction

In organic synthesis, C−H functionalization in the presence of transition metal catalysts has become one of the fundamental methods, and has had a massive impact on synthetic organic chemistry, medicinal chemistry, and material science [1,2,3,4,5,6,7,8]. In this context, cross dehydrogenative coupling (CDC) reactions have gained much interest in the last decade [9,10,11,12,13,14,15] among all types of C-H functionalization/activation reactions. This type of coupling reaction allows the construction of a C-C bond or C-X bond directly from C-H-containing substrates in the presence of an oxidant via the formal removal of a H_2_ molecule. In addition, these methods avoid the prefunctionalization of starting materials, which makes the synthetic routes straightforward and more efficient. For CDC reactions, various transition metals such as Pd, Cu, Ag, Rh, and Ru have been extensively studied due to their high efficiency. However, the exploration of manganese catalysis in CDC reactions is in high demand due to its low price, ready availability, sustainability, nontoxicity, and environmentally friendly properties [16]. Simple manganese salts were sensibly employed in the CDC reaction due to their ability to undergo the reaction in a radical way.

**Figure 1:**
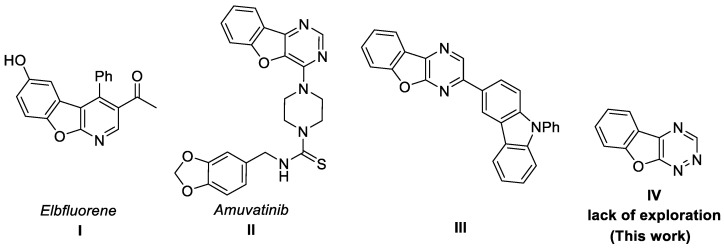
Representative benzofuro-fused N-heterocycles.

Benzofuro-fused N-heterocycles are considered as common structural motifs in biologically active compounds, drug candidates and fluorescence materials (Figure 1). For example, benzofuro [2,3-*b*]pyridine, in particular Elbfluorene **I**, and its derivatives are important cyclin-dependent kinase inhibitors [17,18,19,20], the benzofuro [3,2-*d*]pyrimidine derivative Amuvatinib **II** is a multitarget tyrosine kinase inhibitor [21,22,23,24], and benzofuro[2,3-*b*]pyrazine **III** was designed as a deep-blue fluorescent emitter [25].

**Scheme 1:**
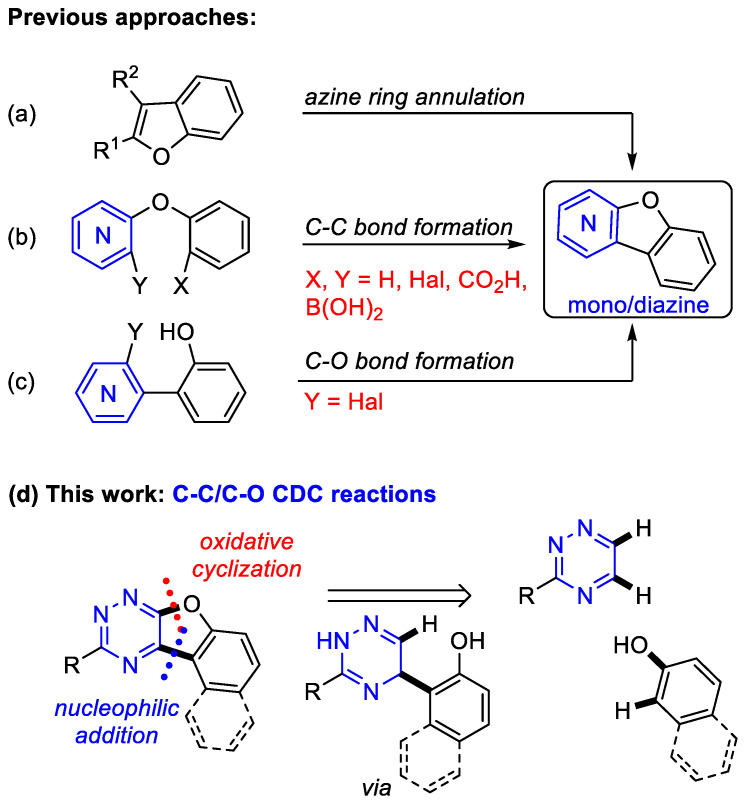
Approaches to Benzofuro-fused N-heterocycles. (**a**–**c**): Known approaches; (**d**) Approach of this work.

Considering the importance of this moiety, several methods have been developed for developing benzofuro-fused N-heterocycles such as pyridine, pyrimidine and pyrazine derivatives. The first synthetic approach comprises the annulation of the heterocyclic ring to a benzofuran core (Scheme 1a) [26,27,28,29,30,31,32,33,34]. Alternatively, other approaches include the intramolecular cyclization (C-C bond formation) of arylhetaryl ethers [35,36,37,38,39,40] (Scheme 1b) or the intramolecular cyclization (C-O bond formation) of 2-hetaryl-substituted phenol derivatives [41,42,43,44,45,46,47] (Scheme 1c), as well as the intermolecular tandem C-C/C-O cross-coupling reaction of prefunctionalized substrate [48,49,50] to form a furan ring fused between the benzene and mono/diazine ring. However, these approaches usually require multistep synthesis, harsh reaction conditions, and the use of transition metal catalysts or special reagents and conditions.

On the other hand, it is worth mentioning that information about the synthetic and applied data of benzofuro-fused triazines is lacking in the literature. To date, only a few studies have investigated the synthesis of benzofurotriazine derivatives. In 1988, Eid et al. reported the synthesis of naphthofuro[2,3-*e*][1,2,4]triazine in a 33% overall yield via the annulation of the triazine core to naphthofuran-1,2-dione [51]. The reaction was carried out in four steps. Later, Seitz and Richter reported that the intramolecular [4+2]-cycloaddition of 2-(tetrazinyloxy)benzonitrile led to the formation of benzofuro[3,2-*e*][1,2,4]triazine derivatives [52]. Neunhoeffer et al. synthesized benzofuro[2,3-e][1,2,4]triazine at 26% yield using the tandem S_N_^H^-S_N_^ipso^ reaction of resorcinol and 1,2,4-triazine with a good leaving group [53]. Unfortunately, these methods represent the only examples of benzofurotriazine derivatives, and provide poor yields of the desired products. At the same time, 1,2,4-triazines represent readily accessible and cheap building blocks for the construction of pyridine [54,55,56,57,58,59,60,61,62], pyrimidine [63,64] or pyrazine [59,65,66] cores via the sequence of Diels-Alder/*retro*-Diels-Alder reactions.

In a continuation of our research on CDC reactions in triazines [54,67] and diazines [68], herein, we are pleased to report an unusual synthesis of benzofuro-fused 1,2,4-triazines via the sequence of C-C/C-O CDC reactions of 1,2,4-triazines with 2-naphthols or phenols (Scheme 1d). The reaction proceeded through the formation of 1,4-dihydrotriazine, followed by oxidative cyclization.

## 2. Results and Discussion

**Scheme 2:**
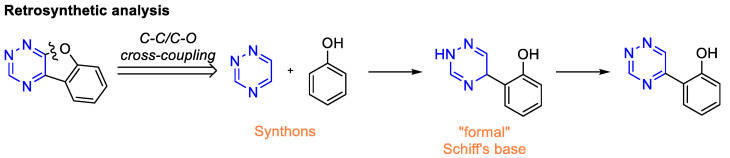
Retrosynthetic analyses of benzofurotriazine scaffold.

Based on retrosynthetic analysis of benzofurotriazine (Scheme 1d), we assume that 5,6-unsubstituted triazine and α-unsubstituted phenol are the best building blocks for the construction of the desired molecule through a sequence of C-C/C-O cross-coupling reactions. Earlier, our [67,69,70] and other [70,71] research groups demonstrated that 5,6-unsubstituted 1,2,4-triazines may be used in a two-step CDC reaction with various aromatic C-nucleophiles via the formation of 1,4-dihydrotriazine derivatives as intermediates, followed by aromatization to bi(het)aryl products. As mentioned above, the prefunctionalized azine is required for C-O cross-coupling reaction [53]. On the other hand, it is well known that the phenolic Schiff’s bases readily undergoes intramolecular oxidative cyclization in the presence of various oxidizing agents, in particular, hypervalent iodine compounds [72,73,74,75], lead(IV) acetate [76,77,78], or manganese salts such as Mn(OAc)_3_^.^2H_2_O [78,79] and MnO_2_ [80]. We hypothesized that 1,4-dihydrotriazines containing 2-hydroxyaryl moiety can be considered as a “formal” phenolic Schiff’s base (Scheme 2).

**Scheme 3:**
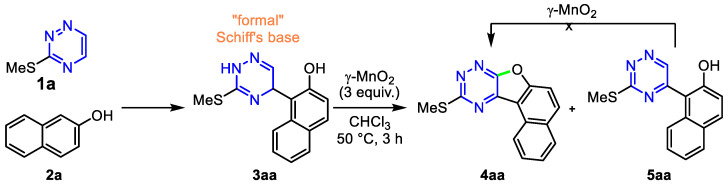
Synthesis and oxidation of dihydrotriazine **3aa**.

Based on this hypothesis, we have focused our attention on the oxidative cyclization of dihydrotriazines easily obtainable from triazine and naphthol. For example, the reaction of readily available 3-methylthio-1,2,4-triazine **1a** and naphthol **2a** yields dihydrotriazine **3aa** (Scheme 3), which was used for the initial screening of the optimal conditions. Using standard oxidizing agent, such as phenyliodonine(III) diacetate, phenyliodonine(III) bis(trifluoroacetate) or Pb(OAc)_4_, for the oxidative cyclization of the phenolic Schiff’s base, only a complex mixture was isolated from the reaction. Surprisingly, when using MnO_2_ for the oxidation of the “formal” Schiff’s base **3aa,** the desired oxidative coupling product naphthofurotriazine **4aa** was formed in one step. At the same time, the side product **5aa** was also observed in the reaction (Scheme 3). After comprehensive screening (Please see Supporting Information for details, Section S6), we found that the vigorous stirring (1500 rpm) of **3aa** in CHCl_3_ at 50 °C in the presence of 3 equiv. of γ-MnO_2_ [81] provided the naphthofurotriazine **4aa** in an almost quantitative yield after 3 h (Table 1, entry 1). Besides γ-MnO_2_, other manganese salts such as Mn(OAc)_3_^.^2H_2_O, Mn(OAc)_2_.4H_2_O and MnCl_2_, Mn(acac)_2_ were not so effective for this reaction, or provided **4aa** in very poor yields (Table 1, entries 3-6), except MnO_2_ impregnated with nitric acid [82], which afforded **4aa** in a good yield (Table 1, entry 2). One may assume that Mn(OAc)_3_ has low oxidative potential in organic media [83] compared to MnO_2_. Other alternative oxidants such as Ag_2_O, DTBP and DDQ led to low yields (Table 1, entries 7-9), and *p*-chloranil exclusively provided compound **5aa** in a high yield (Table 1, entry 10). The use of other solvents such as 1,1,1,3,3,3-hexafluoro-2-propanol (HFIP), EtOH, DCE or benzene clearly gave worse results (Table 1, entries 11-14). By increasing the temperature, the yield of **4aa** was decreased (Table 1, entry 15). In contrast, lower conversion was observed at room temperature (Table 1, entry 16). Carrying out the reaction in the presence of a decreased amount of MnO_2_ (Table 1, entry 17) had negative effects on the efficiency of the reaction. In contrast, using 5 equiv. of MnO_2_ increased the yield of the side product **5aa** (Table 1, entry 18). At the same time, all attempts were unsuccessful to cyclize **5aa** to **4aa**.

**Scheme 4:**
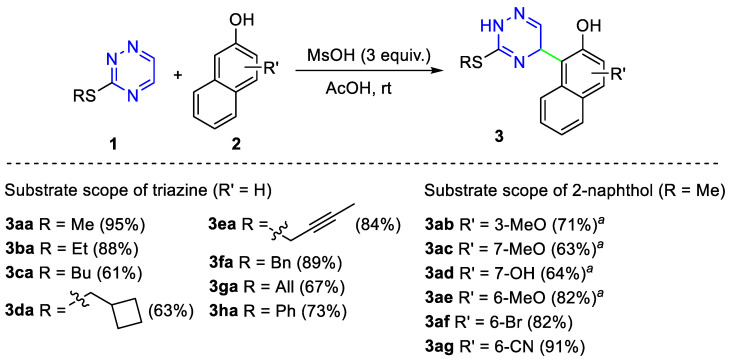
Substrate scope for the synthesis of compounds **3**. Reaction conditions*:* **1** (1 mmol), **2** (1 mmol), MsOH (3 mmol) in AcOH (4 mL). *^a^* **1** (1 mmol), **2** (1 mmol), BF_3_^.^OEt_2_ (8 mmol) in methanol 4 mL under reflux.

In order to study the applicability of the proposed oxidative coupling reaction, we synthesized a series of starting dihydrotriazines **3**. It was observed that our earlier proposed method [54] of the nucleophilic addition of 5,7-dimethoxycoumarins to 1,2,4-triazines with some modifications allowed us to prepare a series of compounds **3** using a variety of 3-S-substituted 1,2,4-triazines **1** and 2-naphthols **2** (Scheme 4). In all cases, the reaction proceeded with high regioselectivity to give compounds **3** in good to high yields. When methoxy- or hydroxy-substituted 2-naphthols **2b-e** were involved in the reaction with 1,2,4-triazine, the best yields were achieved in the presence of BF_3_^.^OEt_2_ under refluxed conditions in methanol.

**Scheme 5:**
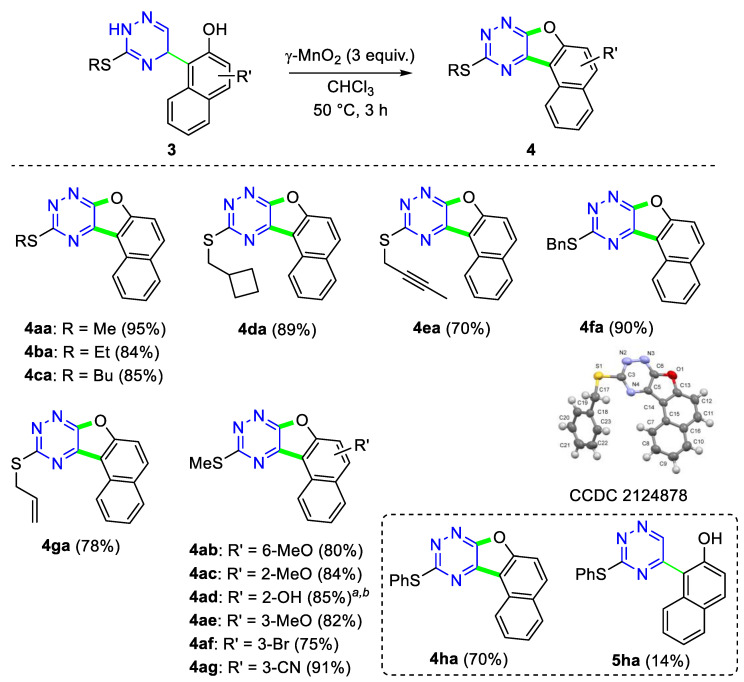
Substrate scope for the synthesis of benzofuro-fused 1,2,4-triazines 4. Reaction conditions: **3** (0.2 mmol), γ-MnO_2_ (0.6 mmol) in CHCl_3_ (3 mL), 50 °C, 3 h. *^a^ *Two-step yield; *^b^ *CHCl_3_:EtOH (*v*/*v* = 4:1, 3 mL).

With the optimized reaction conditions and a set of dihydrotriazines **3** in hand, we then examined the applicability and scope of this MnO_2_-induced oxidative cyclization reaction of dihydrotriazines **3**. At first, the scope of the reaction was studied with respect to different S-substituents in the dihydrotriazine core, and the results are summarized in Scheme 5. The naphthofuro-fused triazine **4aa** was isolated in a 95% yield under optimal reaction conditions after recrystallization from MeCN. Other 3-alkylthio-substituted triazine derivatives **3ba-3da** and **3fa** also underwent oxidative cyclization, producing only the desired cyclic product **4** in good to high yields. Moreover, 3-(but-2-yn-1-yl)- and 3-allylthio derivatives **3ea** and **3ga** smoothly transformed to **4ea** and **4ga** in 70% and 78% yields, respectively. However, in the case of phenylthio-substituted derivative **3ha,** a 5:1 mixture of **4ha** and **5ha** was isolated. Next, an investigation of this coupling reaction on 3-methylthiotriazine adducts **3ab**-**3ag** showed that the naphthyl ring substituted with various functional groups at different positions afforded the corresponding products with good to excellent yields. For example, bromo-, hydroxy-, methoxy- and cyano-substituted adducts **3ab**-**3ag** underwent oxidative cyclization with high regioselectivity to give only naphthofuro[3,2-*e*]triazine derivatives **4** in up to 91% yields (Scheme 5).

**Scheme 6:**
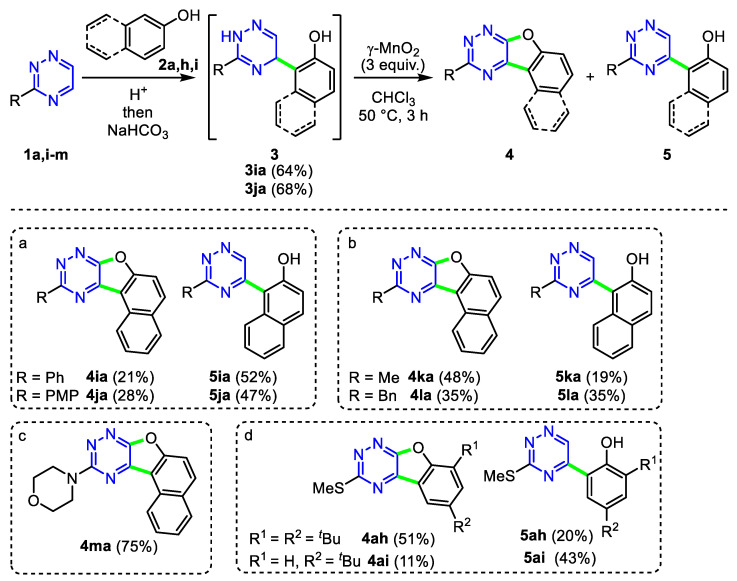
Addition/oxidative cyclization sequence of triazine with naphthol and phenol. Reaction conditions: **1** (1 mmol), **2** (1 mmol), (**a**) MsOH (3 mmol), AcOH (5 mL), rt; (**b**) AcOH (5 mL), rt; (**c**) BF_3_^.^OEt_2_ (3 mmol), MeOH (5 mL), reflux; (**d**) TFA (4 mL), rt.

Encouraged by these results, we then investigated the oxidative cyclization reaction of triazine not bearing S-substituents (Scheme 6). In particular, 3-phenyl and 3-(4-methoxyphenyl) (PMP) derivatives **3ia** and **3ja** prepared under standard conditions (MsOH, AcOH) underwent MnO_2_-induced oxidative cyclization to afford cyclic products **4ia** and **4ja,** respectively, as minor products with up to 28% yield. In contrast, 3-methyltriazine **1k** smoothly reacted with 2-naphthol **2a** in AcOH without the addition of MsOH, leading to the corresponding adduct **3la**, which was oxidized in the presence of MnO_2_ to generate the desired **4la** as a major product in a 48% overall yield. Similar to triazine **1k**, 3-benzyltriazine **1l** was also involved in the same cascade reaction to give the mixture of **4ka** and **5ka** in a ratio of 1:1. In addition, we were pleased to find that the oxidative cyclization of **3ma** bearing the *N*-morpholinyl group in a triazine core produced the respective oxidative product **4ma** in a 75% yield. Actually, the adduct **3ma** was synthesized in situ by the interaction between triazine **1m** and 2-naphthol **2a** in the presence of BF_3_^.^OEt_2_ under reflux in methanol.

Further, we explored the reactivity of *p*-substituted phenols in these sequence reactions. Unfortunately, all attempts to prepare the starting materials (**3**) under standard conditions (MsOH, AcOH, rt) failed, and only starting materials **1** and **2** were isolated from the reactions. However, we found that the use of trifluoroacetic acid (TFA) as the activator and medium at room temperature could allow the formation of unstable compounds **3ah** and **3ai** by the nucleophilic addition of phenol to the triazine core. These two compounds (**3ah** and **3ai**) underwent the oxidative cyclization reaction, giving benzofuro[3,2-*e*]triazine **4** in lower to moderate yields. At the same time, biaryl by-products **5ah** and **5ai** were also isolated.

For further assessing the synthetic utility of the method, we performed the addition and coupling reaction sequence again at the gram scale. Thus, under slightly optimized conditions, we synthesized compound **4ad** from triazine **1a** and naphthol **2d** at 40 mmol loading in an 85% yield via two steps (Scheme 4).

**Scheme 7:**
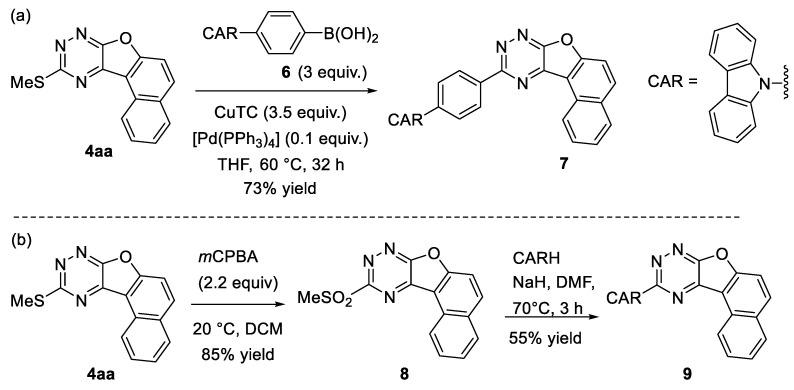
Further application of the synthesized compound. (**a**) Suzuki coupling; (**b**) Replacement of good leaving group.

The thiomethyl group is a versatile moiety for coupling reactions. In triazines, the thiomethyl group may be easily substituted with aryl boronic acids [84,85] or trialkyl(aryl)stannanes [86] using Liebeskind–Srogl coupling [87]. To demonstrate the synthetic potential of benzofuro-annulated triazines, we performed the substitution of the thiomethyl group with an aryl substituent. The reaction of triazine **4aa** with 4-carbazolyl-phenylboronic acid **6** provided the corresponding coupling product **7** in a 73% yield (Scheme 7a).

Furthermore, a thiomethyl group can easily be oxidized with *m*CPBA to afford the methylsulfonyl group, which can be substituted with various nucleophiles [88,89,90]. Treatment of the synthesized compound **4aa** with *m*CPBA gave the corresponding sulfonyl derivative **8** at an 85% yield. After that, we successfully synthesized carbazole-substituted naphthofuro-fused 1,2,4-triazine **9** via the subsequent replacement of the sulfonyl group in **8** with carbazole in the presence of sodium hydride (Scheme 7b). It is worth mentioning that these types of carbazole-substituted triazine derivatives have potential uses in biological fields [91,92] and OLED applications [93].

**Scheme 8:**
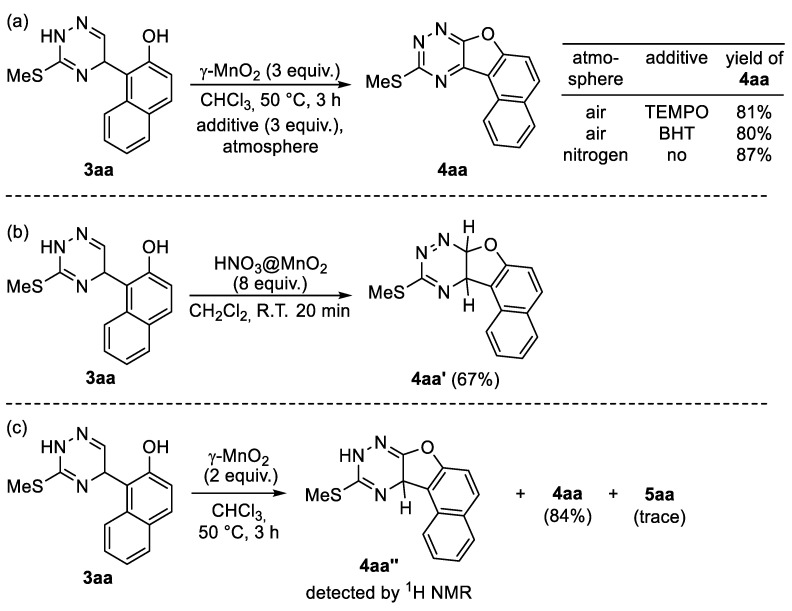
Control experiments for mechanistic investigation. (**a**) Oxidation in an inert and oxygen atmosphere; (**b**) Using MnO_2_ impregnated with HNO_3_; (**c**) Isolation of intermediates.

To gain some mechanistic insights into this oxidative cyclization, we first carried out several control experiments. When (2,2,6,6-tetramethylpiperidin-1-yl)oxyl (TEMPO) or butylated hydroxytoluene (BHT) was added to the oxidative cyclization of **3aa** under the standard reaction conditions (Scheme 8a), the desired product **4aa** was obtained in a yield up 81%, suggesting that radicals may not be involved in the catalytic cycle, in contrast to the earlier published cyclization of the Schiff’s base in the presence of Mn salt [78,79]. The slight decrease in yield is probably due to the deactivation of manganese oxide under the reducing action of TEMPO and BHT. In addition, a high yield of **4aa** was achieved, even when performing the reaction under a N_2_ atmosphere, demonstrating that aerobic oxygen is not the oxidizing agent in this transformation (Scheme 8a).

Subsequently, in order to get some information about possible reaction intermediates, we carried out the oxidative cyclization of **3aa** under various conditions. After several trials, we managed to isolate one of the possible intermediates, **4aa′**, in the presence of MnO_2_ impregnated with nitric acid [82] in CH_2_Cl_2_ at room temperature (Scheme 8b). The structure of the intermediate **4aa′** was supported by NMR and HRMS data. The ^1^H NMR spectrum comprises two dihydrotriazine proton doublets at 5.69 and 5.66 ppm with an SSCC (spin–spin coupling constant) of 10.8 Hz. Another intermediate **4aa′′** was detected by ^1^H NMR analysis (Please see Supporting Information for details, Section S6) in the crystallized reaction mixture when the reaction was carried out in the presence of a twofold excess of MnO_2_ (Scheme 8c). We ascribed the structure of dihydrotriazine to this compound since a single proton resonance at the *sp*^3^ carbon is observed in the ^1^H NMR spectrum.

**Scheme 9:**
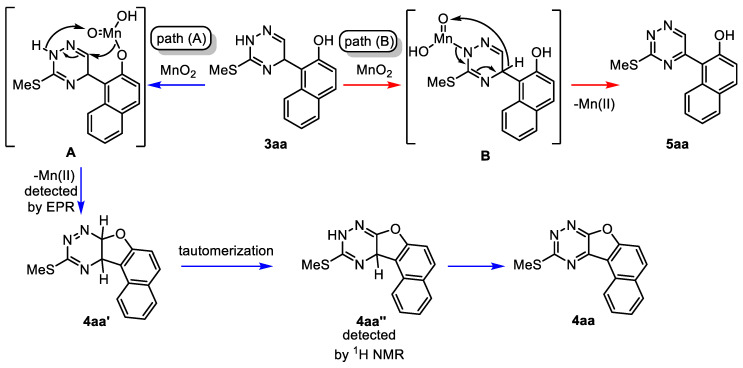
Proposed pathways of oxidation of dihydrotriazines. (**A**) Oxidative cyclization; (**B**) Regular aromatization.

After summarizing these preliminary mechanistic studies, a plausible reaction mechanism of the oxidative cyclization has been postulated (Scheme 9). The reaction may proceed through two different pathways: path a and path b. In path a, the reaction starts with the formation of an O-coordinated complex **A**, which agrees well with the oxidation of alcohol to aldehyde in the presence of MnO_2_ [94]. Then, complex **A** undergoes intramolecular nucleophilic addition to generate an intermediate **4aa′** with the elimination of Mn(II) species detected by an EPR experiment (Please see Supporting Information for details, Section S6). Then, the quick tautomerization of **4aa′** leads to the intermediate **4aa′′**, which is aromatized with the second equivalent of MnO_2,_ as well as with 1,4-dihydropyridine [95,96,97,98] or 1,4-dihydrotriazine [71,99], to give the final product **4aa**. On the other hand, if we consider path b, at the first step, MnO_2_ may coordinate with the nitrogen atom of the triazine core, leading to *N*-coordinated complex **B**, which is also aromatized with the formation of biaryl product **5aa**. Thus, the formation of the final product depends on the position of the initial coordination of the manganese dioxide, through which the reaction can proceed through the regular aromatization of dihydrotriazine (path A) or through the path of oxidative cyclization (path A).

**Figure 2:**
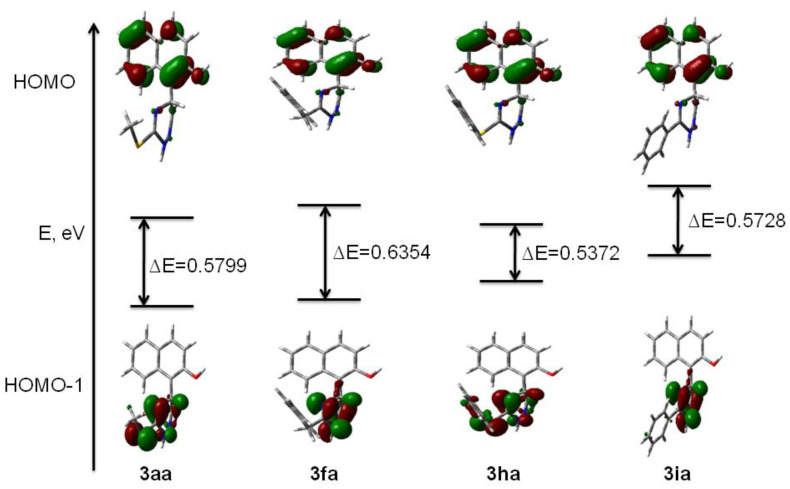
Localizations of electron density on nitrogen and oxygen atoms for compounds **3aa**, **3fa**, **3ha**, and **3ia**.

In order to rationalize the regioselectivity of pathways of the products’ formation (**4** vs. **5**) we have performed a series of DFT calculations of the electron density of HOMO and HOMO-1 in the compounds **3aa**, **3fa**, **3ha** and **3ia** (Figure 2). The results show that the electron density on the oxygen atom of the hydroxyl group is comparable with the one on the nitrogen of the triazine core in compounds **3aa** and **3ha**. However, the larger energy gap between HOMO and HOMO-1 of **3aa** compared with the energy gap in **3ha** increased the regioselectivity of the formation of O-coordinated manganese ester. In the case of compound **3ia**, the localization of the orbitals on the triazine N2 nitrogen (HOMO-1) was higher than those on the phenol oxygen (HOMO). So, the reaction proceeds partially via the aromatization of dihydrotriazine, rather than through oxidative cyclization. As follows from Figure 2, the important role of the alkylthio group is that it reduces the electron density at the nitrogen atom of dihydrotriazine, which leads to a reaction at the phenolic oxygen atom. Therefore, these results suggest that MnO_2_ may coordinate with either oxygen or nitrogen atoms, depending on the delocalization of the electron density of HOMO and HOMO-1 on the corresponding oxygen or nitrogen atom, and the energy gap between these orbitals.

## 3. Materials and Methods

**General Information:** All commercially available chemicals were used without further purifications. ^1^H NMR (400 MHz) and ^13^C NMR (101 MHz) spectra were registered on a Bruker DRX-400 Avance spectrometer with DMSO-*d_6_* or CDCl_3_ as the solvent at ambient temperature. Chemical shifts are reported in ppm, and coupling constants are given in Hz. Data for ^1^H NMR are recorded as follows: chemical shift (ppm), multiplicity (s, singlet; d, doublet; t, triplet; q, quartet; quin, quintet; sex, sextet; m, multiplet; br s, broad signal), integration and coupling constant (Hz). High-resolution mass spectra were recorded on an Agilent UHPLC/MS Accurate-Mass Q-TOF 1290/6545. EPR spectra were obtained using a Bruker Elexsys E500 CW-EPR spectrometer (modulation amplitude was set as 0.3 mT). The simulation of the EPR spectra was performed using the package EasySpin 5.2 software [100]. Molecular geometry optimization and the calculation of energies of molecules were carried out in the gas phase using the B3LYP DFT functional [101] with a 6-311 + G (d, p) basis set [102] according to [103] in Gaussian09 [104]. The plots of electron densities of molecular orbitals were obtained using the GaussView 6.0 software [105]. X-ray analysis for compound **5fa** was executed on an Xcalibur 3 diffractometer (MoKα radiation, graphite monochromator, 295(2) K, φ- and ω-scanning with a step of 1°). Thin-layer chromatography (TLC) was performed on a silica gel-coated glass slide (Merck, Silica gel G for TLC). Column chromatography was carried out on silica gel (60 Å, 0.035−0.070 mm). Images of ^1^H and ^13^C NMR spectra are provided on pages S26–S81 of the Supplementary Materials.

### 3.1. Synthesis of S-substituted 3-thio-1,2,4-triazines **1a-f**

S-substituted 3-thio-1,2,4-triazines **1a-f** were prepared from the corresponding salt of S-substituted isothiosemicarbazide (2 mmol) and glyoxal solution according to the following procedure [106].

A solution of 40% glyoxal (8 mmol, 1160 mg) and NaHCO_3_ (5 mmol, 420 mg) in ice water (40 mL) was added to a solution of S-substituted isothiosemicarbazide hydrogen iodide (2 mmol) dissolved in ice water (40 mL). The reaction mixture was stirred for 15 min; during that time, the evolution of gas (CO_2_) was observed. The reaction mixture was left in the fridge overnight and the aqueous solution was extracted with chloroform. The combined organic layer was washed with 10% oxalic acid, dried over anhydrous Na_2_SO_4_, filtered, and concentrated in vacuo to obtain oil or a solid triazine compound.

#### 3.1.1. Synthesis of 3-(phenylthio)-1,2,4-triazine **1h**

Compound **1h** was prepared via the oxidation of **1a** with *m*CPBA using a modified procedure [107] followed by the treatment of compound **1a′** with thiophenol.

*m*CPBA (11.6 g, 77%, 52 mmol) and anhydrous Na_2_SO_4_ (4.0 g) were successively added to DCM (60 mL); the mixture was stirred for 15 min and then filtered and the filter cake was washed with 10 mL of DCM to obtain a clear dichloromethane solution of *m*CPBA. A dichloromethane solution of 3-methylthio-1,2,4-triazine **1a** (3.0 g, 23.6 mmol) was added to this dichloromethane solution of *m*CPBA at −10 °C with stirring. The reaction mixture was allowed to heat to ambient temperature and then stirred for an additional 3 h. Dichloromethane was evaporated under reduced pressure to obtain a dry mixture of 3-(methylsulfonyl)-1,2,4-triazine **1a′** and *m*-chlorobenzoic acid. The mixture was dissolved in pyridine (40 mL) and thiophenol (5.3 mL, 5.72 g, 52 mmol) was added after. After 24 h the mixture was evaporated in vacuo, and the residue was treated with a mixture of dichloromethane and aqueous NaHCO_3_. The organic layer was evaporated, yielding pure compound **1h**.

#### 3.1.2. Synthesis of 3-phenyl- and 3-(4-methoxyphenyl)-1,2,4-triazines **1i** and **1j**

Compounds **1i** and **1j** were prepared according to the published procedure [108]. The spectroscopic data for compound **1i** are in agreement with the literature [109].

#### 3.1.3. Synthesis of 3-methyl- and 3-benzyl-1,2,4-triazine **1k** and **1l**

Compounds **1k** and **1l** were prepared according to the known procedure [110]. The spectroscopic data of compounds **1k** are in agreement with the published data [110].

#### 3.1.4. Synthesis of 4-(1,2,4-triazin-3-yl)morpholine **1m**

Compound **1m** was prepared according to the published procedure [111].

### 3.2. General Procedure for the Synthesis of Dihydrotriazines **3**

#### 3.2.1. Method A

To a stirred solution of triazine **1a-j** (1 mmol, 1 equiv.) and 2-naphthol **2a,f,g** (1 mmol, 1 equiv.) in acetic acid (4 mL), we added a methanesulfonic acid (195 μL, 3 mmol, 3 equiv.). The resulting mixture was stirred at room temperature for 1-5 h. The progress of the reaction was monitored using TLC. After the completion of the reaction, the reaction mixture was diluted with water (20 mL), neutralized with aq. NaHCO_3_ solution and extracted with AcOEt (3 × 10 mL). The combined organic phase was dried over anhydrous Na_2_SO_4_, filtered, and concentrated under reduced pressure. The residue was purified by silica gel chromatography or recrystallization from the corresponding solvent to afford product **3**.

#### 3.2.2. Method B

To a stirred solution of triazine **1a** (1 mmol, 1 equiv.) and 2-naphthol **2b-e** (1 mmol, 1 equiv.) in methanol (4 mL) we added BF_3_^.^OEt_2_ (985 μL, 8 mmol, 8 equiv.) and the resulting mixture was refluxed for 5 h. After cooling the methanol was evaporated under reduced pressure, and the residue was dissolved in AcOEt (10 mL) and washed with 5% aq. NaHCO_3_ solution (50 mL). The organic layer was dried over anhydrous Na_2_SO_4_, filtered, and evaporated under reduced pressure. The crude product was recrystallized from MeCN to obtain the product **3ab-3ae**.

### 3.3. General Procedure for the Synthesis of Naphthofuro-Fused Triazines **4**

To a stirred solution of **3** (0.2 mmol, 1 equiv.) in CHCl_3_ (3 mL), MnO_2_ (52 mg, 0.6 mmol, 3 equiv.) was added in one portion. The resulting mixture was stirred at 50 °C for 3 h. The completion of the reaction was monitored by TLC. The reaction mixture was then cooled to room temperature; the MnO_2_ was filtered and the filter cake was washed with CHCl_3_ (3 × 10 mL). The combined organic phase was concentrated under reduced pressure. The residue was purified by chromatography on silica gel or recrystallization to afford the pure product **4**.

#### 3.3.1. 10-(Methylthio)naphtho[1′,2′:4,5]furo[3,2-*e*][1,2,4]triazine **4aa**

Pale yellow needles after recrystallization from MeCN. Yield 51 mg, 95%; m.p. 185–187 °C. ^1^H NMR (CDCl_3_): 8.78–8.66 (m, 1H, H-1), 8.20–8.06 (m, 1H, H-5), 7.97–7.86 (m, 1H, H-4), 7.76–7.52 (m, 3H, H-2, H-3, H-6), 2.79 (s, 3H, SCH_3_); ^13^C NMR (CDCl_3_): 169.8, 160.0, 158.6, 143.7, 137.0, 130.5, 129.6, 129.2, 128.7, 126.7, 124.7, 112.7, 112.6, 14.8. Anal. Calcd. For C_14_H_9_N_3_OS: C, 62.91; H, 3.39; N, 15.72%; found: C, 62.96; H, 3.47; N, 15.79%.

#### 3.3.2. 10-(Ethylthio)naphtho[1′,2′:4,5]furo[3,2-*e*][1,2,4]triazine **4ba**

Pale yellow needles after recrystallization from MeCN. Yield 47 mg, 84%; m.p. 141–143 °C. ^1^H NMR (CDCl_3_): 8.97–8.88 (m, 1H, H-1), 8.28–8.21 (m, 1H, H-5), 8.06–7.99 (m, 1H, H-4), 7.87–7.75 (m, 2H, H-2, H-6), 7.70–7.62 (m, 1H, H-3), 3.44 (q, 2H, *J* = 7.3 Hz, SCH_2_), 1.56 (t, 3H, *J* = 7.3 Hz, CH_3_); ^13^C NMR (CDCl_3_): 169.6, 160.1, 158.7, 143.9, 137.0, 130.6, 129.6, 129.3, 128.9, 126.8, 124.8, 112.9, 112.7, 26.1, 14.4. Anal. Calcd. For C_15_H_11_N_3_OS: C, 64.04; H, 3.94; N, 14.94%; found: C, 64.12; H, 3.99; N, 14.85%.

#### 3.3.3. 10-(Butylthio)naphtho[1′,2′:4,5]furo[3,2-*e*][1,2,4]triazine **4ca**

Pale yellow needles after recrystallization from MeCN. Yield 53 mg, 85%; m.p. 121–123 °C. ^1^H NMR (CDCl_3_): 8.83–8.75 (m, 1H, H-1), 8.20–8.12 (m, 1H, H-5), 7.99–7.92 (m, 1H, H-4), 7.79–7.55 (m, 3H, H-2, H-3, H-6), 3.45–3.33 (m, 2H, SCH_2_), 1.96–1.83 (m, 2H, SCH_2_CH_2_), 1.68–1.53 (m, 2H, SCH_2_CH_2_CH_2_), 1.08–0.97 (m, 3H, CH_3_); ^13^C NMR (CDCl_3_): 169.8, 160.1, 158.7, 143.9, 137.1, 130.6, 129.6, 129.4, 128.9, 126.8, 124.9, 113.0, 112.8, 31.4, 31.3, 22.3, 13.9. Anal. Calcd. For C_17_H_15_N_3_OS: C, 66.00; H, 4.89; N, 13.58%; found: C, 66.09; H, 4.82; N, 13.50%.

#### 3.3.4. 10-((Cyclobutylmethyl)thio)naphtho[1′,2′:4,5]furo[3,2-*e*][1,2,4]triazine **4da**

Yellow needles after recrystallization from MeCN. Yield 57 mg, 89%; m.p. 154–156 °C. ^1^H NMR (CDCl_3_): 8.81–8.76 (m, 1H, H-1), 8.17 (d, 1H, *J* = 9.0 Hz, H-5), 7.98–7.93 (m, 1H, H-4), 7.78–7.72 (m, 1H, H-2), 7.70 (d, 1H, *J* = 9.0 Hz, H-6), 7.63–7.57 (m, 1H, H-3), 3.53–3.44 (m, 2H, SCH_2_), 2.90–2.80 (m, 1H, CH-1′), 2.29–2.18 (m, 2H, CH_2_-3′), 1.98–1.83 (m, 4H, CH_2_-2′, CH_2_-4′); ^13^C NMR (CDCl_3_): 169.7, 160.0, 158.7, 143.8, 137.0, 130.6, 129.6, 129.3, 128.8, 126.7, 124.8, 112.8, 112.7, 37.9, 34.7, 28.0, 18.2. Anal. Calcd. For C_18_H_15_N_3_OS: C, 67.27; H, 4.70; N, 13.07%; found: C, 67.35; H, 4.78; N, 13.12%.

#### 3.3.5. 10-(But-2-yn-1-ylthio)naphtho[1′,2′:4,5]furo[3,2-*e*][1,2,4]triazine **4ea**

Brown powder after purification by chromatography on silica gel using *n*-hexane-ethyl acetate (10:1). Yield 45 mg, 70%; m.p. 163–165 °C. ^1^H NMR (CDCl_3_): 8.88–8.83 (m, 1H, H-1), 8.22 (d, 1H, *J* = 9.0 Hz, H-5), 8.02–7.97 (m, 1H, H-4), 7.82–7.77 (m, 1H, H-2), 7.74 (d, 1H, *J* = 9.0 Hz, H-6), 7.67–7.61 (m, 1H, H-3), 4.15 (q, 2H, *J* = 2.5 Hz, SCH_2_), 1.84 (t, 3H, *J* = 2.5 Hz, CH_3_); ^13^C NMR (CDCl_3_): 168.4, 160.1, 158.8, 143.9, 137.3, 130.6, 129.8, 129.8, 128.9, 126.9, 124.9, 112.9, 112.7, 79.5, 73.7, 21.0, 3.9. Anal. Calcd. For C_17_H_11_N_3_OS: C, 66.87; H, 3.63; N, 13.76%; found: C, 66.80; H, 3.60; N, 13.86%.

#### 3.3.6. 10-(Benzylthio)naphtho[1′,2′:4,5]furo[3,2-*e*][1,2,4]triazine **4fa**

Yellow needles after recrystallization from MeCN. Yield 63 mg, 90%; m.p. 161–163 °C. ^1^H NMR (CDCl_3_): 8.82–8.73 (m, 1H, H-1), 8.17 (d, 1H, *J* = 9.1 Hz, H-5), 7.97–7.93 (m, 1H, H-4), 7.78–7.72 (m, 1H, H-3), 7.70 (d, 1H, *J* = 9.1 Hz, H-6), 7.26–7.56 (m, 3H, H-2, Ph), 7.38–7.32 (m, 2H, Ph), 7.30–7.25 (m, 1H, Ph), 4.66 (m, 1H, SCH_2_); ^13^C NMR (CDCl_3_): 169.0, 160.2, 158.8, 143.9, 137.2, 137.1, 130.6, 129.7, 129.4, 129.4, 128.9, 128.7, 127.6, 126.8, 124.9, 112.9, 112.7, 36.1. Anal. Calcd. For C_20_H_13_N_3_OS: C, 69.95; H, 3.82; N, 12.24%; found: C, 69.85; H, 3.76; N, 12.20%.

#### 3.3.7. 10-(Allylthio)naphtho[1′,2′:4,5]furo[3,2-*e*][1,2,4]triazine **4ga**

Pale yellow powder after recrystallization from MeCN. Yield 46 mg, 78%; m.p. 126–128 °C. ^1^H NMR (CDCl_3_): 8.83–8.74 (m, 1H, H-1), 8.17 (d, 1H, *J* = 9.1 Hz, H-5), 7.99–7.93 (m, 1H, H-4), 7.78–7.72 (m, 1H, H-3), 7.70 (d, 1H, *J* = 9.1 Hz, H-6), 7.64–7.57 (m, 1H, H-2), 6.13 (ddt, 1H, ^3^*J* = 6.9 Hz, ^3^*J*(cis) = 10.0 Hz, ^3^*J*(trans) = 17.0 Hz, CH-2′), 5.47 (dd, 1H, ^3^*J*(trans) = 16.9 Hz, *J* = 1.2 Hz, CH-3a′), 5.22 (d, 1H, ^3^*J*(cis) = 10.0 Hz, CH-3b′), 4.06 (d, 2H, ^3^*J* = 6.9 Hz, SCH_2_); ^13^C NMR (CDCl_3_): 168.9, 160.1, 158.7, 143.8, 137.1, 133.1, 130.6, 129.7, 129.3, 128.8, 126.8, 124.8, 118.7, 112.9, 112.7, 34.5. Anal. Calcd. For C_16_H_9_N_3_OS: C, 65.97; H, 3.11; N, 14.42%; found: C, 65.90; H, 3.18; N, 14.50%.

A mixture of **4ha** and **5ha** was separated by silica gel chromatography using *n*-hexane-ethyl acetate (17:1) to isolate **4ha** and *n*-hexane-ethyl acetate (10:1) to give **5ha**.

#### 3.3.8. 10-(Phenylthio)naphtho[1′,2′:4,5]furo[3,2-*e*][1,2,4]triazine **4ha**

Yellow powder. Yield 46 mg, 70%; m.p. 196–198 °C. ^1^H NMR (CDCl_3_): 8.58–8.53 (m, 1H, H-1), 8.22–8.17 (m, 1H, H-5), 8.00–7.95 (m, 1H, H-4), 7.83–7.77 (m, 2H, Ph), 7.75–7.67 (m, 2H, H-3, H-6), 7.64–7.58 (m, 1H, H-2), 7.56–7.50 (m, 3H, Ph); ^13^C NMR (CDCl_3_): 170.0, 160.3, 158.8, 144.0, 137.2, 135.8, 130.6, 129.7, 129.6, 129.5, 129.3, 129.2, 128.8, 126.8, 124.8, 113.0, 112.7. Anal. Calcd. For C_19_H_11_N_3_OS: C, 69.29; H, 3.37; N, 12.76%; found: C, 69.20; H, 3.45; N, 12.70%.

#### 3.3.9. 1-(3-(Phenylthio)-1,2,4-triazin-5-yl)naphthalen-2-ol **5ha**

Yellow powder. Yield 9 mg, 14%; m.p. 149–151 °C. ^1^H NMR (CDCl_3_): 11.01 (s, 1H, OH), 9.51 (s, 1H, H-6′), 8.09–8.03 (m, 1H, H-8), 7.84 (d, 1H, *J* = 9.0 Hz, H-4), 7.82–7.76 (m, 1H, H-5), 7.75–7.67 (m, 2H, Ph), 7.59–7.51 (m, 4H, Ph, H-7), 7.44–7.37 (m, 1H, H-6), 7.07 (d, 1H, *J* = 9.0 Hz, H-3); ^13^C NMR (CDCl_3_): 172.3, 160.8, 155.7, 146.2, 136.2, 136.0, 130.8 (2C), 130.3, 129.5, 129.2, 128.7, 126.9, 124.7, 123.2, 119.7, 108.9. Anal. Calcd. For C_19_H_13_N_3_OS: C, 68.86; H, 3.95; N, 12.68%; found: C, 68.77; H, 3.90; N, 12.60%.

A mixture of **4ia** and **5ia** was separated by chromatography using *n*-hexane-ethyl acetate (15:1) to give **4ia** and *n*-hexane-ethyl acetate (8:1) to give **5ia**.

#### 3.3.10. 10-Phenylnaphtho[1′,2′:4,5]furo[3,2-*e*][1,2,4]triazine **4ia**

Pale yellow solid. Yield 12 mg, 21%, m.p. 189–191 °C. Rf = 0.54 (ethyl acetate:hexane, 1:1). ^1^H NMR (CDCl_3_): 9.09–8.99 (m, 1H, H-1), 8.75–8.64 (m, 2H, Ph), 8.24–8.14 (m, 1H, H-5), 8.03–7.94 (m, 1H, H-4), 7.86–7.73 (m, 2H, H-2, H-6), 7.66–7.52 (m, 4H, H-3, Ph); ^13^C NMR (CDCl_3_): 161.5, 160.8, 158.5, 143.9, 136.7, 135.7, 131.2, 130.7, 129.6, 129.3, 129.1, 129.0, 128.5, 126.7, 124.9, 113.7, 112.8. Anal. Calcd. For C_19_H_11_N_3_O: C, 76.76; H, 3.73; N, 14.13%; found: C, 76.83; H, 3.70; N, 14.19%.

#### 3.3.11. 1-(3-Phenyl-1,2,4-triazin-5-yl)naphthalen-2-ol **5ia**

Yellow solid. Yield 31 mg, 52%, m.p. 204–206 °C. Rf = 0.39 (ethyl acetate:hexane, 1:1). ^1^H NMR (DMSO-d_6_): 12.45–12.16 (br s, 1H, OH), 9.77 (s, 1H, H-6′), 8.56–8.47 (m, 2H, Ph), 8.21–8.11 (m, 1H, H-8), 7.95 (d, 1H, *J* = 9.0 Hz, H-4), 7.90–7.83 (m, 1H, H-5), 7.67–7.55 (m, 4H, H-6, Ph), 7.49–7.42 (m, 1H, H-7), 7.28 (d, 1H, *J* = 9.0 Hz, H-3); ^13^C NMR (DMSO-*d_6_*): 161.3, 160.3, 155.9, 148.3, 135.8, 134.2, 132.4, 131.0, 129.6, 129.3 (2C), 128.7, 128.4, 124.7, 123.1, 119.6, 109.4. Anal. Calcd. For C_19_H_13_N_3_O: C, 76.24; H, 4.38; N, 14.04%; found: C, 76.32; H, 4.45; N, 14.12%.

A mixture of **4ja** and **5ja** was separated by silica gel chromatography using *n*-hexane-ethyl acetate (17:1) to isolate **4ja** and *n*-hexane-ethyl acetate (7:1) to give **5ja**.

#### 3.3.12. 10-(4-Methoxyphenyl)naphtho[1′,2′:4,5]furo[3,2-*e*][1,2,4]triazine **4ja**

Pale yellow solid. Yield 18 mg, 28%; m.p. 205–207 °C. Rf = 0.53 (ethyl acetate:hexane, 1:1). ^1^H NMR (CDCl_3_): 9.05–8.99 (m, 1H, H-1), 8,64 (d, 2H, *J* = 8.8 Hz, Ph), 8.19 (d, 1H, *J* = 9.0 Hz, H-5), 8.02–7.94 (m, 1H, H-4), 7.85–7.78 (m, 1H, H-3), 7.76 (d, 1H, *J* = 9.0 Hz, H-6), 7.66–7.58 (m, 1H, H-2), 7.08 (d, 2H, *J* = 8.8 Hz, Ph), 3.93 (s, 3H, OCH_3_); ^13^C NMR (CDCl_3_): 162.3, 161.4, 160.6, 158.4, 143.9, 136.5, 130.7, 130.1, 129.5, 129.3, 129.1, 128.3, 126.7, 124.9, 114.3, 113.7, 112.8, 55.6. Anal. Calcd. For C_20_H_13_N_3_O_2_: C, 73.38; H, 4.00; N, 12.84%; found: C, 73.30; H, 3.92; N, 12.94%.

#### 3.3.13. 1-(3-(4-Methoxyphenyl)-1,2,4-triazin-5-yl)naphthalen-2-ol **5ja**

Yellow solid. Yield 31 mg, 47%; m.p. 178–180 °C. Rf = 0.41 (ethyl acetate:hexane, 1:1). ^1^H NMR (DMSO-d_6_): 12.33–12.08 (br s, 1H, OH), 9.59 (s, 1H, H-6′), 8.38 (d, 2H, *J* = 8.7 Hz, Ph), 8.09–8.01 (m, 1H, H-8), 7.87–7.81 (m, 1H, H-4), 7.80–7.72 (m, 1H, H-5), 7.54–7.45 (m, 1H, H-7 or H-6), 7.39–7.30 (m, 1H, H-6 or H-7), 7.22–7.13 (m, 1H, H-3), 7.03–6.95 (d, 2H, *J* = 8.7 Hz, Ph), 3.83 (s, 3H, OCH_3_); ^13^C NMR (DMSO-*d_6_*): 162.5, 156.8, 154.1, 151.0, 135.1, 132.6, 132.2, 131.6, 129.1, 128.4, 128.0, 127.8, 127.6, 123.4, 123.1, 118.1, 113.9, 55.7. Anal. Calcd. For C_20_H_15_N_3_O_2_: C, 72.94; H, 4.59; N, 12.76%; found: C, 72.83; H, 4.65; N, 12.70%.

#### 3.3.14. 6-Methoxy-10-(methylthio)naphtho[1′,2′:4,5]furo[3,2-*e*][1,2,4]triazine **4ab**

Yellow powder after recrystallization from MeCN. Yield 48 mg, 80%; m.p. 183–185 °C. ^1^H NMR (CDCl_3_): 8.55–8.51 (m, 1H, H-4 or H-1), 7.71–7.66 (m, 1H, H-1 or H-4), 7.55–7.50 (m, 1H, H-3 or H-2), 7.79–7.50 (m, 1H, H-2 or H-3), 7.32 (s, 1H, H-5), 4.13 (s, 3H, OCH_3_), 2.78 (s, 3H, SCH_3_); ^13^C NMR (CDCl_3_): 169.9, 159.9, 149.9, 145.0, 143.3, 131.2, 127.6, 126.9, 126.8, 124.3, 123.6, 114.1, 113.3, 56.5, 14.8. Anal. Calcd. For C_15_H_11_N_3_O_2_S: C, 60.59; H, 3.73; N, 14.13%; found: C, 60.67; H, 3.65; N, 14.04%.

#### 3.3.15. 2-Methoxy-10-(methylthio)naphtho[1′,2′:4,5]furo[3,2-*e*][1,2,4]triazine **4ac**

Yellow powder after recrystallization from MeCN. Yield 50 mg, 84%; m.p. 219–221 °C. ^1^H NMR (CDCl_3_): 8.22–8.19 (m, 1H, H-1), 8.14–8.10 (m, 1H, H-4), 7.88 (d, 1H, *J* = 8.9 Hz, H-4), 7.57 (d, 1H, *J* = 8.9 Hz, H-3), 7.27–7.22 (m, 1H, H-3), 4.06 (s, 3H, OCH_3_), 2.81 (s, 3H, SCH_3_); ^13^C NMR (CDCl_3_): 169.6, 161.0, 160.2, 159.4, 144.1, 136.8, 131.0, 125.7, 118.8, 112.0, 109.8, 104.2, 96.3, 55.8, 14.8. Anal. Calcd. For C_15_H_11_N_3_O_2_S: C, 60.59; H, 3.73; N, 14.13%; found: C, 60.50; H, 3.66; N, 14.10%.

#### 3.3.16. 10-(Methylthio)naphtho[1′,2′:4,5]furo[3,2-*e*][1,2,4]triazin-2-ol **4ad**

According to the general procedure, in the mixture of CHCl_3_:EtOH (4:1) as solvent, **4ad** was obtained as yellow powder after recrystallization from EtOH. Yield 38 mg, 68%; m.p. 282–284 °C. ^1^H NMR (DMSO-d_6_): 10.61 (s, 1H, OH), 8.30 (d, 1H, *J* = 8.9 Hz, H-6), 8.03–7.97 (m, 2H, H-1, H-4), 7.70 (d, 1H, *J* = 8.9 Hz, H-4), 7.57 (d, 1H, *J* = 8.9 Hz, H-3), 7.19–7.14 (m, 1H, H-3), 2.76 (s, 3H, SCH_3_); ^13^C NMR (DMSO-d_6_): 168.0, 159.9, 159.1, 159.1, 144.0, 137.4, 131.6, 130.2, 124.4, 118.4, 110.5, 108.9, 106.5, 14.1. Anal. Calcd. For C_14_H_9_N_3_O_2_S: C, 59.35; H, 3.20; N, 14.83%; found: C, 59.25; H, 3.15; N, 14.75%.

#### 3.3.17. 3-Methoxy-10-(methylthio)naphtho[1′,2′:4,5]furo[3,2-*e*][1,2,4]triazine **4ae**

Yellow powder after recrystallization from MeCN. Yield 49 mg, 82%; m.p. 214–216 °C. ^1^H NMR (CDCl_3_): 8.83 (d, 1H, *J* = 8.9 Hz, H-1), 8.13 (d, 1H, *J* = 9.0 Hz, H-5), 7.74 (d, 1H, *J* = 9.0 Hz, H-6), 7.47 (dd, 1H, *J* = 8.9 Hz, *J* = 2.5 Hz, H-2), 7.33 (d, 1H, *J* = 2.5 Hz, H-4), 3.98 (s, 3H, OCH_3_), 2.83 (s, 3H, SCH_3_); ^13^C NMR (CDCl_3_): 169.7, 160.2, 158.3, 157.6, 143.9, 135.9, 132.2, 126.3, 123.8, 121.7, 113.1 (2C), 108.2, 55.6, 14.9. Anal. Calcd. For C_15_H_11_N_3_O_2_S: C, 60.59; H, 3.73; N, 14.13%; found: C, 60.65; H, 3.82; N, 14.10%.

#### 3.3.18. 3-Bromo-10-(methylthio)naphtho[1′,2′:4,5]furo[3,2-*e*][1,2,4]triazine **4af**

Yellow powder after recrystallization from toluene. Yield 53 mg, 75%; m.p. 244–246 °C. ^1^H NMR (CDCl_3_): 8.86 (d, 1H, *J* = 8.7 Hz, H-1), 8.23 (d, 1H, *J* = 1.8 Hz, H-4), 8.19 (d, 1H, *J* = 9.1 Hz, H-5), 7.92 (dd, 1H, *J* = 8.7 Hz, *J* = 1.8 Hz, H-2), 7.85 (d, 1H, *J* = 9.1 Hz, H-6), 2.85 (s, 3H, SCH_3_); ^13^C NMR (CDCl_3_): 170.2 160.3, 158.7, 143.6, 136.0, 133.0, 132.0, 131.5, 127.5, 126.6, 120.8, 114.1, 113.2, 14.9. Anal. Calcd. For C_14_H_8_BrN_3_OS: C, 48.57; H, 2.33; N, 12.14%; found: C, 48.50; H, 2.26; N, 12.06%.

#### 3.3.19. 10-(Methylthio)naphtho[1′,2′:4,5]furo[3,2-*e*][1,2,4]triazine-3-carbonitrile **4ag**

Yellow solid after recrystallization from MeCN. Yield 54 mg, 91%; m.p. 283–285 °C. ^1^H NMR (CDCl_3_): 9.09 (d, 1H, *J* = 8.5 Hz, H-1), 8.44 (d, 1H, *J* = 1.5 Hz, H-4) 8.33 (d, 1H, *J* = 9.1 Hz, H-5), 7.99 (dd, 1H, *J* = 8.5 Hz, *J* = 1.5 Hz, H-2), 7.96 (d, 1H, *J* = 9.1 Hz, H-6), 2.85 (s, 3H, SCH_3_); ^13^C NMR (CDCl_3_): 170.7, 160.4, 159.8, 143.3, 136.8, 134.9, 130.9, 130.6, 129.9, 126.3, 118.6, 115.1, 113.4, 110.7, 14.9. Anal. Calcd. For C_15_H_8_N_4_OS: C, 61.63; H, 2.76; N, 19.17%; found: C, 61.72; H, 2.86; N, 19.22%.

#### 3.3.20. Synthesis of 10-alkyl naphtho[1′,2′:4,5]furo[3,2-*e*][1,2,4]triazines **4ka** and **4la**

To a stirred solution of corresponding triazine **1k** or **1l** (1 mmol, 1 equiv.) in acetic acid (4 mL), 2-naphthol **2a** (144 mg, 1 mmol, 1 equiv.) was added. Then the mixture was stirred at room temperature for 5 h, concentrated under reduced pressure, dissolved in CHCl_3_ (10 mL) and washed with saturated aq. NaHCO_3_ solution (10 mL). The organic layer was dried over anhydrous Na_2_SO_4_ and filtered. To the organic phase, MnO_2_ (261 mg, 3.0 mmol, 3 equiv.) was added in one portion and the mixture was stirred at 50 °C for 3 h. The reaction mixture was then cooled to room temperature. MnO_2_ was filtered and washed with CHCl_3_ (3 × 10 mL). The combined organic phase was concentrated under reduced pressure to give a mixture of **4** and **5**, which was separated by chromatography on silica gel using a mixture of *n*-hexane-ethyl acetate as the eluent.

A mixture of **4ka** and **5ka** was separated by chromatography on silica gel using *n*-hexane-ethyl acetate (25:1) to isolate **4ka** and *n*-hexane-ethyl acetate (8:1) to give **5ka**.

#### 3.3.21. 10-Methylnaphtho[1′,2′:4,5]furo[3,2-*e*][1,2,4]triazine **4ka**

Yellow solid. Yield 113 mg, 48%; m.p. 190–192 °C. Rf = 0.46 (ethyl acetate:hexane, 1:1). ^1^H NMR (CDCl_3_): 8.98–8.92 (m, 1H, H-1), 8.24–8.17 (m, 1H, H-5), 8.04–7.98 (m, 1H, H-4), 7.84–7.72 (m, 2H, H-2, H-6), 7.68–7.59 (m, 1H, H-3), 3.08 (s, 3H, CH_3_); ^13^C NMR (CDCl_3_): 164.4, 160.6, 158.4, 144.0, 136.7, 130.7, 129.5, 129.3, 129.0, 126.7, 124.9, 113.4, 112.8, 23.9. Anal. Calcd. For C_14_H_9_N_3_O: C, 71.48; H, 3.86; N, 17.86%; found: C, 71.39; H, 3.93; N, 17.92%.

#### 3.3.22. 1-(3-Methyl-1,2,4-triazin-5-yl)naphthalen-2-ol **5ka**

Pale yellow solid. Yield 45 mg, 19%; m.p. 168–170 °C. Rf = 0.25 (ethyl acetate:hexane 1:1). ^1^H NMR (DMSO-*d_6_*): 10.46 (s, 1H, OH), 9.44 (s, 1H, H-6′), 8.01–7.96 (m, 1H, H-4), 7.92–7.85 (m, 1H, H-8 or H-5), 7.73–7.67 (m, 1H, H-5 or H-8), 7.45–7.29 (m, 3H, H-3, H-6, H-7), 2.85 (s, 3H, CH_3_); ^13^C NMR (DMSO-*d_6_*): 166.2, 156.4, 153.8, 150.2, 132.3, 132.1, 128.3, 127.9, 127.4, 123.3, 123.2, 118.1, 113.8, 23.6. Anal. Calcd. For C_14_H_11_N_3_O: C, 70.87; H, 4.67; N, 17.71%; found: C, 70.77; H, 4.72; N, 17.80%.

A mixture of **4la** and **5la** was separated by chromatography on silica gel using *n*-hexane-ethyl acetate (17:1) to give **4la,** and *n*-hexane-ethyl acetate (10:1) to give **5la**.

#### 3.3.23. 10-Benzylnaphtho[1′,2′:4,5]furo[3,2-*e*][1,2,4]triazine **4la**

Pale yellow solid. Yield 109 mg, 35%; m.p. 226–228 °C. Rf = 0.57 (ethyl acetate:hexane, 1:1). ^1^H NMR (DMSO-*d_6_*): 8.89–8.82 (m, 1H, H-1), 8.49 (d, 1H, *J* = 9.0 Hz H-5), 8.25–8.20 (m, 1H, H-4), 8.06 (d, 1H, *J* = 9.0 Hz H-6), 7.93–7.87 (m, 1H, H-3 or H-2), 7.74–7.69 (m, 1H, H-2 or H-3), 7.49–7.44 (m, 2H, Ph), 7.37–7.32 (m, 2H, Ph), 7.27–7.22 (m, 1H, Ph), 4.62 (s, 2H, CH_2_); ^13^C NMR (DMSO-*d_6_*): 165.0, 160.2, 158.0, 143.9, 138.1, 137.0, 130.2, 129.5 (2C), 129.0 (2C), 128.3 (2C), 128.1, 126.4 (2C), 123.7, 112.9, 112.5, 42.7. Anal. Calcd. For C_20_H_13_N_3_O: C, 77.16; H, 4.21; N, 13.50%; found: C, 77.25; H, 4.30; N, 13.57%.

#### 3.3.24. 1-(3-Methyl-1,2,4-triazin-5-yl)naphthalen-2-ol **5la**

Pale yellow solid. Yield 110 mg, 35%; m.p. 155–157 °C. Rf = 0.34 (ethyl acetate:hexane, 1:1). ^1^H NMR (DMSO-*d_6_*): 10.55 (s, 1H, OH), 9.49 (s, 1H, H-6′), 8.01–7.94 (m, 1H, H-4), 7.90–7.84 (m, 1H, H-8 or H-5), 7.63–7.55 (m, 1H, H-5 or H-8), 7.43–7.22 (m, 8H, H-3, H-6, H-7, Ph), 4.47 (s, 2H, CH_2_); ^13^C NMR (DMSO-*d_6_*): 167.9, 156.7, 154.0, 150.6, 137.7, 132.6, 132.0, 129.2, 128.5, 128.3, 128.0, 127.3, 126.6, 123.4, 123.1, 118.0, 113.5, 43.0. Anal. Calcd. For C_20_H_15_N_3_O: C, 76.66; H, 4.83; N, 13.41%; found: C, 76.75; H, 4.74; N, 13.48%.

#### 3.3.25. 10-Morpholinonaphtho[1′,2′:4,5]furo[3,2-*e*][1,2,4]triazine **4ma**

To a stirred solution of 4-(1,2,4-triazin-3-yl)morpholine **1m** (1 mmol, 1 equiv.) and 2-naphthol **2a** (1 mmol, 1 equiv.) in methanol (4 mL) BF_3_^.^OEt_2_ (370 μL, 3 mmol, 3 equiv.) was added dropwise, and the resulting mixture was refluxed for 3 h. After cooling to room temperature the methanol was evaporated under reduced pressure, and the residue was dissolved in CHCl_3_ (10 mL) and washed with aq. NaHCO_3_. Then, the organic layer was dried over Na_2_SO_4_ and filtered. To the resulting solution MnO_2_ (261 mg, 3 mmol, 3 equiv.) was added in one portion and the mixture was stirred at 50 °C for 3 h. The reaction mixture was cooled to room temperature. MnO_2_ was filtered and washed with CHCl_3_ (3 × 10 mL). The combined organic phase was concentrated under reduced pressure, and the residue was crystallized from MeCN to afford pure **4ma**. Yellow powder. Yield 225 mg, 75%; m.p. 230–232 °C. ^1^H NMR (CDCl_3_): 8.87–8.82 (m, 1H, H-1), 8.19 (d, 1H, *J* = 9.1 Hz, H-5), 8.02–7.96 (m, 1H, H-4), 7.80–7.73 (m, 1H, H-3), 7.70 (d, 1H, *J* = 9.1 Hz, H-6), 7.65–7.58 (m, 1H, H-2), 4.07–4.00 (m, 4H, morpholine), 3.95–3.88 (m, 4H, morpholine); ^13^C NMR (CDCl_3_): 161.1, 158.9, 157.7, 144.2, 136.0, 130.5, 129.3, 129.3, 129.2, 126.3, 124.6, 113.5, 113.0, 67.0, 45.1. Anal. Calcd. For C_17_H_14_N_4_O_2_: C, 66.66; H, 4.61; N, 18.29%; found: C, 66.75; H, 4.54; N, 18.36%.

#### 3.3.26. Synthesis of Benzofuro-Fused Triazines **4ah** and **4ai**

To a solution of triazine **1a** (127 mg, 1 mmol) in TFA (4 mL), a corresponding phenol **2h** or **2i** (1 mmol) was added, and the resulting mixture was stirred at room temperature for 24 h. The completion of the reaction was monitored by TLC. Then, the reaction mixture was concentrated under reduced pressure. The residue was dissolved in CHCl_3_ (10 mL) and washed with 5% aq. NaHCO_3_. The organic layer was dried over Na_2_SO_4_ and filtered. MnO_2_ (52 mg, 0.6 mmol, 3 equiv.) was added to the resulting solution in one portion and the mixture was stirred at 50 °C for 3 h, cooled to room temperature, and MnO_2_ was filtered and the filter cake washed with CHCl_3_ (3 × 10 mL). The combined organic phase was concentrated under reduced pressure. The residue was purified by silica gel chromatography to afford the pure product, using *n*-hexane-ethyl acetate (80:1) to afford **4ah** or **4ai** and *n*-hexane-ethyl acetate (40:1) to give **5ah** or **5ai**.

#### 3.3.27. 6,8-Di-tert-butyl-3-(methylthio)benzofuro[3,2-*e*][1,2,4]triazine **4ah**

Yellow powder. Yield 167 mg, 51%; m.p. 105–107 °C. Rf = 0.67 (ethyl acetate:hexane, 1:1). ^1^H NMR (CDCl_3_): 8.08 (d, 1H, *J* = 1.8 Hz H-5), 7.78 (d, 1H, *J* = 1.8 Hz, H-7), 2.79 (s, 3H, SCH_3_), 1.57 (s, 9H, C(CH_3_)_3_), 1.41 (s, 9H, C(CH_3_)_3_); ^13^C NMR (CDCl_3_): 169.1, 160.7, 155.8, 148.6, 144.2, 136.1, 130.3, 118.8, 118.0, 35.4, 35.0, 31.7, 29.8, 14.8. Anal. Calcd. For C_18_H_23_N_3_OS: C, 65.62; H, 7.04; N, 12.75%; found: C, 65.72; H, 7.10; N, 12.82%.

#### 3.3.28. 2,4-Di-tert-butyl-6-(3-(methylthio)-1,2,4-triazin-5-yl)phenol **5ah**

Pale yellow powder. Yield 67 mg, 20%; m.p. 113–115 °C. Rf = 0.64 (ethyl acetate:hexane, 1:1). ^1^H NMR (CDCl_3_): 12.73 (s, 1H, OH), 9.50 (s, 1H, H-6′), 7.69 (d, 1H, *J* = 2.3 Hz, H-5), 7.57 (d, 1H, *J* = 2.3 Hz, H-3), 2.75 (s, 3H, SCH_3_), 1.46 (s, 9H, C(CH_3_)_3_), 1.35 (s, 9H, C(CH_3_)_3_); ^13^C NMR (CDCl_3_): 170.1, 160.0, 156.0, 141.8, 141.6, 139.0, 130.9, 121.2, 113.0, 35.5, 34.6, 31.5, 29.5, 14.1. Anal. Calcd. For C_18_H_25_N_3_OS: C, 65.22; H, 7.60; N, 12.68%; found: C, 65.31; H, 7.51; N, 12.74%.

#### 3.3.29. 6-(tert-Butyl)-3-(methylthio)benzofuro[3,2-*e*][1,2,4]triazine **4ai**

Yellow powder. Yield 30 mg, 11%; m.p. 136–138 °C. Rf = 0.64 (ethyl acetate:hexane, 1:1). ^1^H NMR (CDCl_3_): 8.23 (d, 1H, *J* = 1.8 Hz, H-5), 7.88 (dd, 1H, *J* = 1.8 Hz, *J* = 8.9 Hz, H-7), 7.61 (d, 1H, *J* = 8.9 Hz, H-8), 2.78 (s, 3H, SCH_3_), 1.42 (s, 9H, C(CH_3_)_3_); ^13^C NMR (CDCl_3_): 169.3, 161.0, 157.2, 149.0, 144.1, 133.5, 120.6, 118.5, 112.8, 35.3, 31.6, 14.8. Anal. Calcd. For C_14_H_15_N_3_OS: C, 61.52; H, 5.53; N, 15.37%; found: C, 61.59; H, 5.44; N, 15.30%.

#### 3.3.30. 4-(tert-Butyl)-2-(3-(methylthio)-1,2,4-triazin-5-yl)phenol **5ai**

Yellow powder. Yield 120 mg, 43%; m.p. 123–125 °C. Rf = 0.59 (ethyl acetate:hexane, 1:1). ^1^H NMR (CDCl_3_): 11.97 (s, 1H, OH), 9.49 (s, 1H, H-6′), 7.80 (d, 1H, *J* = 2.0 Hz, H-3), 7.53 (dd, 1H, *J* = 2.0 Hz, *J* = 8.8 Hz, H-5), 6.99 (d, 1H, *J* = 8.8 Hz, H-8), 2.71 (s, 3H, SCH_3_), 1.34 (s, 9H, C(CH_3_)_3_); ^13^C NMR (CDCl_3_): 170.6, 160.3, 155.3, 142.9, 141.2, 133.5, 123.2, 119.1, 113.2, 34.4, 31.4, 14.0. Anal. Calcd. For C_14_H_17_N_3_OS: C, 61.06; H, 6.22; N, 15.26%; found: C, 61.13; H, 6.29; N, 15.16%.

#### 3.3.31. 40 mmol Scaled Synthesis of **3ad**

To a stirred solution of triazine **1a** (5.10 g, 40 mmol, 1 equiv.) and 2,7-dihydroxynaphthalene **2d** (6.40 g, 40 mmol, 1 equiv.) in methanol (40 mL), BF_3_.OEt_2_ (40 mL, 320 mmol, 8 equiv.) was added dropwise and the resulting mixture was refluxed for 8 h. After cooling the methanol was evaporated under reduced pressure, and then the residue was treated with AcOEt (30 mL) and stirred for 15 min. The precipitate formed was filtered and washed with AcOEt (10 mL). The precipitate was suspended in AcOEt and the resulting mixture was washed with aq. NaHCO_3_ solution. The organic layer was dried over anhydrous Na_2_SO_4_, filtered, and evaporated under reduced pressure to give **3ad**. The **3ad** was dissolved in a mixture of CHCl_3_:EtOH (4:1, 300 mL). To the resulting solution, MnO_2_ (10.44 g, 120 mmol, 3 equiv.) was added in one portion. The resulting mixture was stirred at 50 °C for 6 h. The completion of the reaction was monitored by TLC. The reaction mixture was then cooled to room temperature, and the MnO_2_ was filtered and washed with CHCl_3_ (3 × 50 mL). The combined organic phase was concentrated under reduced pressure. The residue was recrystallized in EtOH to give pure **4ad** (9.62 g, 85% in two steps).

### 3.4. Further Modifications of Compound **4aa**

#### 3.4.1. 10-(4-(Carbazol-9-yl)phenyl)naphtho[1′,2′:4,5]furo[3,2-*e*][1,2,4]triazine 7

To a solution of 10-(methylthio)naphtho[1′,2′:4,5]furo[3,2-*e*][1,2,4]triazine **4aa** (100 mg, 1 equiv.) in dry THF (5 mL), we added CuTC (249 mg, 3.5 equiv), Pd[PPh_3_]_4_ (43 mg, 10 mol%) and (4-(9*H*-carbazol-9-yl)phenyl)boronic acid (322 mg, 3 equiv.). Then, the reaction mixture was stirred at reflux for 32 h. The progress of the reaction was monitored by TLC. After completion, the solvent was evaporated under reduced pressure and the residue was purified by flash chromatography using *n*-hexane-ethyl acetate (10:1→5:1) to give a pure product **7** as yellow powder. Yield 126 mg, 73%; m.p. 280–282 °C. ^1^H NMR (CDCl_3_): 9.22–9.18 (m, 1H, H-1), 9.01–8.97 (m, 2H, H-2′′), 8.34–8.29 (m, 1H, H-5), 8.20–8.16 (m, 2H, H-1′), 8.11–8.08 (m, 1H, H-4), 7.94–7.82 (m 4H, H-2, H-6, H-3′′), 7.74–7.70 (m, 1H, H-3), 7.60-7.57 (m, 2H, H-4′), 7.48–7.43 (m, 2H, H-3′ or H-2′), 7.36-7.31 (m, 2H, H-2′ or H-3′); ^13^C NMR (CDCl_3_): 161.1, 161.0, 158.8, 144.3, 140.7, 140.5, 137.1, 134.5, 130.9, 130.2, 129.8, 129.6, 129.2, 127.2, 126.9, 126.3, 125.1, 123.8, 120.6, 120.5, 113.8, 112.9, 110.1. Anal. Calcd. For C_31_H_18_N_4_O: C, 80.50; H, 3.92; N, 12.11%; found: C, 80.31; H, 4.07; N, 11.96%.

#### 3.4.2. 10-(Methylsulfonyl)naphtho[1′,2′:4,5]furo[3,2-*e*][1,2,4]triazine **8**

*m*CPBA (427 mg, ≤77%, 2.2 equiv.) was dissolved in dry DCM (5 mL), Na_2_SO_4_ (2.0 g) was added to the resulting solution and the mixture was stirred for 10 min. Na_2_SO_4_ was filtered and washed with DCM (3 × 5 mL). The obtained solution of *m*CPBA was added dropwise to a solution of **4aa** (133 mg, 0.5 mmol) in DCM (4 mL) at 0 °C. Then the reaction mixture was stirred at room temperature for 12 h. The progress of the reaction was monitored by TLC. After completion of the reaction, the mixture was quenched with an aqueous solution of NaHCO_3_, washed with water and dried over Na_2_SO_4,_ and the solvent was evaporated under reduced pressure. The residue was purified by flash chromatography using *n*-hexane-chloroform (2:1) as eluent to give pure **8** as **a** yellow powder. Yield 127 mg, 85%; m.p. 248–251 °C. ^1^H NMR (CDCl_3_): 9.07–9.04 (m, 1H, H-1), 8.43–8.39 (m, 1H, H-5), 8.11–8.08 (m, 1H, H-4), 7.93–7.88 (m, 2H, H-2, H-6), 7.76–7.72 (m, 1H, H-3), 7.65–7.58 (m, 1H, H-2), 3.66 (s, 2H, SO_2_CH_3_); ^13^C NMR (CDCl_3_): 163.8, 161.6, 160.4, 145.3, 139.5, 131.0, 130.7, 129.7, 128.7, 127.8, 125.5, 113.0, 112.6, 40.6. Anal. Calcd. For C_14_H_9_N_3_O_3_S: C, 56.18; H, 3.03; N, 14.04%; found: C, 56.05; H, 3.20; N, 13.96%.

#### 3.4.3. 10-(Carbazol-9-yl)naphtho[1′,2′:4,5]furo[3,2-*e*][1,2,4]triazine **9**

To a solution of carbazole (106 mg, 1.9 equiv.) in dry DMF (3 mL), we added NaH (60% suspension in mineral oil, 19 mg, 1.4 equiv.) and the mixture was stirred for 10 min. Then methylsulfonyl derivative **8** (100 mg, 0.33 mmol) was added to the resulting solution and the mixture was heated at 70 °C for 12 h. After completion of the reaction, the mixture was diluted with water (15 mL), and the forming precipitate was filtered and washed with water and ethanol and purified by flash chromatography using n-hexane:chloroform (2:1) as the eluent to give pure **9** as a yellow powder. Yield 71 mg, 55%; m.p. 250–253 °C. ^1^H NMR (HMPA d-18): 9.02–8.98 (m, 1H, H-1), 8.95-8.90 (d, *J* = 9.1 Hz, 1H, H-5), 8.85–8.78 (m, 2H, H-1′), 8.58–8.54 (m, 1H, H-4), 8.51–8.46 (m, 2H, H-4′), 8.42 (d, *J* = 9.1 Hz, 1H, H-6), 8.15–8.09 (m, 1H, H-3 and H-2), 7.89–7.82 (m, 1H, H-2 and H-3), 7.68–7.61 (m, 2H, H-3′ or H-2′), 7.50–7.43 (m, 2H, H-2′ or H-3′); ^13^C NMR (HMPAd-18): 160.2, 159.8, 157.9, 144.5, 139.4, 139.3, 131.5, 130.9, 130.6, 128.9, 127.3, 127.1, 126.0, 124.0, 122.9, 121.0, 115.1, 113.8, 113.2. Anal. Calcd. For C_25_H_14_N_4_O: C, 77.71; H, 3.65; N, 14.50%; found: C, 77.90; H, 2.81; N, 14.29%.

## 4. Conclusions

In summary, we have developed an unusual MnO_2_-induced oxidative cyclization in adducts of phenols and triazines. This method provides easy two-step access to benzofuro-fused triazine via the nucleophilic addition of the 2-naphthol to 1,2,4-triazine, followed by oxidative cyclization. The scope and limitations of this novel reaction have been investigated. Further application of the synthesized compound has been demonstrated by synthesizing carbazole-substituted benzofuro-fused triazines. The mechanistic study has revealed that the process proceeds through the formation of an O-coordinated Mn complex. We believe that the present methodology will open a new door to synthesizing important building blocks of α-sulfonylamino ketones.

## Figures and Tables

**Table 1:** Optimization of the reaction conditions ^1^.

Entry	Conditions	4aa (%) ^b^	5aa (%) ^b^
1	Using 3 equiv. of γ-MnO_2_	>99	trace
2	HNO_3_@γ-MnO_2_ instead of γ-MnO_2_	75	trace
3	Mn(OAc)_2_.4H_2_O instead of γ-MnO_2_	-	-
4	MnCl_2_ instead of γ-MnO_2_	-	-
5	Mn(acac)_2_ instead of γ-MnO_2_	-	-
6	Mn(OAc)_3_.2H_2_O instead of γ-MnO_2_	29	5
7	Ag_2_O instead of γ-MnO_2_	60	-
8	DTBP instead of γ-MnO_2_	35	15
9	DDQ instead of γ-MnO_2_	39	43
10	*p*-Chloranil instead of γ-MnO_2_	-	89
11	HFIP instead of CHCl_3_	89	trace
12	EtOH instead of CHCl_3_	66	trace
13	DCE instead of CHCl_3_	85	trace
14	Benzene instead of CHCl_3_	90	trace
15	Performed at 60 °C	94	4
16	Performed at 25 °C	91	3
17	Using 2 equiv. of γ-MnO_2_	85	3
18	Using 5 equiv. of γ-MnO_2_	90	6

^1^ Conditions: **3aa** (0.2 mmol), solvent (4 mL), 3 h. ^b 1^H NMR yield using 1,3,5-trimethoxybenzene as an internal standard.

## Data Availability

Data are contained within the article or Supplementary Materials.

## Supplementary Materials

The supporting information can be downloaded at: https://www.mdpi.com/article/10.3390/molecules27207105/s1. Synthesis of the starting 1,2,4-triazines, **3**, **4**, **4aa** derivatives; Preliminary mechanistic studies; DFT calculations; ^1^H and ^13^C NMR spectra for compounds **1**,**3–5**,**7–9**, **4aa′**.

## Sample Availability

Samples of the all compounds are available from the authors.
