# Supplementary material for: MnO2-Mediated Oxidative Cyclization of “Formal” Schiff’s Bases: Easy Access to Diverse Naphthofuro-Annulated Triazines
Source: Molecules. 2022 Oct 21;27(20):7105. doi: 10.3390/molecules27207105 (PMC9611995; doi:10.3390/molecules27207105)
Supplement: Supplementary file 1 [file molecules-27-07105-s001.zip › molecules-1977826-supplementary.pdf]

Supporting Information

MnO_2-Mediated Oxidative Cyclization of “Formal” Schiff’s Bases: Easy Access to Diverse Naphthofuro-Annulated Triazines

Ramil F. Fatykhov,[a] Igor A. Khalymbadzha,[a,b] Ainur D. Sharapov,[a] Anastasia Potapova,[a] Nataliya Mochulskaya,[a] Anton Tsmokalyuk,[a] Alexandra Ivoilova,[a] Polina Mozharovskaia,[a] Sougata Santra,[a]* and Oleg Chupakhin[a,b]

[a] Ural Federal University, 19 Mira Str., 620002 Ekaterinburg, Russia

[b] Postovsky Institute of Organic Synthesis, Ural Branch of the Russian Academy of Sciences, 22 S. Kovalevskaya Str., 620990 Ekaterinburg, Russia

*Email: sougatasantra85@gmail.com

Table of Contents

1. General information S2
2. Synthesis of the starting 1,2,4-triazines S2
3. Synthesis of the dihydrotriazines **3** S5
4. Synthesis of naphthofuro-fused triazines **4** S9
5. Further modifications of compound **4aa** S17
6. Preliminary mechanistic studies S20
7. DFT calculations S22
8. References S22
9. Copies of ^{1}H and ^{13}C NMR spectra for compounds **1** S26
10. Copies of ^{1}H and ^{13}C NMR spectra for compounds **3** S34
11. Copies of ^{1}H and ^{13}C NMR spectra for compounds **4** S49
12. Copies of ^{1}H and ^{13}C NMR spectra for compounds **5** S71
13. Copies of ^{1}H and ^{13}C NMR spectra for compound **7**,**8**,**9** S78
14. Copies of ^{1}H and ^{13}C NMR spectra for compound **4aa′** S81

1. General information

All commercially available chemicals were used without further purifications. ^{1}H NMR (400 MHz) and ^{13}C NMR (101 MHz) spectrum were recorded on a Bruker DRX-400 Avance spectrometer with DMSO-d_6 or $CDCl_3$ as solvent at ambient temperature. Chemical shifts are reported in ppm and coupling constants are given in Hz. Data for ^{1}H NMR are recorded as follows: chemical shift (ppm), multiplicity (s, singlet; d, doublet; t, triplet; q, quartet; quin, quintet; sex, sextet; m, multiplet; br s, broad signal), integration, coupling constant (Hz). High resolution mass spectra were recorded on Agilent UHPLC/MS Accurate-Mass Q-TOF 1290/6545. EPR spectra were obtained using Bruker Elexsys E500 CW-EPR spectrometer (modulation amplitude was set as 0.3 mT). Simulation of EPR spectra was performed using the package EasySpin 5.2 software.[1] Molecular geometry optimization and calculation energies of molecules was carried out in the gas phase using the B3LYP DFT functional[2] with a 6-311 + G (d, p) basis set[3] according to [4] in Gaussian09.[5] Electron density of molecular orbitals plots were obtained using the GaussView 6.0 software.[6] X-ray analysis for compound **5fa** was executed on an Xcalibur 3 diffractometer (MoKα radiation, graphite monochromator, 295(2) K, φ- and ω-scanning with a step of 1°. Column chromatography was carried out on silica gel (60Å, 0.035−0.070 mm).

2. Synthesis of the starting 1,2,4-triazines

2.1. Synthesis of S-substituted 3-thio-1,2,4-triazines 1a-f

S-substituted 3-thio-1,2,4-triazines **1a-f** were prepared from corresponding salt of S-substituted isothiosemicarbazide (2 mmol) and glyoxal solution according to the following procedure.[7]

A solution of 40% glyoxal (8 mmol, 1160 mg) and $NaHCO_3$ (5 mmol, 420 mg) in ice water (40 mL) was added to a solution of S-substituted isothiosemicarbazide hydrogen iodide (2 mmol) dissolved in ice water (40 mL). The reaction mixture was stirred for 15 min, during that time evolution of gas (CO_2) was observed. The reaction mixture was left in the fridge overnight and the aqueous solution was extracted with chloroform. The combined organic layer washed with 10% oxalic acid, dried over anhydrous Na_2SO_4, filtered, concentrated *in vacuo* to obtain oil or solid triazine compound.

Structure	NMR data
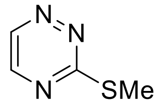	3-(Methylthio)-1,2,4-triazine[7] **1a**: light-orange solid. Yield 201 mg, 79%. **^{1}H NMR** ($CDCl_3$): 8.93 (d, 1H, *J*=2.3 Hz, H-6), 8.38 (d, 1H, *J*=2.3 Hz, H-5), 2.66 (s, 3H, CH_3).
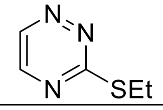	3-(Ethylthio)-1,2,4-triazine[7] **1b**: orange oil. Yield 206 mg, 73%. **^{1}H NMR** (DMSO-d_6): 9.05 (d, 1H, *J*=2.5 Hz, H-6), 8.56 (d, 1H, *J*=2.5 Hz, H-5), 3.20 (q, 2H, *J*=7.3 Hz, CH_2), 1.39 (t, 3H, *J*=7.3 Hz, CH_3).
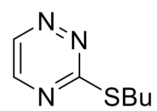	3-(Butylthio)-1,2,4-triazine[7] **1c**: orange oil. Yield 254 mg, 75%. **^{1}H NMR** ($CDCl_3$): 8.91 (d, 1H, *J*=2.4 Hz, H-6), 8.36 (d, 1H, *J*=2.4 Hz, H-5), 3.25 (t, 2H, *J*=7.4 Hz, $C\underline{H_2}CH_2CH_2CH_3$), 1.75 (quin, 2H, *J*=7.4 Hz, $CH_2C\underline{H_2}CH_2CH_3$), 1.49 (sex, 2H, *J*=7.4 Hz, $CH_2CH_2C\underline{H_2}CH_3$), 0.95 (t, 3H, *J*=7.4 Hz, CH_3).
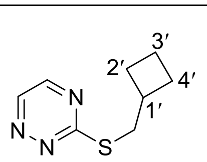	3-((Cyclobutylmethyl)thio)-1,2,4-triazine **1d**: dark red oil. Yield 257 mg, 71%. **^{1}H NMR** ($CDCl_3$): 8.89 (d, 1H, *J*=2.2 Hz, H-6), 8.33 (d, 1H, *J*=2.2 Hz, H-5), 3.40–3.27 (m, 2H, $SC\underline{H_2}$), 2.79–2.61 (m, 1H, H-1′), 2.22–2.05 (m, 2H, $C\underline{H_2}$-3′), 1.96–1.69 (m, 4H, $C\underline{H_2}$-2′ and $C\underline{H_2}$-4′); **^{13}C NMR** ($CDCl_3$): 174.8, 148.2, 145.3, 37.0, 34.5, 27.8, 18.0.
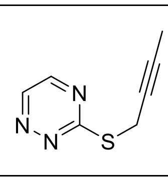	3-(But-2-yn-1-ylthio)-1,2,4-triazine **1e**: cream needles; Yield 222 mg, 67%. M.p. 52–54 °C. **^{1}H NMR** ($CDCl_3$): 8.95 (d, 1H, *J*=2.3 Hz, H-6), 8.40 (d, 1H, *J*=2.3 Hz, H-5), 3.99 (q, 2H, *J*=2.5 Hz, $SC\underline{H_2}$), 1.80 (t, 3H, *J*=2.5 Hz, $C\underline{H_3}$); **^{13}C NMR** ($CDCl_3$): 173.4, 148.4, 145.7, 79.7, 73.2, 20.2, 3.8.

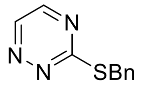	3-(Benzylthio)-1,2,4-triazine **1f**: pale yellow powder. Yield 365 mg, 90%. M.p. 55–57 °C.<br>**^{1}H NMR** (DMSO-d$_6$): 9.18 (d, 1H, *J*=2.4 Hz, H-6), 8.68 (d, 1H, *J*=2.4 Hz, H-5), 7.50–7.22 (m, 5H, Ph), 4.51 (s, 2H, SC<u>H</u>$_2$);<br>**^{13}C NMR** (DMSO-d$_6$): 172.4, 149.7, 146.7, 136.9, 129.0, 128.5, 127.3, 33.9.
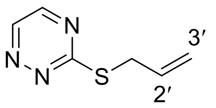	3-(Allylthio)-1,2,4-triazine **1g**: red oil. Yield 115 mg, 75%.<br>**^{1}H NMR** (CDCl$_3$): 8.92 (d, 1H, *J*=2.3 Hz, H-6), 8.36 (d, 1H, *J*=2.3 Hz, H-5), 5.97 (ddt, 1H, *J*=6.8 Hz, *J*(cis)=10.0 Hz, *J*(trans)=16.9 Hz, CH-2′), 5.35 (dd, 1H, ^2J=1.2 Hz, ^3J(trans)=16.9 Hz, CH-3′), 5.15 (dd, 1H, ^2J=1.2 Hz, ^3J(cis)=10.0 Hz, CH-3′), 3.89 (d, 2H, *J*=6.8 Hz, SC<u>H</u>$_2$);<br>**^{13}C NMR** (CDCl$_3$): 173.9, 148.3, 145.6, 132.4, 118.8, 33.5.

2.2.Synthesis of 3-(phenylthio)-1,2,4-triazine 1h

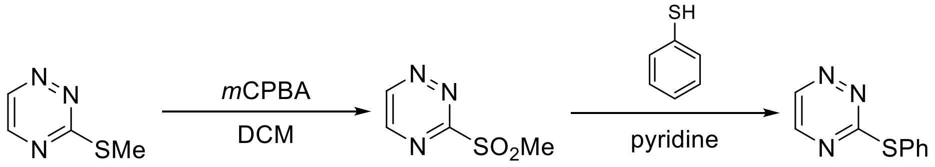

1a **1a′** **1h**

Compound **1h** was prepared *via* oxidation of **1a** with *m*CPBA using modified procedure[8] followed by the treatment of compound **1a′** with thiophenol.

*m*CPBA (11.6 g, 77%, 52 mmol) and anhydrous Na_2SO_4 (4.0 g) were successively added to DCM (60 ml), the mixture was stirred for 15 min and then filtered and the filter cake was washed with 10 ml of DCM to obtain a clear dichloromethane solution of *m*CPBA. A dichloromethane solution of 3-methylthio-1,2,4-triazine **1a** (3.0 g, 23.6 mmol) was added to this dichloromethane solution of *m*CPBA at -10°C with stirring. The reaction mixture was allowed to heat to ambient temperature and then stirred for additional 3 hours. Dichloromethane was evaporated under reduced pressure to obtain a dry mixture of 3-(methylsulfonyl)-1,2,4-triazine **1a′** and *m*-chlorobenzoic acid. The mixture was dissolved in pyridine (40 ml) and thiophenol (5.3 ml, 5.72 g, 52 mmol) was added hereto. After 24 hours the mixture was evaporated *in vacuo*, and the residue was treated with mixture of dichloromethane and aqueous $NaHCO_3$. The organic layer was evaporated yielding pure compound **1h**.

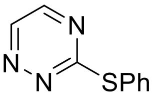	3-(Phenylthio)-1,2,4-triazine **1h**: yellow powder; yield 2251 mg, 60% (overall); m.p. 100–102 °C.<br>**^{1}H NMR** (DMSO-*d$_6$*): 9.18 (d, 1H, *J*=2.4 Hz, H-6), 8.62 (d, 1H, *J*=2.4 Hz, H-5), 7.68–7.63 (m, 2H, Ph), 7.54–7.48 (m, 3H, Ph);<br>**^{13}C NMR** (DMSO-*d$_6$*): 173.2, 150.1, 147.1, 135.3, 129.9, 129.7, 127.2.

2.3.Synthesis of 3-phenyl- and 3-(4-methoxyphenyl)-1,2,4-triazine 1i and 1j

Compounds **1i** and **1j** were prepared according to the published procedure.[9] The spectroscopic data for compound **1i** were in agreement with literature.[10]

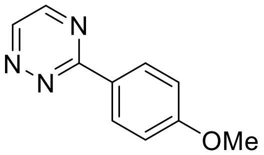	3-(4-Methoxyphenyl)-1,2,4-triazine **1j**: yellow powder; m.p. 99–101 °C.<br>**^{1}H NMR** (DMSO-*d$_6$*): 9.31 (d, 1H, *J*=2.4 Hz, H-6), 8.86 (d, 1H, *J*=2.4 Hz, H-5), 8.40 (d, 2H, *J*=9.0 Hz, Ph), 7.14 (d, 2H, *J*=9.0 Hz, Ph), 3.86 (s, 3H, MeO);<br>**^{13}C NMR** (DMSO-*d$_6$*): 162.7, 162.3, 149.8, 148.1, 129.5, 126.9, 114.5, 55.4.

2.4.Synthesis of 3-methyl- 1k and 3-benzyl-1,2,4-triazine 1l

Compounds **1k** and **1l** were prepared according to the known procedure.[11] The spectroscopic data of compounds **1k** were in agreement with the published data.[11]

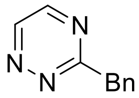	3-Benzyl-1,2,4-triazine **1l**: cream powder. M.p. 51–53 °C.<br>**^{1}H NMR** (DMSO-d_6): 9.31 (d, 1H, *J*=1.4 Hz, H-6), 8.78 (d, 1H, *J*=1.4 Hz, H-5), 7.32–7.22 (m, 5H, Ph), 34.40 (s, 2H, C<u>H</u>$_2$);<br>**^{13}C NMR** (DMSO-d_6): 168.6, 150.0, 148.5, 137.4, 129.1, 128.5, 126.6, 42.9.

2.5.Synthesis of 4-(1,2,4-triazin-3-yl)morpholine 1m

Compound **1m** was prepared according to the published procedure.[12]

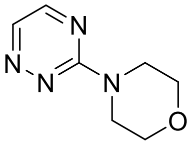	4-(1,2,4-Triazin-3-yl)morpholine **1m**: cream solid; m.p. 86–88 °C.<br>**^{1}H NMR** ($CDCl_3$): 8.53 (d, 1H, *J*=2.2 Hz, H-6), 8.13 (d, 1H, *J*=2.2 Hz, H-5), 3.93–3.85 (m, 4H, 2C<u>H</u>$_2$), 3.82–3.76 (m, 4H, 2C<u>H</u>$_2$);<br>**^{13}C NMR** ($CDCl_3$): 161.3, 148.9, 140.4, 66.8, 43.9.

3. Synthesis of dihydrotriazines 3

3.1.Optimization studies[a]

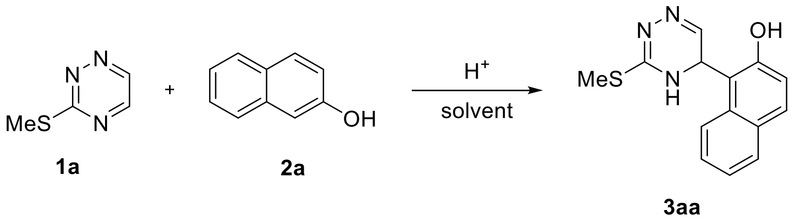

1a **2a** **3aa**

Entry	Conditions	Solvent	Yield,%	Ref.
1	$MeSO_3H$ (1 equiv.)	CH_2Cl_2	52	[13]
2	$MeSO_3H$ (3 equiv.)	CH_2Cl_2	78	[14]
**3**	**$MeSO_3H$ (3 equiv.)**	**AcOH**	**90**	**-**
4	$BF_3{\cdot}OEt_2$ (8 equiv.)	MeOH	81	[15]
5	CF_3CO_2H		58	[16]

[a]Reaction conditions: **1a** (1 mmol), **2a** (1mmol), solvent (4 ml). Isolated yield

3.2.General procedures

A: To a stirred solution of triazine **1a-j** (1 mmol, 1 equiv.) and 2-naphthol **2a,f,g** (1 mmol, 1 equiv.) in acetic acid (4 ml) was added a methanesulfonic acid (195 µl, 3 mmol, 3 equiv.). The resulting mixture was stirred at room temperature for 1-5 h. The progress of the reaction was monitored using TLC. After completion of the reaction, the reaction mixture was diluted with water (20 ml), neutralized with aq. $NaHCO_3$ solution and extracted with AcOEt (3×10 ml). The combined organic phase was dried over anhydrous Na_2SO_4, filtered and concentrated under reduced pressure. The residue was purified by silica gel chromatography or recrystallization from corresponding solvent to afford the product **3**.

B: To a stirred solution of triazine **1a** (1 mmol, 1 equiv.) and 2-naphthol **2b-e** (1 mmol, 1 equiv.) in methanol (4 mL) was added $BF_3{\cdot}OEt_2$ (985 µL, 8 mmol, 8 equiv.) and the resulting mixture was refluxed for 5 h. After cooling the methanol was evaporated under reduced pressure, the residue was dissolved in AcOEt (10 mL) and washed with aq. $NaHCO_3$ solution. The organic layer was dried over anhydrous Na_2SO_4, filtered, and evaporated under reduced pressure. Crude product was recrystallized from MeCN to obtain the product **3ab-3ae**.

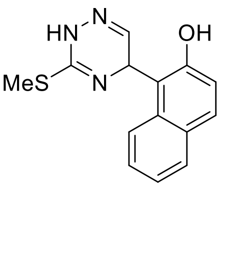	1-(3-(Methylthio)-2,5-dihydro-1,2,4-triazin-5-yl)naphthalen-2-ol **3aa**: according to the general procedure **A**, **3aa** pale yellow powder solid after recrystallization from MeCN. Yield 257 mg, 95%; m.p. 174–176 °C. **^{1}H NMR** (DMSO-d_6): 11.09 (s, 1H, NH), 10.70 (s, 1H, OH), 7.87–7.69 (m, 3H, H-5, H-8, H-4), 7.46–7.35 (m, 1H, H-7 or H-6), 7.33–7.25 (m, 1H, H-6 or H-7), 7.21–7.11 (m, 1H, H-3), 6.77 (s, 1H, H-6′), 5.20 (s, 1H, H-5′), 2.34 (s, 3H, SC$\underline{H}_3$); **^{13}C NMR** ($CDCl_3$): 154.8, 154.0, 141.7, 132.6, 129.4, 128.6, 128.5, 126.2, 123.1, 122.5, 118.8, 114.6, 53.1, 13.2. Anal. Calcd. For $C_{14}H_{13}N_3OS$: C, 61.97; H, 4.83; N, 15.49%; Found: C, 61.90; H, 4.89; N, 15.40%.
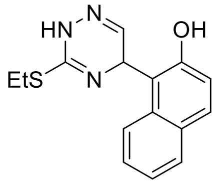	1-(3-(Ethylthio)-2,5-dihydro-1,2,4-triazin-5-yl)naphthalen-2-ol **3ba**: according to the general procedure **A**, **3ba** pale yellow powder after recrystallization from MeCN. Yield 251 mg, 88%; m.p. 174–176 °C. **^{1}H NMR** (DMSO-d_6): 11.03 (s, 1H, NH), 10.65 (s, 1H, OH), 7.86–7.71 (m, 3H, H-4, H-5, H-8), 7.43–7.35 (m, 1H, H-7 or H-6), 7.33–7.25 (m, 1H, H-6 or

	H-7), 7.20–7.13 (m, 1H, H-3), 6.76 (s, 1H, H-6′), 5.18 (s, 1H, H-5′), 3.00–2.80 (m, 2H, SC$\underline{H}_2$), 1.28–1.14 (m, 3H, C$\underline{H}_3$);<br>**^{13}C NMR** (DMSO-*d*$_6$): 153.9 (2C), 141.7, 132.6, 129.4, 128.6 (2C), 126.1, 123.2, 122.5, 118.7, 114.7, 53.0, 24.6, 14.9.<br>Anal. Calcd. For $C_{15}H_{15}N_3OS$: C, 63.13; H, 5.30; N, 14.73%; Found: C, 63.19; H, 5.38; N, 14.82%
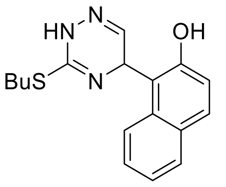	1-(3-(Butylthio)-2,5-dihydro-1,2,4-triazin-5-yl)naphthalen-2-ol **3ca**: according to the general procedure **A**, **3ca** pale yellow powder after recrystallization from MeCN. Yield 191 mg, 61%; m.p. 117–119 °C.<br>**^{1}H NMR** (DMSO-*d*$_6$): 11.05 (s, 1H, NH), 10.69 (s, 1H, OH), 7.86–7.70 (m, 3H, H-4, H-5, H-8), 7.43–7.36 (m, 1H, H-7 or H-6), 7.33–7.25 (m, 1H, H-6 or H-7), 7.17–7.15 (m, 1H, H-3), 6.75 (s, 1H, H-6′), 5.17 (s, 1H, H-5′), 3.00–2.85 (m, 2H, SC$\underline{H}_2$), 1.60–1.49 (m, 2H, CH$_2$), 1.38–1.24 (m, 2H, C$\underline{H}_2$), 0.86–0.77 (m, 3H, C$\underline{H}_3$);<br>**^{13}C NMR** (DMSO-*d*$_6$): 154.1, 154.0, 141.6, 132.6, 129.4, 128.6, 128.5, 126.1, 123.1, 122.5, 118.8, 114.6, 53.2, 31.1, 29.9, 21.2, 13.4.<br>Anal. Calcd. For $C_{17}H_{19}N_3OS$: C, 65.15; H, 6.11; N, 13.41%; Found: C, 65.20; H, 6.04; N, 13.43%
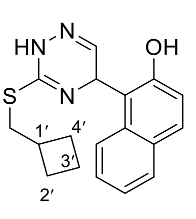	1-(3-((Cyclobutylmethyl)thio)-2,5-dihydro-1,2,4-triazin-5-yl)naphthalen-2-ol **3da**: according to the general procedure **A**, **3da** light yellow powder after recrystallization from MeCN. Yield 205 mg, 63%; m.p. 129–131 °C.<br>**^{1}H NMR** (DMSO-*d*$_6$): 11.03 (s, 1H, NH), 10.64 (s, 1H, OH), 7.85–7.70 (m, 3H, H-4, H-5, H-8), 7.43–7.35 (m, 1H, H-6 or H-7), 7.33–7.26 (m, 1H, H-6 or H-7), 7.19–7.13 (m, 1H, H-3), 6.74 (s, 1H, H-6′), 5.16 (s, 1H, H-5′), 3.09–2.91 (m, 2H, SC$\underline{H}_2$), 2.57–2.43 (m, 1H, C$\underline{H}$-1′), 2.04–1.90 (m, 2H, C$\underline{H}_2$-3′), 1.82–1.57 (m, 4H, C$\underline{H}_2$-2′, C$\underline{H}_2$-4′);<br>**^{13}C NMR** (DMSO-*d*$_6$): 154.0 (2C), 141.6, 132.6, 129.4, 128.6 (2C), 126.1, 123.2, 122.5, 118.7, 114.6, 53.1, 36.2, 34.4, 26.9 (2C), 17.3.<br>Anal. Calcd. For $C_{18}H_{19}N_3OS$: C, 66.43; H, 5.89; N, 12.91%; Found: C, 66.48; H, 5.96; N, 12.94%.
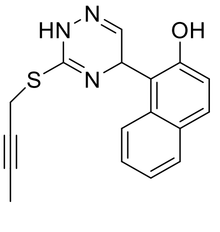	1-(3-(But-2-yn-1-ylthio)-2,5-dihydro-1,2,4-triazin-5-yl)naphthalen-2-ol **3ea**: according to the general procedure **A**, **3ea** light brown powder after recrystallization from MeCN. Yield 258 mg, 84%; m.p. 166–168 °C.<br>**^{1}H NMR** (DMSO-*d*$_6$): 11.11 (s, 1H, NH), 10.63–10.44 (br s, 1H, OH), 7.85–7.73 (m, 3H, H-4, H-5, H-8), 7.43–7.37 (m, 1H, H-6 or H-7), 7.32–7.27 (m, 1H, H-6 or H-7), 7.20–7.16 (m, 1H, H-3), 6.78 (s, 1H, H-6′), 5.21 (s, 1H, H-5′), 3.76 (dd, 1H, *J*=16.3 Hz, *J*=2.5 Hz, SC$\underline{H}_2$), 3.68 (dd, 1H, *J*=16.3 Hz, *J*=2.5 Hz, SC$\underline{H}_2$), 1.76 (t, 3H, *J*=2.5 Hz, C$\underline{H}_3$);<br>**^{13}C NMR** (DMSO-*d*$_6$): 153.9, 152.9, 141.9, 132.7, 129.5, 128.6 (2C), 126.2, 123.2, 122.5, 118.7, 114.8, 79.1, 74.9, 53.0, 19.3, 3.3.<br>Anal. Calcd. For $C_{17}H_{15}N_3OS$: C, 66.00; H, 4.89; N, 13.58%; Found: C, 66.08; H, 4.95; N, 13.54%.
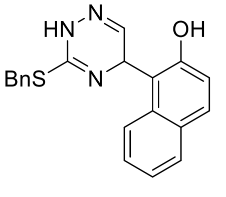	1-(3-(Benzylthio)-2,5-dihydro-1,2,4-triazin-5-yl)naphthalen-2-ol **3fa**: according to the general procedure **A**, **3fa** yellow powder after recrystallization from MeCN. Yield 309 mg, 89%; m.p. 165–166 °C.<br>**^{1}H NMR** (DMSO-*d*$_6$) 11.02 (s, 1H, NH), 10.42 (s, 1H, OH), 7.87–7.75 (m, 3H, H-4, H-5, H-8), 7.46–7.35 (m, 1H, H-6), 7.35–7.28 (m, 3H, Ph), 7.26–7.18 (m, 4H, H-3, H-7, Ph), 6.80 (s, 1H, H-6′), 5.23 (s, 1H, H-5′), 4.20 (d, *J*=13.4 Hz, 2H, C$\underline{H}_2$), 4.16 (d, *J*=13.4 Hz, 2H, C$\underline{H}_2$);<br>**^{13}C NMR** (DMSO-*d*$_6$): 153.7, 153.3, 142.1, 137.9, 132.8, 129.5, 128.9, 128.7, 128.6, 128.4, 127.1, 126.1, 123.5, 122.5, 118.5, 115.5, 52.6, 34.0.<br>Anal. Calcd. For $C_{20}H_{17}N_3OS$: C, 69.14; H, 4.93; N, 12.09%; Found: C, 69.22; H, 4.99; N, 12.01%.

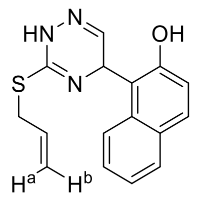	1-(3-(Allylthio)-2,5-dihydro-1,2,4-triazin-5-yl)naphthalen-2-ol **3ga**: according to the general procedure **A**, **3ga** orange powder after recrystallization from MeCN. Yield 199 mg, 67%; m.p. 140–142 °C.<br>**^{1}H NMR** (DMSO-d_6) 11.08 (s, 1H, NH), 10.56, (s, 1H, OH), 7.85–7.72 (m, 3H, H-4, H-5, H-8), 7.43–7.36 (m, 1H, H-6 or H-7), 7.33–7,25 (m, 1H, H-6 or H-7), 7.21–7.14 (m, 1H, H-3), 6.76 (s, 1H, H-6′), 5.89–5.80 (m, 1H, CH(All)), 5.25–5.14 (m, 2H, H-5′, H(All)), 5.10–5.03 (m, 1H, CH(All)), 3.68–3.52 (m, 2H, SCH_2);<br>**^{13}C NMR** (DMSO-d_6): 153.9, 153.3, 141.8, 133.7, 132.7, 129.5, 128.6 (2C), 126.1, 123.3, 122.5, 118.7, 117.9, 114.9, 53.0, 32.9.<br>Anal. Calcd. For $C_{16}H_{13}N_3OS$: C, 65.07; H, 4.44; N, 14.23%; Found: C, 65.17; H, 4.53; N, 14.30%.
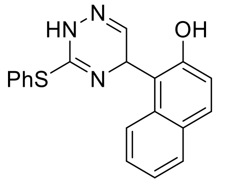	1-(3-(Phenylthio)-2,5-dihydro-1,2,4-triazin-5-yl)naphthalen-2-ol **3ha**: according to the general procedure **A**, **3ha** pale yellow needles after purification by silica gel chromatography using *n*-hexane-ethyl acetate (10:1→5:1). Yield 243 mg, 73%; m.p. 148–150 °C.<br>**^{1}H NMR** (DMSO-d_6) 11.41 (s, 1H, NH), 10.68 (s, 1H, OH), 7.82–7.67 (m, 3H, H-4, H-5, H-8), 7.61–7.52 (m, 2H, Ph), 7.48–7.35 (m 4H, H-6, Ph), 7.33–7.24 (m, 1H, H-7), 7.09–7.00 (m, 1H, H-3), 6.74 (s, 1H, H-6′), 5.13 (s, 1H, H-5′);<br>**^{13}C NMR** (DMSO-d_6): 154.3, 153.6, 141.4, 134.0, 132.2, 129.5, 129.4, 129.2, 128.6, 128.6, 128.4, 126.3, 122.7, 122.5, 119.0, 113.3, 54.2.<br>Anal. Calcd. For $C_{19}H_{15}N_3OS$: C, 68.45; H, 4.53; N, 12.60%; Found: C, 68.38; H, 4.46; N, 12.50%.
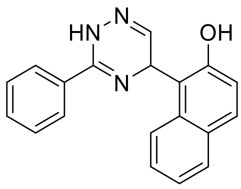	1-(3-Phenyl-2,5-dihydro-1,2,4-triazin-5-yl)naphthalen-2-ol **3ia**: according to the general procedure **A**, **3ia** pale yellow powder after purification by silica gel chromatography using *n*-hexane-ethyl acetate (10:1→5:1). Yield 193 mg, 64%; m.p. 170–172 °C.<br>**^{1}H NMR** (DMSO-d_6) 11.63 (s, 1H, NH), 11.22 (s, 1H, OH), 8.00–7.92 (m, 2H, Ph), 7.87–7.74 (m, 3H, H-4, H-5, H-8), 7.59–7.47 (m, 3H, Ph), 7.45–7.36 (m, 1H, H-6 or H-7), 7.33–7.27 (m, 1H, H-6 or H-7), 7.21–7.13 (m, 1H, H-3), 6.80 (s, 1H, H-6′), 5.26 (s, 1H, H-5′);<br>**^{13}C NMR** (DMSO-d_6): 154.5, 153.2, 140.2, 132.5, 131.7, 131.2, 129.4, 128.7, 128.7, 128.5, 127.2, 126.3, 122.9, 122.5, 119.1, 114.1, 52.6.<br>Anal. Calcd. For $C_{19}H_{15}N_3O$: C, 75.73; H, 5.02; N, 13.94%; Found: C, 75.77; H, 5.10; N, 13.98%.
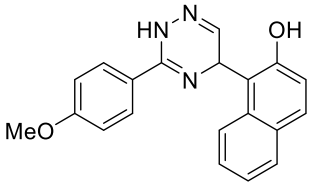	1-(3-(4-Methoxyphenyl)-2,5-dihydro-1,2,4-triazin-5-yl)naphthalen-2-ol **3ja**: according to the general procedure **A**, **3ja** pale yellow powder after purification by silica gel chromatography using *n*-hexane-ethyl acetate (10:1→5:1). Yield 247 mg, 68%; m.p. 173–175 °C.<br>**^{1}H NMR** (DMSO-d_6) 11.66–11.53 (m, 2H, NH, OH), 7.94 (d, 2H, *J*=8.8 Hz, Ph), 7.88–7.70 (m, 3H, H-4, H-5, H-8), 7.46–7.37 m, 1H, H-6 or H-7), 7.34–7.26 (m, 1H, H-6 or H-7), 7.18–7.10 (m, 1H, H-3), 7.06 (d, 2H, *J*=8.8 Hz, Ph), 6.78 (s, 1H, H-6′), 5.21 (s, 1H, H-5′), 3.83 (s, 3H, OCH_3);<br>**^{13}C NMR** (DMSO-d_6): 161.7, 154.8, 152.9, 140.2, 132.3, 129.4, 128.9, 128.6, 128.4, 126.4, 123.7, 122.6, 122.5, 119.3, 114.1, 113.4, 55.4, 53.0.<br>Anal. Calcd. For $C_{20}H_{17}N_3O_2$: C, 72.49; H, 5.17; N, 12.68%; Found: C, 72.40; H, 5.25; N, 12.60%.
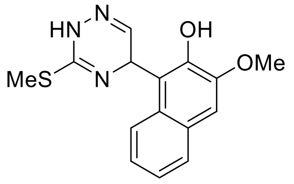	3-Methoxy-1-(3-(methylthio)-2,5-dihydro-1,2,4-triazin-5-yl)naphthalen-2-ol **3ab**: according to the general procedure **B**, **3ab** pale yellow solid. Yield 214 mg, 71%; m.p. 193–195 °C.<br>**^{1}H NMR** (DMSO-d_6): 11.07 (s, 1H, NH), 10.21 (s, 1H, OH), 7.79–7.74 (m, 1H, H-5 or H-8), 7.68–7.64 (m, 1H, H-5 or H-8), 7.33 (s, 1H, H-4), 7.30–7.24 (m, 2H, H-6, H-7), 6.76 (d, 1H, *J*=1.2 Hz, H-6′), 5.20 (d, 1H, *J*=1.2 Hz, H-5′), 3.94 (s, 3H, OCH_3), 2.32 (s, 1H, SCH_3);<br>**^{13}C NMR** (DMSO-d_6): 154.7, 148.4, 146.0, 141.6, 128.7, 127.5, 127.3, 123.8, 123.1, 123.0, 115.7, 106.4, 55.7, 53.2, 13.1.

	Anal. Calcd. For $C_{15}H_{15}N_3O_2S$: C, 59.78; H, 5.02; N, 13.94%; Found: C, 59.89; H, 5.14; N, 13.90%.
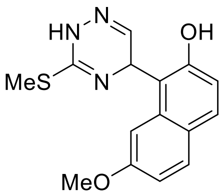	7-Methoxy-1-(3-(methylthio)-2,5-dihydro-1,2,4-triazin-5-yl)naphthalen-2-ol **3ac**: according to the general procedure **B**, **3ac** pale yellow powder. Yield 190 mg, 63%; m.p.149–151 °C.<br>**^{1}H NMR** (DMSO-d_6): 11.05 (s, 1H, NH), 10.35 (s, 1H, OH), 7.75–7.67 (m, 2H, H-4, H-5), 7.13–7.08 (m, 1H, H-8), 7.02–6.93 (m, 2H, H-3, H-6), 6.76 (s, 1H, H-6′), 5.25 (s, 1H, H-5′), 3.75 (s, 3H, $OC\underline{H}_3$), 2.34 (s, 3H, $SC\underline{H}_3$);<br>**^{13}C NMR** (DMSO-d_6): 157.4, 154.5 (2C), 141.5, 134.0, 130.1, 129.1, 123.8, 116.1, 114.1, 114.0, 103.0, 54.8, 53.0, 13.0.<br>Anal. Calcd. For $C_{15}H_{15}N_3O_2S$: C, 59.78; H, 5.02; N, 13.94%; Found: C, 59.70; H, 5.09; N, 13.84%.
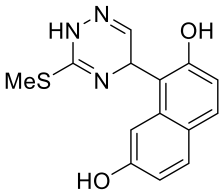	1-(3-(Methylthio)-2,5-dihydro-1,2,4-triazin-5-yl)naphthalene-2,7-diol **3ad**: according to the general procedure **B**, **3ad** pale yellow solid. Yield 184 mg, 64%; m.p. 172–174 °C.<br>**^{1}H NMR** (DMSO-d_6): 11.07 (s, 1H, NH), 10.70–10.46 (br s, 1H, OH), 9.68–9.47 (br s, 1H, OH), 7.71–7.56 (m, 2H, H-4, H-5), 6.97–6.93 (m, 1H, H-8), 6.92–6.87 (m, 1H, H-3), 6.86–6.81 (m, 1H, H-6), 6.75 (s, 1H, H-6′), 5.00 (s, 1H, H-5′), 2.35 (s, 3H, $SC\underline{H}_3$);<br>**^{13}C NMR** (DMSO-d_6): 155.6, 154.9, 154.4, 141.9, 134.2, 130.1, 129.2, 123.1, 115.3, 114.8, 112.6, 105.3, 53.4, 13.2.<br>Anal. Calcd. For $C_{14}H_{13}N_3O_2S$: C, 58.52; H, 4.56; N, 14.62%; Found: C, 58.62; H, 4.65; N, 14.60%.
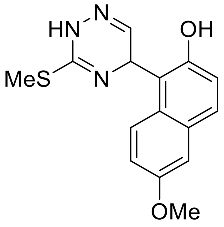	6-Methoxy-1-(3-(methylthio)-2,5-dihydro-1,2,4-triazin-5-yl)naphthalen-2-ol **3ae**: according to the general procedure **B**, **3ae** pale yellow solid. Yield 247 mg, 82%; m.p. 186–188 °C.<br>**^{1}H NMR** (DMSO-d_6): 11.05 (s, 1H, NH), 10.35 (s, 1H, OH), 7.73–7.60 (m, 2H, H-4, H-8), 7.29–7.22 (m, 1H, H-5), 7.16–7.04 (m, 2H, H-3, H-7), 6.74 (s, 1H, H-6′), 5.16 (s, 1H, H-5′), 3.83 (s, 3H, $OC\underline{H}_3$), 2.33 (s, 3H, $SC\underline{H}_3$)<br>**^{13}C NMR** (DMSO-d_6): 155.6, 154.9, 154.4, 141.9, 134.2, 130.1, 129.2, 123.1, 115.3, 114.8, 112.6, 105.3, 53.4, 13.2.<br>Anal. Calcd. For $C_{15}H_{15}N_3O_2S$: C, 59.78; H, 5.02; N, 13.94%; Found: C, 59.70; H, 5.09; N, 13.99%.
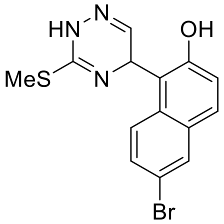	6-Bromo-1-(3-(methylthio)-2,5-dihydro-1,2,4-triazin-5-yl)naphthalen-2-ol **3af**: according to the general procedure **A**, **3ca** pale yellow powder after recrystallization from MeCN. Yield 287 mg, 82%; m.p. 186–188 °C.<br>**^{1}H NMR** (DMSO-d_6): 11.10 (s, 1H, NH), 10.76, (s, 1H, OH), 8.09 (d, *J*=1.6 Hz, 1H, H-5), 7.78 (d, *J*=8.9 Hz, 1H, H-4), 7.70 (d, *J*=9.1 Hz, 1H, H-8), 7.52 (dd, *J*=1.6 Hz, *J*=9.1 Hz, H-7), 7.21 (d, *J*=8.9 Hz, 1H, H-3), 6.77 (s, 1H, H-6′), 5.16 (s, 1H, H-5′), 2.32 (s, 3H, $SC\underline{H}_3$);<br>**^{13}C NMR** (DMSO-d_6): 154.8, 154.3, 141.4, 131.3, 130.2, 129.9, 128.9, 128.7, 125.6, 119.8, 115.4, 115.4, 52.8, 13.1.<br>Anal. Calcd. For $C_{14}H_{12}BrN_3OS$: C, 48.01; H, 3.45; N, 12.00%; Found: C, 48.11; H, 3.40; N, 12.09%.
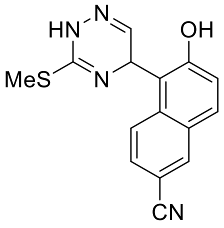	6-Hydroxy-5-(3-(methylthio)-2,5-dihydro-1,2,4-triazin-5-yl)-2-naphthonitrile **3ag**: according to the general procedure **A**, **3ag** cream powder after recrystallization from MeCN. Yield 270 mg, 91%; m.p. 184–186 °C.<br>**^{1}H NMR** (DMSO-d_6): 11.19 (s, 1H, NH), 11.10, (s, 1H, OH), 8.45 (d, *J*=1.7 Hz, 1H, H-5), 7.94 (d, *J*=9.0 Hz, 1H, H-4), 7.89 (d, *J*=8.9 Hz, 1H, H-8), 7.66 (dd, *J*=1.7 Hz, *J*=8.9 Hz, H-7), 7.32 (d, *J*=9.0 Hz, 1H, H-3), 6.79 (s, 1H, H-6′), 5.19 (s, 1H, H-5′), 2.31 (s, 3H, $SC\underline{H}_3$).<br>**^{13}C NMR** (DMSO-d_6): 156.7, 154.8, 141.2, 134.8, 134.6, 130.4, 127.5, 126.5, 124.6, 120.3, 119.5, 115.9, 104.6, 52.6, 13.1.<br>Anal. Calcd. For $C_{15}H_{12}N_4OS$: C, 60.80; H, 4.08; N, 18.91%; Found: C, 60.72; H, 4.01; N, 18.85%.

4. Synthesis of naphthofuro-fused triazines 4

4.1. Optimization studies

Screening of the oxidant[a]

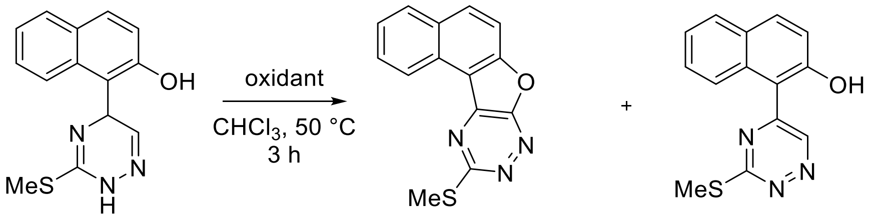

3aa **4aa** **5aa**

Entry	Oxidant	Yield of **4aa** (%)[b]	Yield of **5aa** (%)[b]
1[c]	γ-MnO_2 (3 equiv)	>99	trace
2	$Mn(OAc)_3·2H_2O$ (3 equiv)	29	5
3	$Mn(OAc)_2·4H_2O$ (3 equiv)	-	-
4	$MnCl_2$ (3 equiv)	-	-
5	$Mn(acac)_2$ (3 equiv)	-	-
6	Ag_2O (3 equiv)	60	-
7	DDQ (2 equiv)	39	43
8	*p*-Chloranil (1.2 equiv.)	-	89
9	DTBP (2 equiv)	35	15

[a]Conditions: **3aa** (0.2 mmol), oxidant in 3 ml $CHCl_3$; NMR yield by using 1,3,5-trimethoxybenzene as an internal standard; [c]MnO_2 was prepared according to the published procedure.[17]

Screening of various MnO_2[a]

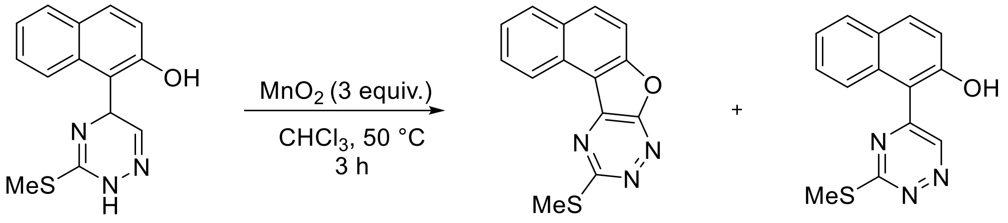

3aa **4aa** **5aa**

Entry	MnO_2	Yield of **4aa** (%)[b]	Yield of **5aa** (%)[b]
1[c]	γ-MnO_2	>99	trace
2[d]	MnO_2	Trace	trace
3[e]	MnO_2	25	trace
4[f]	HNO_3@MnO_2	75	trace
5[g]	*nano*-MnO_2	35	10

[a]Conditions: **3aa** (0.2 mmol), oxidant in 3 ml $CHCl_3$; [b]NMR yield by using 1,3,5-trimethoxybenzene as an internal standard;

[c]γ-MnO_2 was prepared according to the published procedure;[17]

[d]MnO_2 was prepared from the reaction of potassium permanganate and dihydrogen peroxide according to published procedure;[18]

[e]MnO_2 was prepared from the reaction of potassium permanganate with methanol according to the published procedure;[19]

[f]MnO_2 impregnated with nitric acid was prepared according to the published procedure;[19]

[g]*nano*-MnO_2 was prepared according to the published procedure.[20]

Screening of the solvent[a]

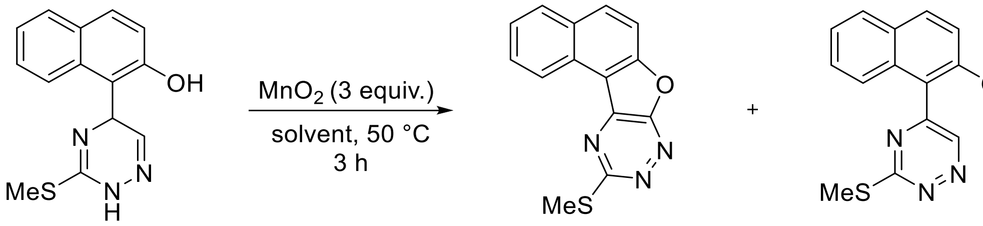

3aa **4aa** **5aa**

Entry	Solvent	Yield of **4aa** (%)[b]	Yield of **5aa** (%)[b]
1	$CHCl_3$	>99	trace
2	CH_2Cl_2 (R.T.)	70	5
3	$(ClCH_2)_2$	88	trace
4	HFIP	89	trace
5	AcOH	67	trace
7	*i*PrOH	80	4
8	Benzene	69	trace

[a]Conditions: **3aa** (0.2 mmol), oxidant in 3 ml $CHCl_3$; [b]NMR yield by using 1,3,5-trimethoxybenzene as an internal standard.

Screening of the other parameters[a]

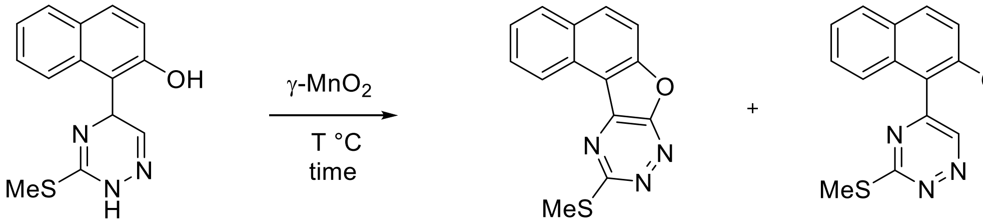

3aa **4aa** **5aa**

Entry	MnO_2 (equiv.)	T, °C	Time, h	Yield of **4aa** (%)[b]	Yield of **5aa** (%)[b]
1	3.0	50	3	>99	trace
2	3.0	60	3	94	4
3	5.0	50	3	90	6
4	3.0	25	18	91	3
5	4.0	25	12	94	4
7	2.0	50	24	85	3
8	1.0	50	24	35	2

[a]Conditions: **3aa** (0.2 mmol), γ-MnO_2 in 3 ml $CHCl_3$; [b]NMR yield by using 1,3,5-trimethoxybenzene as an internal standard.

4.2.General procedure

To a stirred solution of **3** (0.2 mmol, 1 equiv.) in $CHCl_3$ (3 ml) MnO_2 (52 mg, 0.6 mmol, 3 equiv.) was added in one portion. The resulting mixture was stirred at 50 °C for 3 h. The completion of the reaction was monitored by TLC. The reaction mixture was then cooled to room temperature and MnO_2 was filtered and the filter cake was washed with $CHCl_3$ (3×10 ml). The combined organic phase is concentrated under reduced pressure. The residue was purified by chromatography on silica gel or recrystallization to afford the pure product **4**.

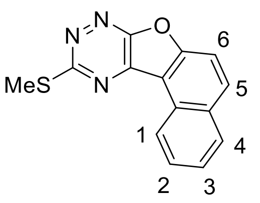	10-(Methylthio)naphtho[1',2':4,5]furo[3,2-*e*][1,2,4]triazine **4aa**: pale yellow needles after recrystallization from MeCN. Yield 51 mg, 95%; m.p. 185–187 °C.<br>**^{1}H NMR** ($CDCl_3$): 8.78–8.66 (m, 1H, H-1), 8.20–8.06 (m, 1H, H-5), 7.97–7.86 (m, 1H, H-4), 7.76–7.52 (m, 3H, H-2, H-3, H-6), 2.79 (s, 1H, SC$\underline{H}_3$);<br>**^{13}C NMR** ($CDCl_3$): 169.8, 160.0, 158.6, 143.7, 137.0, 130.5, 129.6, 129.2, 128.7, 126.7, 124.7, 112.7, 112.6, 14.8.<br>Anal. Calcd. For $C_{14}H_9N_3OS$: C, 62.91; H, 3.39; N, 15.72%; Found: C, 62.96; H, 3.47; N, 15.79%.
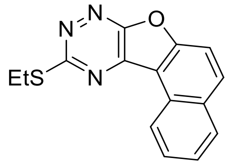	10-(Ethylthio)naphtho[1',2':4,5]furo[3,2-*e*][1,2,4]triazine **4ba**: pale yellow needles after recrystallization from MeCN. Yield 47 mg, 84%; m.p. 141–143 °C.<br>**^{1}H NMR** ($CDCl_3$): 8.97–8.88 (m, 1H, H-1), 8.28–8.21 (m, 1H, H-5), 8.06–7.99 (m, 1H, H-4), 7.87–7.75 (m, 2H, H-2, H-6), 7.70–7.62 (m, 1H, H-3), 3.44 (q, 2H, *J*=7.3 Hz, SC$\underline{H}_2$), 1.56 (t, 3H, *J*=7.3 Hz, C$\underline{H}_3$);<br>**^{13}C NMR** ($CDCl_3$): 169.6, 160.1, 158.7, 143.9, 137.0, 130.6, 129.6, 129.3, 128.9, 126.8, 124.8, 112.9, 112.7, 26.1, 14.4.<br>Anal. Calcd. For $C_{15}H_{11}N_3OS$: C, 64.04; H, 3.94; N, 14.94%; Found: C, 64.12; H, 3.99; N, 14.85%.
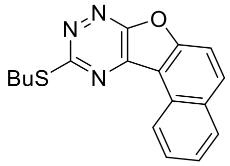	10-(Butylthio)naphtho[1',2':4,5]furo[3,2-*e*][1,2,4]triazine **4ca**: pale yellow needles after recrystallization from MeCN. Yield 53 mg, 85%; m.p. 121–123 °C.<br>**^{1}H NMR** ($CDCl_3$): 8.83–8.75 (m, 1H, H-1), 8.20–8.12 (m, 1H, H-5), 7.99–7.92 (m, 1H, H-4), 7.79–7.55 (m, 3H, H-2, H-3, H-6), 3.45–3.33 (m, 2H, SC$\underline{H}_2$), 1.96–1.83 (m, 2H, $SCH_2C\underline{H}_2$), 1.68–1.53 (m, 2H, $SCH_2CH_2C\underline{H}_2$), 1.08–0.97 (m, 3H, C$\underline{H}_3$);<br>**^{13}C NMR** ($CDCl_3$): 169.8, 160.1, 158.7, 143.9, 137.1, 130.6, 129.6, 129.4, 128.9, 126.8, 124.9, 113.0, 112.8, 31.4, 31.3, 22.3, 13.9.<br>Anal. Calcd. For $C_{17}H_{15}N_3OS$: C, 66.00; H, 4.89; N, 13.58%; Found: C, 66.09; H, 4.82; N, 13.50%.
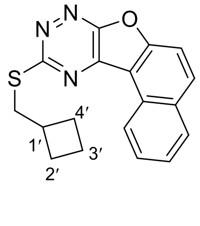	10-((Cyclobutylmethyl)thio)naphtho[1',2':4,5]furo[3,2-*e*][1,2,4]triazine **4da**: yellow needles after recrystallization from MeCN. Yield 57 mg, 89%; m.p. 154–156 °C.<br>**^{1}H NMR** ($CDCl_3$): 8.81–8.76 (m, 1H, H-1), 8.17 (d, 1H, *J*=9.0 Hz, H-5), 7.98–7.93 (m, 1H, H-4), 7.78–7.72 (m, 1H, H-2), 7.70 (d, 1H, *J*=9.0 Hz, H-6), 7.63–7.57 (m, 1H, H-3), 3.53–3.44 (m, 2H, SC$\underline{H}_2$), 2.90–2.80 (m, 1H, C$\underline{H}$-1′), 2.29–2.18 (m, 2H, C$\underline{H}_2$-3′), 1.98–1.83 (m, 4H, C$\underline{H}_2$-2′, C$\underline{H}_2$-4′);<br>**^{13}C NMR** ($CDCl_3$): 169.7, 160.0, 158.7, 143.8, 137.0, 130.6, 129.6, 129.3, 128.8, 126.7, 124.8, 112.8, 112.7, 37.9, 34.7, 28.0, 18.2.<br>Anal. Calcd. For $C_{18}H_{15}N_3OS$: C, 67.27; H, 4.70; N, 13.07%; Found: C, 67.35; H, 4.78; N, 13.12%.
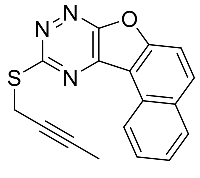	10-(But-2-yn-1-ylthio)naphtho[1',2':4,5]furo[3,2-*e*][1,2,4]triazine **4ea**: brown powder after purification by chromatography on silica gel using *n*-hexane-ethyl acetate (10:1). Yield 45 mg, 70%; m.p. 163–165 °C.<br>**^{1}H NMR** ($CDCl_3$): 8.88–8.83 (m, 1H, H-1), 8.22 (d, 1H, *J*=9.0 Hz, H-5), 8.02–7.97 (m, 1H, H-4), 7.82–7.77 (m, 1H, H-2), 7.74 (d, 1H, *J*=9.0 Hz, H-6), 7.67–7.61 (m, 1H, H-3), 4.15 (q, 2H, *J*=2.5 Hz, SC$\underline{H}_2$), 1.84 (t, 3H, *J*=2.5 Hz, C$\underline{H}_3$);<br>**^{13}C NMR** ($CDCl_3$): 168.4, 160.1, 158.8, 143.9, 137.3, 130.6, 129.8, 129.8, 128.9, 126.9, 124.9, 112.9, 112.7, 79.5, 73.7, 21.0, 3.9.<br>Anal. Calcd. For $C_{17}H_{11}N_3OS$: C, 66.87; H, 3.63; N, 13.76%; Found: C, 66.80; H, 3.60; N, 13.86%.

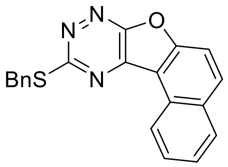

10-(Benzylthio)naphtho[1',2':4,5]furo[3,2-*e*][1,2,4]triazine **4fa**: yellow needles after recrystallization from MeCN. Yield 63 mg, 90%; m.p. 161–163 °C.

^{1}H NMR ($CDCl_3$): 8.82–8.73 (m, 1H, H-1), 8.17 (d, 1H, *J*=9.1 Hz, H-5), 7.97–7.93 (m, 1H, H-4), 7.78–7.72 (m, 1H, H-3), 7.70 (d, 1H, *J*=9.1 Hz, H-6), 7.26–7.56 (m, 3H, H-2, Ph), 7.38–7.32 (m, 2H, Ph), 7.30–7.25 (m, 1H, Ph), 4.66 (m, 1H, $SC\underline{H}_2$);

^{13}C NMR ($CDCl_3$): 169.0, 160.2, 158.8, 143.9, 137.2, 137.1, 130.6, 129.7, 129.4, 129.4, 128.9, 128.7, 127.6, 126.8, 124.9, 112.9, 112.7, 36.1.

Anal. Calcd. For $C_{20}H_{13}N_3OS$: C, 69.95; H, 3.82; N, 12.24%; Found: C, 69.85; H, 3.76; N, 12.20%.

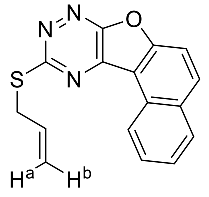

10-(Allylthio)naphtho[1',2':4,5]furo[3,2-*e*][1,2,4]triazine **4ga**: pale yellow powder after recrystallization from MeCN. Yield 46 mg, 78%; m.p. 126–128 °C.

^{1}H NMR ($CDCl_3$): 8.83–8.74 (m, 1H, H-1), 8.17 (d, 1H, *J*=9.1 Hz, H-5), 7.99–7.93 (m, 1H, H-4), 7.78–7.72 (m, 1H, H-3), 7.70 (d, 1H, *J*=9.1 Hz, H-6), 7.64–7.57 (m, 1H, H-2), 6.13 (ddt, 1H, ^{3}J=6.9 Hz, ^{3}J(cis)=10.0 Hz, ^{3}J(trans)=17.0 Hz, CH-2′), 5.47 (dd, 1H, ^{3}J(trans)=16.9 Hz, *J*=1.2 Hz, CH-3a′), 5.22 (d, 1H, ^{3}J(cis)=10.0 Hz, CH-3b′), 4.06 (d, 2H, ^{3}J=6.9 Hz, $SC\underline{H}_2$);

^{13}C NMR ($CDCl_3$): 168.9, 160.1, 158.7, 143.8, 137.1, 133.1, 130.6, 129.7, 129.3, 128.8, 126.8, 124.8, 118.7, 112.9, 112.7, 34.5.

Anal. Calcd. For $C_{16}H_9N_3OS$: C, 65.97; H, 3.11; N, 14.42%; Found: C, 65.90; H, 3.18; N, 14.50%.

A mixture of **4ha** and **5ha** was separated by silica gel chromatography using *n*-hexane-ethyl acetate (17:1) to isolate **4ha** and *n*-hexane-ethyl acetate (10:1) to give **5ha**

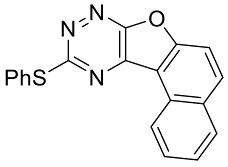

10-(Phenylthio)naphtho[1',2':4,5]furo[3,2-*e*][1,2,4]triazine **4ha**: yellow powder. Yield 46 mg, 70%; m.p. 196–198 °C.

^{1}H NMR ($CDCl_3$): 8.58–8.53 (m, 1H, H-1), 8.22–8.17 (m, 1H, H-5), 8.00–7.95 (m, 1H, H-4), 7.83–7.77 (m, 2H, Ph), 7.75–7.67 (m, 2H, H-3, H-6), 7.64–7.58 (m, 1H, H-2), 7.56–7.50 (m, 3H, Ph);

^{13}C NMR ($CDCl_3$): 170.0, 160.3, 158.8, 144.0, 137.2, 135.8, 130.6, 129.7, 129.6, 129.5, 129.3, 129.2, 128.8, 126.8, 124.8, 113.0, 112.7.

Anal. Calcd. For $C_{19}H_{11}N_3OS$: C, 69.29; H, 3.37; N, 12.76%; Found: C, 69.20; H, 3.45; N, 12.70%.

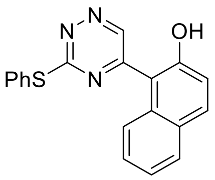

1-(3-(Phenylthio)-1,2,4-triazin-5-yl)naphthalen-2-ol **5ha**: yellow powder Yield 9 mg, 14%; m.p. 149–151 °C.

^{1}H NMR ($CDCl_3$): 11.01 (s, 1H, OH), 9.51 (s, 1H, H-6′), 8.09–8.03 (m, 1H, H-8), 7.84 (d, 1H, *J*=9.0 Hz, H-4), 7.82–7.76 (m, 1H, H-5), 7.75–7.67 (m, 2H, Ph), 7.59–7.51 (m, 4H, Ph, H-7), 7.44–7.37 (m, 1H, H-6), 7.07 (d, 1H, *J*=9.0 Hz, H-3);

^{13}C NMR ($CDCl_3$): 172.3, 160.8, 155.7, 146.2, 136.2, 136.0, 130.8 (2C), 130.3, 129.5, 129.2, 128.7, 126.9, 124.7, 123.2, 119.7, 108.9.

Anal. Calcd. For $C_{19}H_{13}N_3OS$: C, 68.86; H, 3.95; N, 12.68%; Found: C, 68.77; H, 3.90; N, 12.60%.

A mixture of **4ia** and **5ia** was separated by chromatography using *n*-hexane-ethyl acetate (15:1) to give **4ia** and *n*-hexane-ethyl acetate (8:1) to give **5ia**.

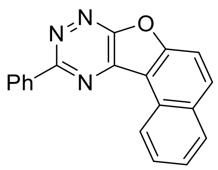

10-Phenylnaphtho[1',2':4,5]furo[3,2-*e*][1,2,4]triazine **4ia**: pale yellow solid. Yield 12 mg, 21%, m.p. 189–191 °C.

^{1}H NMR ($CDCl_3$): 9.09–8.99 (m, 1H, H-1), 8.75–8.64 (m, 2H, Ph), 8.24–8.14 (m, 1H, H-5), 8.03–7.94 (m, 1H, H-4), 7.86–7.73 (m, 2H, H-2, H-6), 7.66–7.52 (m, 4H, H-3, Ph);

^{13}C NMR ($CDCl_3$): 161.5, 160.8, 158.5, 143.9, 136.7, 135.7, 131.2, 130.7, 129.6, 129.3, 129.1, 129.0, 128.5, 126.7, 124.9, 113.7, 112.8.

Anal. Calcd. For $C_{19}H_{11}N_3O$: C, 76.76; H, 3.73; N, 14.13%; Found: C, 76.83; H, 3.70; N, 14.19%.

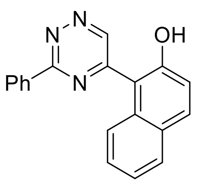	1-(3-Phenyl-1,2,4-triazin-5-yl)naphthalen-2-ol **5ia**: yellow solid. Yield 31 mg, 52%, m.p. 204–206 °C.<br>**^{1}H NMR** (DMSO-d_6): 12.45–12.16 (br s, 1H, OH), 9.77 (s, 1H, H-6′), 8.56–8.47 (m, 2H, Ph), 8.21–8.11 (m, 1H, H-8), 7.95 (d, 1H, *J*=9.0 Hz, H-4), 7.90–7.83 (m, 1H, H-5), 7.67–7.55 (m, 4H, H-6, Ph), 7.49–7.42 (m, 1H, H-7), 7.28 (d, 1H, *J*=9.0 Hz, H-3);<br>**^{13}C NMR** (DMSO-d_6): 161.3, 160.3, 155.9, 148.3, 135.8, 134.2, 132.4, 131.0, 129.6, 129.3 (2C), 128.7, 128.4, 124.7, 123.1, 119.6, 109.4.<br>Anal. Calcd. For $C_{19}H_{13}N_3O$: C, 76.24; H, 4.38; N, 14.04%; Found: C, 76.32; H, 4.45; N, 14.12%.
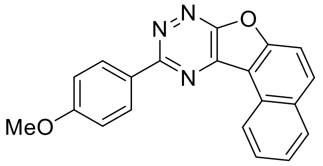<br>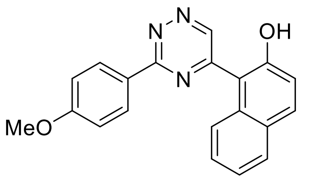	A mixture of **4ja** and **5ja** was separated by silica gel chromatography using *n*-hexane-ethyl acetate (17:1) to isolate **4ja** and *n*-hexane-ethyl acetate (7:1) to give **5ja**.<br>10-(4-Methoxyphenyl)naphtho[1',2':4,5]furo[3,2-*e*][1,2,4]triazine **4ja**: pale yellow solid. Yield 18 mg, 28%; m.p. 205–207 °C.<br>**^{1}H NMR** ($CDCl_3$): 9.05–8.99 (m, 1H, H-1), 8,64 (d, 2H, *J*=8.8 Hz, Ph), 8.19 (d, 1H, *J*=9.0 Hz, H-5), 8.02–7.94 (m, 1H, H-4), 7.85–7.78 (m, 1H, H-3), 7.76 (d, 1H, *J*=9.0 Hz, H-6), 7.66–7.58 (m, 1H, H-2), 7.08 (d, 2H, *J*=8.8 Hz, Ph), 3.93 (s, 3H, OC$\underline{H}_3$);<br>**^{13}C NMR** ($CDCl_3$): 162.3, 161.4, 160.6, 158.4, 143.9, 136.5, 130.7, 130.1, 129.5, 129.3, 129.1, 128.3, 126.7, 124.9, 114.3, 113.7, 112.8, 55.6.<br>Anal. Calcd. For $C_{20}H_{13}N_3O_2$: C, 73.38; H, 4.00; N, 12.84%; Found: C, 73.30; H, 3.92; N, 12.94%.<br>1-(3-(4-Methoxyphenyl)-1,2,4-triazin-5-yl)naphthalen-2-ol **5ja**: yellow solid. Yield 31 mg, 47%; m.p. 178–180 °C.<br>**^{1}H NMR** (DMSO-d_6): 12.33–12.08 (br s, 1H, OH), 9.59 (s, 1H, H-6′), 8.38 (d, 2H, *J*=8.7 Hz, Ph), 8.09–8.01 (m, 1H, H-8), 7.87–7.81 (m, 1H, H-4), 7.80–7.72 (m, 1H, H-5), 7.54–7.45 (m, 1H, H-6 or H-7), 7.39–7.30 (m, 1H, H-6 or H-7), 7.22–7.13 (m, 1H, H-3), 7.03–6.95 (d, 2H, *J*=8.7 Hz, Ph), 3.83 (s, 3H, OC$\underline{H}_3$);<br>**^{13}C NMR** (DMSO-d_6): 162.5, 156.8, 154.1, 151.0, 135.1, 132.6, 132.2, 131.6, 129.1, 128.4, 128.0, 127.8, 127.6, 123.4, 123.1, 118.1, 113.9, 55.7.<br>Anal. Calcd. For $C_{20}H_{15}N_3O_2$: C, 72.94; H, 4.59; N, 12.76%; Found: C, 72.83; H, 4.65; N, 12.70%.
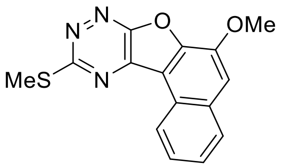	6-Methoxy-10-(methylthio)naphtho[1',2':4,5]furo[3,2-*e*][1,2,4]triazine **4ab**: yellow powder after recrystallization from MeCN. Yield 48 mg, 80%; m.p. 183–185 °C.<br>**^{1}H NMR** ($CDCl_3$): 8.55–8.51 (m, 1H, H-1 or H-4), 7.71–7.66 (m, 1H, H-1 or H-4), 7.55–7.50 (m, 1H, H-2 or H-3), 7.79–7.50 (m, 1H, H-2 or H-3), 7.32 (s, 1H, H-5), 4.13 (s, 3H, OC$\underline{H}_3$), 2.78 (s, 3H, SC$\underline{H}_3$);<br>**^{13}C NMR** ($CDCl_3$): 169.9, 159.9, 149.9, 145.0, 143.3, 131.2, 127.6, 126.9, 126.8, 124.3, 123.6, 114.1, 113.3, 56.5, 14.8.<br>Anal. Calcd. For $C_{15}H_{11}N_3O_2S$: C, 60.59; H, 3.73; N, 14.13%; Found: C, 60.67; H, 3.65; N, 14.04%.
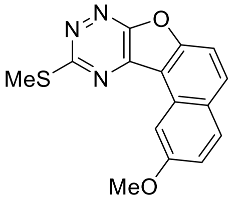	2-Methoxy-10-(methylthio)naphtho[1',2':4,5]furo[3,2-*e*][1,2,4]triazine **4ac**: yellow powder after recrystallization from MeCN. Yield 50 mg, 84%; m.p. 219–221 °C.<br>**^{1}H NMR** ($CDCl_3$): 8.22–8.19 (m, 1H, H-1), 8.14–8.10 (m, 1H, H-4), 7.88 (d, 1H, *J*=8.9 Hz, H-4), 7.57 (d, 1H, *J*=8.9 Hz, H-3), 7.27–7.22 (m, 1H, H-3), 4.06 (s, 3H, OC$\underline{H}_3$), 2.81 (s, 3H, SC$\underline{H}_3$);<br>**^{13}C NMR** ($CDCl_3$): 169.6, 161.0, 160.2, 159.4, 144.1, 136.8, 131.0, 125.7, 118.8, 112.0, 109.8, 104.2, 96.3, 55.8, 14.8.<br>Anal. Calcd. For $C_{15}H_{11}N_3O_2S$: C, 60.59; H, 3.73; N, 14.13%; Found: C, 60.50; H, 3.66; N, 14.10%.

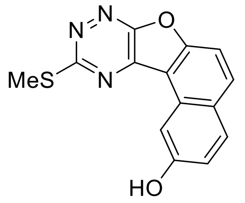	10-(Methylthio)naphtho[1',2':4,5]furo[3,2-*e*][1,2,4]triazin-2-ol **4ad**: according to the general procedure in mixture of $CHCl_3$:EtOH (4:1) as solvent, **4ad** was obtained as yellow powder after recrystallization from EtOH. Yield 38 mg, 68%; m.p. 282–284 °C.<br>**^{1}H NMR** (DMSO-d_6): 10.61 (s, 1H, OH), 8.30 (d, 1H, *J*=8.9 Hz, H-6), 8.03–7.97 (m, 2H, H-1, H-4), 7.70 (d, 1H, *J*=8.9 Hz, H-4), 7.57 (d, 1H, *J*=8.9 Hz, H-3), 7.19–7.14 (m, 1H, H-3), 2.76 (s, 3H, SC$\underline{H}_3$);<br>**^{13}C NMR** (DMSO-d_6): 168.0, 159.9, 159.1, 159.1, 144.0, 137.4, 131.6, 130.2, 124.4, 118.4, 110.5, 108.9, 106.5, 14.1.<br>Anal. Calcd. For $C_{14}H_9N_3O_2S$: C, 59.35; H, 3.20; N, 14.83%; Found: C, 59.25; H, 3.15; N, 14.75%.
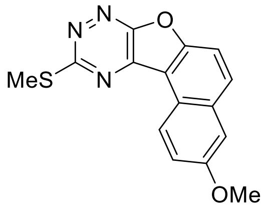	3-Methoxy-10-(methylthio)naphtho[1',2':4,5]furo[3,2-*e*][1,2,4]triazine **4ae**: yellow powder after recrystallization from MeCN. Yield 49 mg, 82%; m.p. 214–216 °C.<br>**^{1}H NMR** ($CDCl_3$): 8.83 (d, 1H, *J*=8.9 Hz, H-1), 8.13 (d, 1H, *J*=9.0 Hz, H-5), 7.74 (d, 1H, *J*=9.0 Hz, H-6), 7.47 (dd, 1H, *J*=8.9 Hz, *J*=2.5 Hz, H-2), 7.33 (d, 1H, *J*=2.5 Hz, H-4), 3.98 (s, 3H, OC$\underline{H}_3$), 2.83 (s, 3H, SC$\underline{H}_3$);<br>**^{13}C NMR** ($CDCl_3$): 169.7, 160.2, 158.3, 157.6, 143.9, 135.9, 132.2, 126.3, 123.8, 121.7, 113.1 (2C), 108.2, 55.6, 14.9.<br>Anal. Calcd. For $C_{15}H_{11}N_3O_2S$: C, 60.59; H, 3.73; N, 14.13%; Found: C, 60.65; H, 3.82; N, 14.10%.
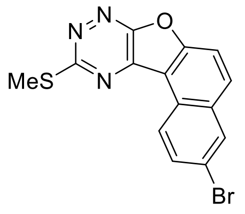	3-Bromo-10-(methylthio)naphtho[1',2':4,5]furo[3,2-*e*][1,2,4]triazine **4af**: yellow powder after recrystallization from toluene. Yield 53 mg, 75%; m.p. 244–246 °C.<br>**^{1}H NMR** ($CDCl_3$): 8.86 (d, 1H, *J*=8.7 Hz, H-1), 8.23 (d, 1H, *J*=1.8 Hz, H-4), 8.19 (d, 1H, *J*=9.1 Hz, H-5), 7.92 (dd, 1H, *J*=8.7 Hz, *J*=1.8 Hz, H-2), 7.85 (d, 1H, *J*=9.1 Hz, H-6), 2.85 (s, 3H, SC$\underline{H}_3$);<br>**^{13}C NMR** ($CDCl_3$): 170.2 160.3, 158.7, 143.6, 136.0, 133.0, 132.0, 131.5, 127.5, 126.6, 120.8, 114.1, 113.2, 14.9.<br>Anal. Calcd. For $C_{14}H_8BrN_3OS$: C, 48.57; H, 2.33; N, 12.14%; Found: C, 48.50; H, 2.26; N, 12.06%.
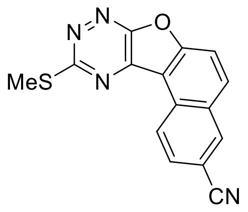	10-(Methylthio)naphtho[1',2':4,5]furo[3,2-*e*][1,2,4]triazine-3-carbonitrile **4ag**: yellow solid after recrystallization from MeCN. Yield 54 mg, 91%; m.p. 283–285 °C.<br>**^{1}H NMR** ($CDCl_3$): 9.09 (d, 1H, *J*=8.5 Hz, H-1), 8.44 (d, 1H, *J*=1.5 Hz, H-4) 8.33 (d, 1H, *J*=9.1 Hz, H-5), 7.99 (dd, 1H, *J*=8.5 Hz, *J*=1.5 Hz, H-2), 7.96 (d, 1H, *J*=9.1 Hz, H-6), 2.85 (s, 3H, SC$\underline{H}_3$);<br>**^{13}C NMR** ($CDCl_3$): 170.7, 160.4, 159.8, 143.3, 136.8, 134.9, 130.9, 130.6, 129.9, 126.3, 118.6, 115.1, 113.4, 110.7, 14.9.<br>Anal. Calcd. For $C_{15}H_8N_4OS$: C, 61.63; H, 2.76; N, 19.17%; Found: C, 61.72; H, 2.86; N, 19.22%.

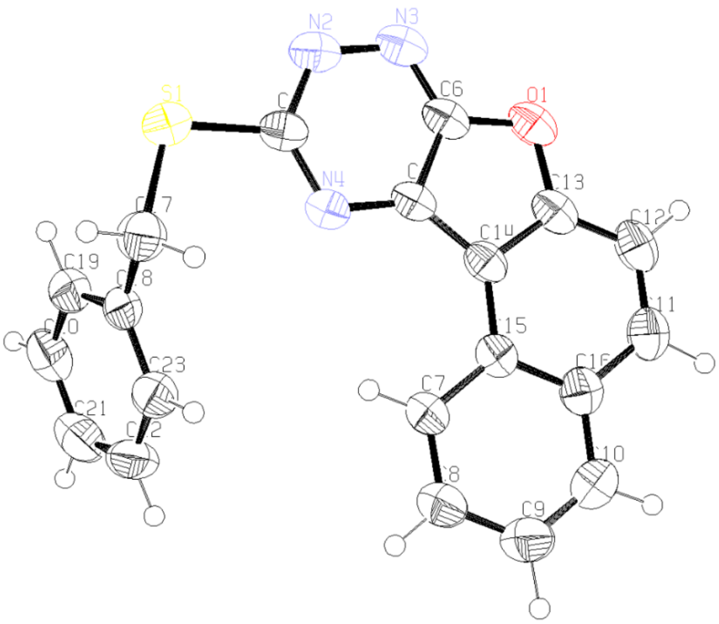

ORTEP diagram of compound **4fa** with ellipsoids at the 50% probability level.

4.3. Nucleophilic addition/oxidative cyclization sequence reactions

4.3.1. Synthesis of 10-alkyl 4ka,4la naphtho[1',2':4,5]furo[3,2-*e*][1,2,4]triazines

To a stirred solution of corresponding triazine **1k** or **1l** (1 mmol, 1 equiv.) in acetic acid (4 ml) 2-naphthol **2** (144 mg, 1 mmol, 1 equiv.) was added. Then the mixture was stirred at room temperature for 5 h, concentrated under reduced pressure, dissolved in $CHCl_3$ (10 ml) and washed wish saturated aq. $NaHCO_3$ solution (10 ml). The organic layer was dried over anhydrous Na_2SO_4 and filtered.. To the organic phase MnO_2 (261 mg, 3.0 mmol, 3 equiv.) was added in one portion and the mixture is stirred at 50 °C for 3 h. The reaction mixture is then cooled to room temperature. MnO_2 is filtered and washed with $CHCl_3$ (3×10 ml). The combined organic phase was concentrated under reduced pressure to give mixture of **4** and **5**, which was separated by chromatography on silica gel using mixture of *n*-hexane-ethyl acetate as eluent.

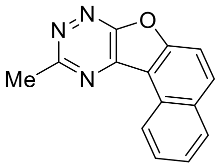	A mixture of **4ka** and **5ka** was separated by chromatography on silica gel using *n*-hexane-ethyl acetate (25:1) to isolate **4ka** and *n*-hexane-ethyl acetate (8:1) to give **5ka**.<br>10-Methylnaphtho[1',2':4,5]furo[3,2-*e*][1,2,4]triazine **4ka**: yellow solid. Yield 113 mg, 48%; m.p. 190–192 °C<br>**^{1}H NMR** ($CDCl_3$): 8.98–8.92 (m, 1H, H-1), 8.24–8.17 (m, 1H, H-5), 8.04–7.98 (m, 1H, H-4), 7.84–7.72 (m, 2H, H-2, H-6), 7.68–7.59 (m, 1H, H-3), 3.08 (s, 3H, C<u>H</u>$_3$);<br>**^{13}C NMR** ($CDCl_3$): 164.4, 160.6, 158.4, 144.0, 136.7, 130.7, 129.5, 129.3, 129.0, 126.7, 124.9, 113.4, 112.8, 23.9.<br>Anal. Calcd. For $C_{14}H_9N_3O$: C, 71.48; H, 3.86; N, 17.86%; Found: C, 71.39; H, 3.93; N, 17.92%.
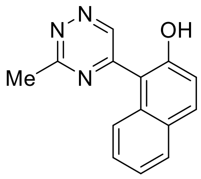	1-(3-Methyl-1,2,4-triazin-5-yl)naphthalen-2-ol **5ka**: pale yellow solid. Yield 45 mg, 19%; m.p. 168–170 °C<br>**^{1}H NMR** (DMSO-d_6): 10.46 (s, 1H, OH), 9.44 (s, 1H, H-6′), 8.01–7.96 (m, 1H, H-4), 7.92–7.85 (m, 1H, H-5 or H-8), 7.73–7.67 (m, 1H, H-5 or H-8), 7.45–7.29 (m, 3H, H-3, H-6, H-7), 2.85 (s, 3H, C<u>H</u>$_3$);

	**^{13}C NMR** (DMSO-d_6): 166.2, 156.4, 153.8, 150.2, 132.3, 132.1, 128.3, 127.9, 127.4, 123.3, 123.2, 118.1, 113.8, 23.6.<br>Anal. Calcd. For $C_{14}H_{11}N_3O$: C, 70.87; H, 4.67; N, 17.71%; Found: C, 70.77; H, 4.72; N, 17.80%.
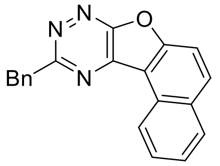<br><br>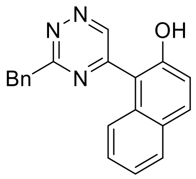	A mixture of **4la** and **5la** was separated by chromatography on silica gel using *n*-hexane-ethyl acetate (17:1) to give **4la** and *n*-hexane-ethyl acetate (10:1) to give **5la**.<br>10-Benzylnaphtho[1',2':4,5]furo[3,2-*e*][1,2,4]triazine **4la**: pale yellow solid. Yield 109 mg, 35%; m.p. 226–228 °C<br>**^{1}H NMR** (DMSO-d_6): 8.89–8.82 (m, 1H, H-1), 8.49 (d, 1H, *J*=9.0 Hz H-5), 8.25–8.20 (m, 1H, H-4), 8.06 (d, 1H, *J*=9.0 Hz H-6), 7.93–7.87 (m, 1H, H-2 or H-3), 7.74–7.69 (m, 1H, H-2 or H-3), 7.49–7.44 (m, 2H, Ph), 7.37–7.32 (m, 2H, Ph), 7.27–7.22 (m, 1H, Ph), 4.62 (s, 2H, CH_2);<br>**^{13}C NMR** (DMSO-d_6): 165.0, 160.2, 158.0, 143.9, 138.1, 137.0, 130.2, 129.5 (2C), 129.0 (2C), 128.3 (2C), 128.1, 126.4 (2C), 123.7, 112.9, 112.5, 42.7.<br>Anal. Calcd. For $C_{20}H_{13}N_3O$: C, 77.16; H, 4.21; N, 13.50%; Found: C, 77.25; H, 4.30; N, 13.57%.<br><br>1-(3-Methyl-1,2,4-triazin-5-yl)naphthalen-2-ol **5la**: pale yellow solid. Yield 110 mg, 35%; m.p. 155–157 °C<br>**^{1}H NMR** (DMSO-d_6): 10.55 (s, 1H, OH), 9.49 (s, 1H, H-6′), 8.01–7.94 (m, 1H, H-4), 7.90–7.84 (m, 1H, H-5 or H-8), 7.63–7.55 (m, 1H, H-5 or H-8), 7.43–7.22 (m, 8H, H-3, H-6, H-7, Ph), 4.47 (s, 2H, CH_2);<br>**^{13}C NMR** (DMSO-d_6): 167.9, 156.7, 154.0, 150.6, 137.7, 132.6, 132.0, 129.2, 128.5, 128.3, 128.0, 127.3, 126.6, 123.4, 123.1, 118.0, 113.5, 43.0.<br>Anal. Calcd. For $C_{20}H_{15}N_3O$: C, 76.66; H, 4.83; N, 13.41%; Found: C, 76.75; H, 4.74; N, 13.48%.

4.3.2. 10-Morpholinonaphtho[1',2':4,5]furo[3,2-*e*][1,2,4]triazine 4ma

To a stirred solution of 4-(1,2,4-triazin-3-yl)morpholine **1m** (1 mmol, 1 equiv.) and 2-naphtol **2a** (1 mmol, 1 equiv.) in methanol (4 ml) $BF_3{\cdot}OEt_2$ (370 µl, 3 mmol, 3 equiv.) was added dropwise and the resulting mixture was refluxed for 3 h. After cooling to room temperature the methanol was evaporated under reduced pressure, the residue was dissolved in $CHCl_3$ (10 ml) and washed with aq. $NaHCO_3$. Then the organic layer was dried over Na_2SO_4 and filtered. To the resulting solution MnO_2 (261 mg, 3 mmol, 3 equiv.) was added in one portion and the mixture was stirred at 50 °C for 3 h. The reaction mixture was cooled to room temperature. MnO_2 was filtered and washed with $CHCl_3$ (3×10 ml). The combined organic phase was concentrated under reduced pressure, and the residue was crystallized from MeCN to afford pure **4ma**.

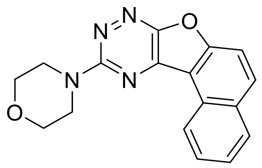	10-Morpholinonaphtho[1',2':4,5]furo[3,2-*e*][1,2,4]triazine **4ma**: yellow powder. Yield 225 mg, 75%; m.p. 230–232 °C.<br>**^{1}H NMR** ($CDCl_3$): 8.87–8.82 (m, 1H, H-1), 8.19 (d, 1H, *J*=9.1 Hz, H-5), 8.02–7.96 (m, 1H, H-4), 7.80–7.73 (m, 1H, H-3), 7.70 (d, 1H, *J*=9.1 Hz, H-6), 7.65–7.58 (m, 1H, H-2), 4.07–4.00 (m, 4H, morpholine), 3.95–3.88 (m, 4H, morpholine);<br>**^{13}C NMR** ($CDCl_3$): 161.1, 158.9, 157.7, 144.2, 136.0, 130.5, 129.3, 129.3, 129.2, 126.3, 124.6, 113.5, 113.0, 67.0, 45.1.<br>Anal. Calcd. For $C_{17}H_{14}N_4O_2$: C, 66.66; H, 4.61; N, 18.29%; Found: C, 66.75; H, 4.54; N, 18.36%.

4.3.3. Synthesis of benzofuro-fused triazines

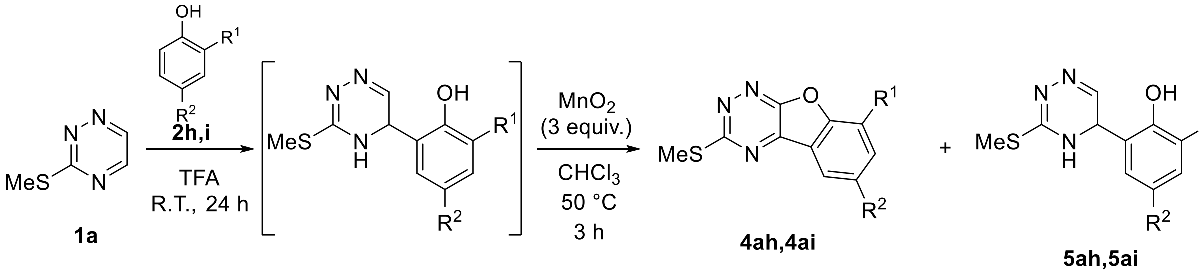

To a solution of triazine **1a** (127 mg, 1 mmol) in TFA (4 ml) a corresponding phenol **2h** or **2i** (1 mmol) was added and the resulting mixture was stirred at room temperature for 24 h. The completion of the reaction was monitored by TLC. Then the reaction mixture was concentrated under reduced pressure. The residue was dissolved in $CHCl_3$ (10 ml) and washed with aq. $NaHCO_3$. The organic layer was dried over Na_2SO_4 and filtered. MnO_2 (52 mg, 0.6 mmol, 3 equiv.) was added to the resulting solution in one portion and the mixture was stirred at 50 °C for 3 h, cooled to room temperature, and MnO_2 was filtered and the filter cake washed with $CHCl_3$ (3×10 ml). The combined organic phase was concentrated under reduced pressure. The residue was purified by silica gel chromatography to afford the pure product using *n*-hexane-ethyl acetate (80:1) to afford **4ah** or **4ai** and *n*-hexane-ethyl acetate (40:1) to give **5ah** or **5ai**.

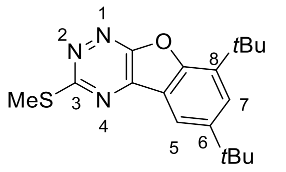<br>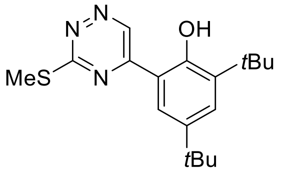	6,8-Di-*tert*-butyl-3-(methylthio)benzofuro[3,2-*e*][1,2,4]triazine **4ah**: yellow powder. Yield 167 mg, 51%; m.p. 105–107 °C.<br>**^{1}H NMR** ($CDCl_3$): 8.08 (d, 1H, *J*=1.8 Hz H-5), 7.78 (d, 1H, *J*=1.8 Hz, H-7), 2.79 (s, 3H, SCH_3), 1.57 (s, 9H, C(CH_3)$_3$), 1.41 (s, 9H, C(CH_3)$_3$);<br>**^{13}C NMR** ($CDCl_3$): 169.1, 160.7, 155.8, 148.6, 144.2, 136.1, 130.3, 118.8, 118.0, 35.4, 35.0, 31.7, 29.8, 14.8.<br>Anal. Calcd. For $C_{18}H_{23}N_3OS$: C, 65.62; H, 7.04; N, 12.75%; Found: C, 65.72; H, 7.10; N, 12.82%.<br><br>2,4-Di-*tert*-butyl-6-(3-(methylthio)-1,2,4-triazin-5-yl)phenol **5ah**: pale yellow powder. Yield 67 mg, 20%; m.p. 113–115 °C<br>**^{1}H NMR** ($CDCl_3$): 12.73 (s, 1H, OH), 9.50 (s, 1H, H-6′), 7.69 (d, 1H, *J*=2.3 Hz, H-5), 7.57 (d, 1H, *J*=2.3 Hz, H-3), 2.75 (s, 3H, SCH_3), 1.46 (s, 9H, C(CH_3)$_3$), 1.35 (s, 9H, C(CH_3)$_3$);<br>**^{13}C NMR** ($CDCl_3$): 170.1, 160.0, 156.0, 141.8, 141.6, 139.0, 130.9, 121.2, 113.0, 35.5, 34.6, 31.5, 29.5, 14.1.<br>Anal. Calcd. For $C_{18}H_{25}N_3OS$: C, 65.22; H, 7.60; N, 12.68%; Found: C, 65.31; H, 7.51; N, 12.74%.
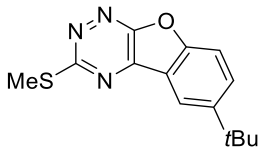	6-(*tert*-Butyl)-3-(methylthio)benzofuro[3,2-*e*][1,2,4]triazine **4ai**: yellow powder. Yield 30 mg, 11%; m.p. 136–138 °C.<br>**^{1}H NMR** ($CDCl_3$): 8.23 (d, 1H, *J*=1.8 Hz, H-5), 7.88 (dd, 1H, *J*=1.8 Hz, *J*=8.9 Hz, H-7), 7.61 (d, 1H, *J*=8.9 Hz, H-8), 2.78 (s, 3H, SCH_3), 1.42 (s, 9H, C(CH_3)$_3$);<br>**^{13}C NMR** ($CDCl_3$): 169.3, 161.0, 157.2, 149.0, 144.1, 133.5, 120.6, 118.5, 112.8, 35.3, 31.6, 14.8.<br>Anal. Calcd. For $C_{14}H_{15}N_3OS$: C, 61.52; H, 5.53; N, 15.37%; Found: C, 61.59; H, 5.44; N, 15.30%;

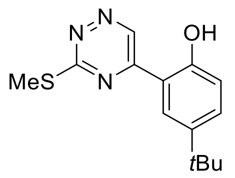	4-(*tert*-Butyl)-2-(3-(methylthio)-1,2,4-triazin-5-yl)phenol **5ai**: yellow powder. Yield 120 mg, 43%; m.p. 123–125 °C.<br>**^{1}H NMR** ($CDCl_3$): 11.97 (s, 1H, OH), 9.49 (s, 1H, H-6′), 7.80 (d, 1H, *J*=2.0 Hz, H-3), 7.53 (dd, 1H, *J*=2.0 Hz, *J*=8.8 Hz, H-5), 6.99 (d, 1H, *J*=8.8 Hz, H-8), 2.71 (s, 3H, SCH_3), 1.34 (s, 9H, $C(CH_3)_3$);<br>**^{13}C NMR** ($CDCl_3$): 170.6, 160.3, 155.3, 142.9, 141.2, 133.5, 123.2, 119.1, 113.2, 34.4, 31.4, 14.0.<br>Anal. Calcd. For $C_{14}H_{17}N_3OS$: C, 61.06; H, 6.22; N, 15.26%; Found: C, 61.13; H, 6.29; N, 15.16%.

5. Further modifications of compound 4aa

5.1. Liebeskind–Srogl coupling of SMe derivative

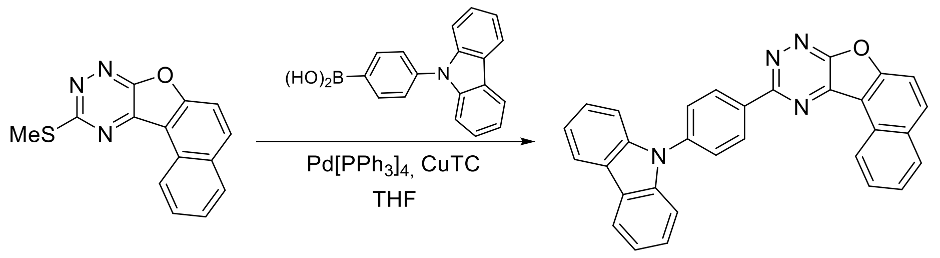

4aa **7**

To a solution of 10-(methylthio)naphtho[1',2':4,5]furo[3,2-*e*][1,2,4]triazine **4aa** (100 mg, 1 equiv.) in dry THF (5 ml) was added CuTC (249 mg, 3.5 equiv), $Pd[PPh_3]_4$ (43 mg, 10 mol%) and (4-(9*H*-carbazol-9-yl)phenyl)boronic acid (322 mg, 3 eqeiv.). Then the reaction mixture was stirred at reflux for 32 hours. The progress of the reaction was monitored by TLC. After completion, the solvent was evaporated under reduced pressure and the residue was purified by flash chromatography using *n*-hexane-ethyl acetate (10:1→5:1) to give a pure product **7**.

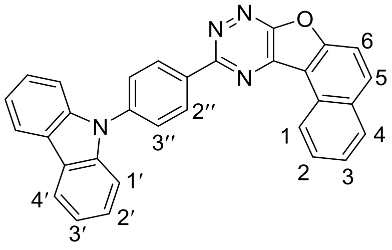	10-(4-(Carbazol-9-yl)phenyl)naphtho[1',2':4,5]furo[3,2-*e*][1,2,4]triazine **7**: yellow powder. Yield 126 mg, 73%; m.p. 280–282 °C.<br>**^{1}H NMR** ($CDCl_3$): 9.22–9.18 (m, 1H, H-1), 9.01–8.97 (m, 2H, H-2″), 8.34-8.29 (m, 1H, H-5), 8.20–8.16 (m, 2H, H-1′), 8.11–8.08 (m, 1H, H-4), 7.94–7.82 (m 4H, H-2, H-6, H-3″), 7.74–7.70 (m, 1H, H-3), 7.60–7.57 (m, 2H, H-4′), 7.48–7.43 (m, 2H, H-2′ or H3′), 7.36–7.31 (m, 2H, H-2′ or H3′);<br>**^{13}C NMR** ($CDCl_3$): 161.1, 161.0, 158.8, 144.3, 140.7, 140.5, 137.1, 134.5, 130.9, 130.2, 129.8, 129.6, 129.2, 127.2, 126.9, 126.3, 125.1, 123.8, 120.6, 120.5, 113.8, 112.9, 110.1.<br>Anal. Calcd. For $C_{31}H_{18}N_4O$: C, 80.50; H, 3.92; N, 12.11%; Found: C, 80.31; H, 4.07; N, 11.96%.

5.2 Oxidation of SMe-group with *m*CPBA

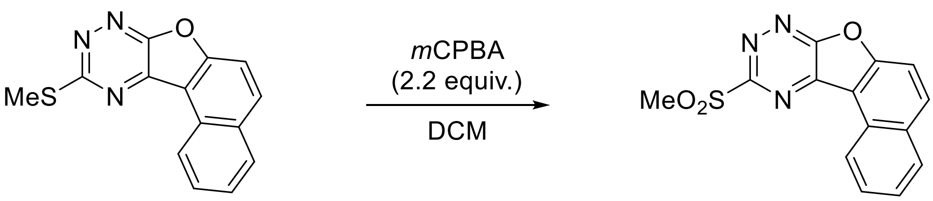

4aa **8**

*m*CPBA (427 mg, ≤77%, 2.2 equiv.) was dissolved in dry DCM (5 ml), Na_2SO_4 (2.0 g) was added to the resulting solution and the mixture was stirred for 10 min. Na_2SO_4 was filtered and washed with DCM (3×5 ml). The obtained solution of *m*CPBA was added dropwise to a solution of **4aa** (133 mg, 0.5 mmol) in DCM (4 ml) at 0 °C. Then the reaction mixture was stirred at room temperature for 12 h. Progress of the reaction was monitored by TLC. After completion the reaction, the mixture was quenched with aqueous solution of $NaHCO_3$, washed with water, dried over Na_2SO_4 and the solvent was evaporated under reduced pressure. The residue was purified by flash chromatography using *n*-hexane-chloroform (2:1) as eluent to give pure **8**.

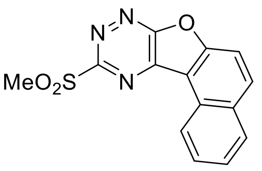	10-(Methylsulfonyl)naphtho[1',2':4,5]furo[3,2-*e*][1,2,4]triazine **8**: yellow powder. Yield 127 mg, 85%; m.p. 248–251 °C.<br>^{1}**H NMR** ($CDCl_3$): 9.07–9.04 (m, 1H, H-1), 8.43–8.39 (m, 1H, H-5), 8.11–8.08 (m, 1H, H-4), 7.93–7.88 (m, 2H, H-2, H-6), 7.76–7.72 (m, 1H, H-3), 7.65–7.58 (m, 1H, H-2), 3.66 (s, 2H, $SO_2C\underline{H}_3$);<br>^{13}**C NMR** ($CDCl_3$): 163.8, 161.6, 160.4, 145.3, 139.5, 131.0, 130.7, 129.7, 128.7, 127.8, 125.5, 113.0, 112.6, 40.6.<br>Anal. Calcd. For $C_{14}H_9N_3O_3S$: C, 56.18; H, 3.03; N, 14.04%; Found: C, 56.05; H, 3.20; N, 13.96%.

5.3 Substitution of SO_2Me-group with carbazole

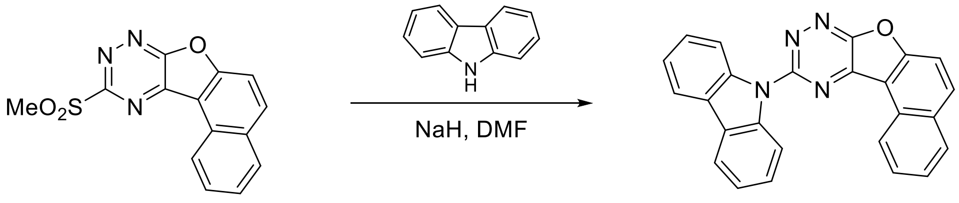

7 9

To a solution of carbazole (106 mg, 1.9 equiv.) in dry DMF (3 mL) was added NaH (60% suspension in mineral oil, 19 mg 1,4 equiv.) and the mixture was stirred for 10 min. Then methylsulfonyl derivative **8** (100 mg, 0.33 mmol) was added to the resulting solution and the mixture was heated at 70 °C for 12 h. After completion the reaction, the mixture was diluted with water (15 ml), the forming precipitate was filtered and washed with water and ethanol and purified by flash chromatography using n-hexane:chloroform (2:1) as eluent to give pure **9**.

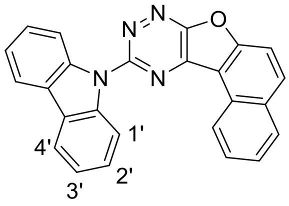	10-(Carbazol-9-yl)naphtho[1',2':4,5]furo[3,2-*e*][1,2,4]triazine **9**: yellow powder. Yield 71 mg, 55%; m.p. 250–253 °C.<br>^{1}**H NMR** (HMPA d-18): 9.02–8.98 (m, 1H, H-1), 8.95-8.90 (d, *J*=9.1 Hz, 1H, H-5), 8.85–8.78 (m, 2H, H-1′), 8.58–8.54 (m, 1H, H-4), 8.51–8.46 (m, 2H, H-4′), 8.42 (d, *J*=9.1 Hz, 1H, H-6), 8.15–8.09 (m, 1H, H-2 or H-3), 7.89–7.82 (m, 1H, H-2 or H-3), 7.68–7.61 (m, 2H, H-2′ or H-3′), 7.50–7.43 (m, 2H, H-2′ or H-3′);<br>^{13}**C NMR** (HMPAd-18): 160.2, 159.8, 157.9, 144.5, 139.4, 139.3, 131.5, 130.9, 130.6, 128.9, 127.3, 127.1, 126.0, 124.0, 122.9, 121.0, 115.1, 113.8, 113.2.<br>Anal. Calcd. For $C_{25}H_{14}N_4O$: C, 77.71; H, 3.65; N, 14.50%; Found: C, 77.90; H, 2.81; N, 14.29%.

6. Preliminary mechanistic studies

6.1. Radical trap experiment using TEMPO

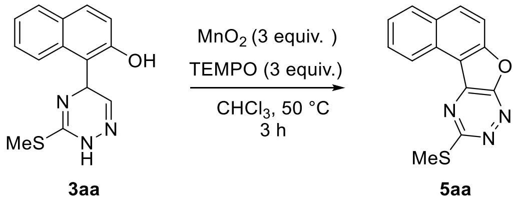

To a stirred solution of **3aa** (54 mg, 0.2 mmol, 1 equiv.) and TEMPO (93 mg, 0.6 mmol, 3 equiv.) in $CHCl_3$ (5 ml) was added MnO_2 (52 mg, 0.6 mmol, 3 equiv.) in one portion. The resulting mixture was stirred at 50 °C for 3 h. The reaction mixture was then cooled to room temperature. MnO_2 was filtered and the filter cake washed with $CHCl_3$ (3×10 mL). The combined organic phase was concentrated under reduced pressure. The residue was recrystallized form MeCN to afford the **4aa** (43.2 mg, 81%).

6.2. Radical trap experiment using BHT

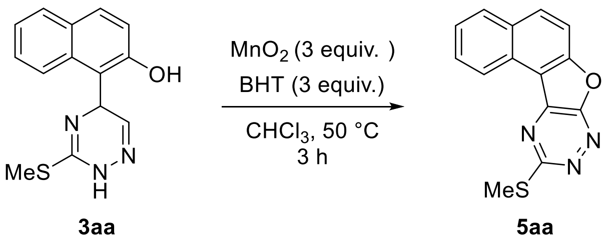

To a stirred solution of **3aa** (54 mg, 0.2 mmol, 1 equiv.) and BHT (132 mg, 0.6 mmol, 3 equiv.) in $CHCl_3$ (5 mL) was added MnO_2 (52 mg, 0.6 mmol, 3 equiv.) in one portion. The resulting mixture was stirred at 50 °C for 3 h. The reaction mixture was then cooled to room temperature. MnO_2 was filtered and washed with $CHCl_3$ (3×10 mL). The combined organic phase was concentrated under reduced pressure. The residue was recrystallized form MeCN to afford the **4aa** (42.7 mg, 80%).

6.3. EPR spectroscopic investigation

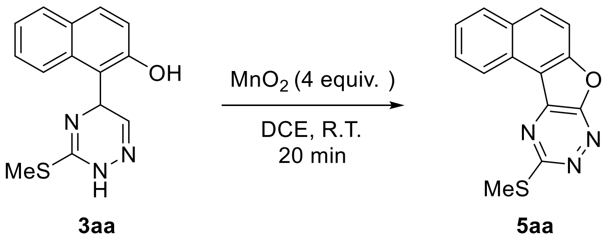

To 0.1 M solution of **3aa** (136 mg, 0.5 mmol) in DCE (5 ml) MnO_2 (174 mg, 2.0 mmol, 4 equiv.) was added and the resulting mixture was stirred at R.T. for 20 min. A sample solution (0.5 ml) was analyzed by direct-detection EPR spectroscopy and a distinct EPR signal is detected (Figure S1). The subsequent simulation of the spectrum of Mn^{2+} is in agreement with experiment and literature data.[21]

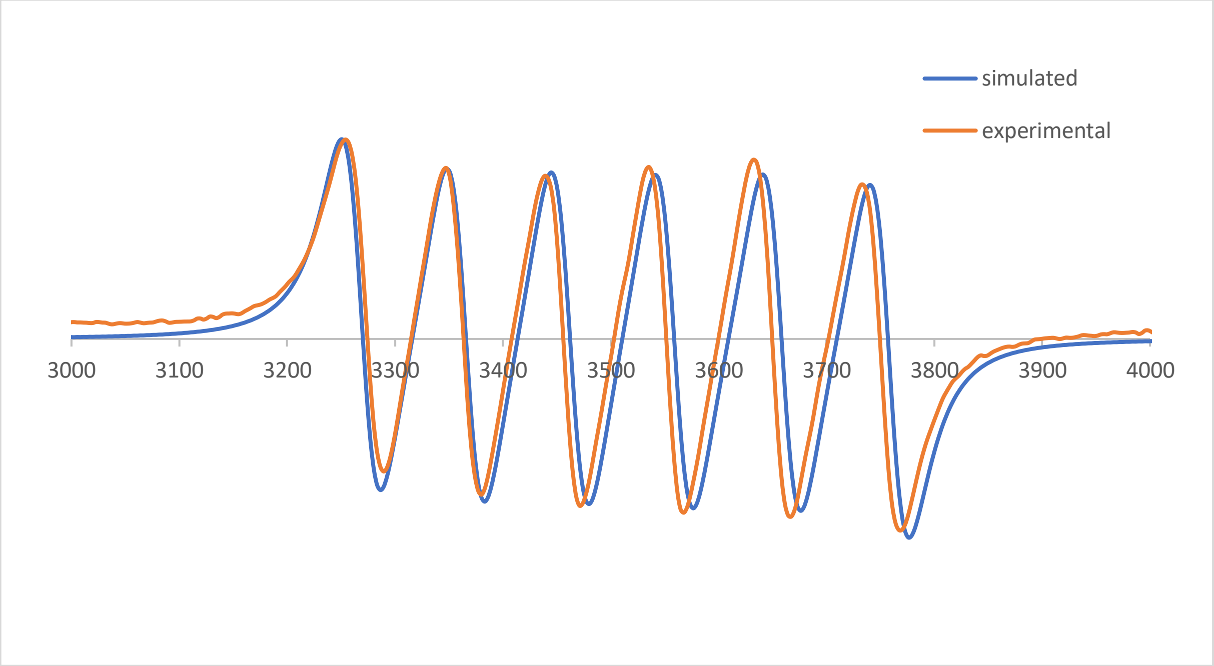

Figure S1. Experimental EPR spectrum of the mixture of **3aa** and MnO_2 (the red line) and the simulated EPR spectrum for Mn^{2+} (the blue line).

6.4.Synthesis of possible intermediates

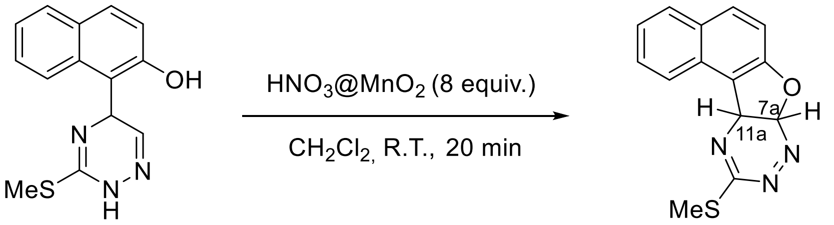

3aa **4aa'**

To a stirred solution of **3aa** (136 mg, 0.5 mmol, 1 equiv.) in CH_2Cl_2 (10 mL) was added HNO_3@MnO_2[19] (348 mg, 8 mmol, 8 equiv.) in one portion. The resulting mixture was stirred at room temperature for 20 min and then MnO_2 was filtered *via* silica gel pad using CH_2Cl_2 as eluent. The resulting red solution was concentrated under reduced pressure to give **4aa′** (90 mg, 67%).

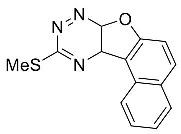<br>Chemical Formula: $C_{14}H_{11}N_3OS$<br>Exact Mass: 269,0623	10-(Methylthio)-7a,11a-dihydronaphtho[1',2':4,5]furo[3,2-*e*][1,2,4]triazine **4aa′**: pale red solid. M.p. 115–117 °C.<br>**^{1}H NMR** ($CDCl_3$): 8.29–8.22 (m, 1H, H-1), 7.84–7.78 (m, 1H, H-4), 7.75 (d, 1H, *J*=8.8 Hz, H-5), 7.58–7.52 (m, 1H, H-3), 7.42–7.36 (m, 1H, H-2), 7.23 (d, 1H, *J*=8.8 Hz, H-6), 5.69 (d, 1H, *J*=10.6 Hz, H-7a), 5.66 (d, 1H, *J*=10.6 Hz, H-11a), 2.52 (s, 3H, SMe);<br>**^{13}C NMR** ($CDCl_3$): 155.7, 154.1, 131.3, 130.2, 130.1, 128.8, 127.4, 124.1, 123.4, 116.4, 112.1, 86.7, 53.9, 13.5.<br>**HRMS**: Calcd. for $C_{14}H_{12}N_3OS$ $[M+H]^+$: 270.0701; found 270.0696.

Qualitative Compound Report

Data File	FR 1230.d	Sample Name	FR 1230
Sample Type	Sample	Position	P1-B8
Instrument Name	LCMS	User Name	Vadim A. Shevyrin
Acq Method	DIRECT.m	Acquired Time	1/13/2022 12:26:44 PM (UTC+05:00)
IRM Calibration Status	Success	DA Method	Main.m
Comment			
Sample Group		Info.	
Stream Name	LC 1	Acquisition Time (Local)	1/13/2022 12:26:44 PM (UTC+05:00)
Acquisition SW Version	6200 series TOF/6500 series Q-TOF B.08.00 (B8058.3 SP1)	QTOF Driver Version	8.00.00
QTOF Firmware Version	20.712	Tune Mass Range Max.	1700

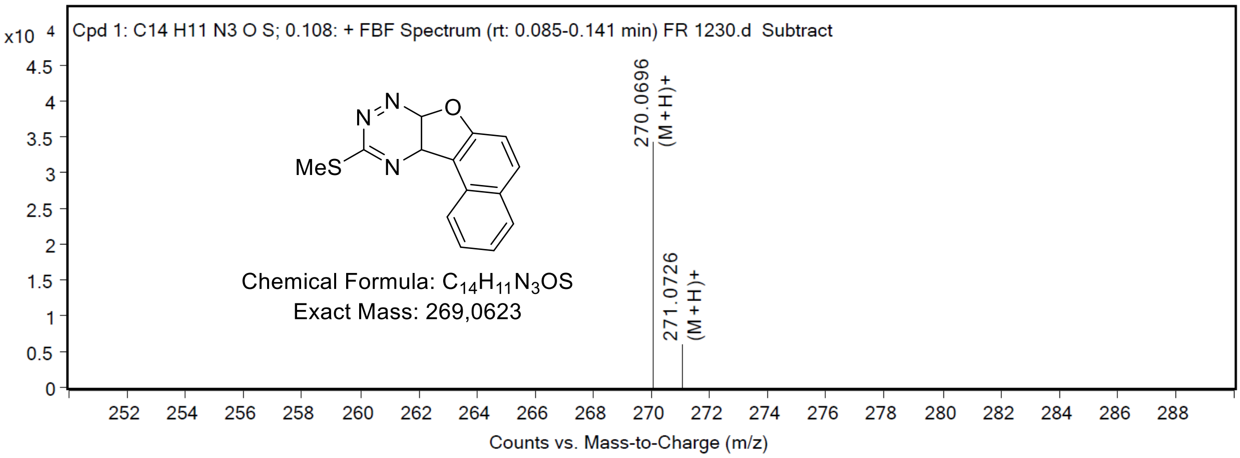

Figure S2. Mass-spectrum of compound **4aa′**.

Synthesis of 4aa″

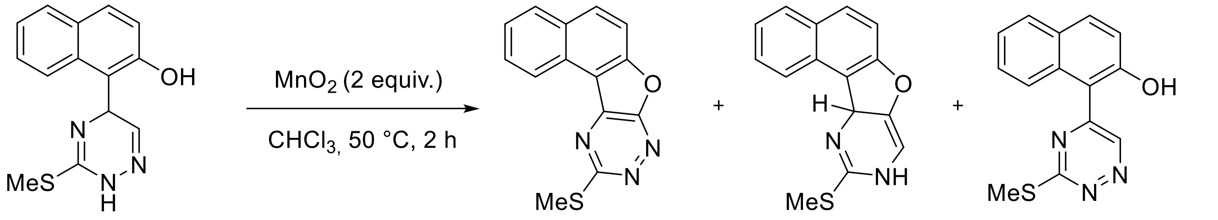

3aa **4aa** **4aa'** **5aa**

To a stirred solution of **3aa** (542 mg, 2 mmol, 1 equiv.) in $CHCl_3$ (20 mL) MnO_2 (348 mg, 4 mmol, 2 equiv.) was added in one portion. The resulting mixture was stirred at 50 °C for 2 h. The reaction mixture was then cooled to room temperature, MnO_2 was filtered and the filter cake was washed with $CHCl_3$ (4×10 mL). The combined organic phase was concentrated under reduced pressure and the residue was recrystallized form MeCN to afford **4aa** (448 mg, 84%). The residue was analyzed by ^{1}H NMR spectroscopy in $CDCl_3$ solution (Figure S3).

The representative peaks of **4aa** and **5aa**[13] as minor products were detected in ^{1}H NMR spectrum (Figure S3). In addition, peak (δ=5.16, $CDCl_3$) of major unknown compound is correlated with peak (δ=5.13, $CDCl_3$) of proton at the C-5 of triazine ring of compound **3aa** (Figure S3). In the same time, representative peak (δ=6.83, $CDCl_3$, Figure S3, A) of proton at the C-6 of triazine ring of compound **3aa** was lack in spectrum of mixture (See Figure S4, **A** vs **C**). All attempts to isolate the unknown compound from the MeCN residue were failed and compound **4aa** was separated as

major component of mixture. Based on both experimental data and our own logical inferences, we assigned the structure of unknown compound as **4aa″**.

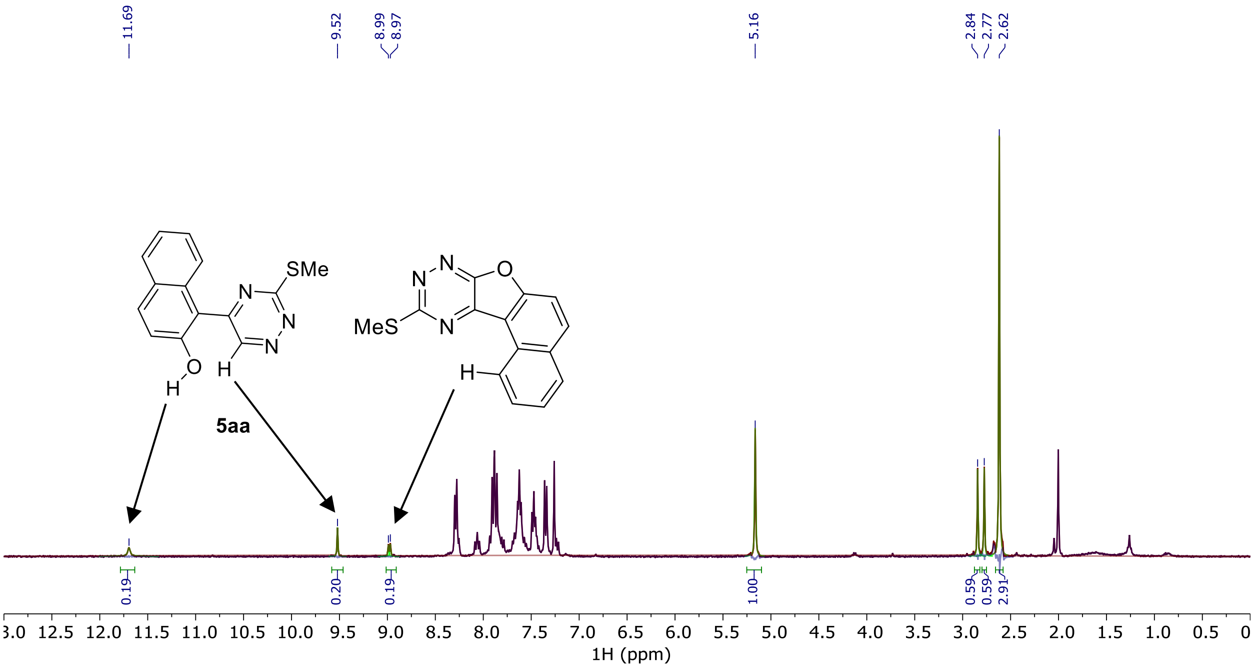

Figure S3. ^{1}H NMR spectrum of the residue

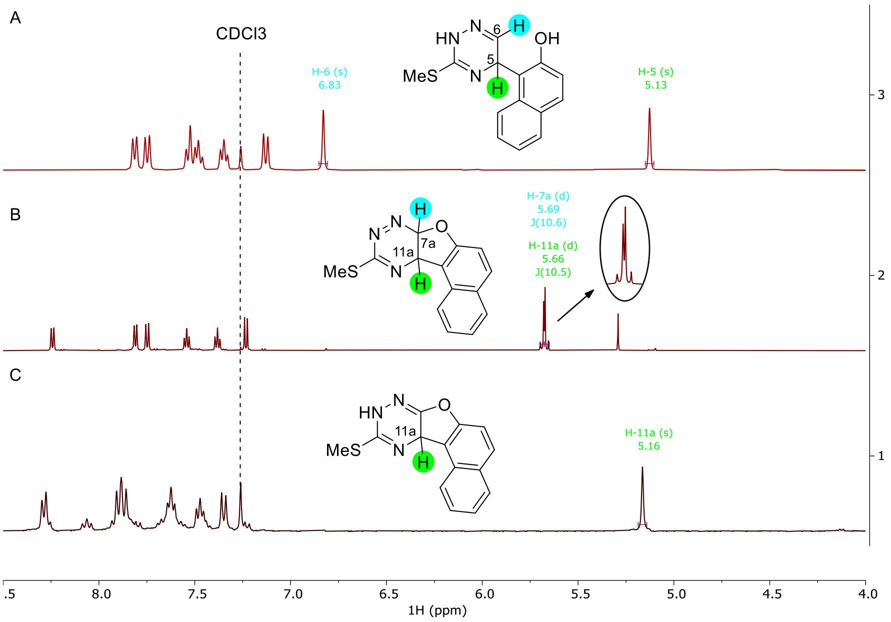

Figure S4. Comparison of the 1H NMR spectra of **3aa**, **4aa′**, **4aa″**.

7. DFT calculations

	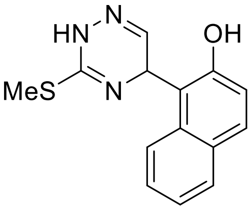 **3aa**	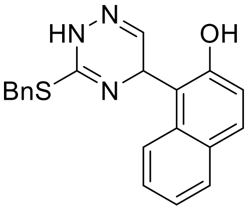 **3fa**	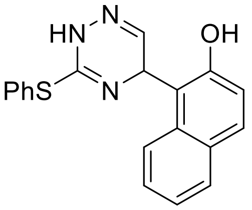 **3ha**	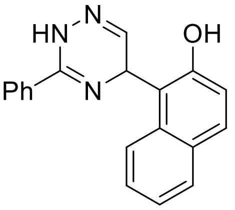 **3ia**
E_{INT}, Ht	-1179.180823	-1012.071649	-1370.957872	-972.7509272
E_G, Ht	-1178.978730	-1011.794681	-1370.708670	-972.4985440
HOMO	-0.21799	-0.21570	-0.21822	-0.21495
HOMO-1	-0.23930	-0.23905	-0.23796	-0.23600
ΔE, eV	0.579875	0.635386	0.537153	0.572800

References

[1] S. Stoll, A. Schweiger, *J. Magn. Reson.* **2006**, *178*, 42–55.

[2] A. D. Becke, *J. Chem. Phys.* **1993**, *98*, 5648–5652.

[3] R. Krishnan, J. S. Binkley, R. Seeger, J. A. Pople, *J. Chem. Phys.* **1980**, *72*, 650–654.

[4] A. D. McLean, G. S. Chandler, *J. Chem. Phys.* **1980**, *72*, 5639–5648.

[5] Gaussian 09, Revision D.01, M. J. Frisch, G. W. Trucks, H. B. Schlegel, G. E. Scuseria, M. A. Robb, J. R. Cheeseman, G. Scalmani, V. Barone, G. A. Petersson, H. Nakatsuji, X. Li, M. Caricato, A. Marenich, J. Bloino, B. G. Janesko, R. Gomperts, B. Mennucci, H. P. Hratchian, J. V. Ortiz, A. F. Izmaylov, J. L. Sonnenberg, D. Williams-Young, F. Ding, F. Lipparini, F. Egidi, J. Goings, B. Peng, A. Petrone, T. Henderson, D. Ranasinghe, V. G. Zakrzewski, J. Gao, N. Rega, G. Zheng, W. Liang, M. Hada, M. Ehara, K. Toyota, R. Fukuda, J. Hasegawa, M. Ishida, T. Nakajima, Y. Honda, O. Kitao, H. Nakai, T. Vreven, K. Throssell, J. A. Montgomery, Jr., J. E. Peralta, F. Ogliaro, M. Bearpark, J. J. Heyd, E. Brothers, K. N. Kudin, V. N. Staroverov, T. Keith, R. Kobayashi, J. Normand, K. Raghavachari, A. Rendell, J. C. Burant, S. S. Iyengar, J. Tomasi, M. Cossi, J. M. Millam, M. Klene, C. Adamo, R. Cammi, J. W. Ochterski, R. L. Martin, K. Morokuma, O. Farkas, J. B. Foresman, and D. J. Fox, Gaussian, Inc., Wallingford CT, **2016**.

[6] *GaussView, Version 6, Roy Dennington, Todd Keith, and John Millam, Semichem Inc., Shawnee Mission, KS*, **2019**.

[7] K. Ban, S. Duffy, Y. Khakham, V. M. Avery, A. Hughes, O. Montagnat, K. Katneni, E. Ryan, J. B. Baell, *Bioorg. Med. Chem. Lett.* **2010**, *20*, 6024–6029.

[8] E. C. Taylor, J. E. Macor, J. L. Pont, *Tetrahedron* **1987**, *43*, 5145–5158.

[9] von Hans Neunhoeffer, H. Hennig, H.-W. Frühauf, M. Mutterer, *Tetrahedron Lett.* **1969**, *10*, 3147–3150.

[10] O. P. Shkurko, L. L. Gogin, S. G. Baram, V. P. Mamaev, *Chem. Heterocycl. Compd.* **1987**, *23*, 216–221.

[11] B. Courcot, D. N. Tran, B. Fraisse, F. Bonhomme, A. Marsura, N. E. Ghermani, *Chem. – Eur. J.* **2007**, *13*, 3414–3423.

[12] S. G. Alekseev, V. N. Charushin, O. N. Chupakhin, S. V. Shorshnev, A. I. Chernyshev, N. A. Klyuev, *Chem. Heterocycl. Compd.* **1986**, *22*, 1242–1249.

[13] I. A. Utepova, A. I. Nemytov, V. A. Ishkhanian, O. N. Chupakhin, V. N. Charushin, *Tetrahedron* **2020**, *76*, 131391.

[14] R. F. Fatykhov, M. I. Savchuk, E. S. Starnovskaya, M. V. Bobkina, D. S. Kopchuk, E. V. Nosova, G. V. Zyryanov, I. A. Khalymbadzha, O. N. Chupakhin, V. N. Charushin, V. G. Kartsev, *Mendeleev Commun.* **2019**, *29*, 299–300.

[15] O. N. Chupakhin, G. L. Rusinov, N. A. Itsikson, D. G. Beresnev, *Russ. Chem. Bull.* **2004**, *53*, 1351–1352.

[16] O. N. Chupakhin, G. L. Rusinov, N. A. Itsikson, D. G. Beresnev, O. V. Fedorova, I. G. Ovchinnikova, *Russ. Chem. Bull.* **2004**, *53*, 2308–2313.
[17] L. I. Vereshagin, S. R. Gainulina, S. A. Podskrebysheva, L. A. Gaivoroskii, L. L. Okhapina, V. G. Vorob'eva, V. P. Latyshev, *J Org Chem USSR* **1972**, *8*, 1143.
[18] O. Glemser, G. Gattow, H. Meisiek, *Z. Für Anorg. Allg. Chem.* **1961**, *309*, 1–19.
[19] R. Cassis, J. A. Valderrama, *Synth. Commun.* **1983**, *13*, 347–356.
[20] B. Nammalwar, C. Fortenberry, R. A. Bunce, S. K. Lageshetty, K. D. Ausman, *Tetrahedron Lett.* **2013**, *54*, 2010–2013.
[21] J. R. Bard, J. T. Holman, J. O. Wear, *Z. Für Naturforschung B* **1969**, *24*, 989–993.

Copies of ^{1}H and ^{13}C NMR spectra for compounds 1

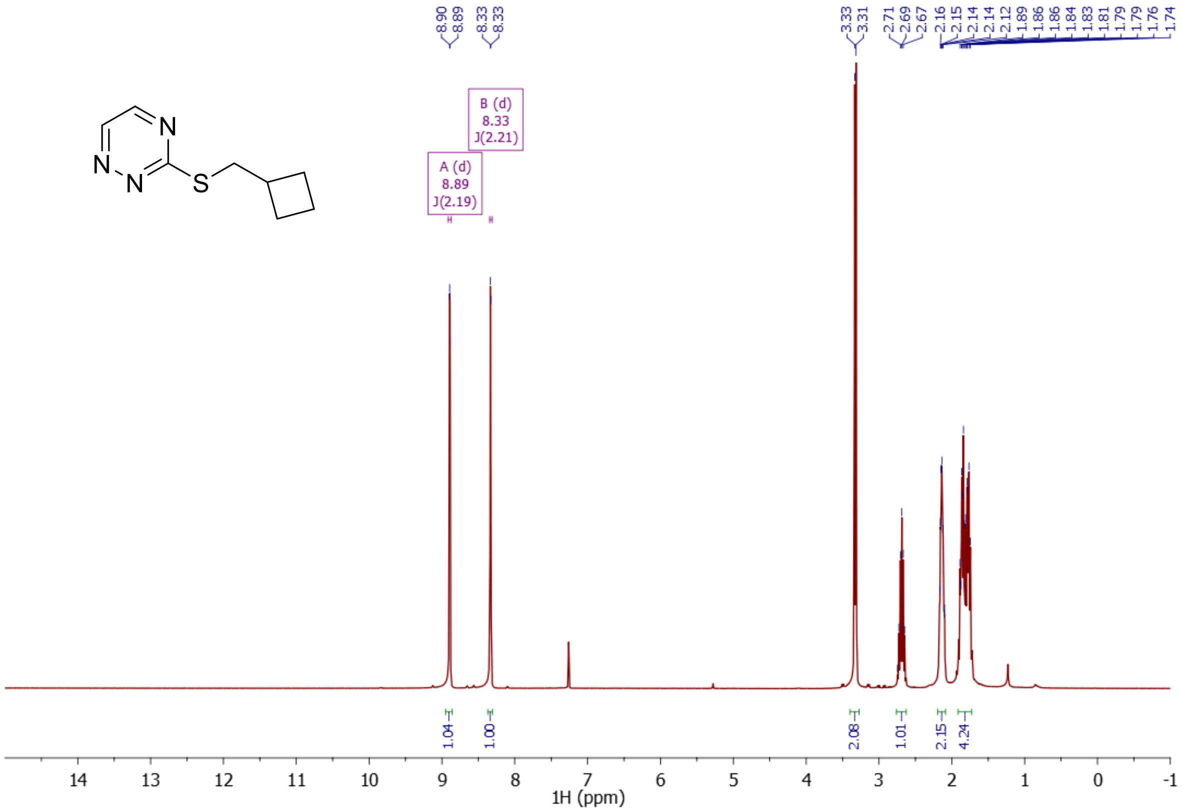

^{1}H NMR spectrum of 3-((cyclobutylmethyl)thio)-1,2,4-triazine **1d**

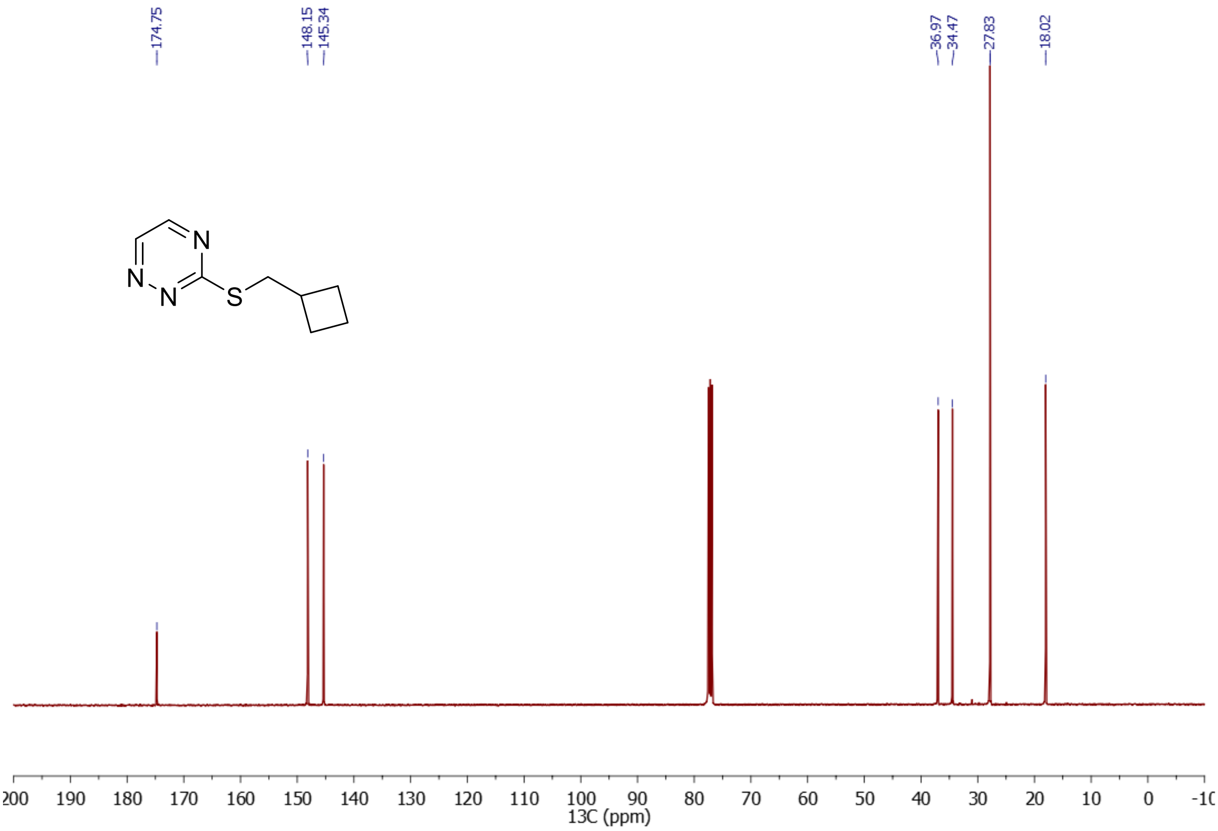

^{13}C NMR spectrum of 3-((cyclobutylmethyl)thio)-1,2,4-triazine **1d**

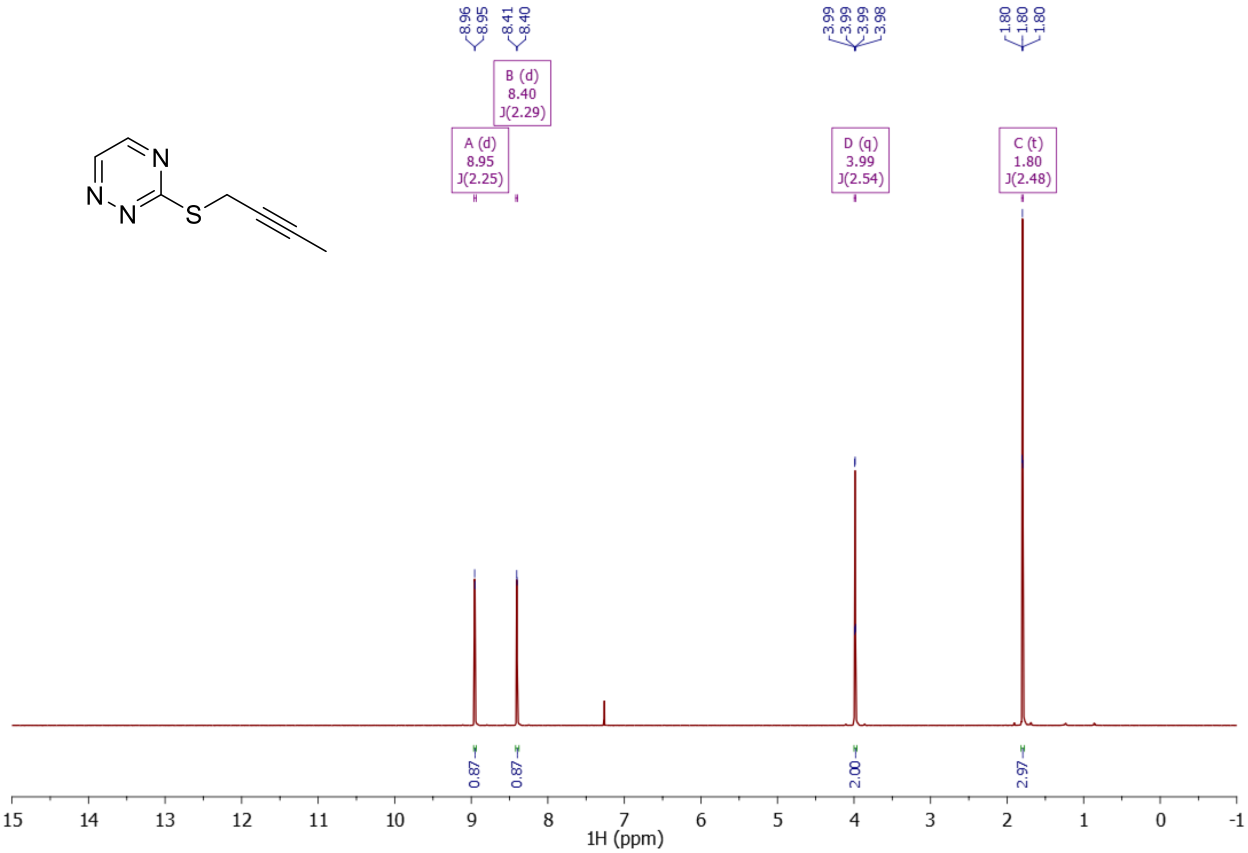

^{1}H NMR spectrum of 3-(but-2-yn-1-ylthio)-1,2,4-triazine **1e**

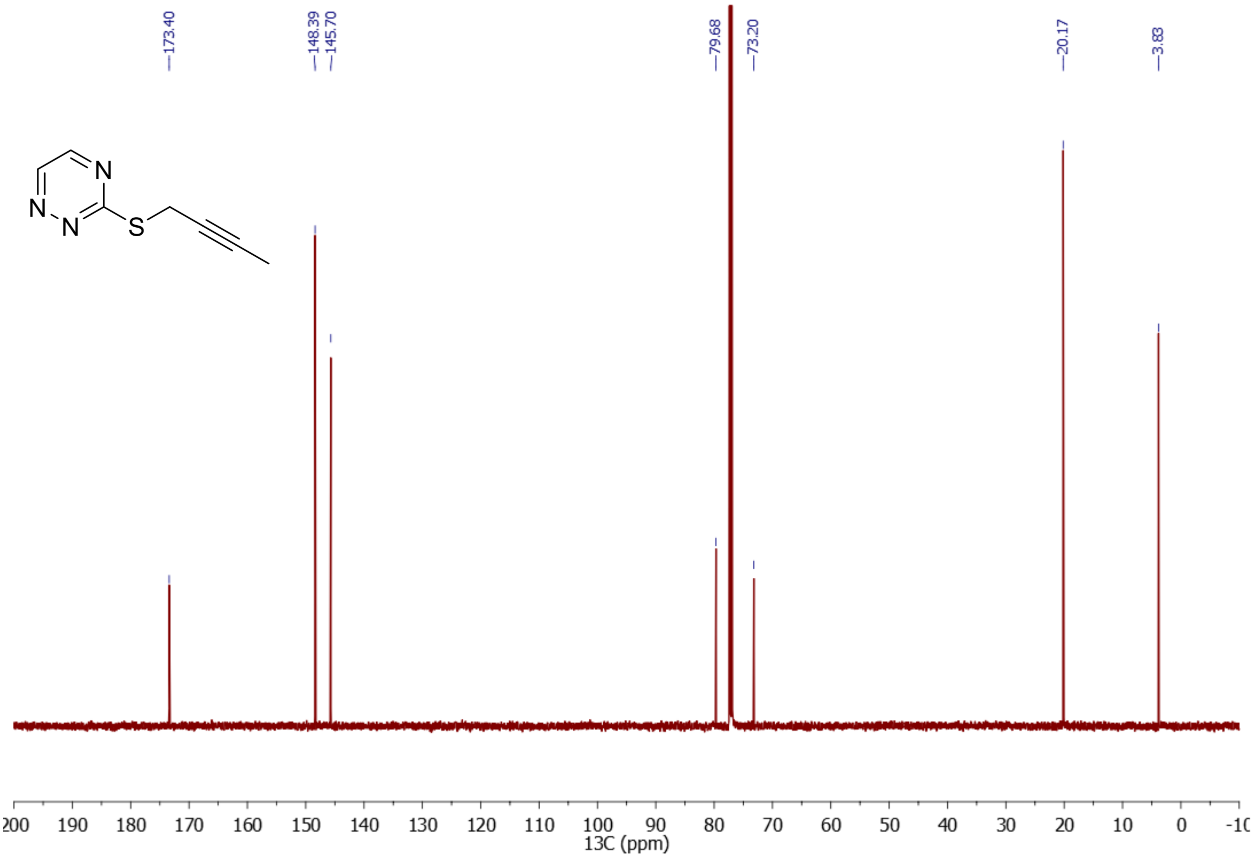

^{13}C NMR spectrum of 3-(but-2-yn-1-ylthio)-1,2,4-triazine **1e**

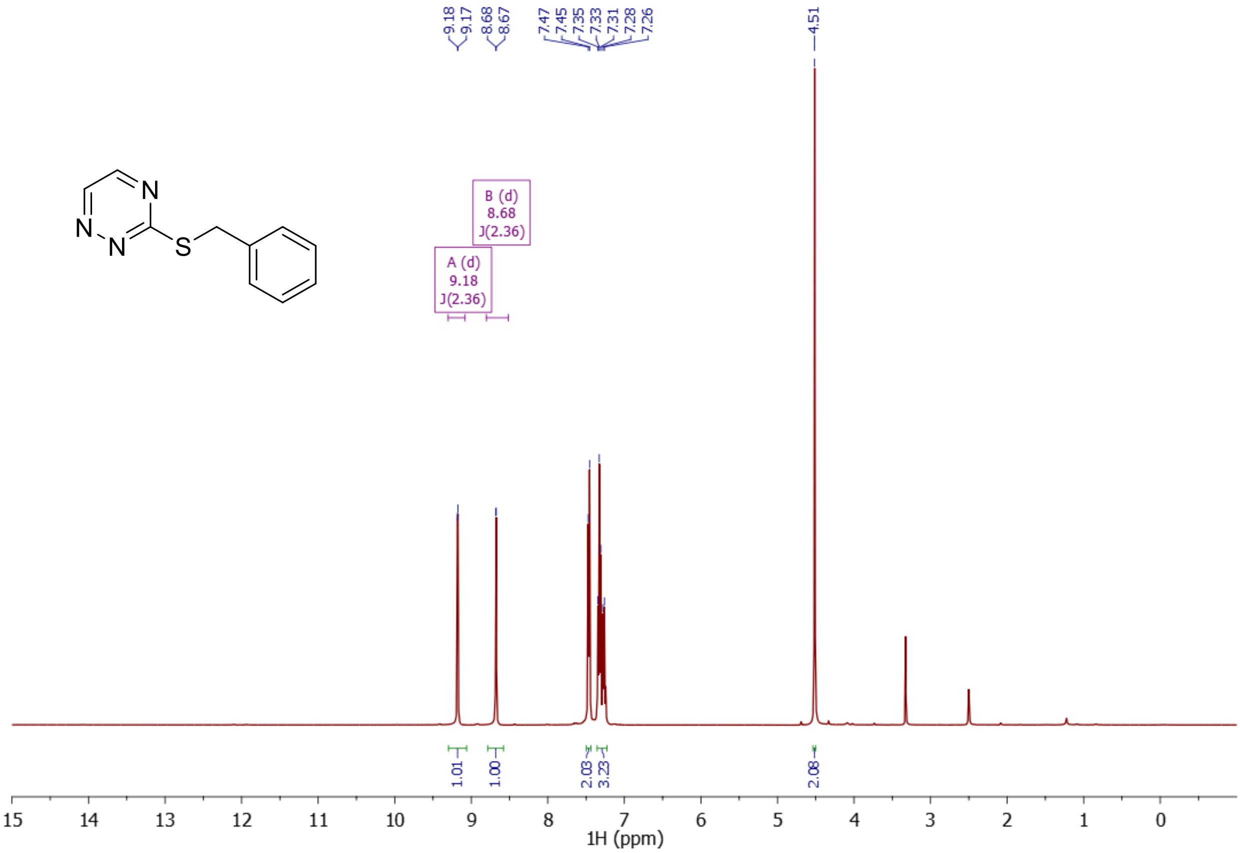

^{1}H NMR spectrum of 3-benzylthio-1,2,4-triazine **1f**

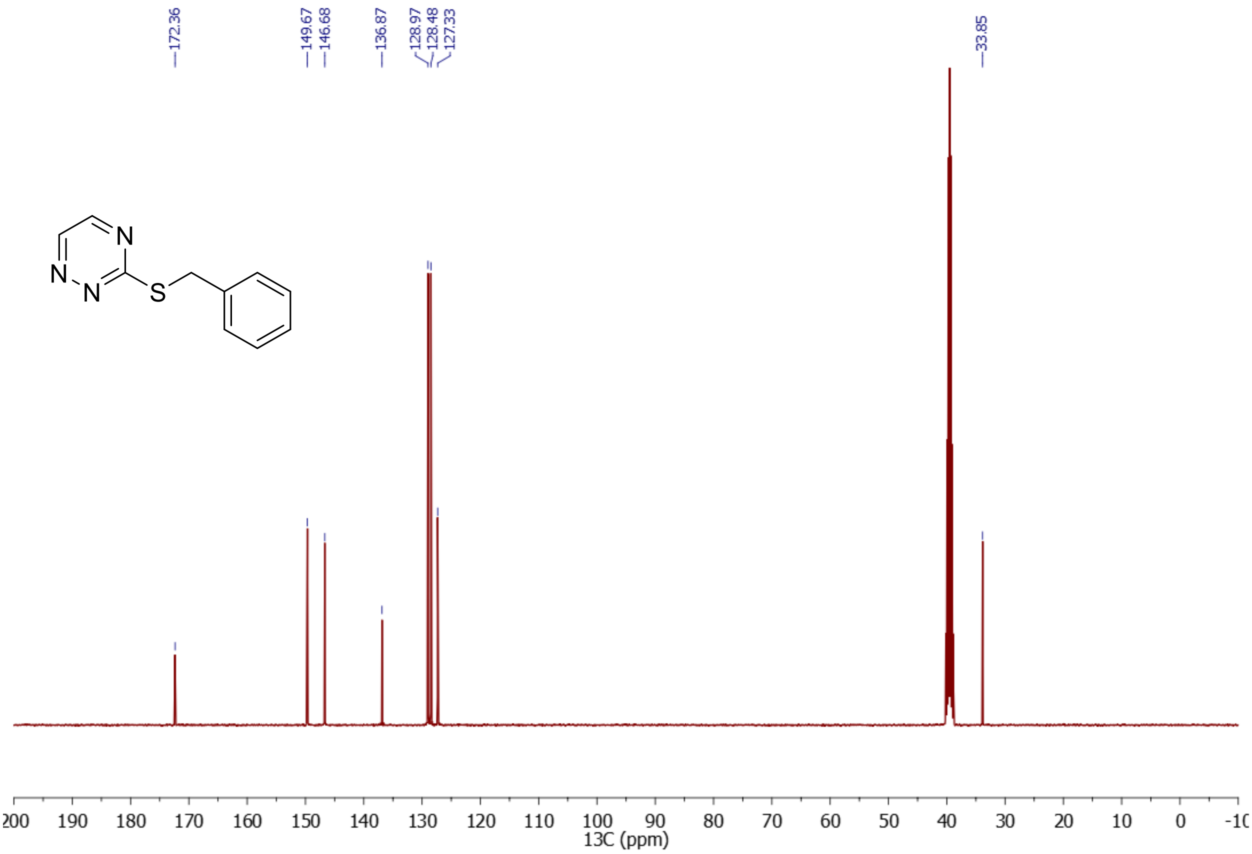

^{13}C NMR spectrum of 3-benzylthio-1,2,4-triazine **1f**

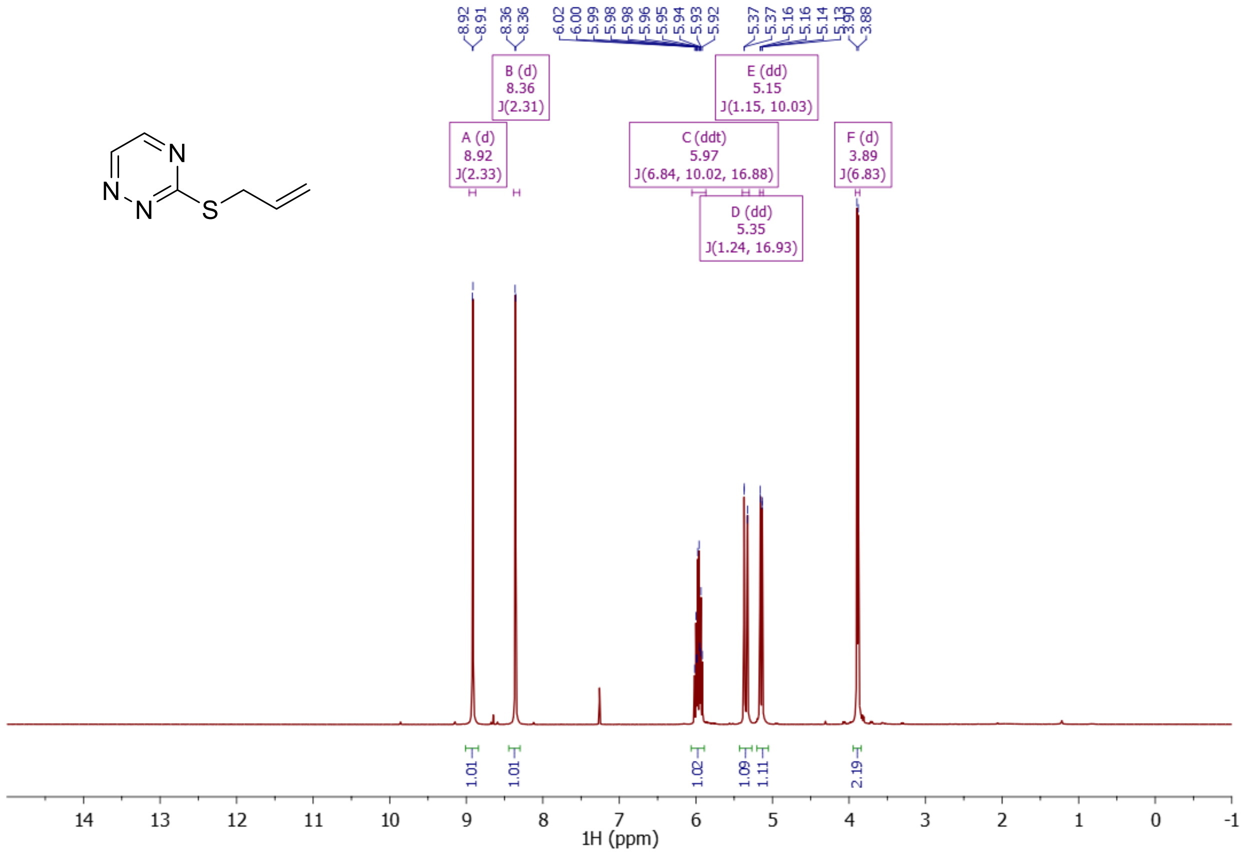

^{1}H NMR spectrum of 3-allylthio-1,2,4-triazine **1g**

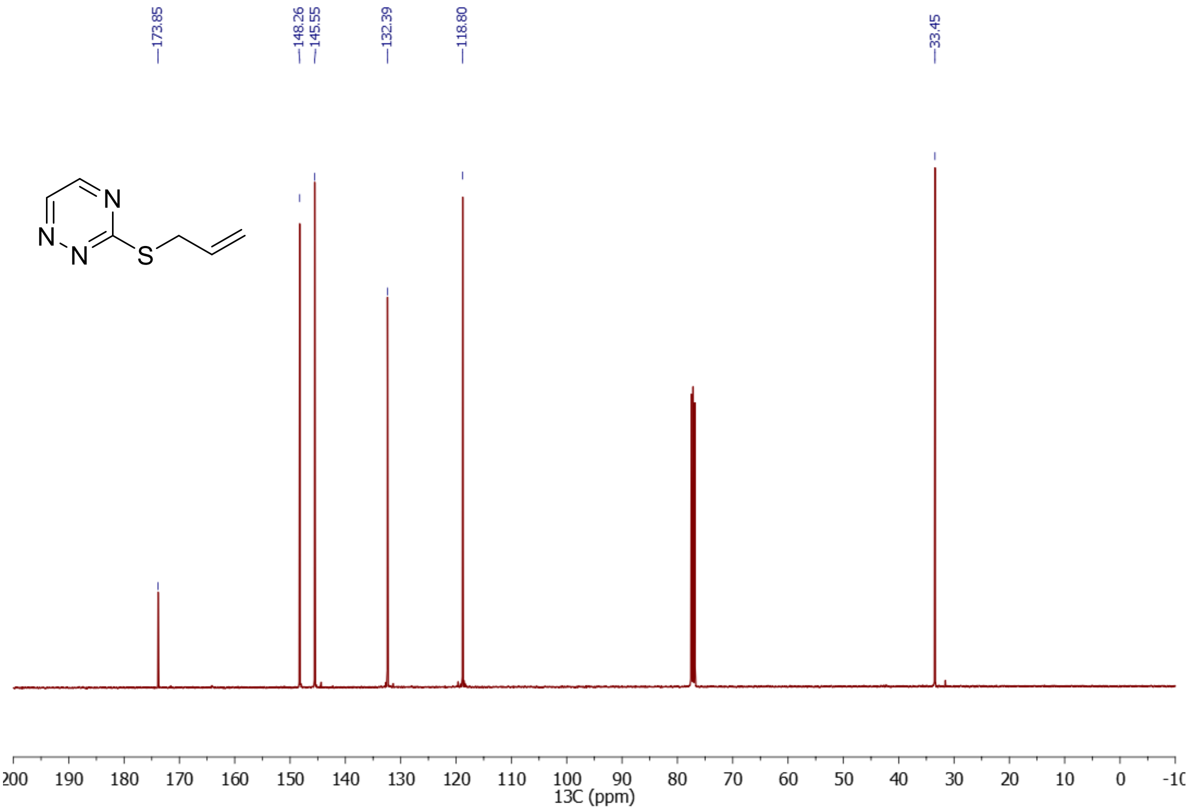

^{13}C NMR spectrum of 3-allylthio-1,2,4-triazine **1g**

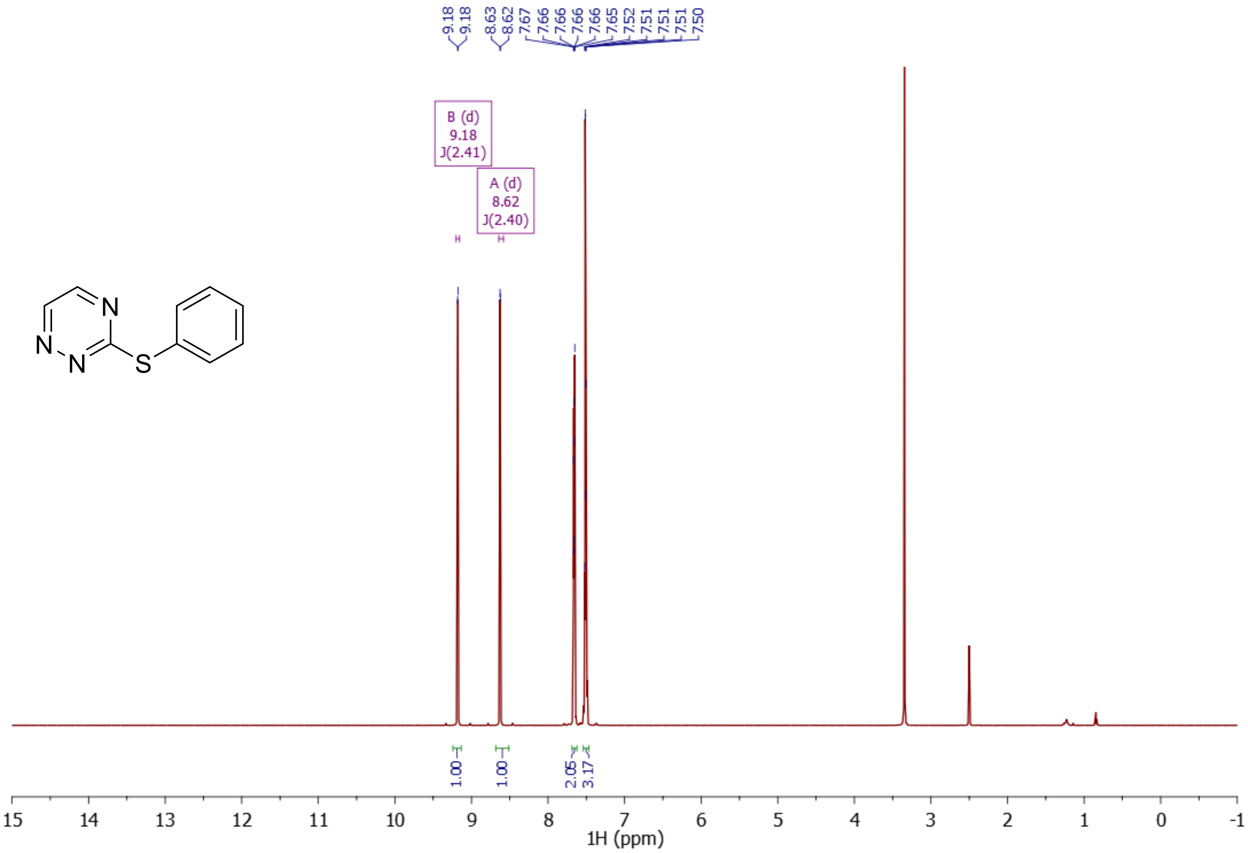

^{1}H NMR spectrum of 3-phenylthio-1,2,4-triazine **1h**

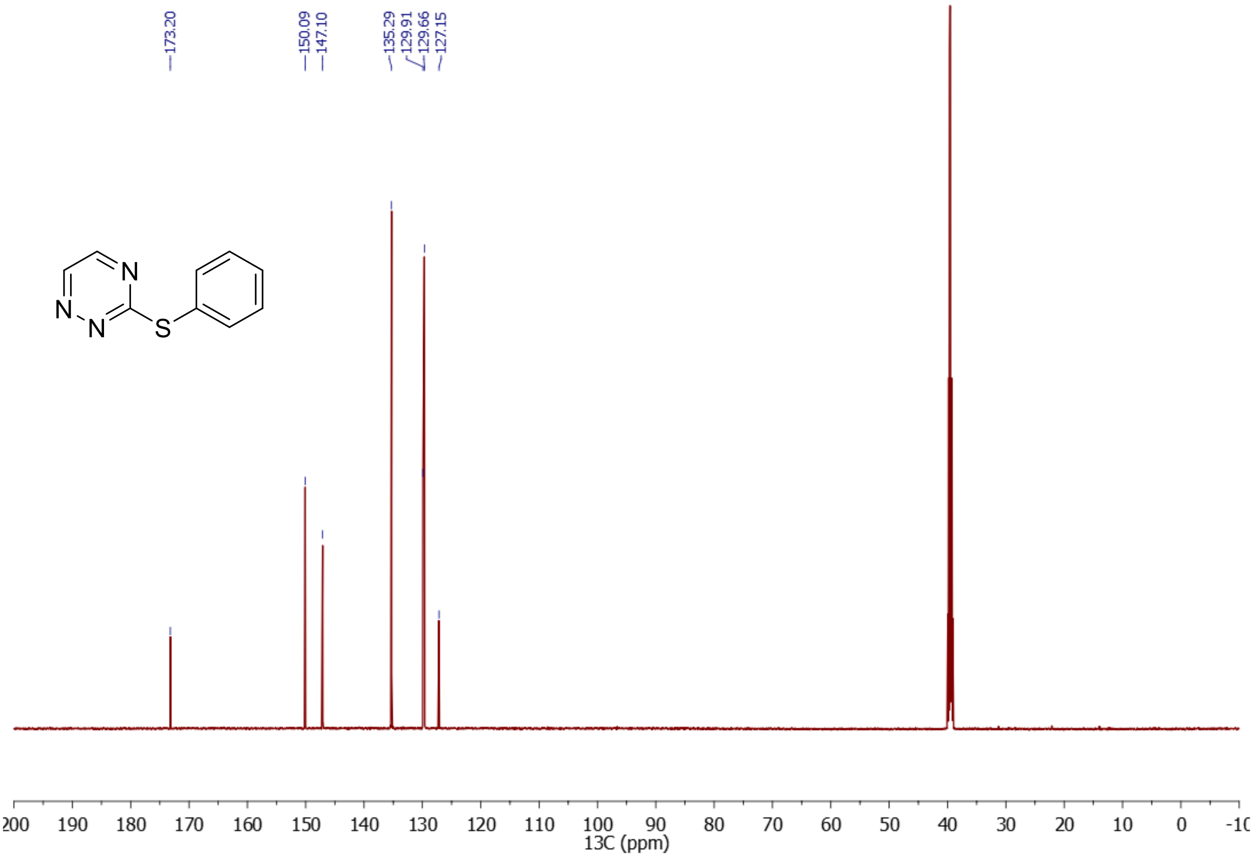

^{13}C NMR spectrum of 3-phenylthio-1,2,4-triazine **1h**

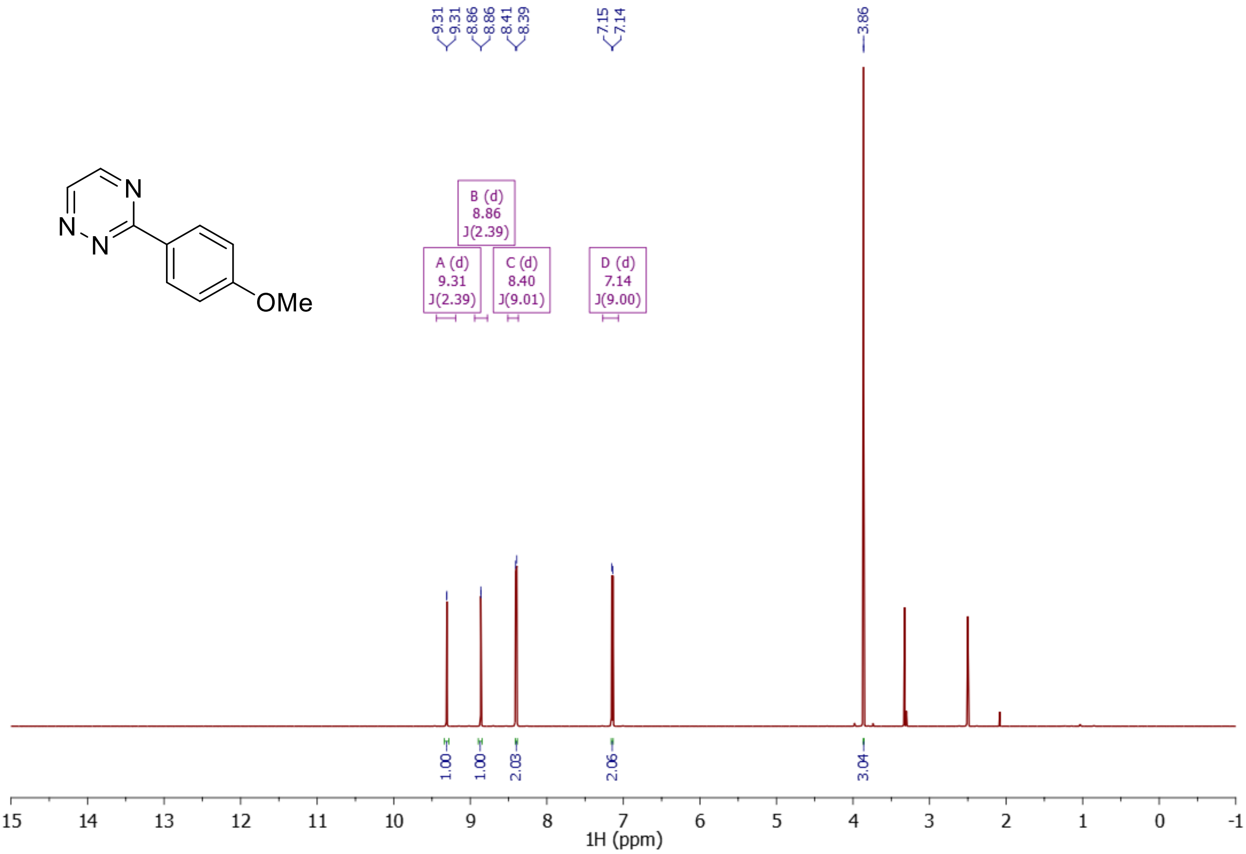

^{1}H NMR spectrum of 3-(4-methoxyphenyl)-1,2,4-triazine **1j**

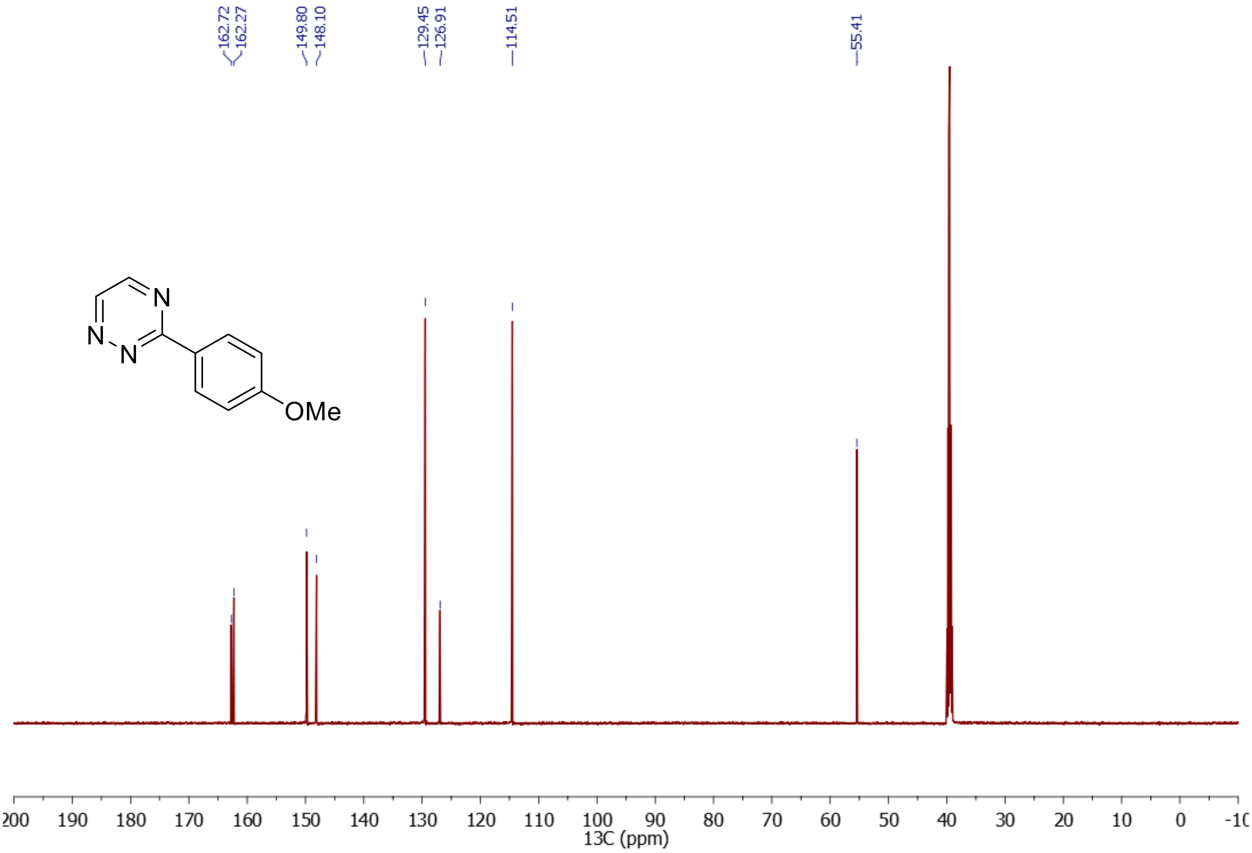

^{13}C NMR spectrum of 3-(4-methoxyphenyl)-1,2,4-triazine **1j**

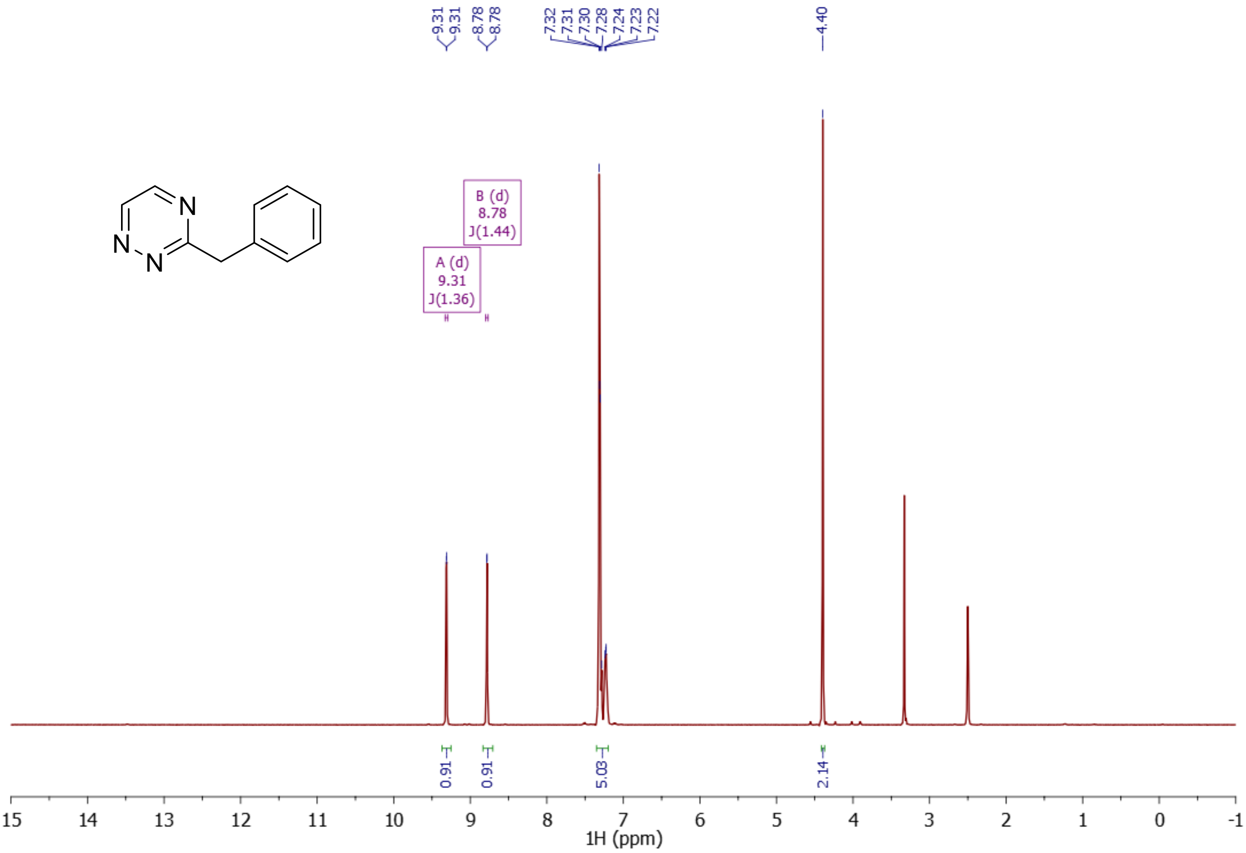

^{1}H NMR spectrum of 3-benzyl-1,2,4-triazine **1l**

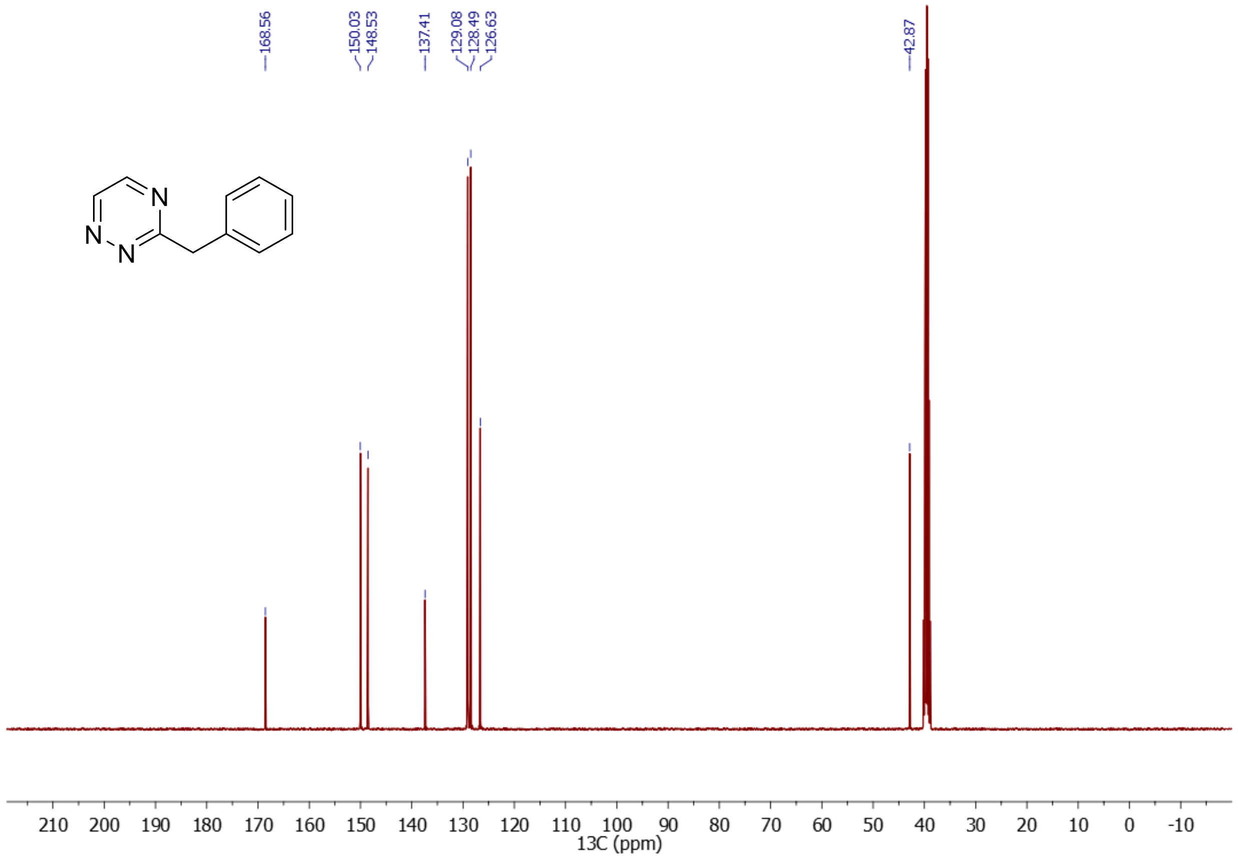

^{13}C NMR spectrum of 3-benzyl-1,2,4-triazine **1l**

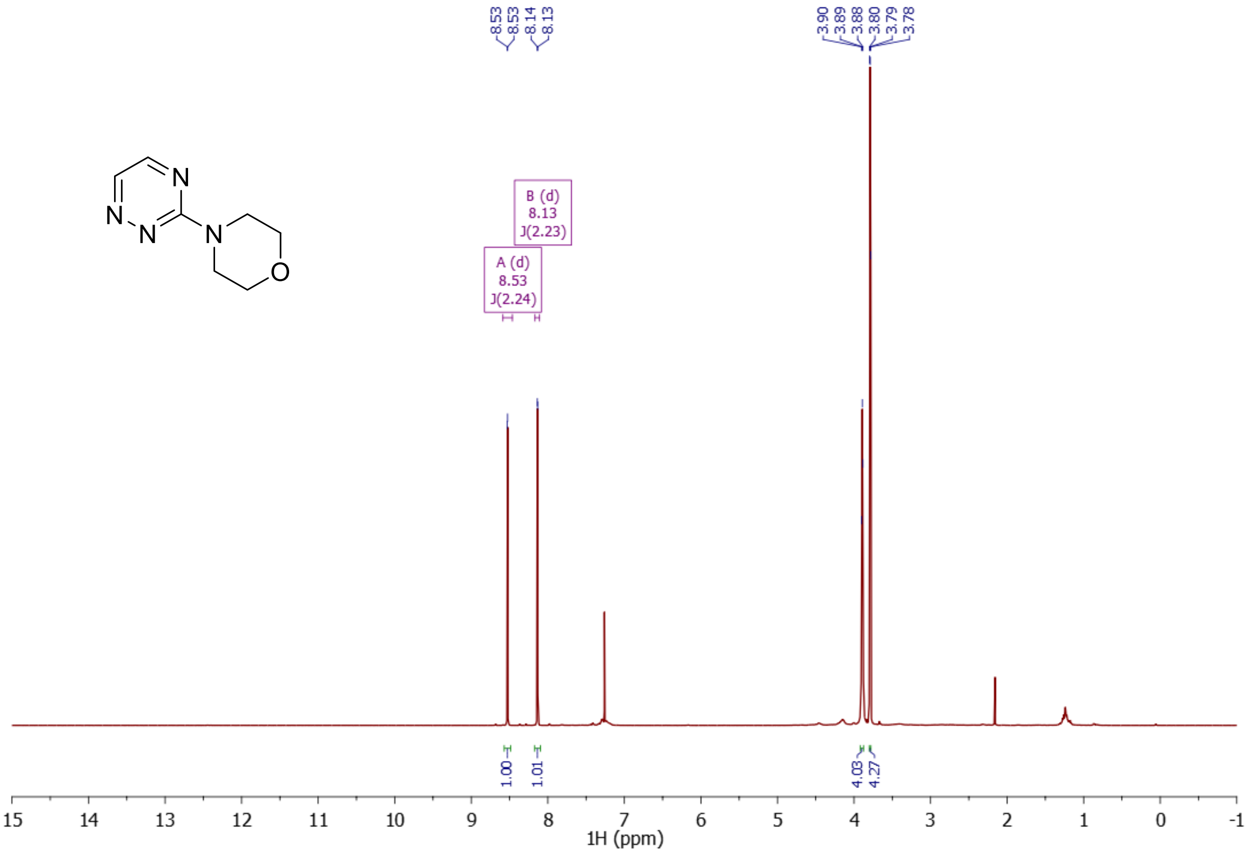

^{1}H NMR spectrum of 4-(1,2,4-triazin-3-yl)morpholine **1m**

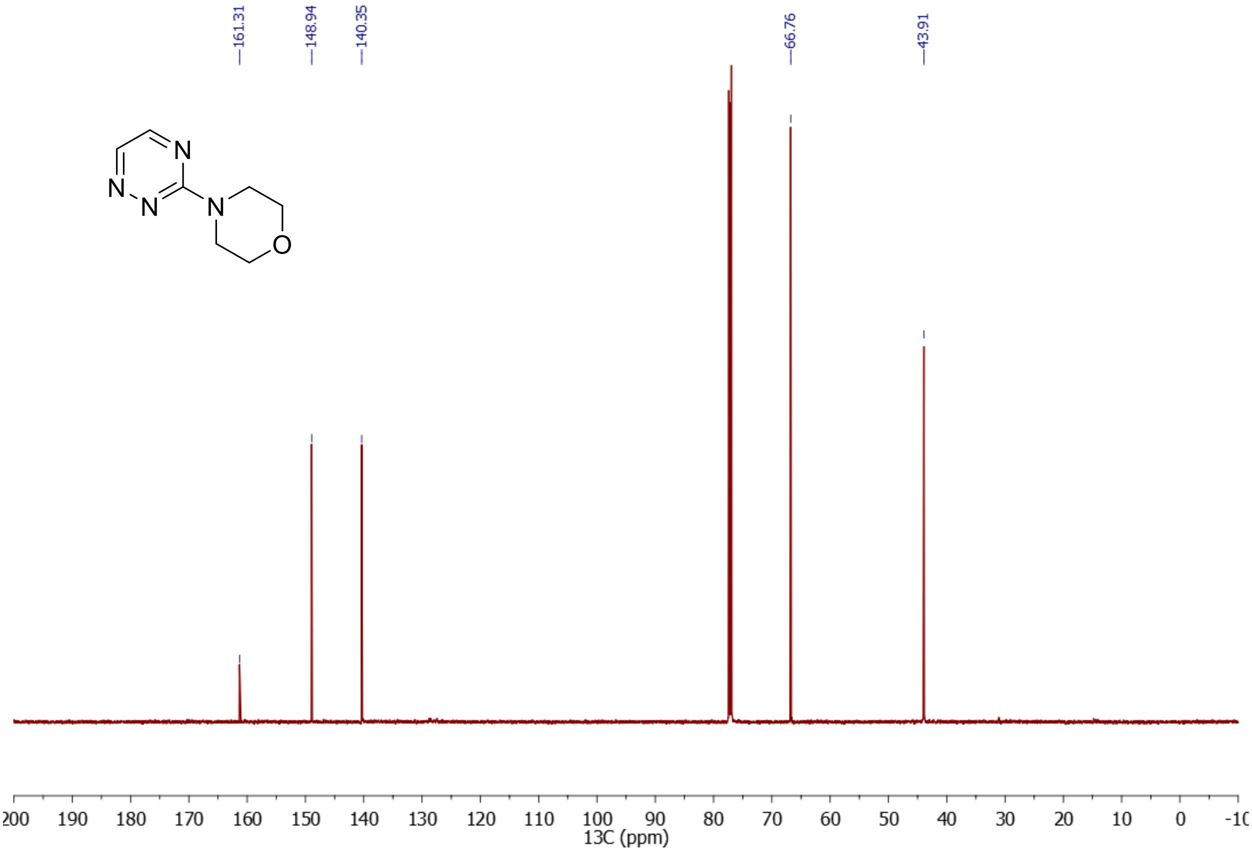

^{13}C NMR spectrum of 4-(1,2,4-triazin-3-yl)morpholine **1m**

Copies of ^{1}H and ^{13}C NMR spectra for compounds 3

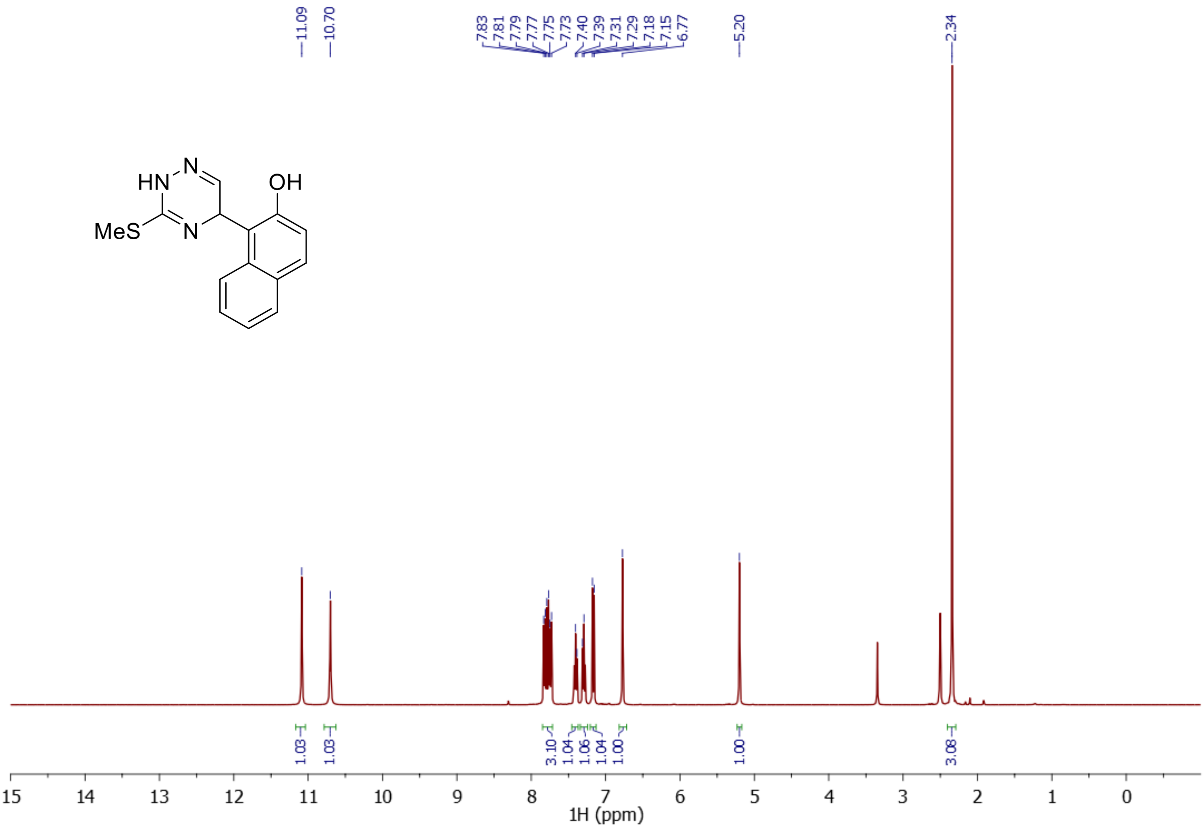

^{1}H NMR spectrum of 1-(3-(methylthio)-2,5-dihydro-1,2,4-triazin-5-yl)naphthalen-2-ol **3aa**

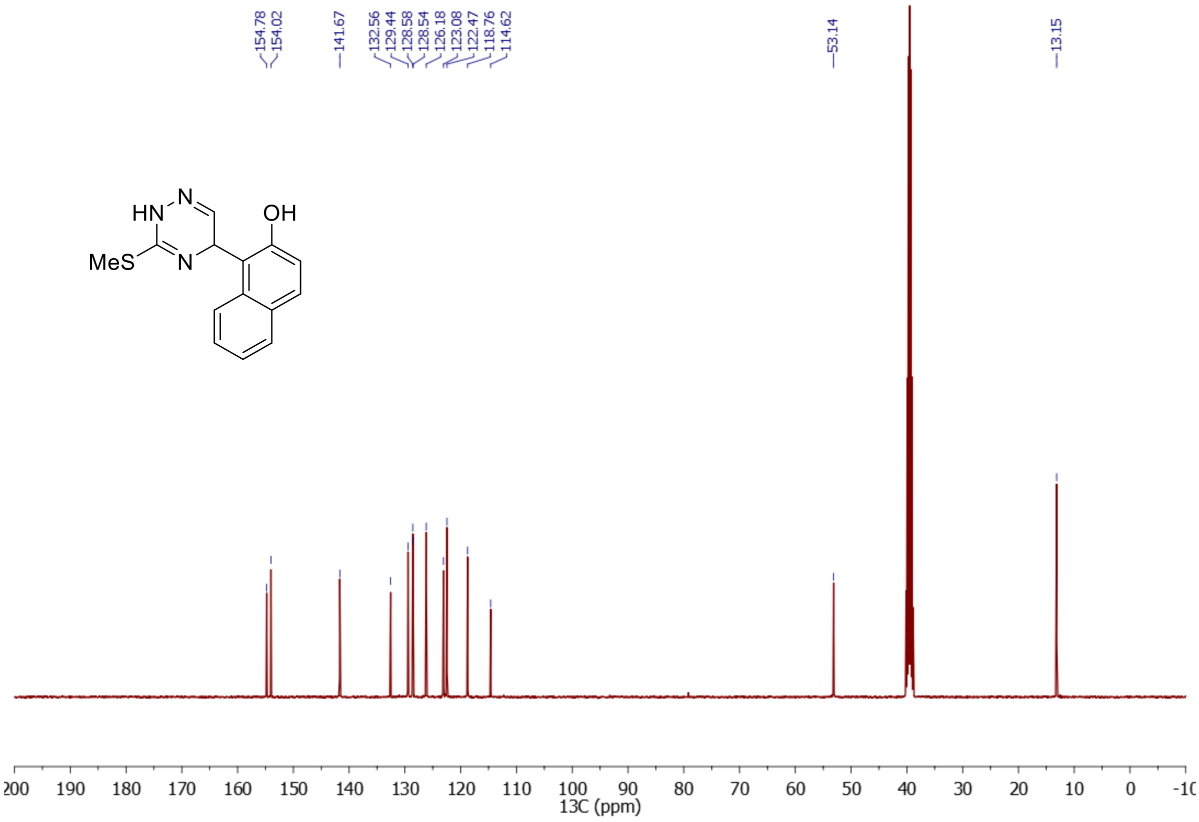

^{13}C NMR spectrum of 1-(3-(methylthio)-2,5-dihydro-1,2,4-triazin-5-yl)naphthalen-2-ol **3aa**

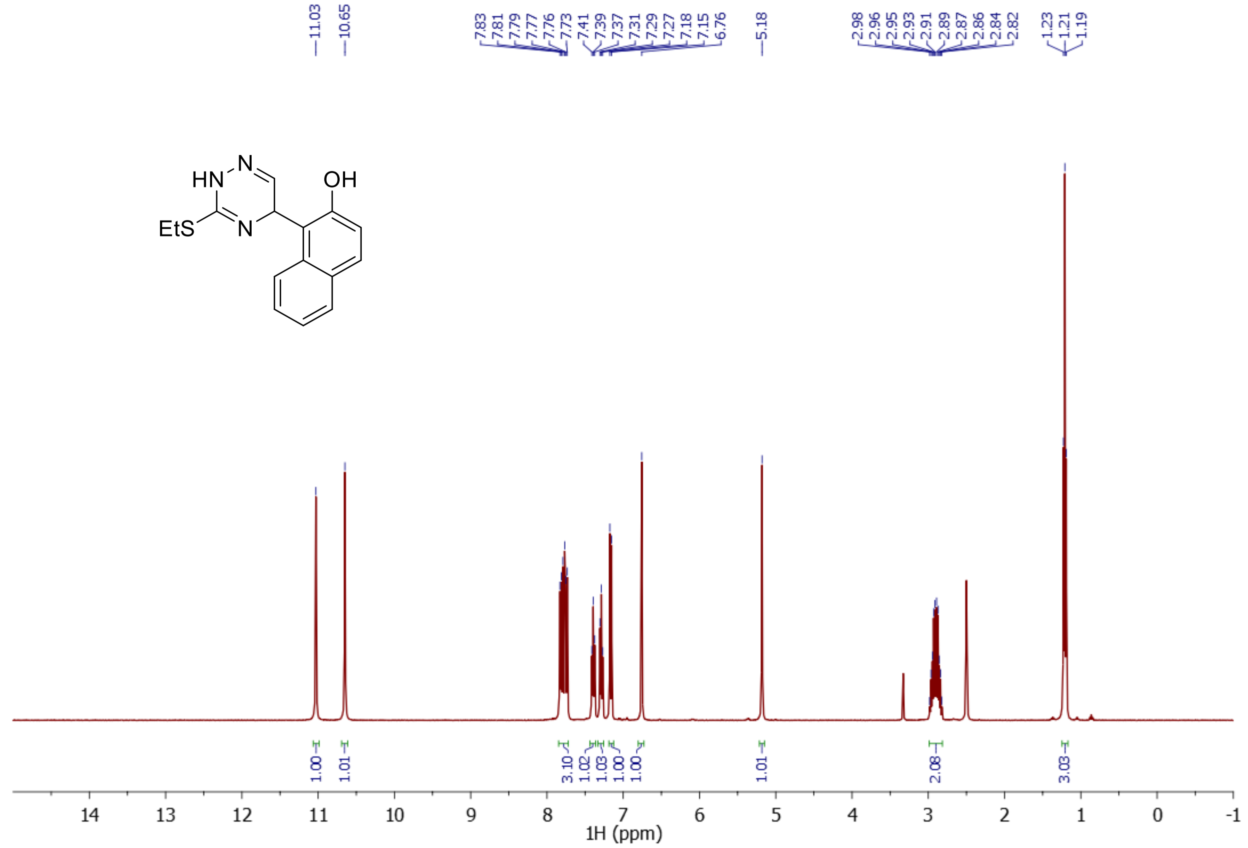

^{1}H NMR spectrum of 1-(3-(ethylthio)-2,5-dihydro-1,2,4-triazin-5-yl)naphthalen-2-ol **3ba**

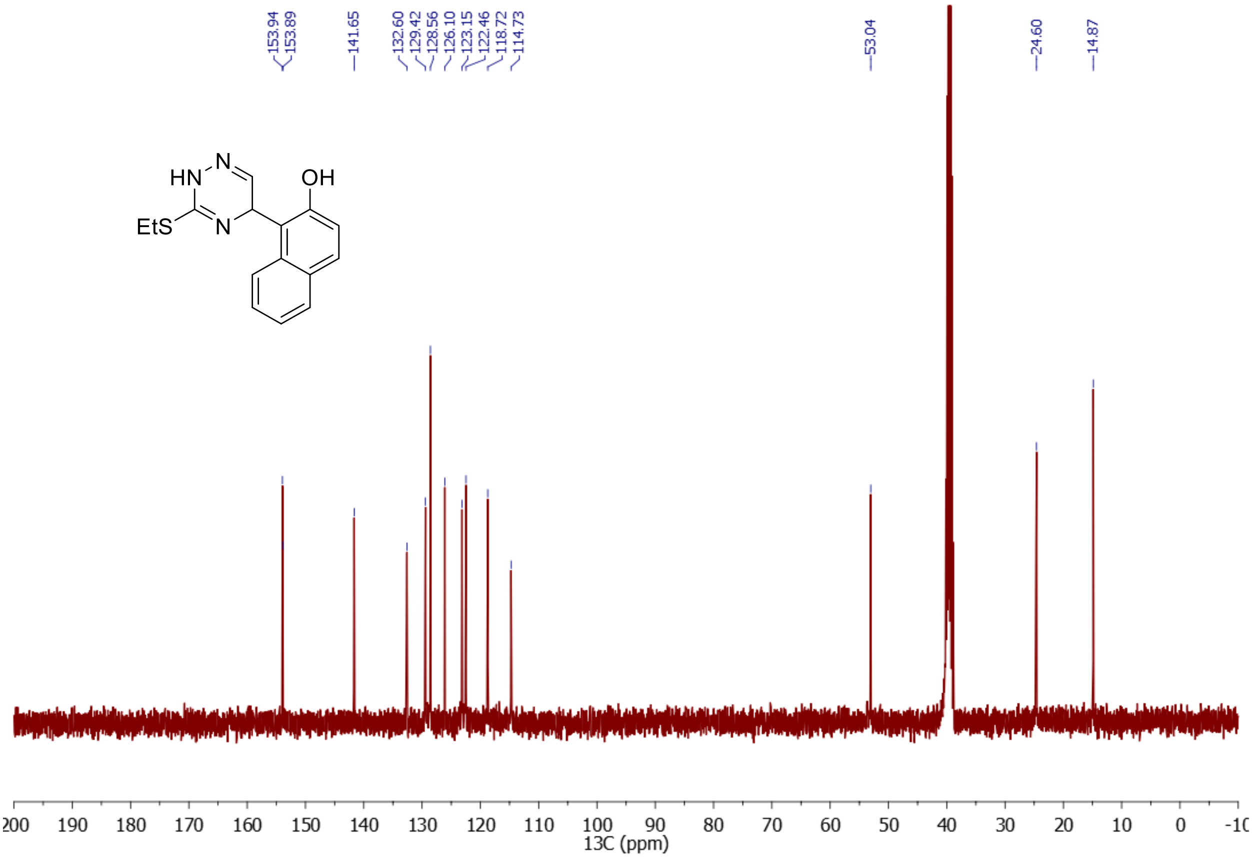

^{13}C NMR spectrum of 1-(3-(ethylthio)-2,5-dihydro-1,2,4-triazin-5-yl)naphthalen-2-ol **3ba**

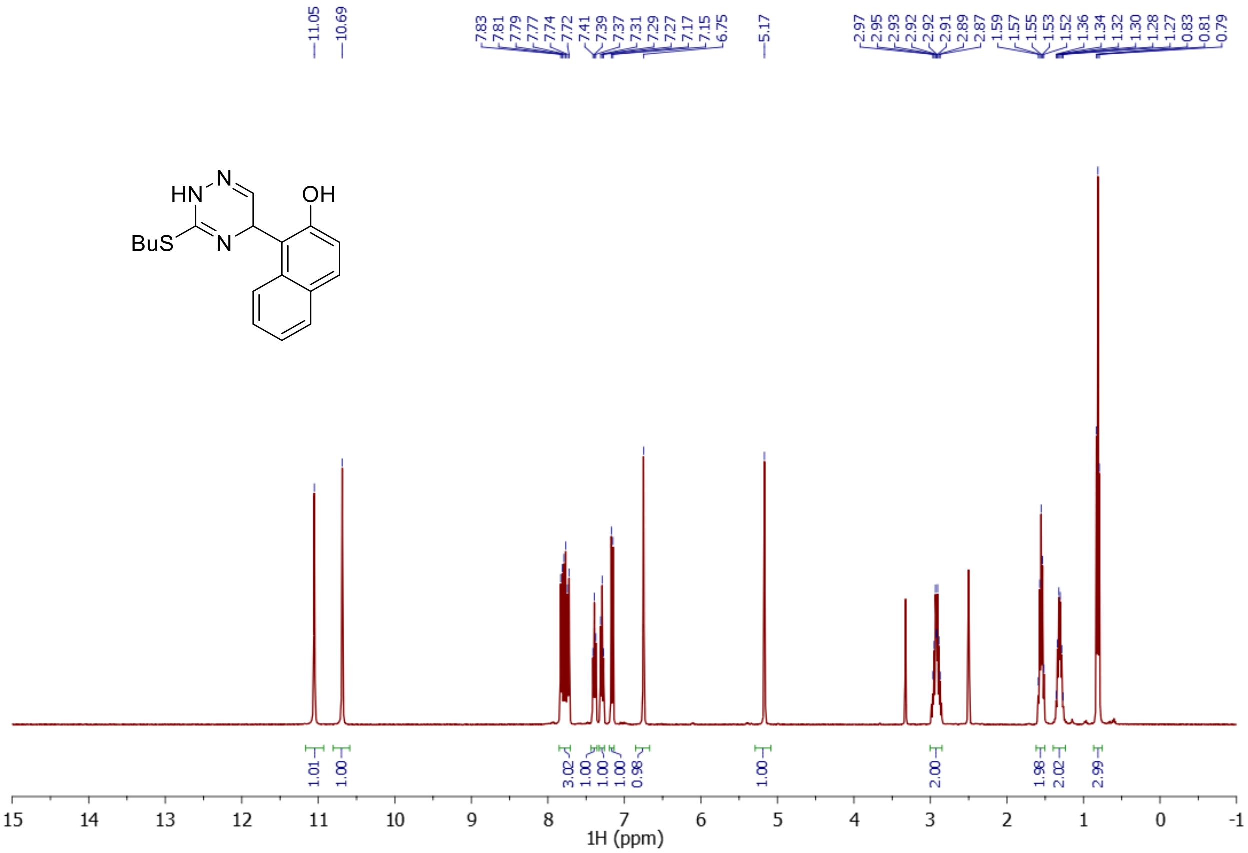

^{1}H NMR spectrum of 1-(3-(butylthio)-2,5-dihydro-1,2,4-triazin-5-yl)naphthalen-2-ol **3ca**

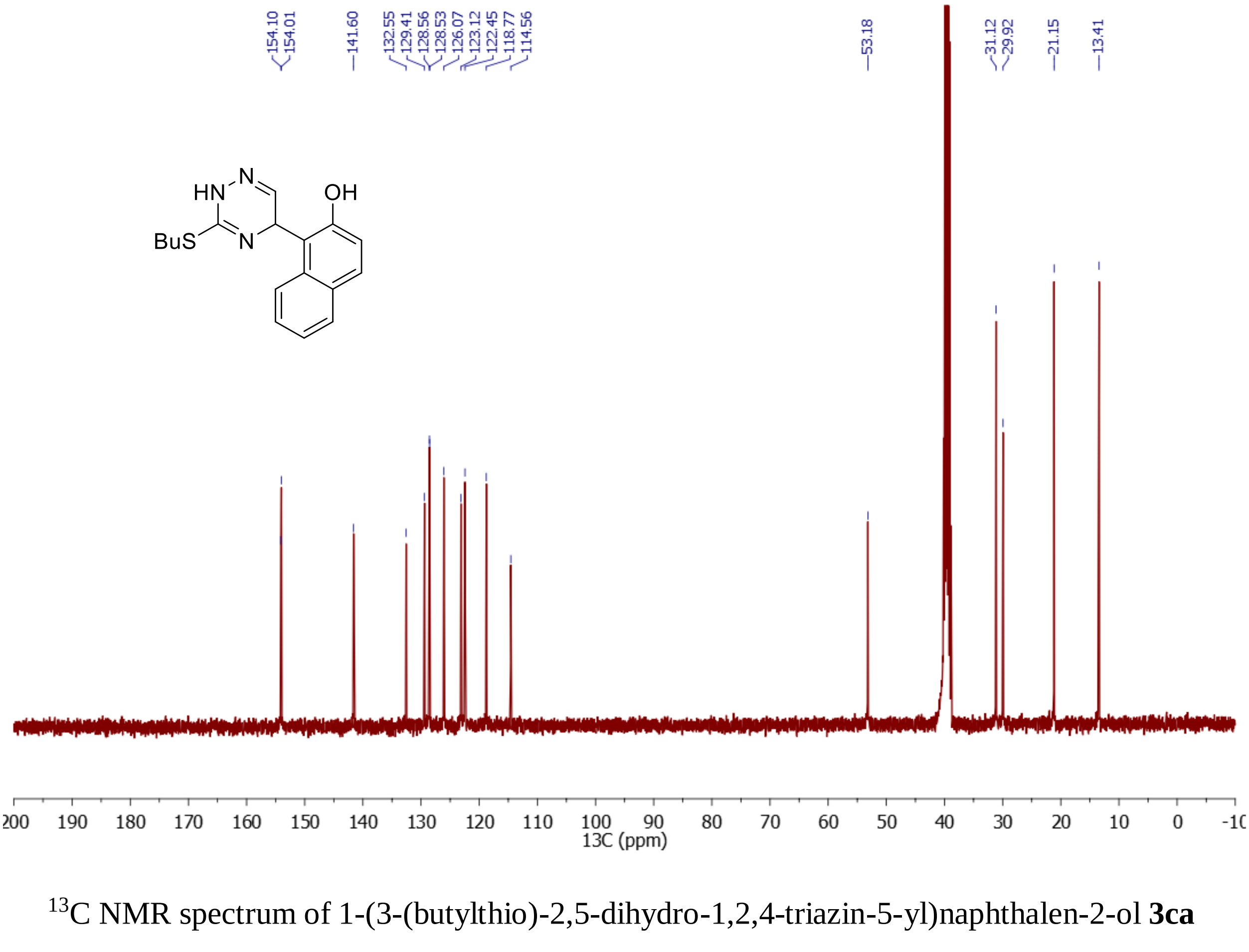

^{13}C NMR spectrum of 1-(3-(butylthio)-2,5-dihydro-1,2,4-triazin-5-yl)naphthalen-2-ol **3ca**

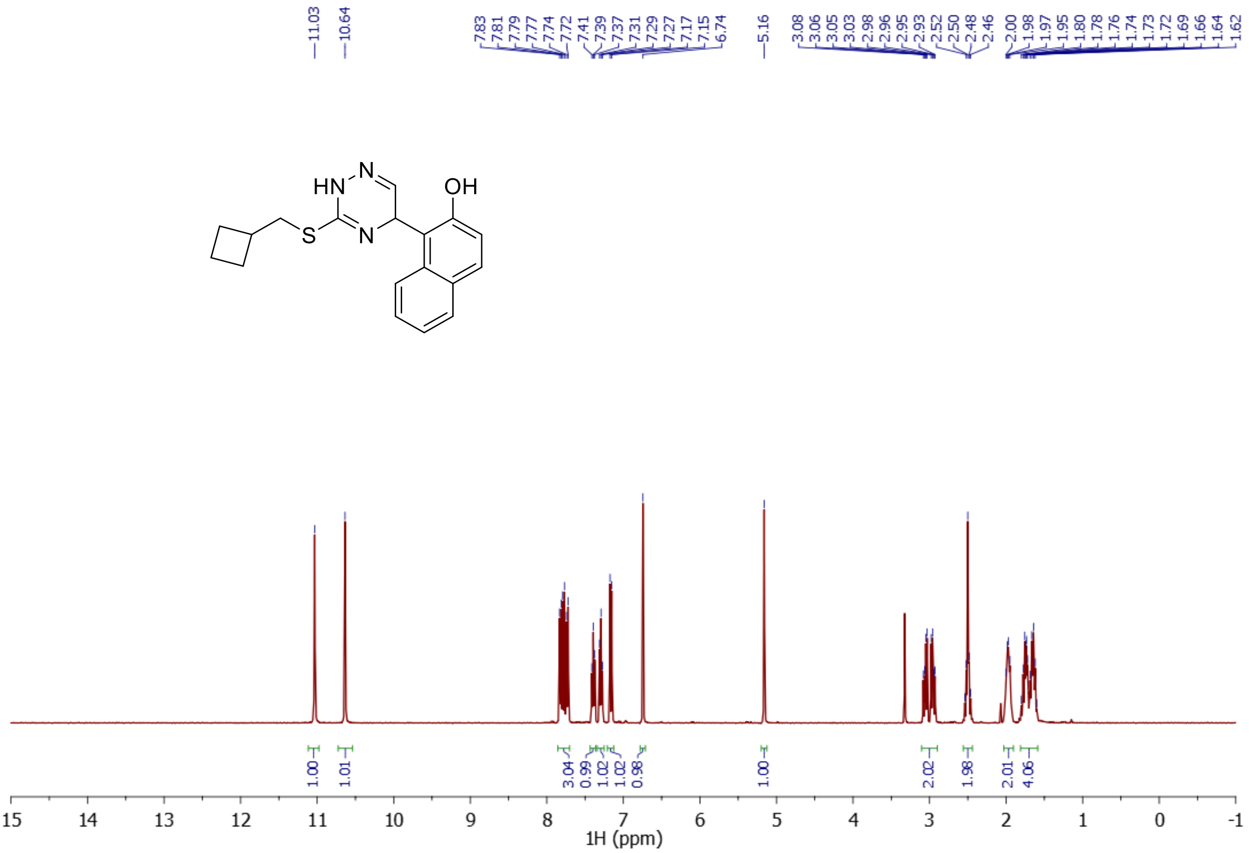

^{1}H NMR spectrum of 1-(3-((cyclobutylmethyl)thio)-2,5-dihydro-1,2,4-triazin-5-yl)naphthalen-2-ol **3da**

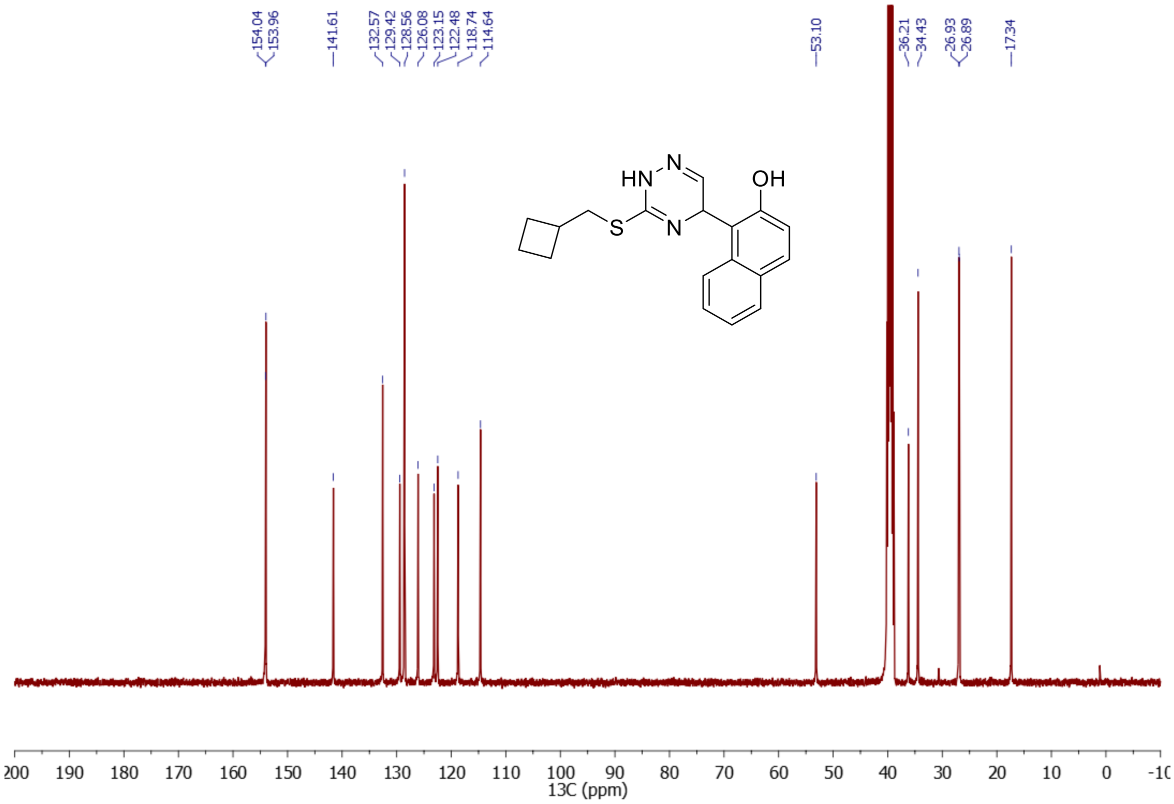

^{13}C NMR spectrum of 1-(3-((cyclobutylmethyl)thio)-2,5-dihydro-1,2,4-triazin-5-yl)naphthalen-2-ol **3da**

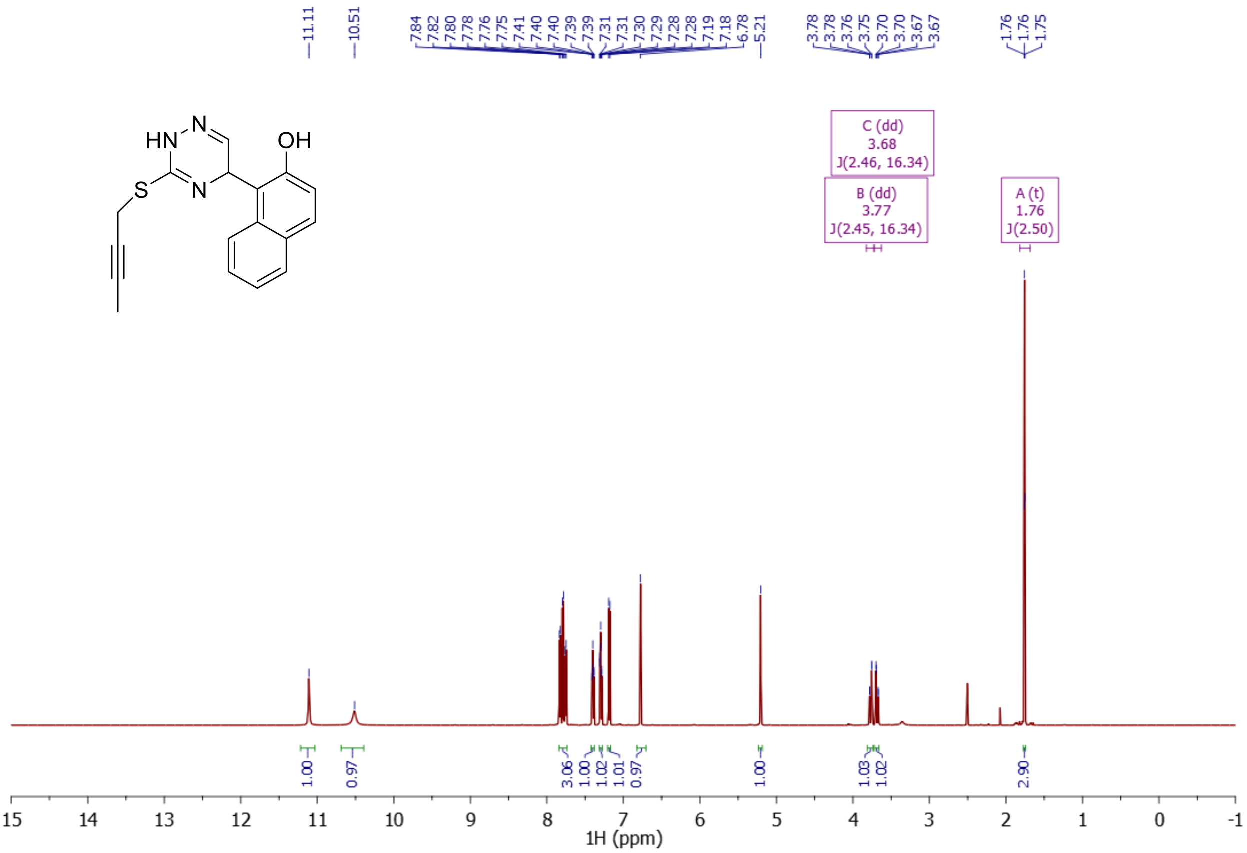

^{1}H NMR spectrum of 1-(3-(but-2-yn-1-ylthio)-2,5-dihydro-1,2,4-triazin-5-yl)naphthalen-2-ol **3ea**

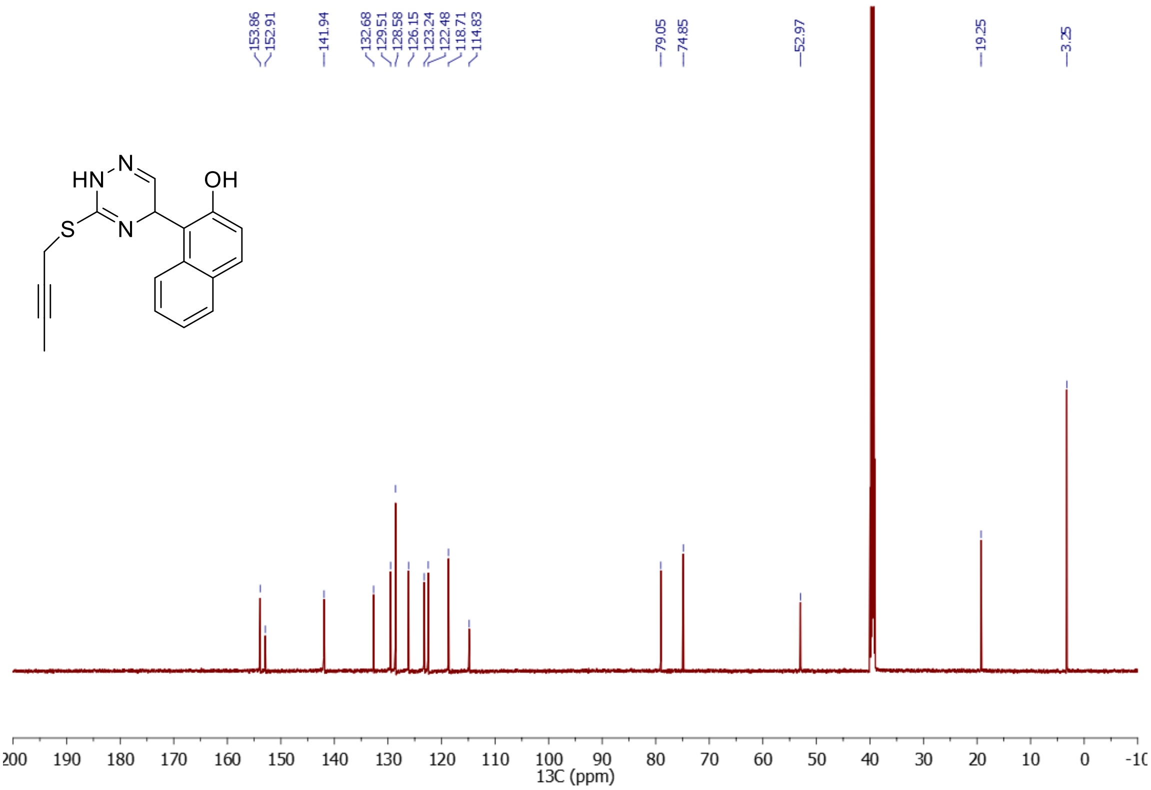

^{13}C NMR spectrum of 1-(3-(but-2-yn-1-ylthio)-2,5-dihydro-1,2,4-triazin-5-yl)naphthalen-2-ol **3ea**

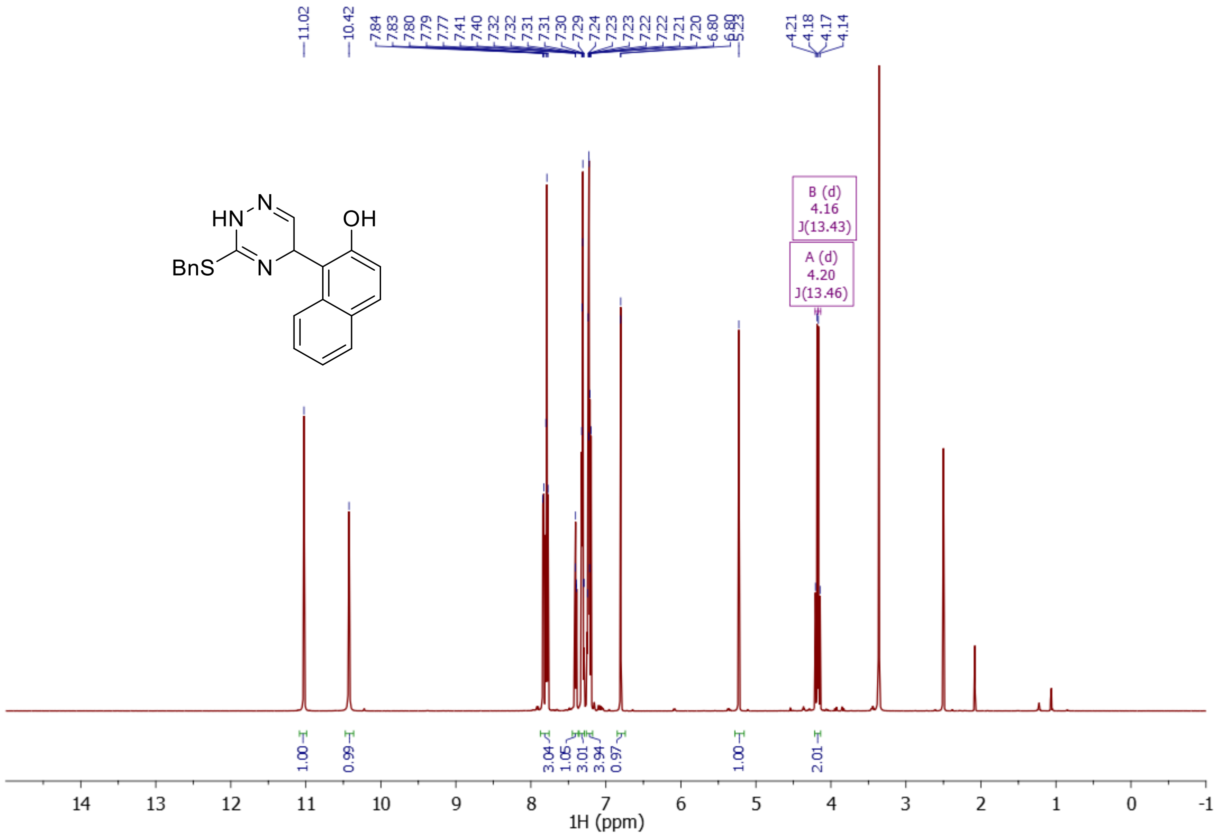

^{1}H NMR spectrum of 1-(3-(benzylthio)-2,5-dihydro-1,2,4-triazin-5-yl)naphthalen-2-ol **3fa**

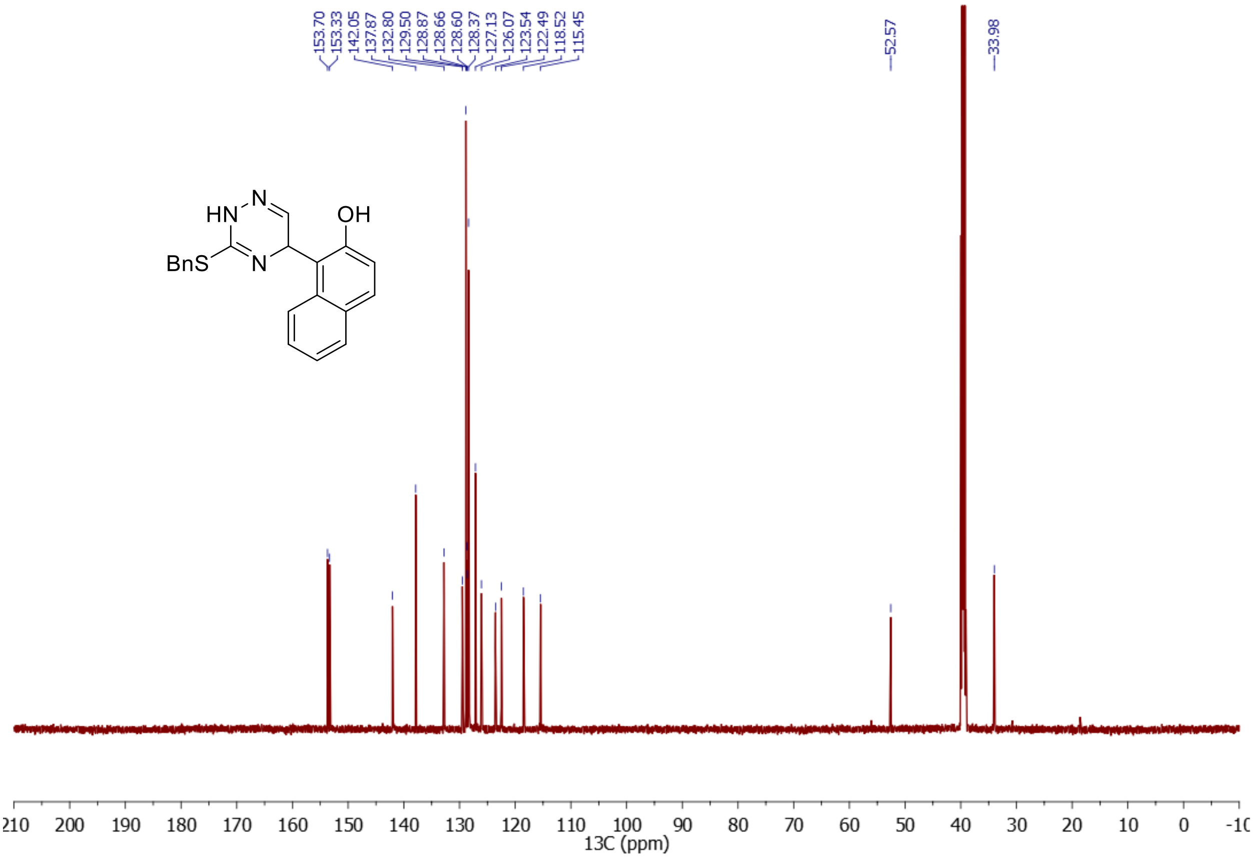

^{13}C NMR spectrum of 1-(3-(benzylthio)-2,5-dihydro-1,2,4-triazin-5-yl)naphthalen-2-ol **3fa**

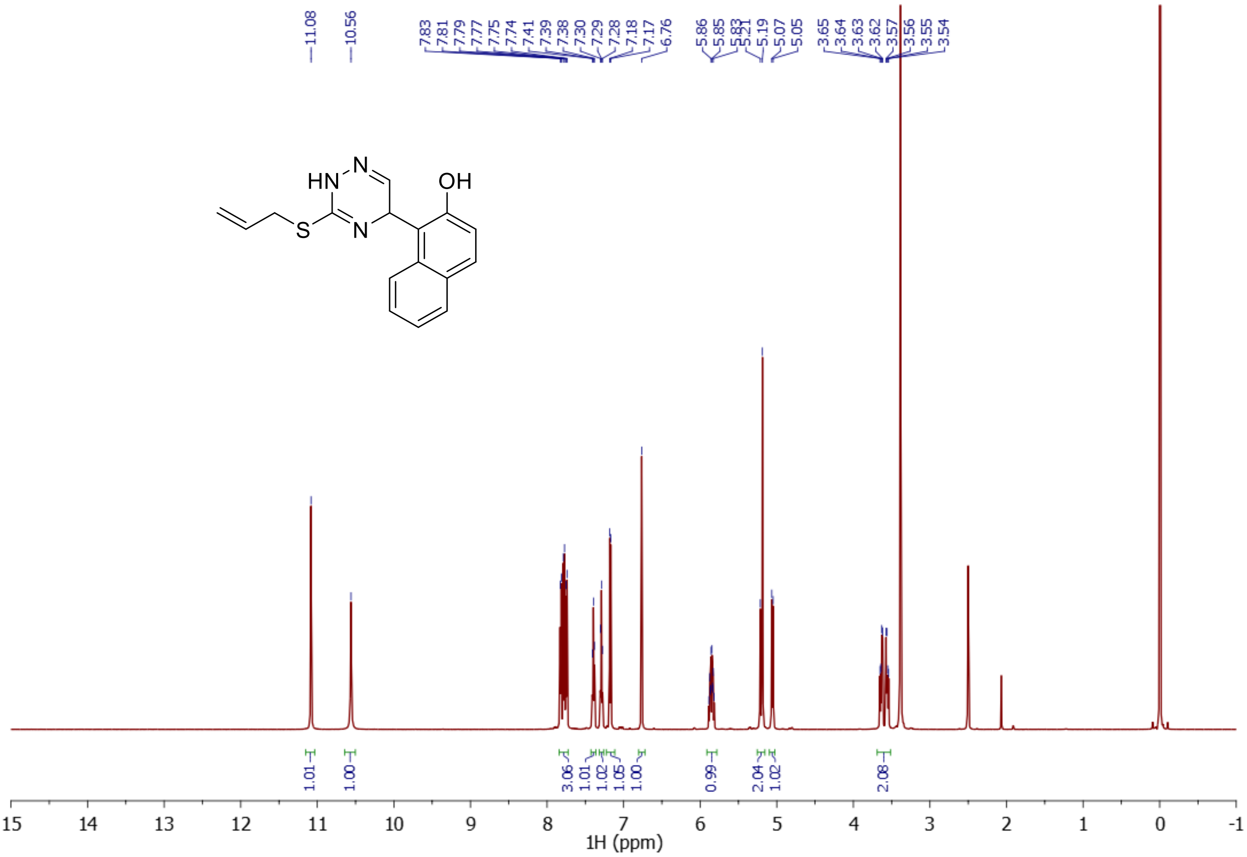

^{1}H NMR spectrum of 1-(3-(allylthio)-2,5-dihydro-1,2,4-triazin-5-yl)naphthalen-2-ol **3ga**

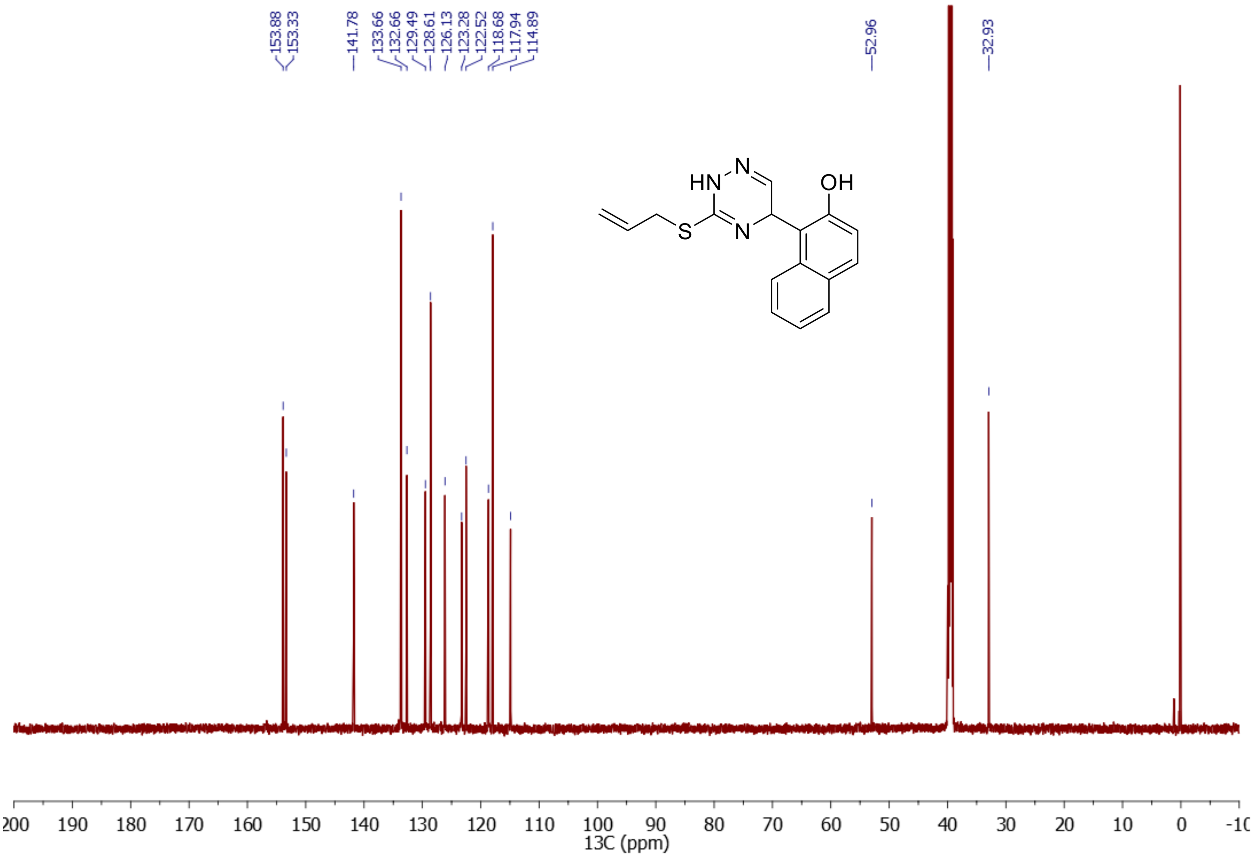

^{13}C NMR spectrum of 1-(3-(allylthio)-2,5-dihydro-1,2,4-triazin-5-yl)naphthalen-2-ol **3ga**

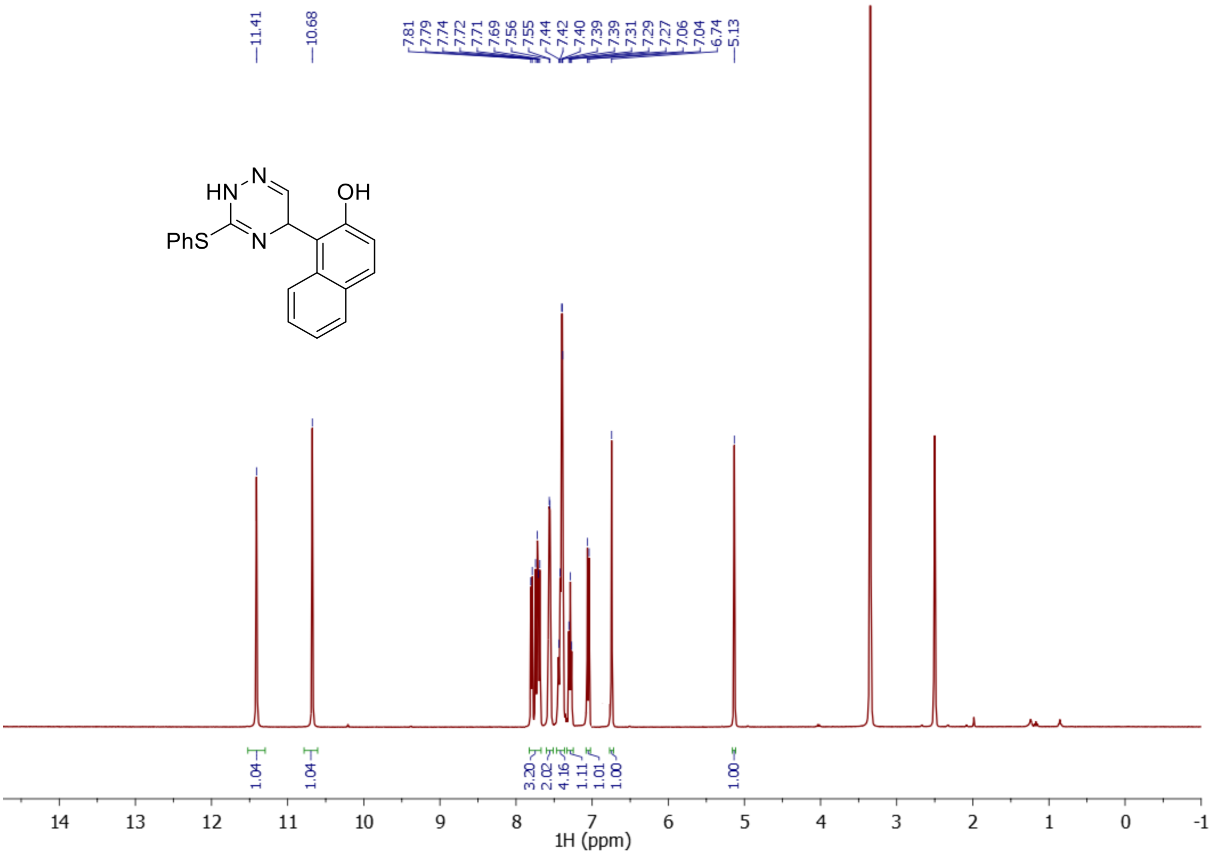

^{1}H NMR spectrum of 1-(3-phenylthio-2,5-dihydro-1,2,4-triazin-5-yl)naphthalen-2-ol **3ha**

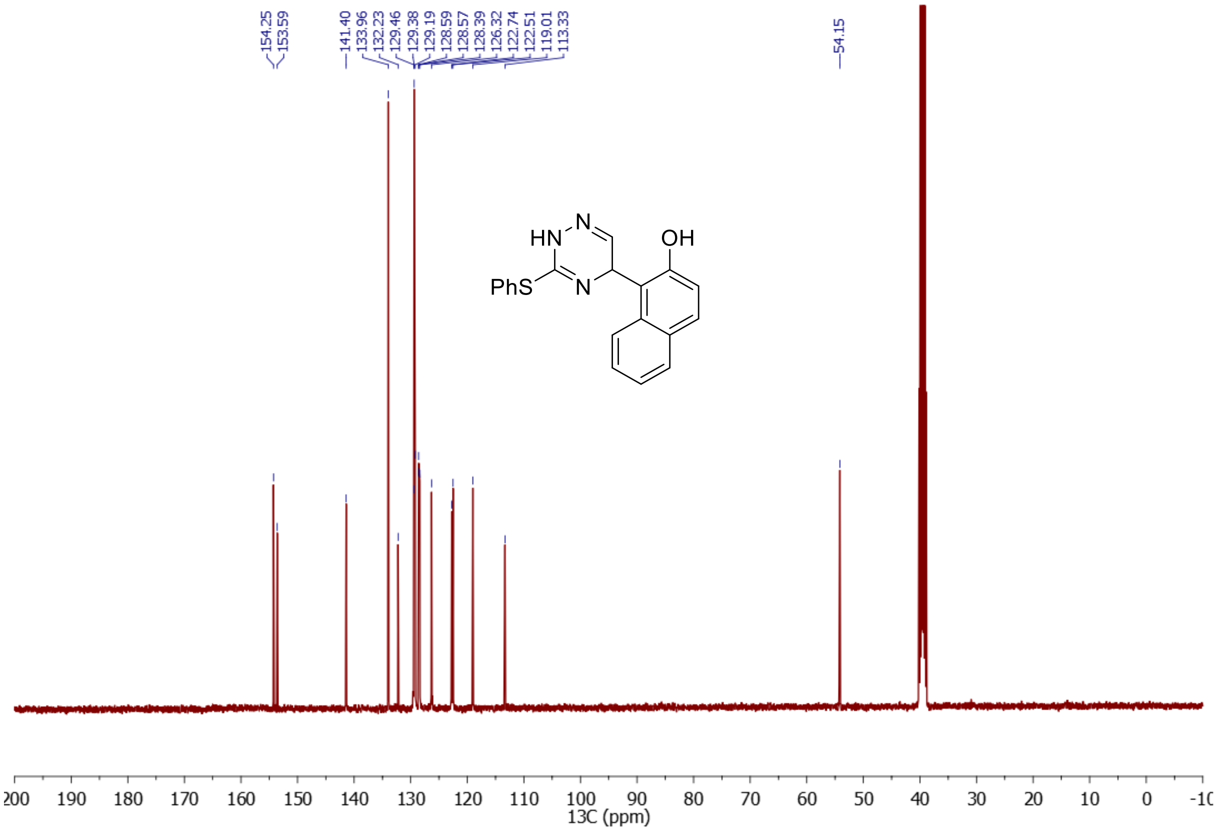

^{13}C NMR spectrum of 1-(3-phenylthio-2,5-dihydro-1,2,4-triazin-5-yl)naphthalen-2-ol **3ha**

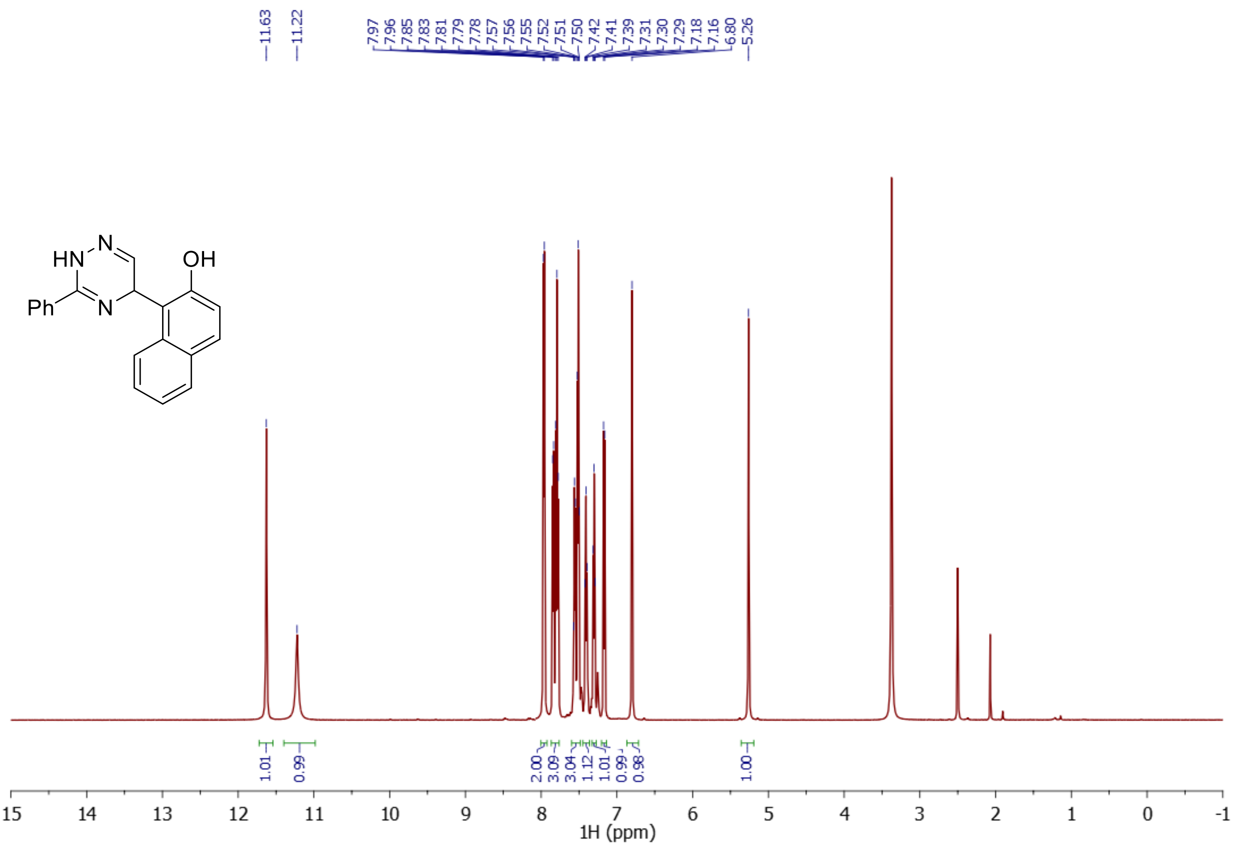

^{1}H NMR spectrum of 1-(3-phenyl-2,5-dihydro-1,2,4-triazin-5-yl)naphthalen-2-ol **3ia**

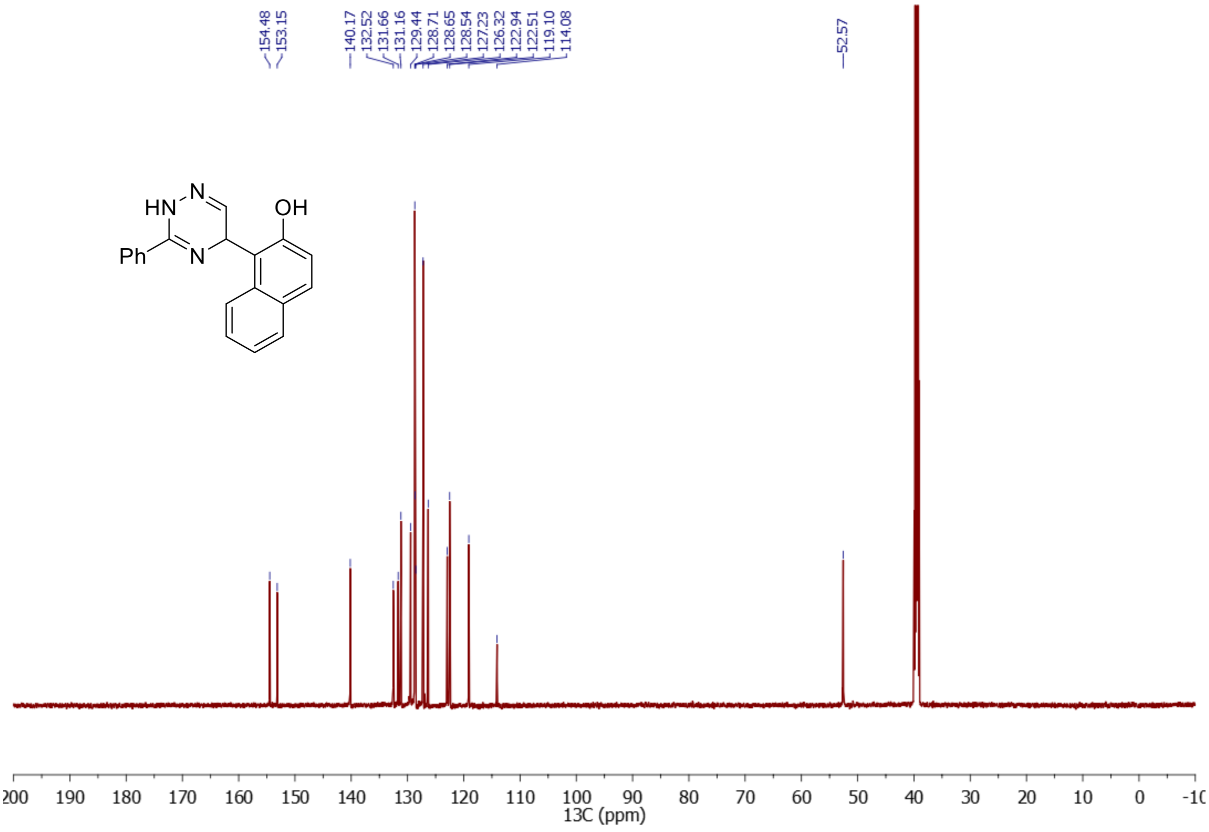

^{13}C NMR spectrum of 1-(3-phenyl-2,5-dihydro-1,2,4-triazin-5-yl)naphthalen-2-ol **3ia**

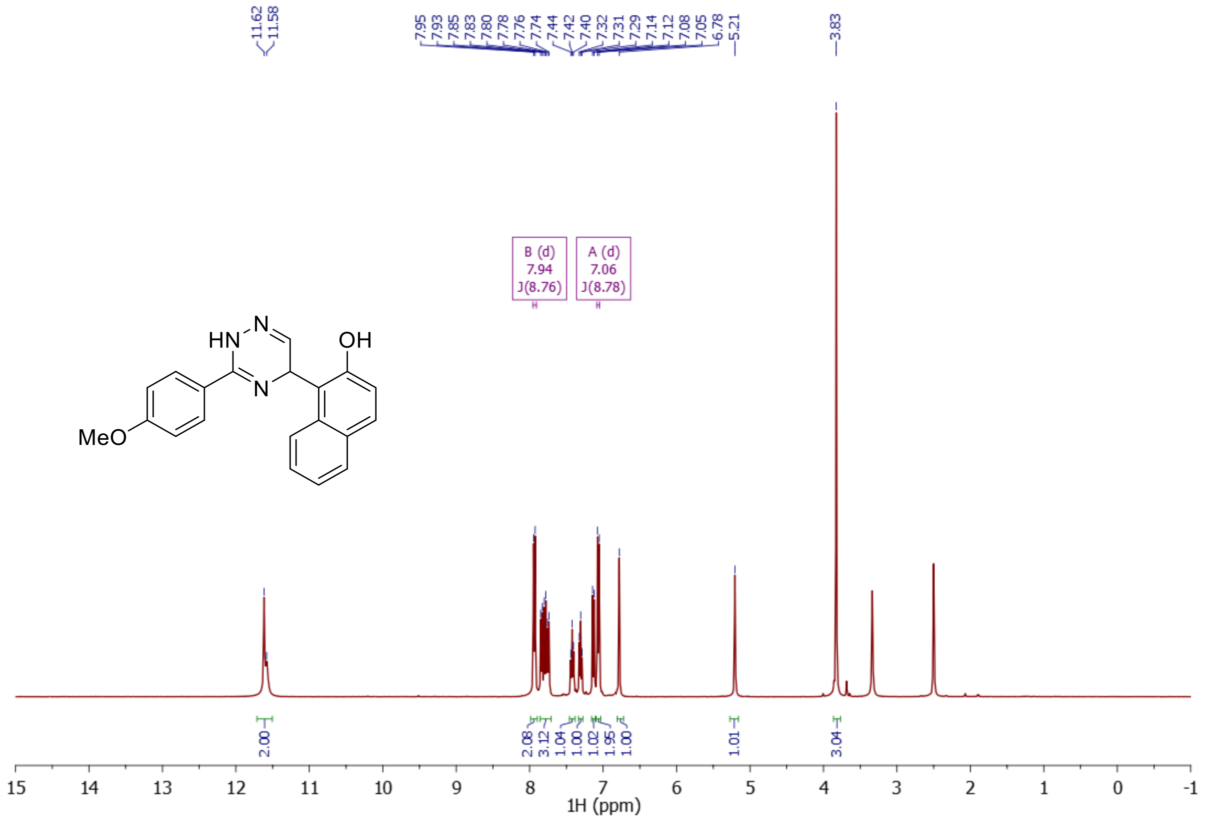

^{1}H NMR spectrum of 1-(3-(4-methoxyphenyl)-2,5-dihydro-1,2,4-triazin-5-yl)naphthalen-2-ol **3ja**

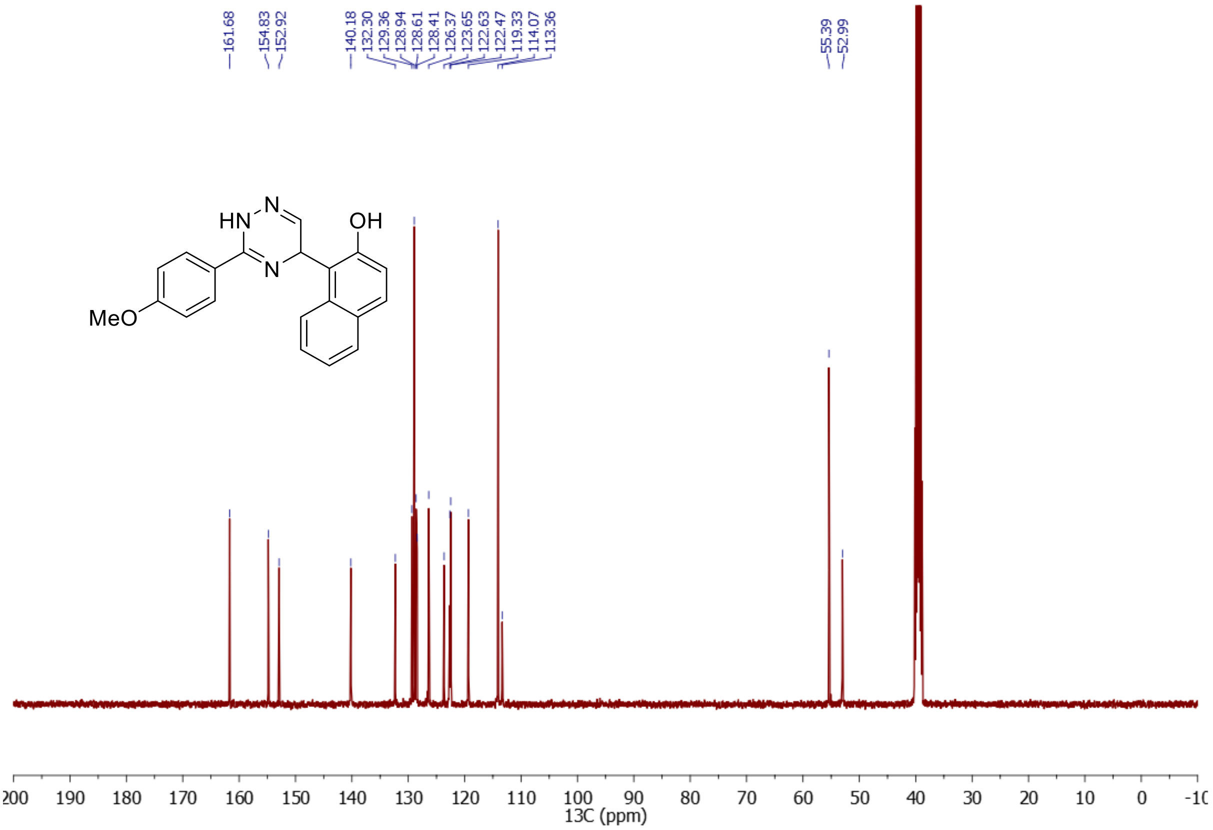

^{13}C NMR spectrum of 1-(3-(4-methoxyphenyl)-2,5-dihydro-1,2,4-triazin-5-yl)naphthalen-2-ol **3ja**

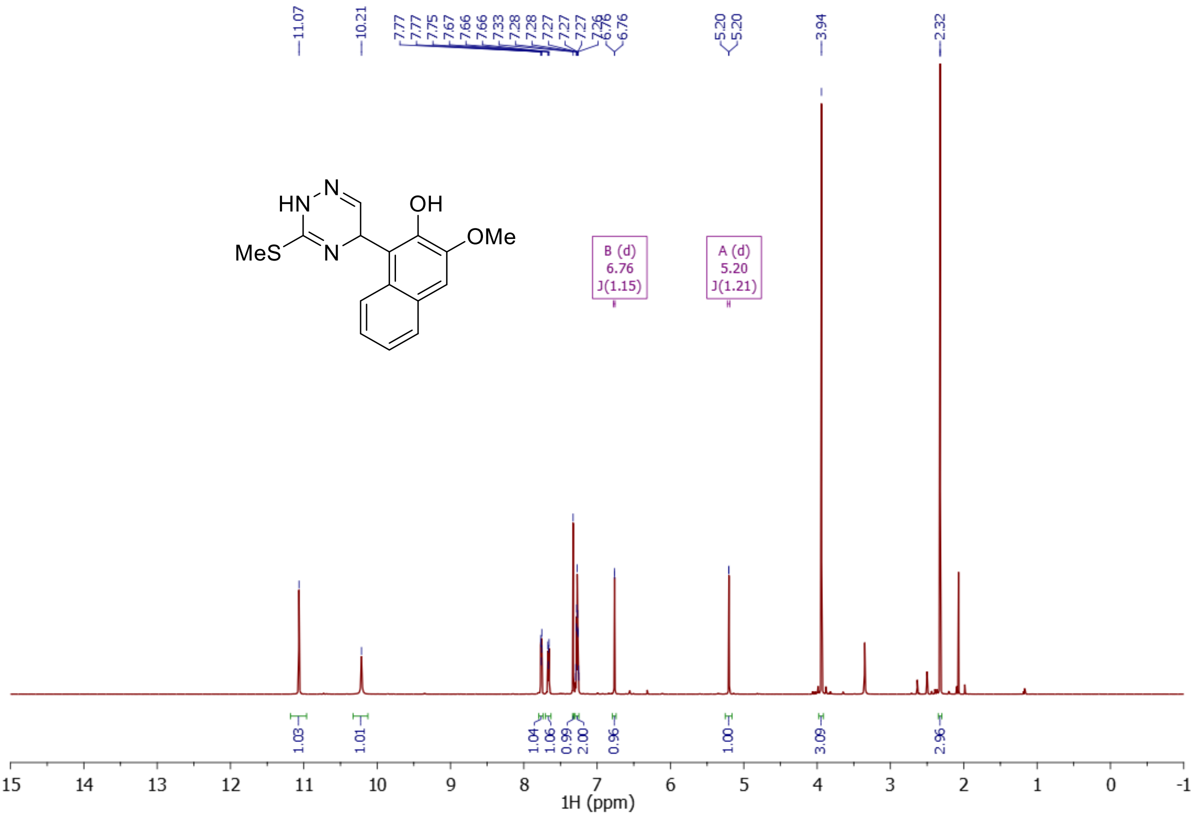

^{1}H NMR spectrum of 3-methoxy-1-(3-(methylthio)-2,5-dihydro-1,2,4-triazin-5-yl)naphthalen-2-ol **3ab**

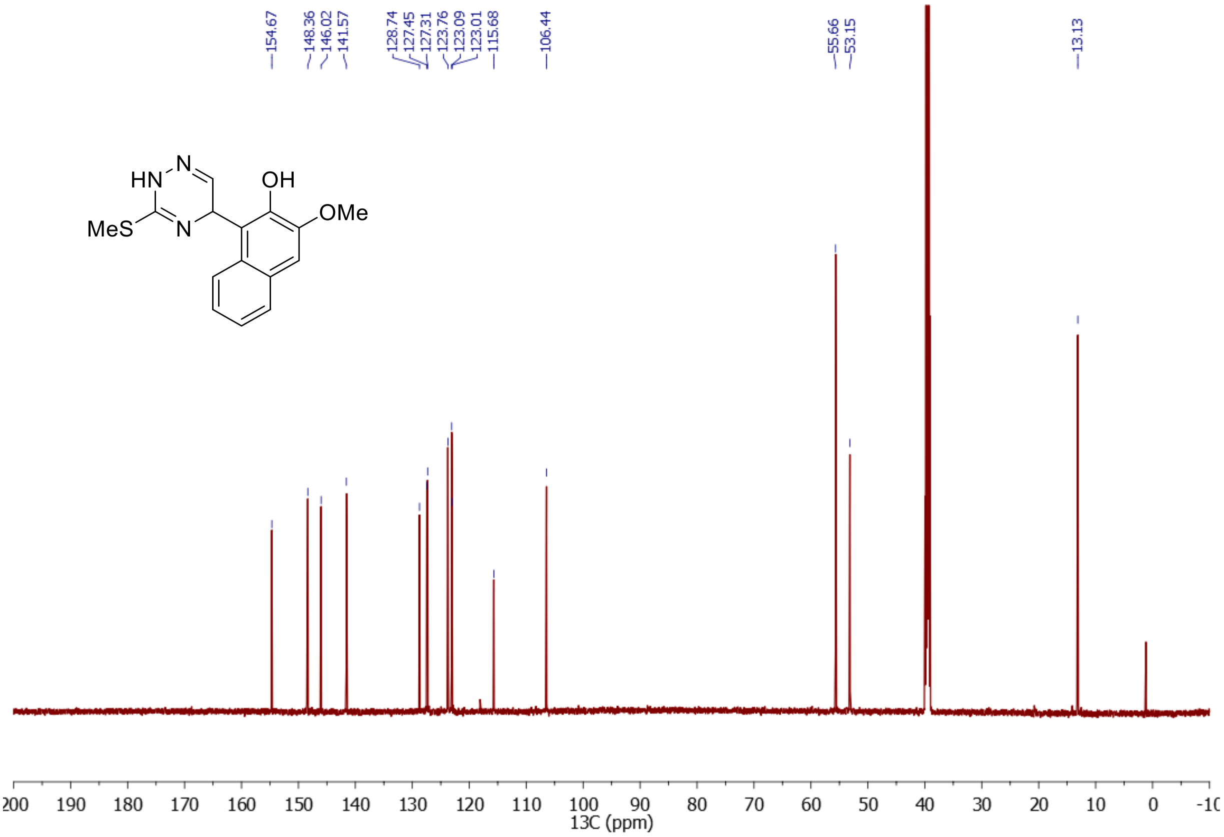

^{13}C NMR spectrum of 3-methoxy-1-(3-(methylthio)-2,5-dihydro-1,2,4-triazin-5-yl)naphthalen-2-ol **3ab**

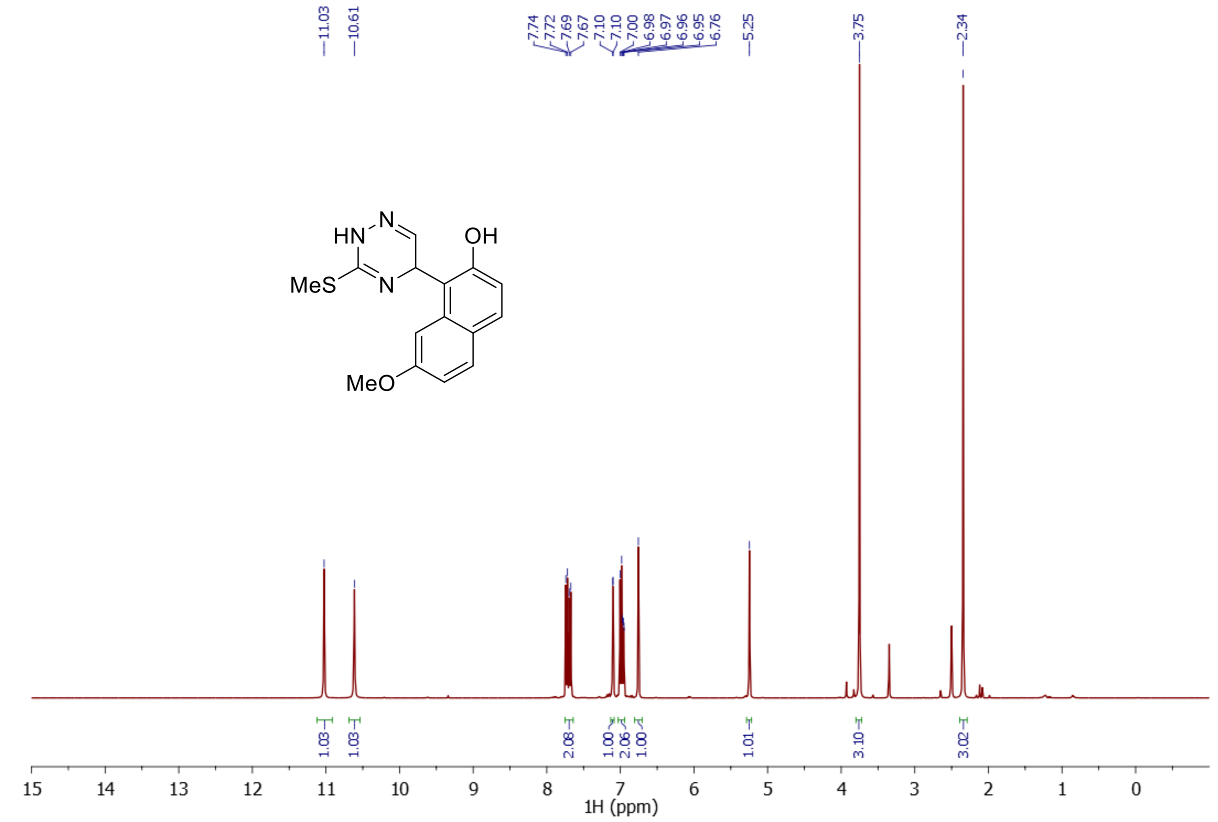

^{1}H NMR spectrum of 7-methoxy-1-(3-(methylthio)-2,5-dihydro-1,2,4-triazin-5-yl)naphthalen-2-ol **3ac**

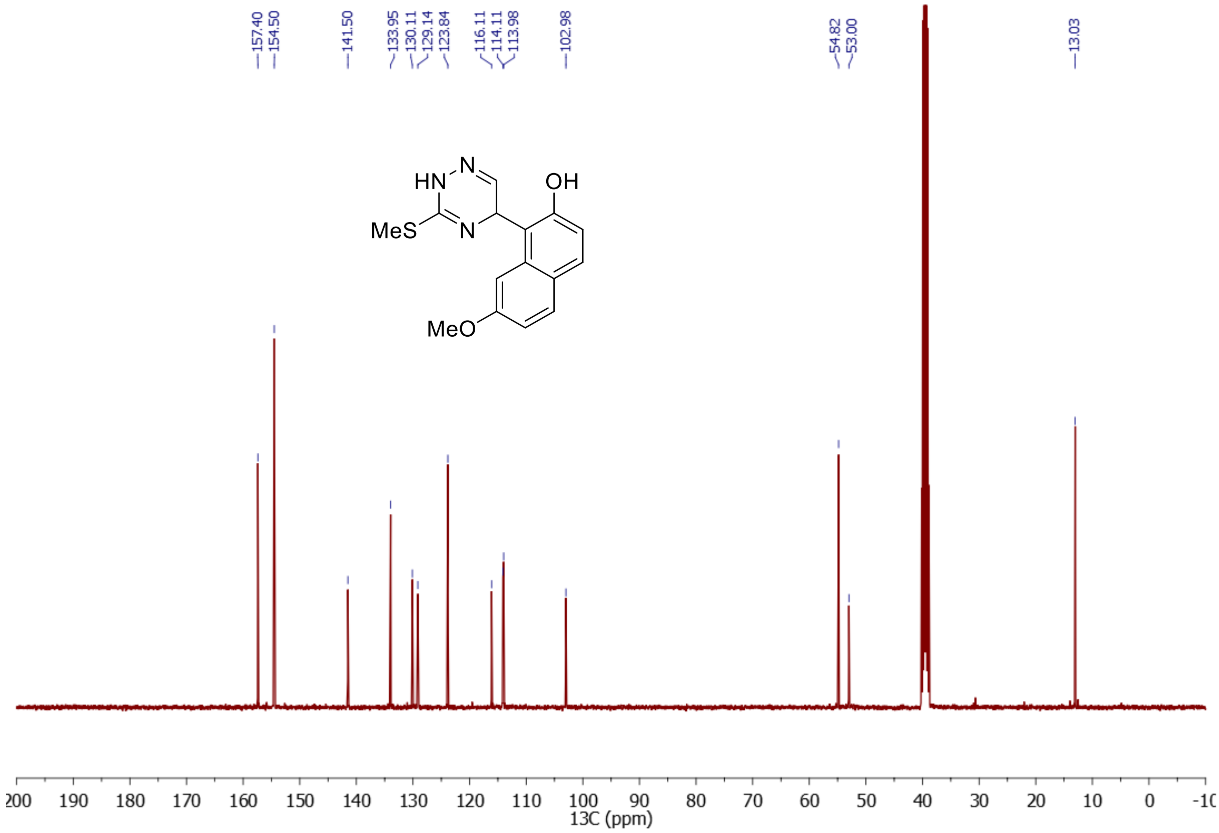

^{13}C NMR spectrum of 7-methoxy-1-(3-(methylthio)-2,5-dihydro-1,2,4-triazin-5-yl)naphthalen-2-ol **3ac**

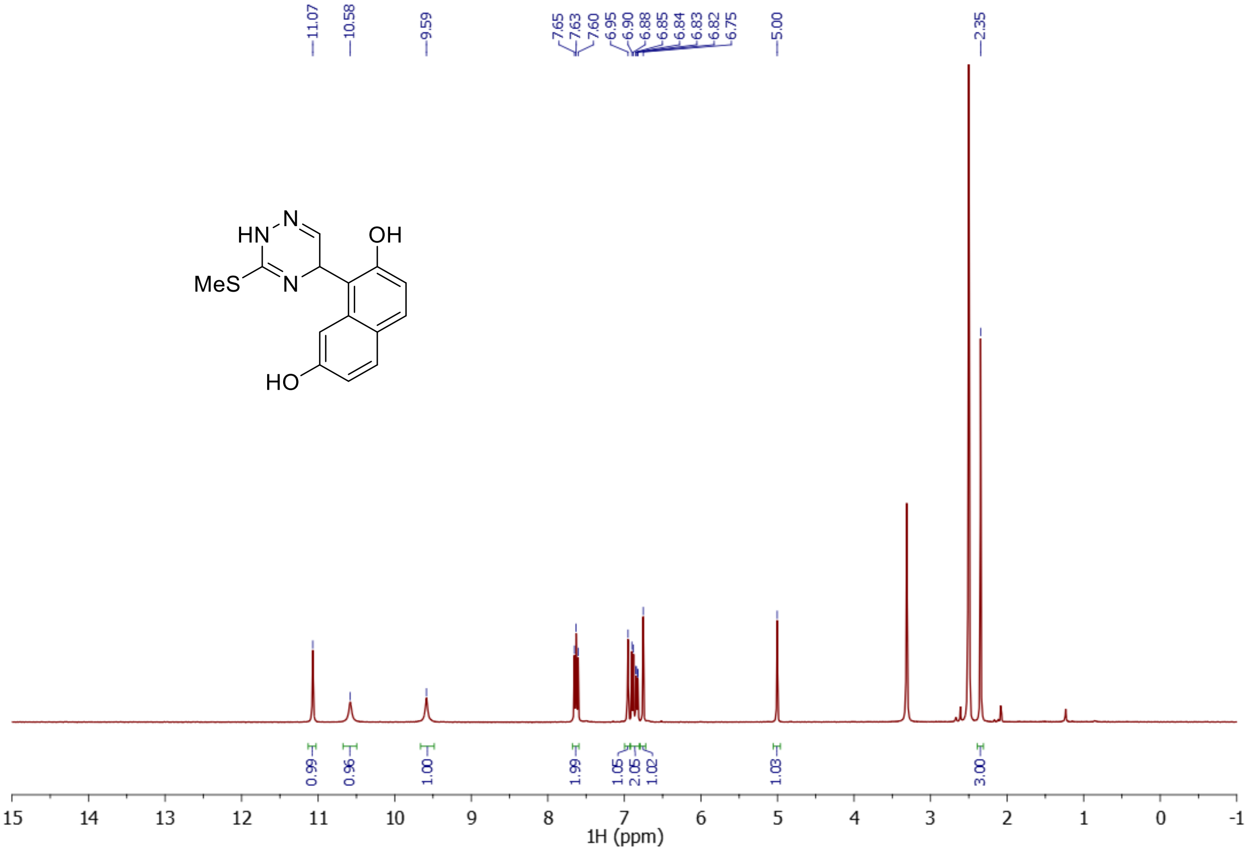

^{1}H NMR spectrum of 1-(3-(methylthio)-2,5-dihydro-1,2,4-triazin-5-yl)naphthalene-2,7-diol **3ad**

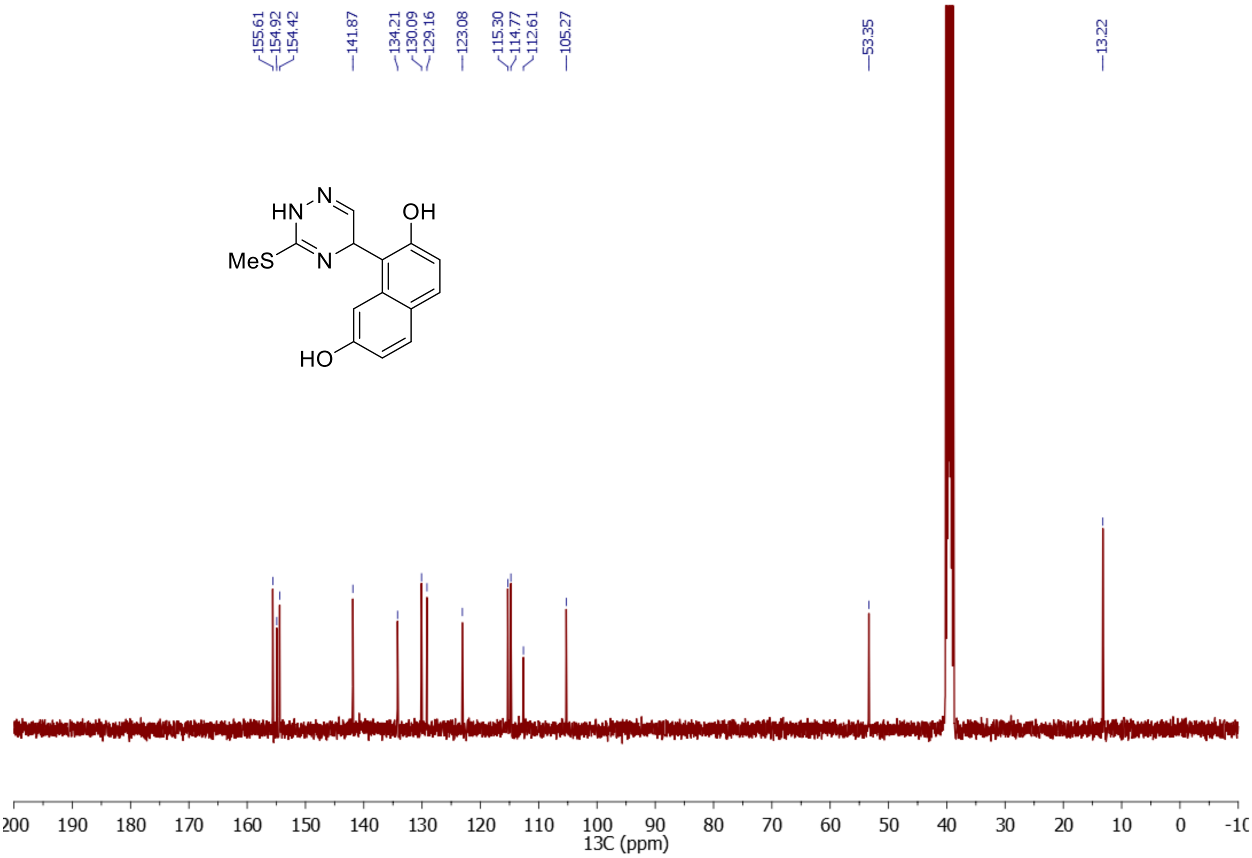

^{13}C NMR spectrum of 1-(3-(methylthio)-2,5-dihydro-1,2,4-triazin-5-yl)naphthalene-2,7-diol **3ad**

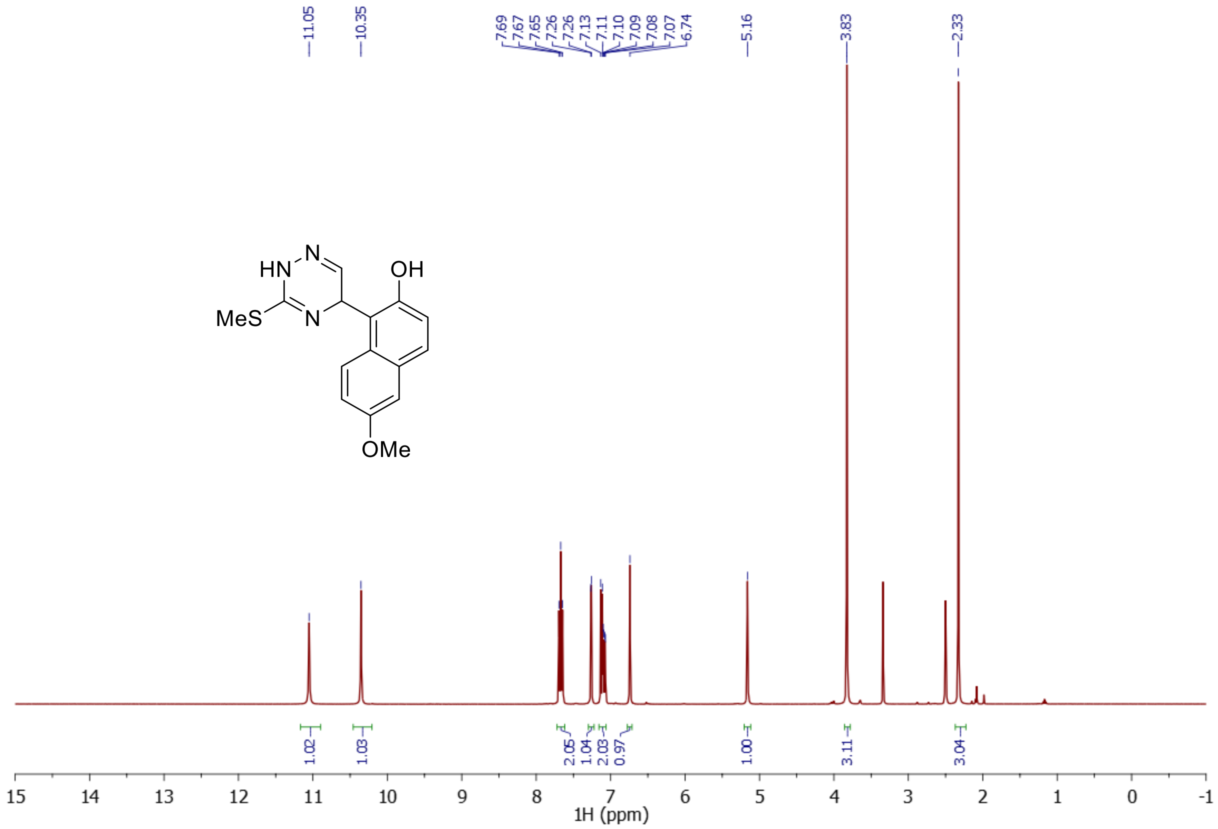

^{1}H NMR spectrum of 6-methoxy-1-(3-(methylthio)-2,5-dihydro-1,2,4-triazin-5-yl)naphthalen-2-ol **3ae**

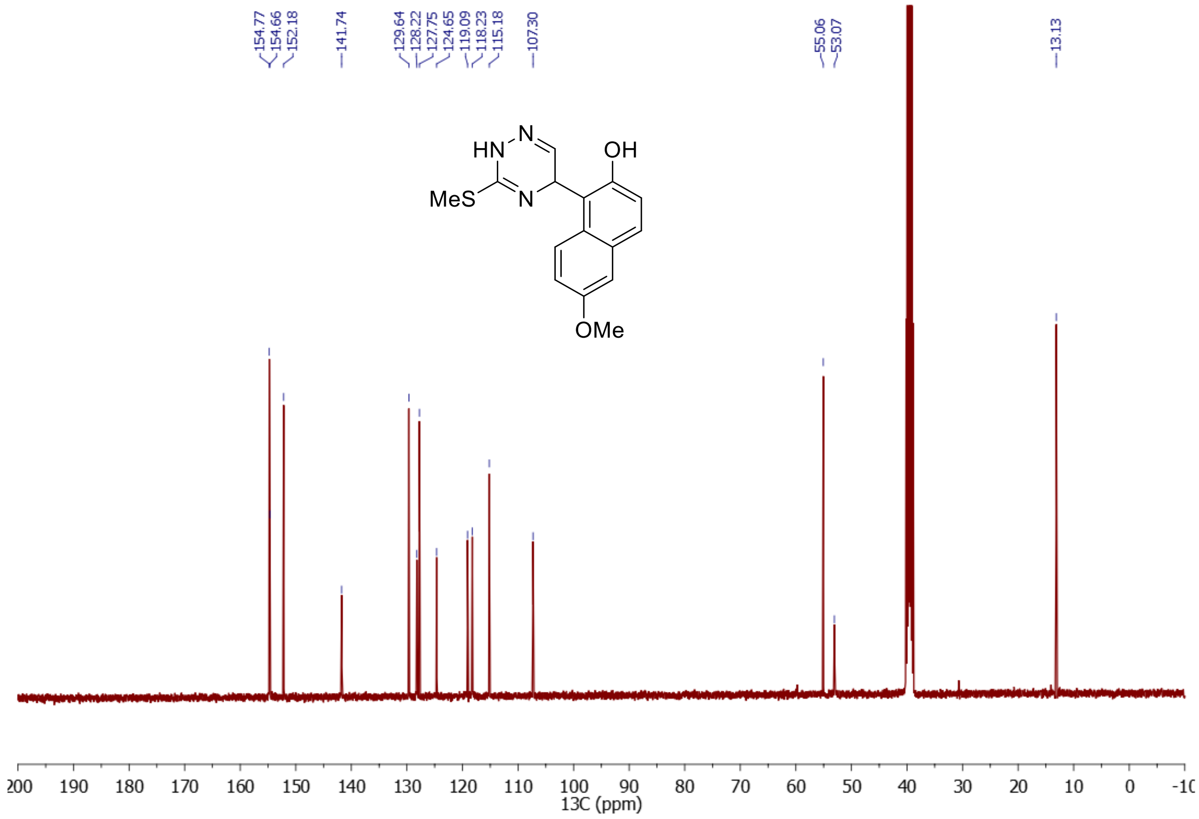

^{13}C NMR spectrum of 6-methoxy-1-(3-(methylthio)-2,5-dihydro-1,2,4-triazin-5-yl)naphthalen-2-ol **3ae**

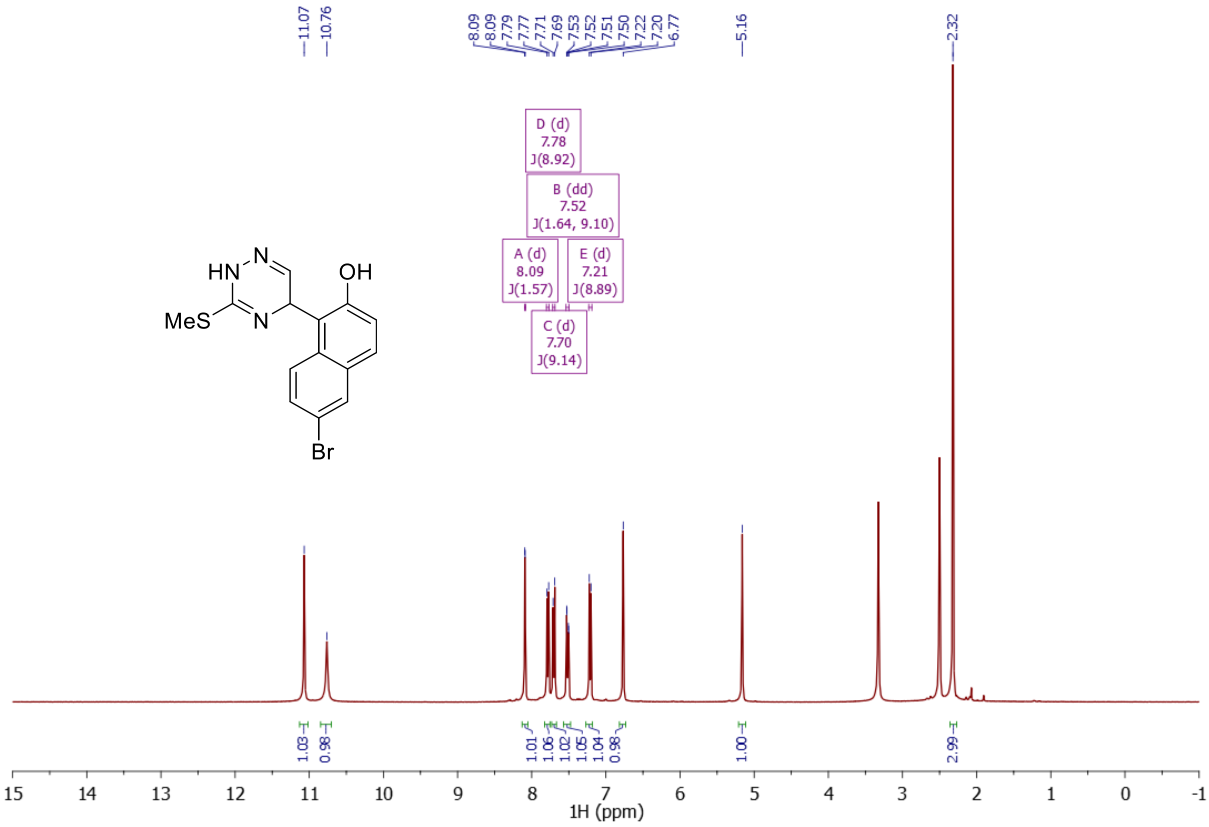

^{1}H NMR spectrum of 6-bromo-1-(3-(methylthio)-2,5-dihydro-1,2,4-triazin-5-yl)naphthalen-2-ol **3af**

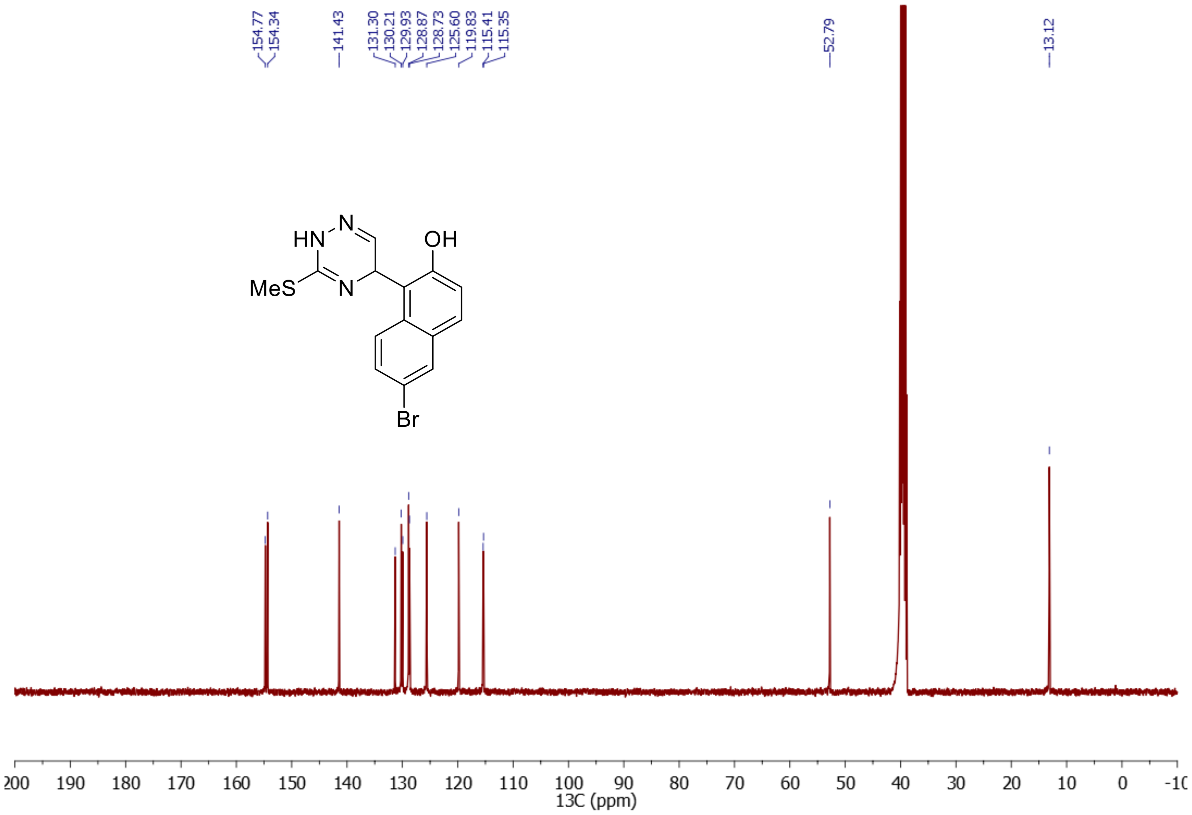

^{13}C NMR spectrum of 6-bromo-1-(3-(methylthio)-2,5-dihydro-1,2,4-triazin-5-yl)naphthalen-2-ol **3af**

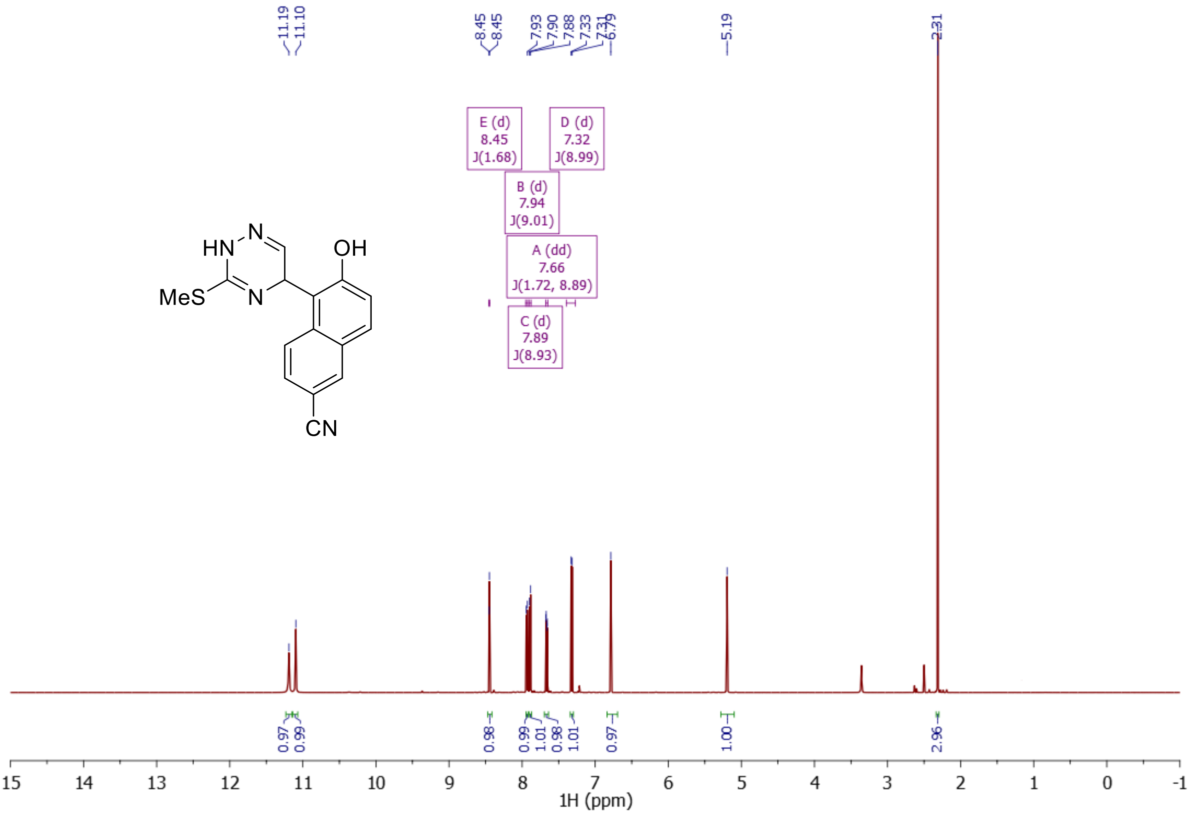

^{1}H NMR spectrum of 6-hydroxy-5-(3-(methylthio)-2,5-dihydro-1,2,4-triazin-5-yl)-2-naphthonitrile **3ag**

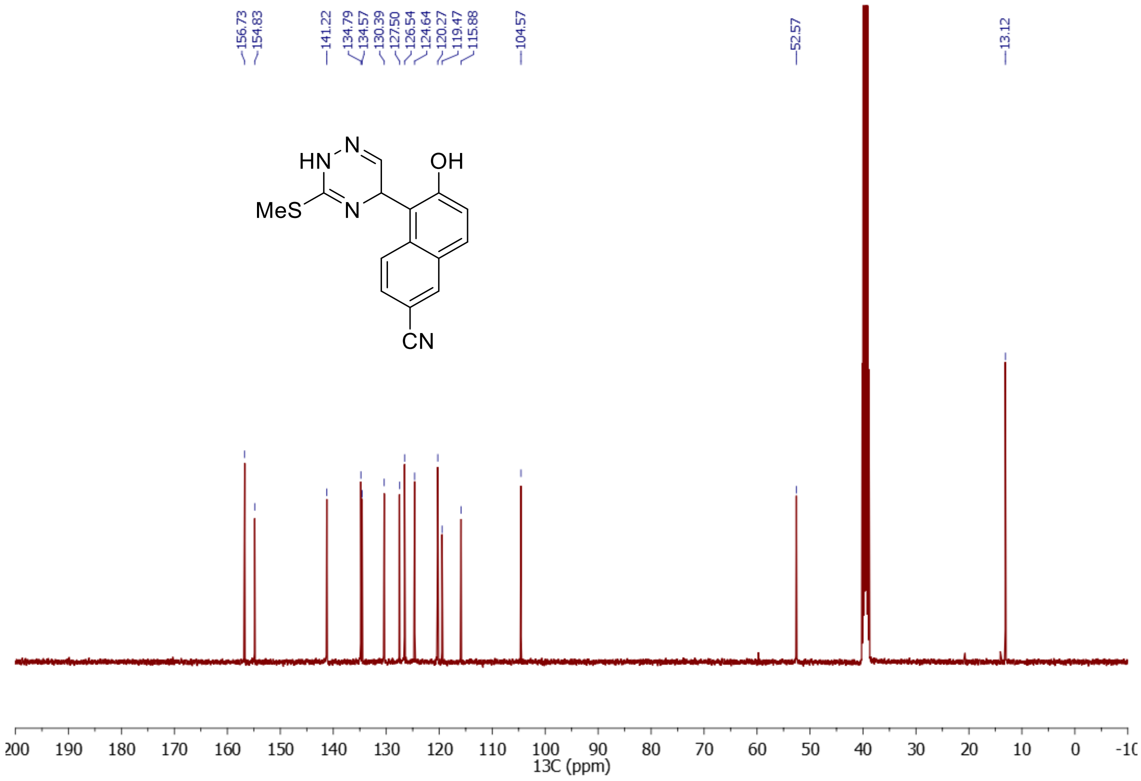

^{13}C NMR spectrum of 6-hydroxy-5-(3-(methylthio)-2,5-dihydro-1,2,4-triazin-5-yl)-2-naphthonitrile **3ag**

Copies of ^{1}H and ^{13}C NMR spectra for compounds 4

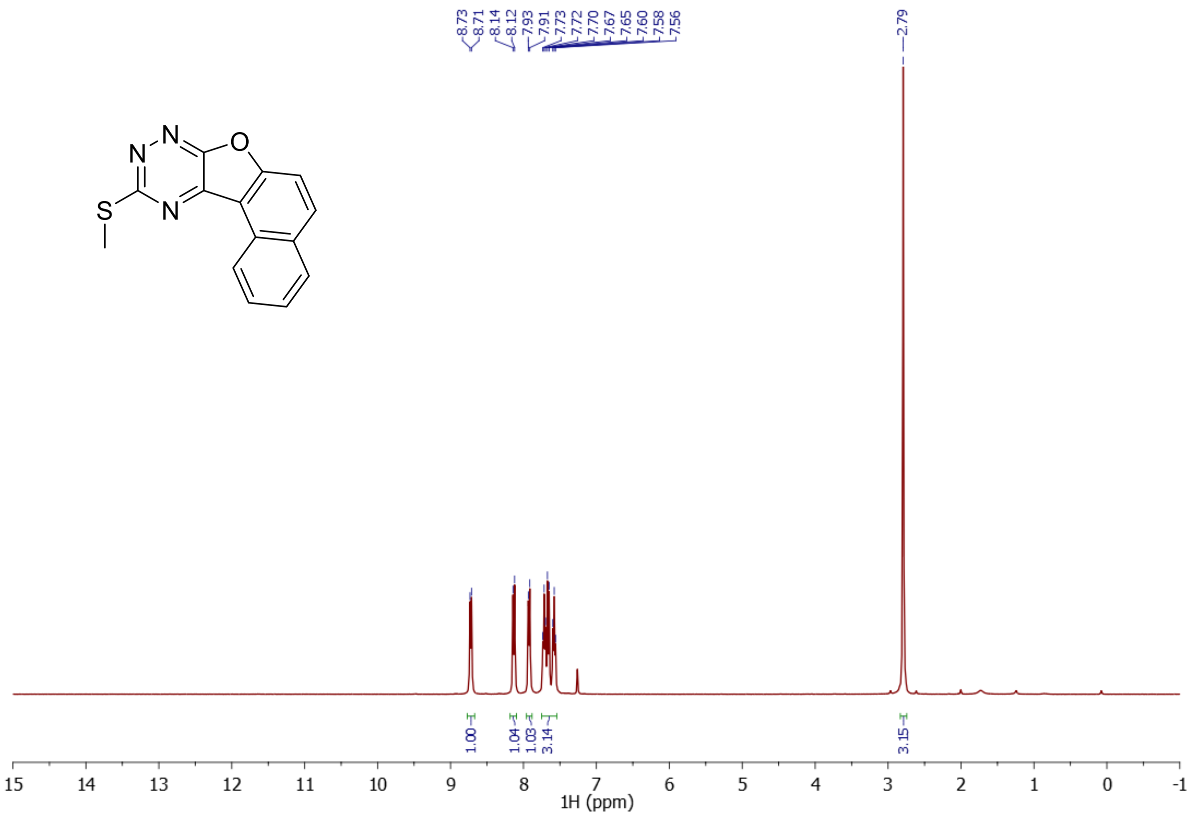

^{1}H NMR spectrum of 10-(methylthio)naphtho[1',2':4,5]furo[3,2-*e*][1,2,4]triazine **4aa**

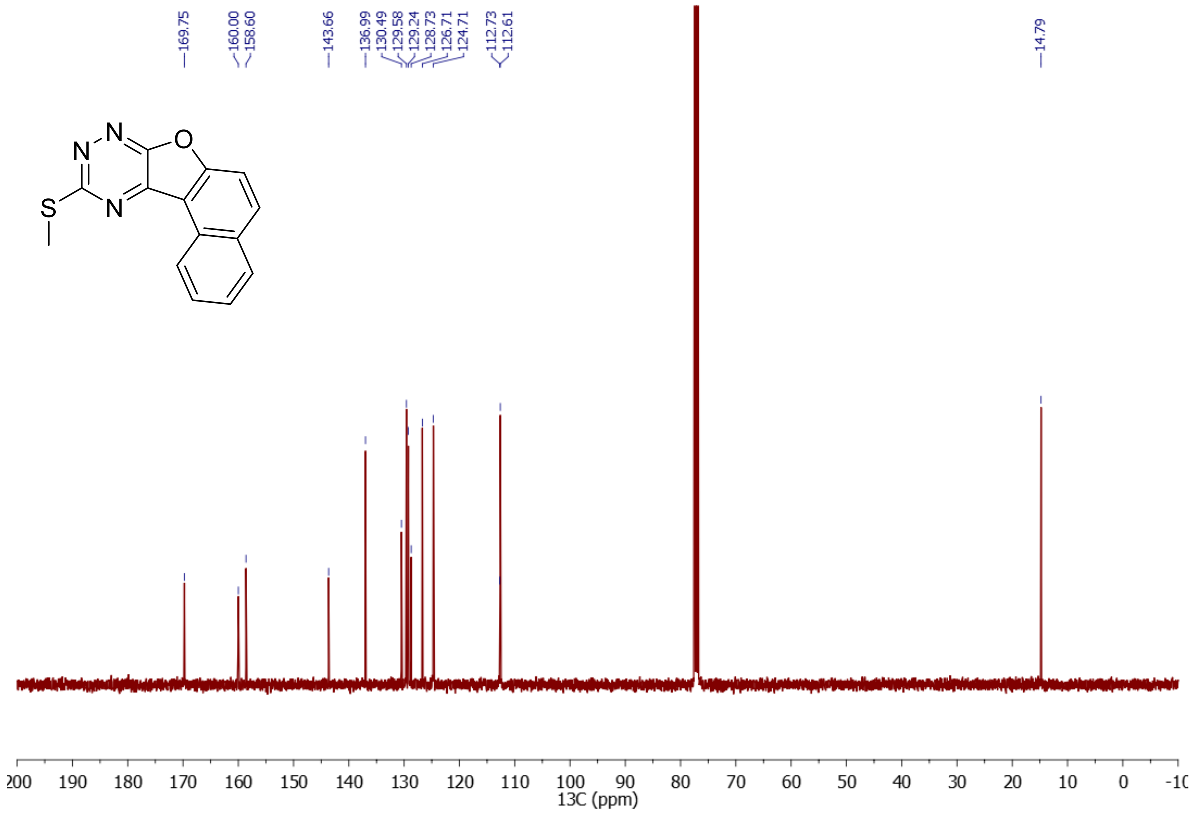

^{13}C NMR spectrum of 10-(methylthio)naphtho[1',2':4,5]furo[3,2-*e*][1,2,4]triazine **4aa**

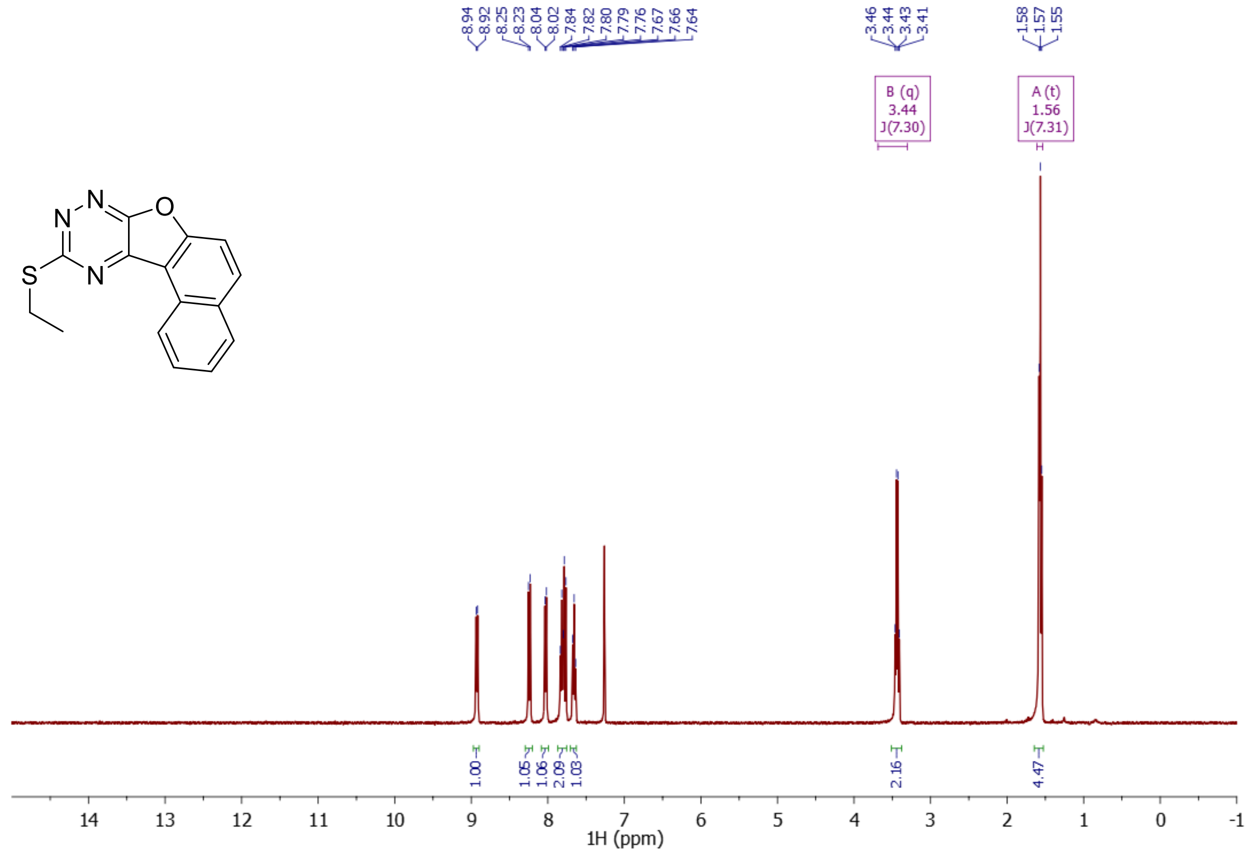

^{1}H NMR spectrum of 10-(ethylthio)naphtho[1',2':4,5]furo[3,2-*e*][1,2,4]triazine **4ba**

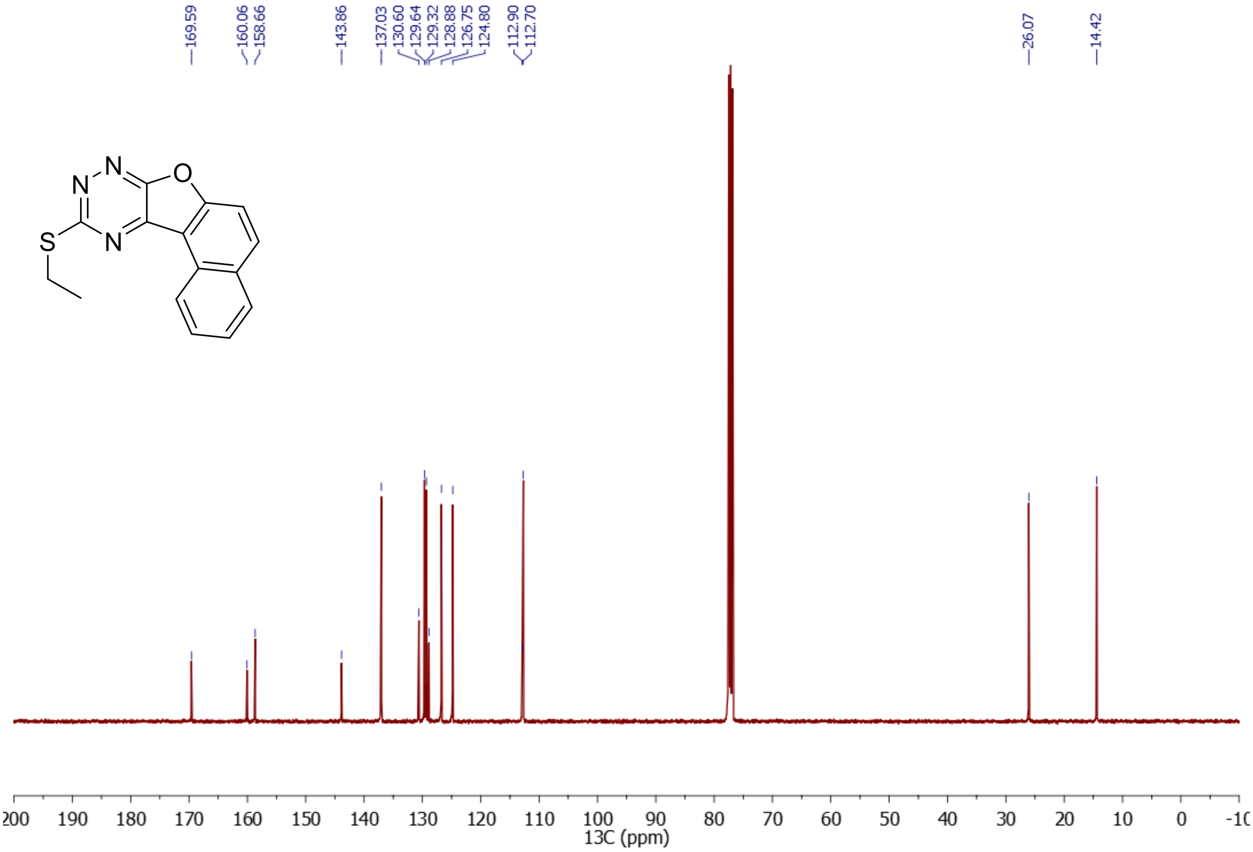

^{13}C NMR spectrum of 10-(ethylthio)naphtho[1',2':4,5]furo[3,2-*e*][1,2,4]triazine **4ba**

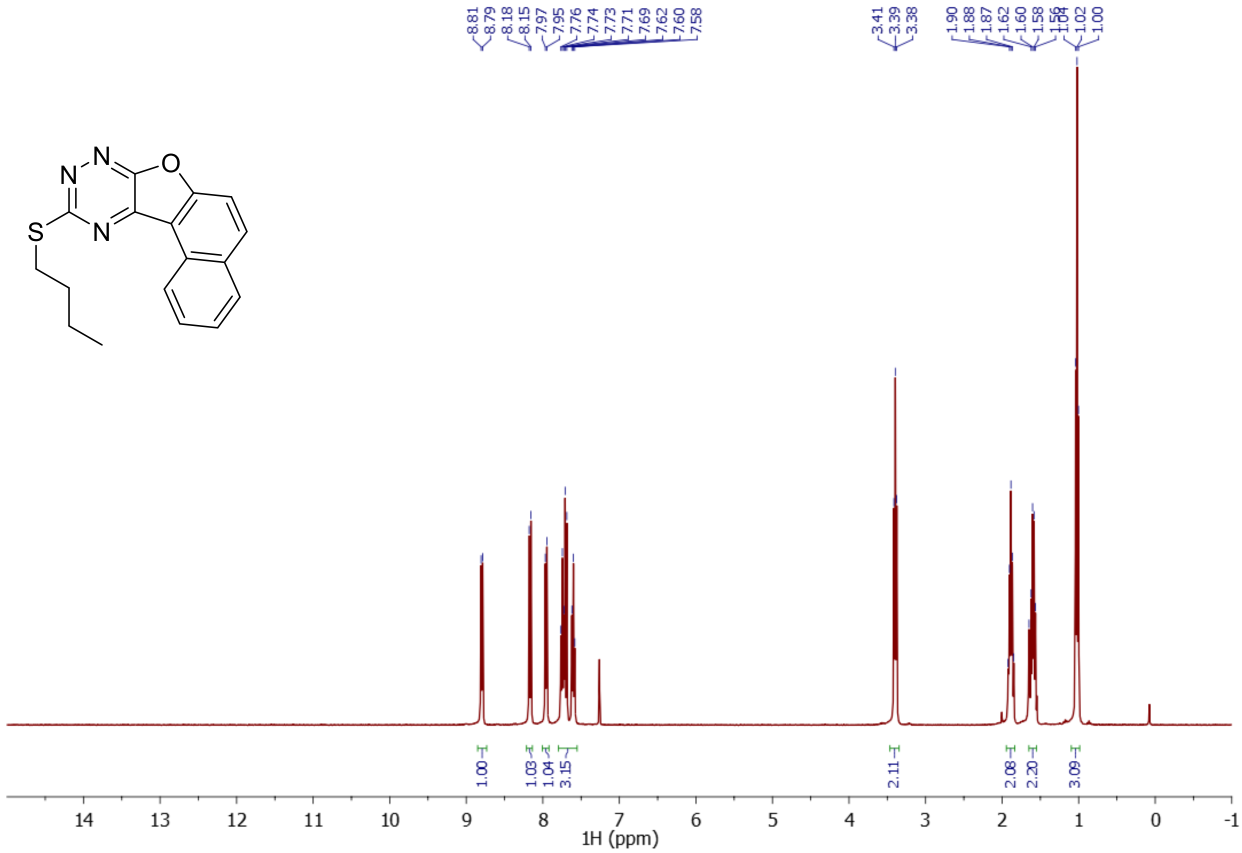

^{1}H NMR spectrum of 10-(butylthio)naphtho[1',2':4,5]furo[3,2-*e*][1,2,4]triazine **4ca**

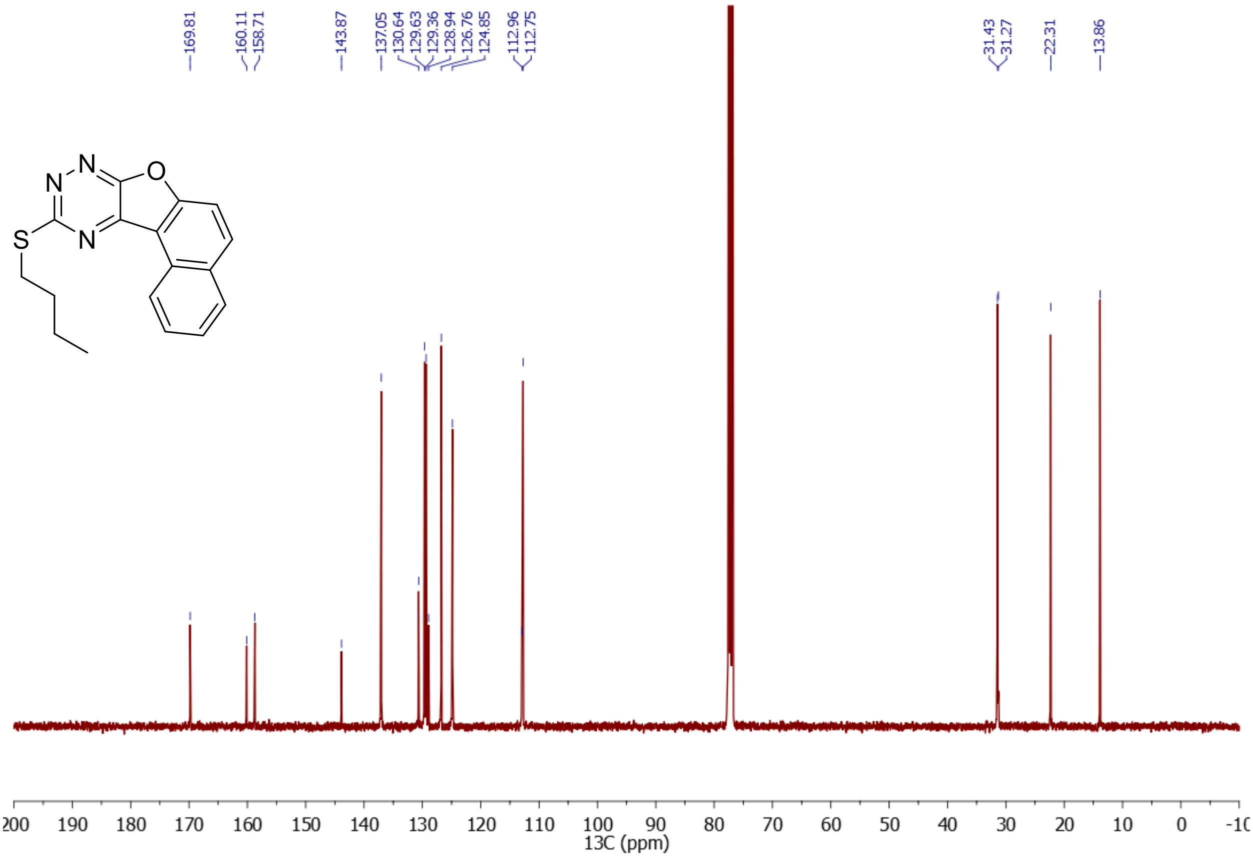

^{13}C NMR spectrum of 10-(butylthio)naphtho[1',2':4,5]furo[3,2-*e*][1,2,4]triazine **4ca**

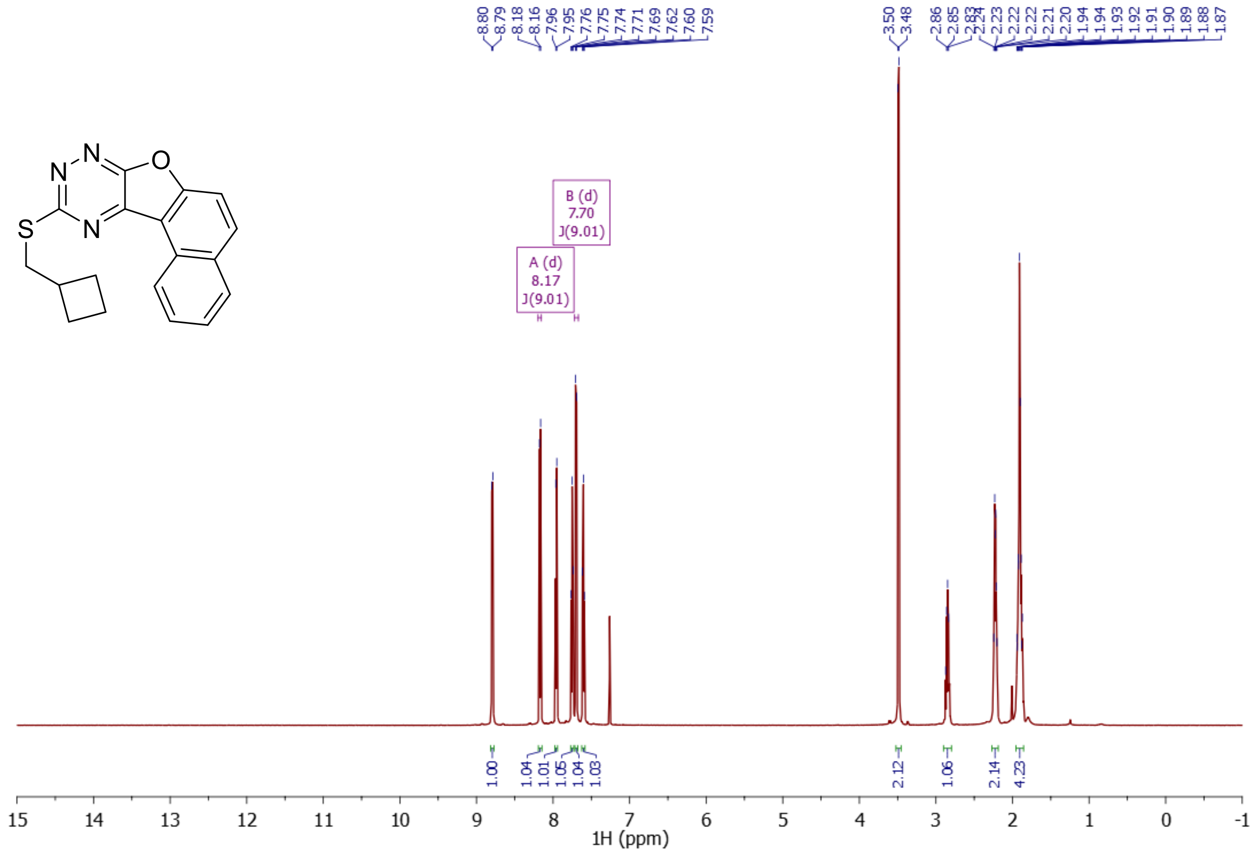

^{1}H NMR spectrum of 10-((cyclobutylmethyl)thio)naphtho[1',2':4,5]furo[3,2-*e*][1,2,4]triazine **4da**

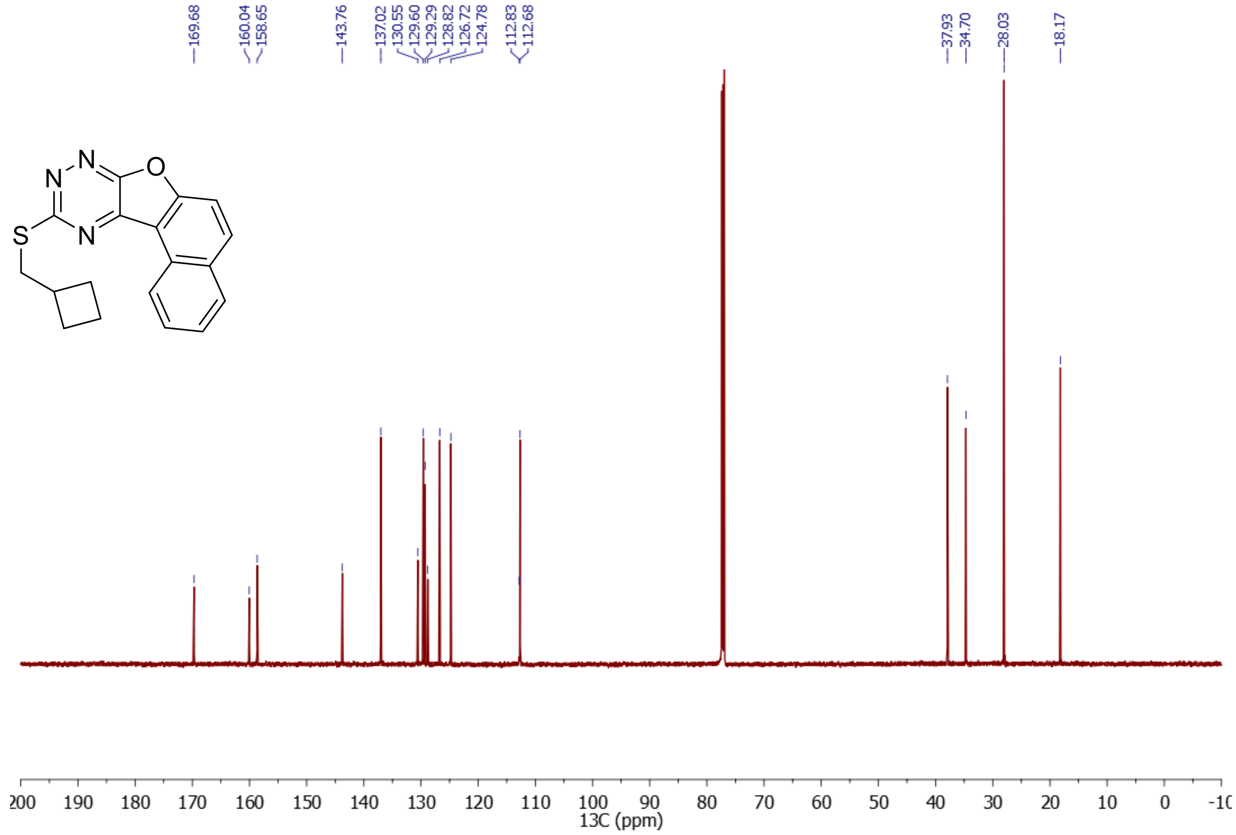

^{13}C NMR spectrum of 10-((cyclobutylmethyl)thio)naphtho[1',2':4,5]furo[3,2-*e*][1,2,4]triazine **4da**

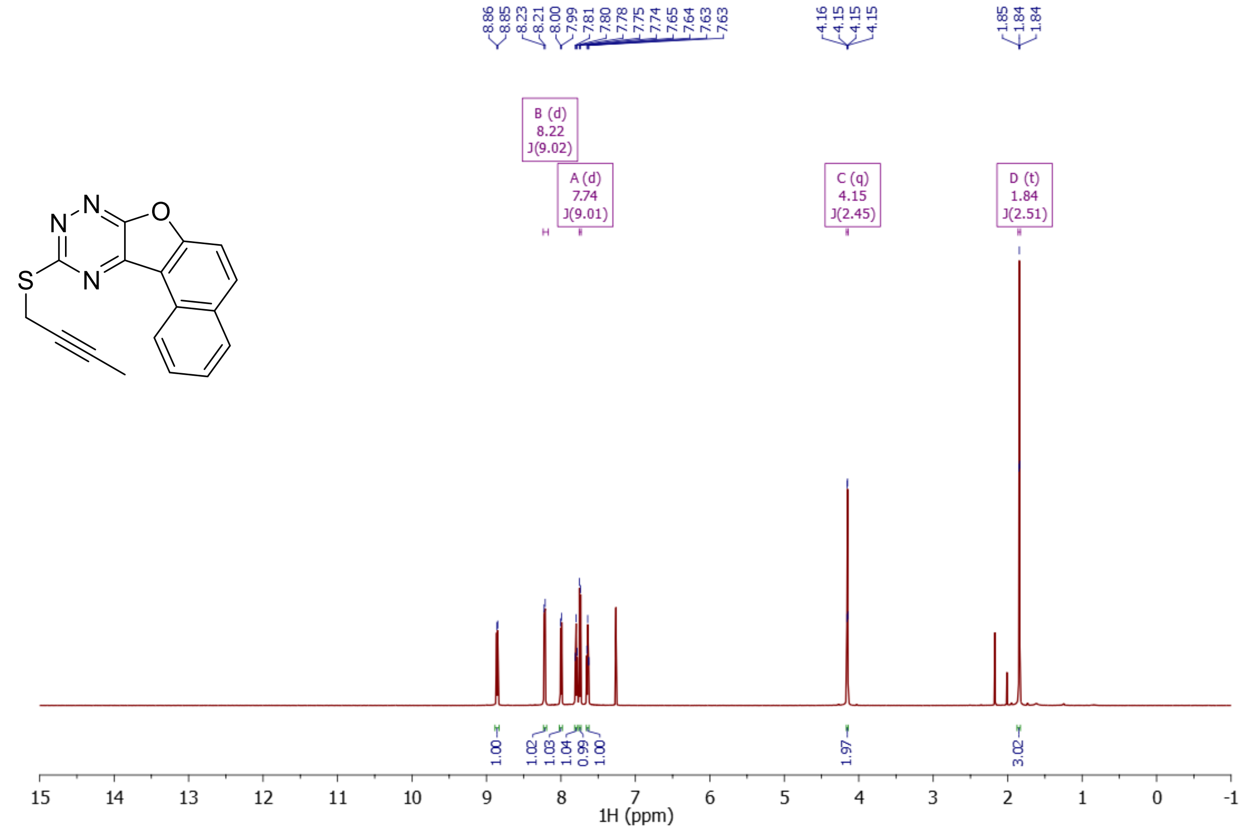

^{1}H NMR spectrum of 10-(but-2-yn-1-ylthio)naphtho[1',2':4,5]furo[3,2-*e*][1,2,4]triazine **4ea**

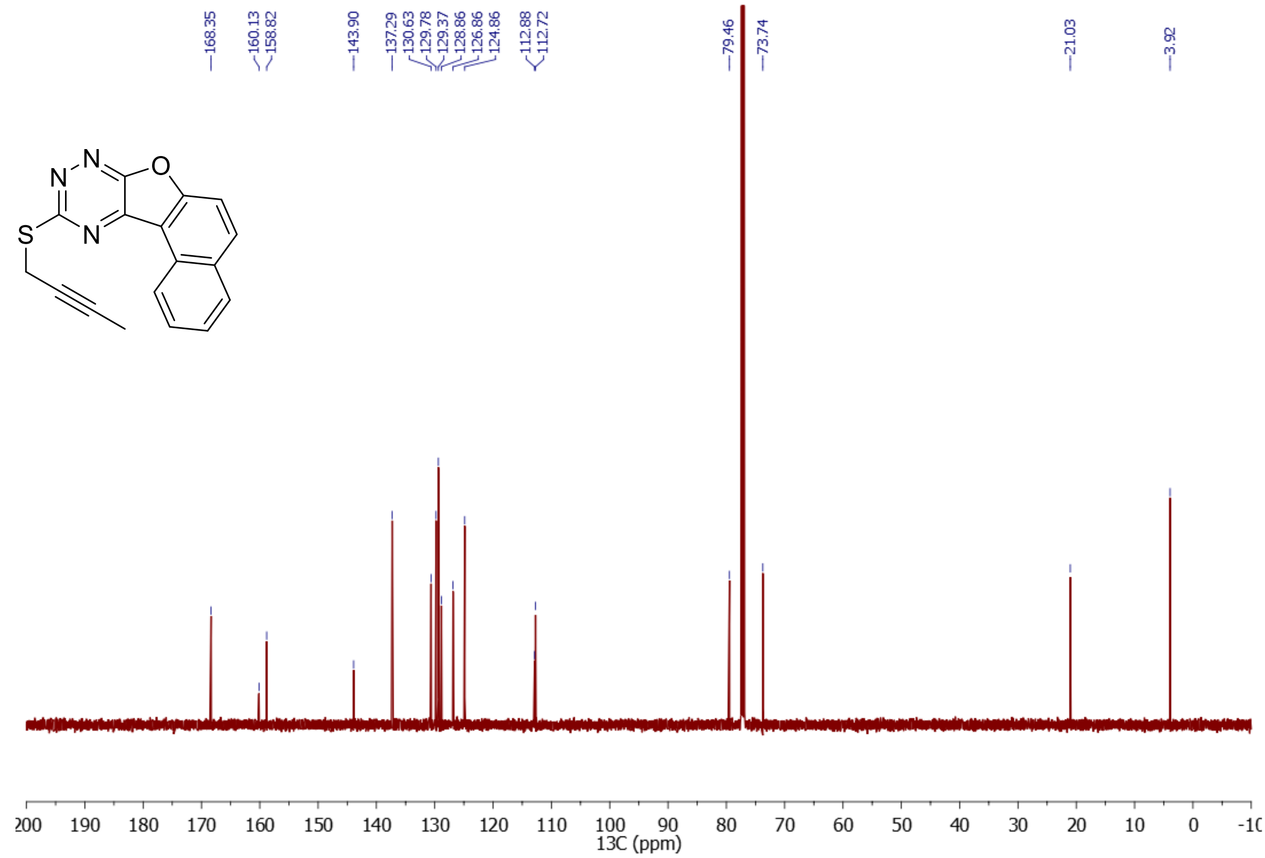

^{13}C NMR spectrum of 10-(but-2-yn-1-ylthio)naphtho[1',2':4,5]furo[3,2-*e*][1,2,4]triazine **4ea**

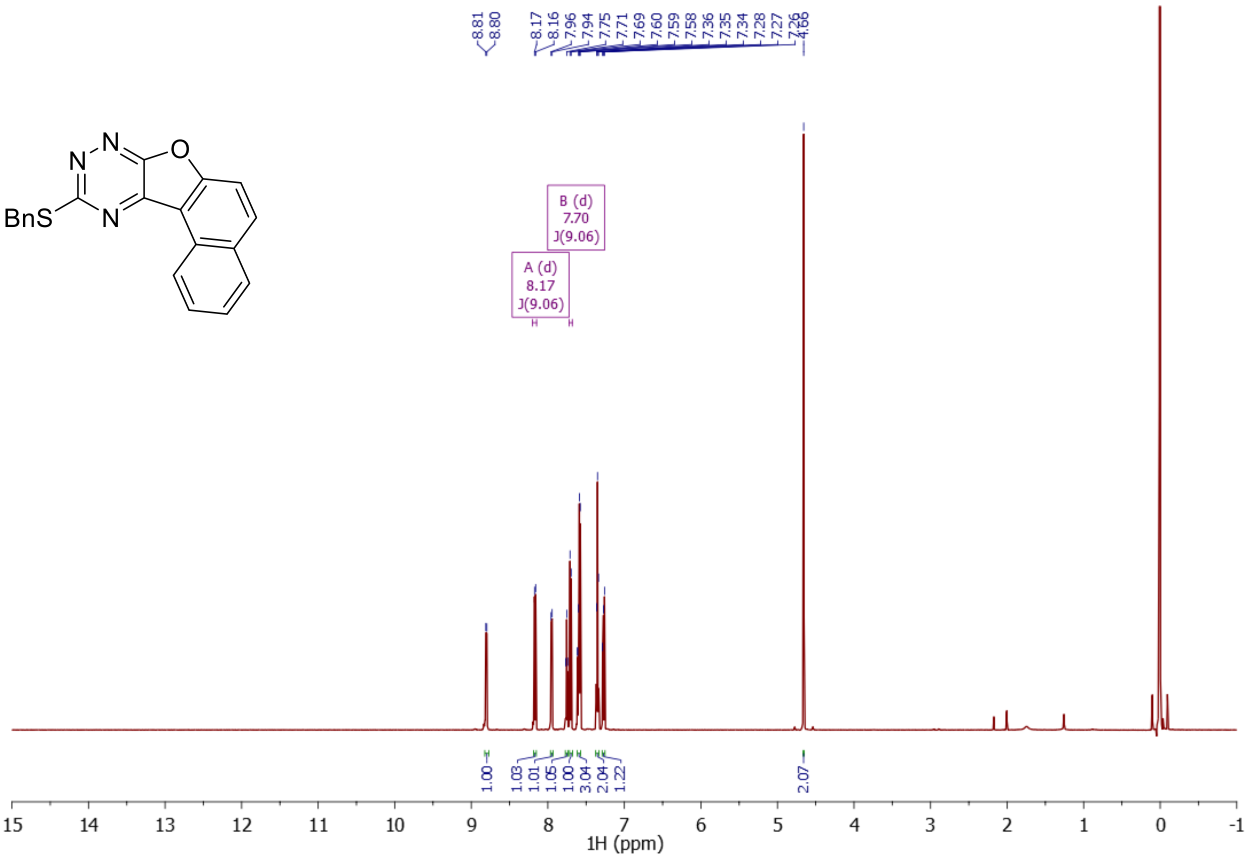

^{1}H NMR spectrum of 10-(benzylthio)naphtho[1',2':4,5]furo[3,2-*e*][1,2,4]triazine **4fa**

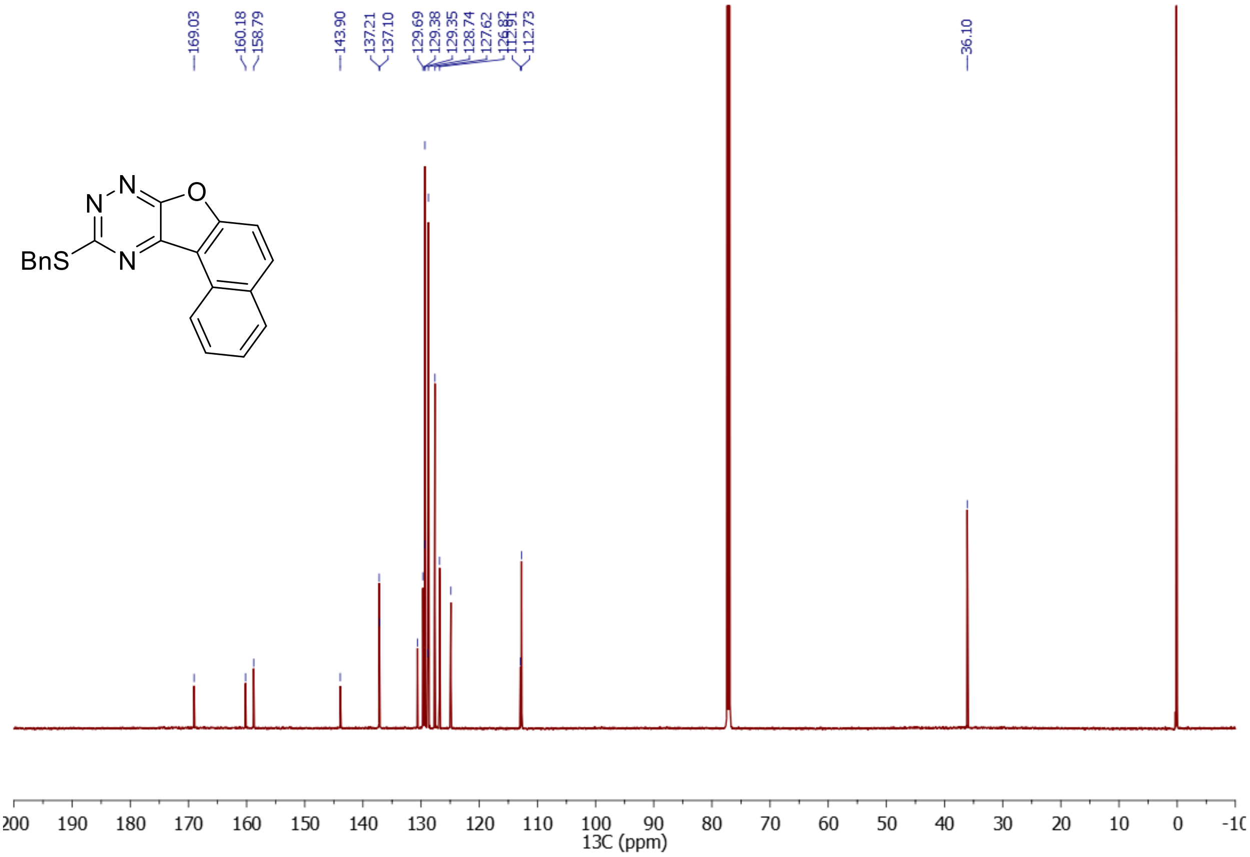

^{13}C NMR spectrum of 10-(benzylthio)naphtho[1',2':4,5]furo[3,2-*e*][1,2,4]triazine **4fa**

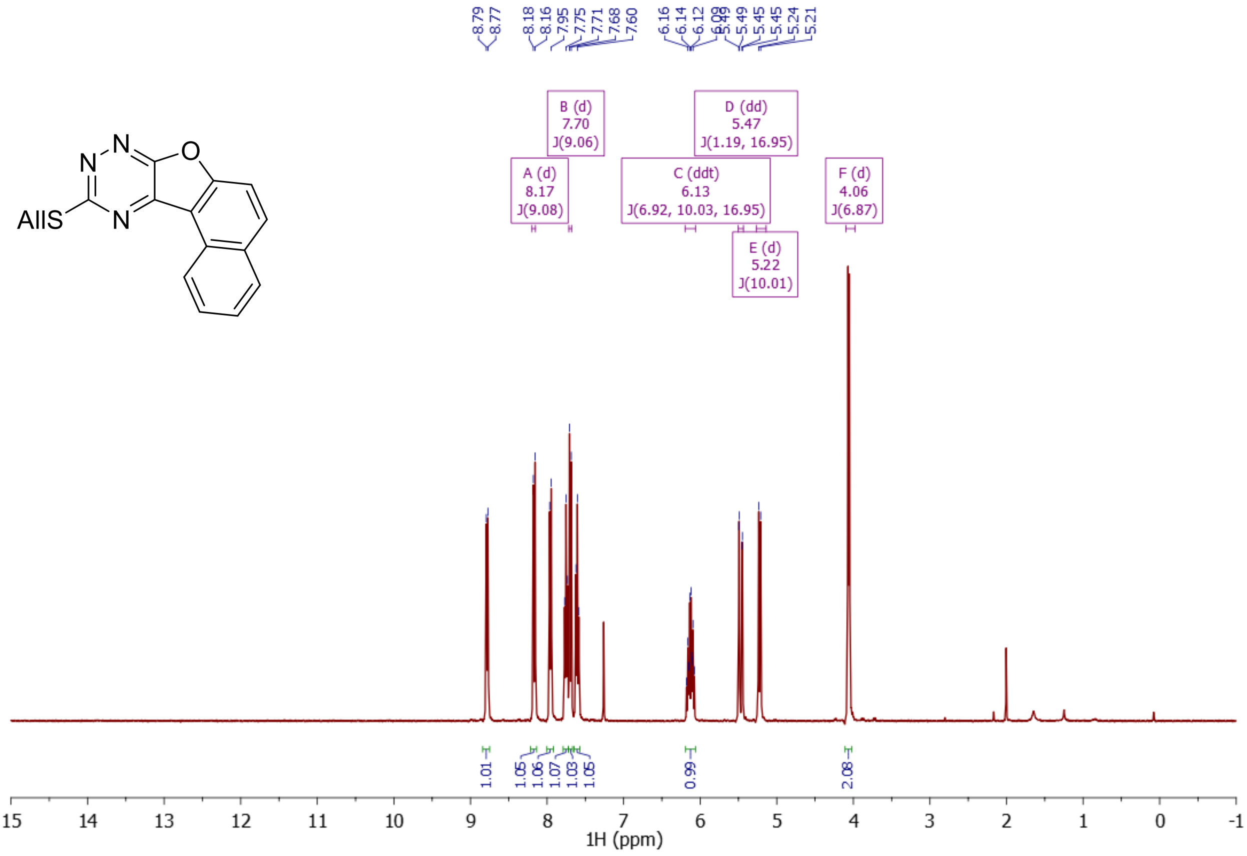

^{1}H NMR spectrum of 10-(allylthio)naphtho[1',2':4,5]furo[3,2-*e*][1,2,4]triazine **4ga**

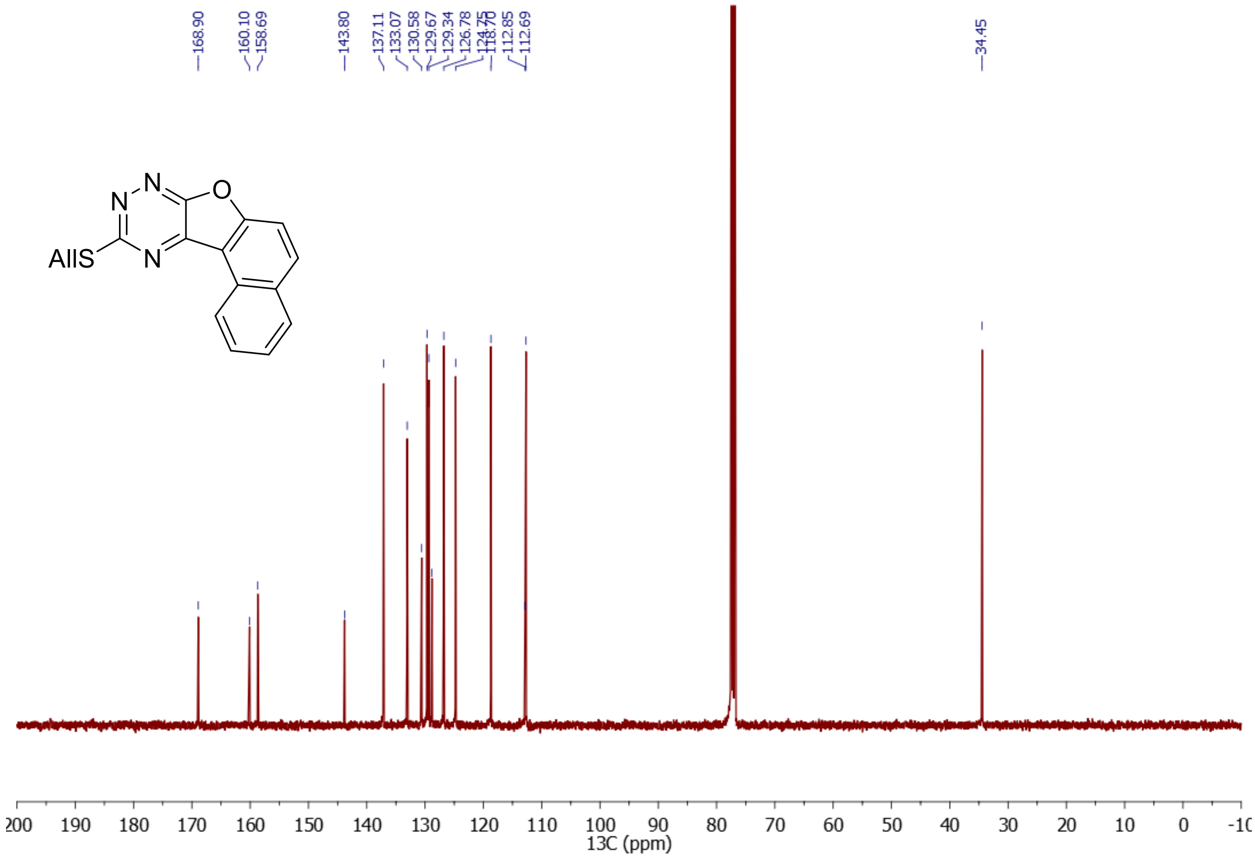

^{13}C NMR spectrum of 10-(allylthio)naphtho[1',2':4,5]furo[3,2-*e*][1,2,4]triazine **4ga**

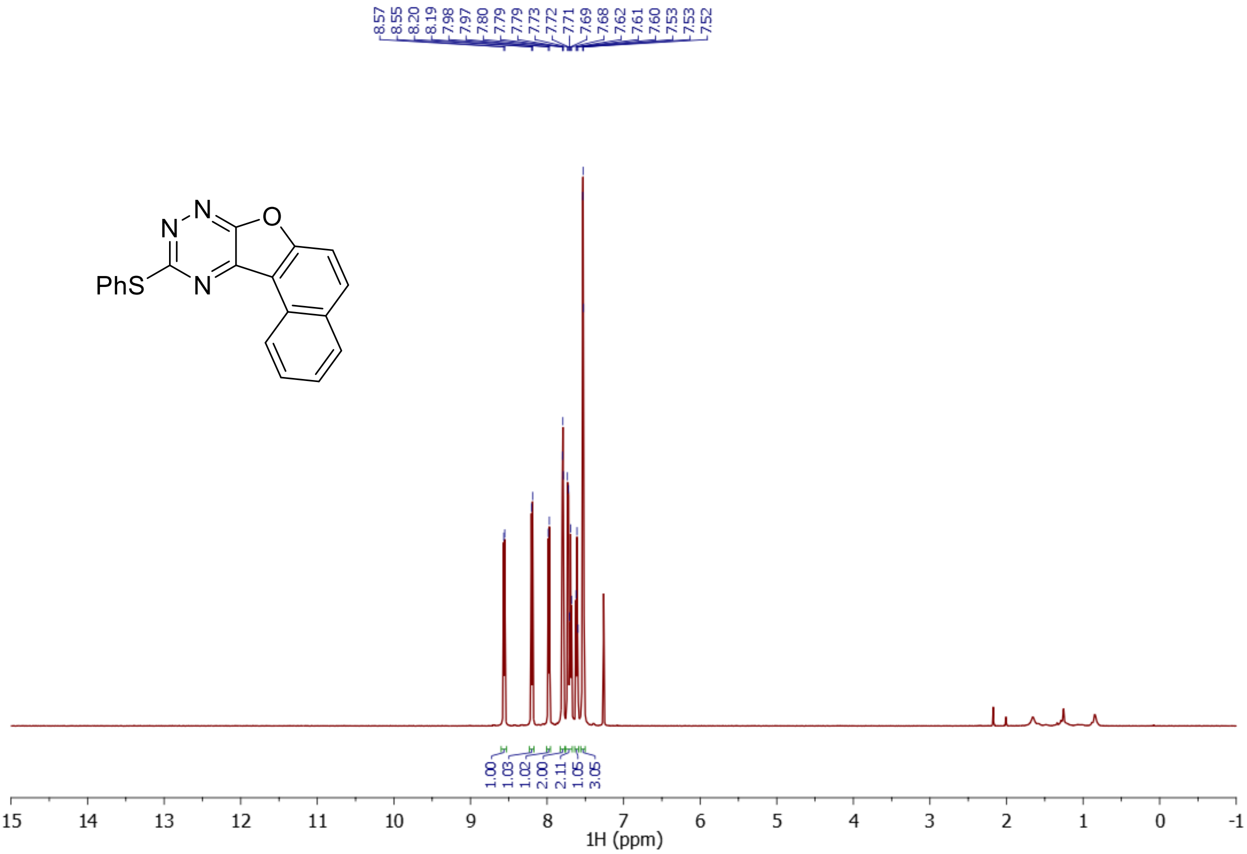

^{1}H NMR spectrum of 10-(phenylthio)naphtho[1',2':4,5]furo[3,2-*e*][1,2,4]triazine **4ha**

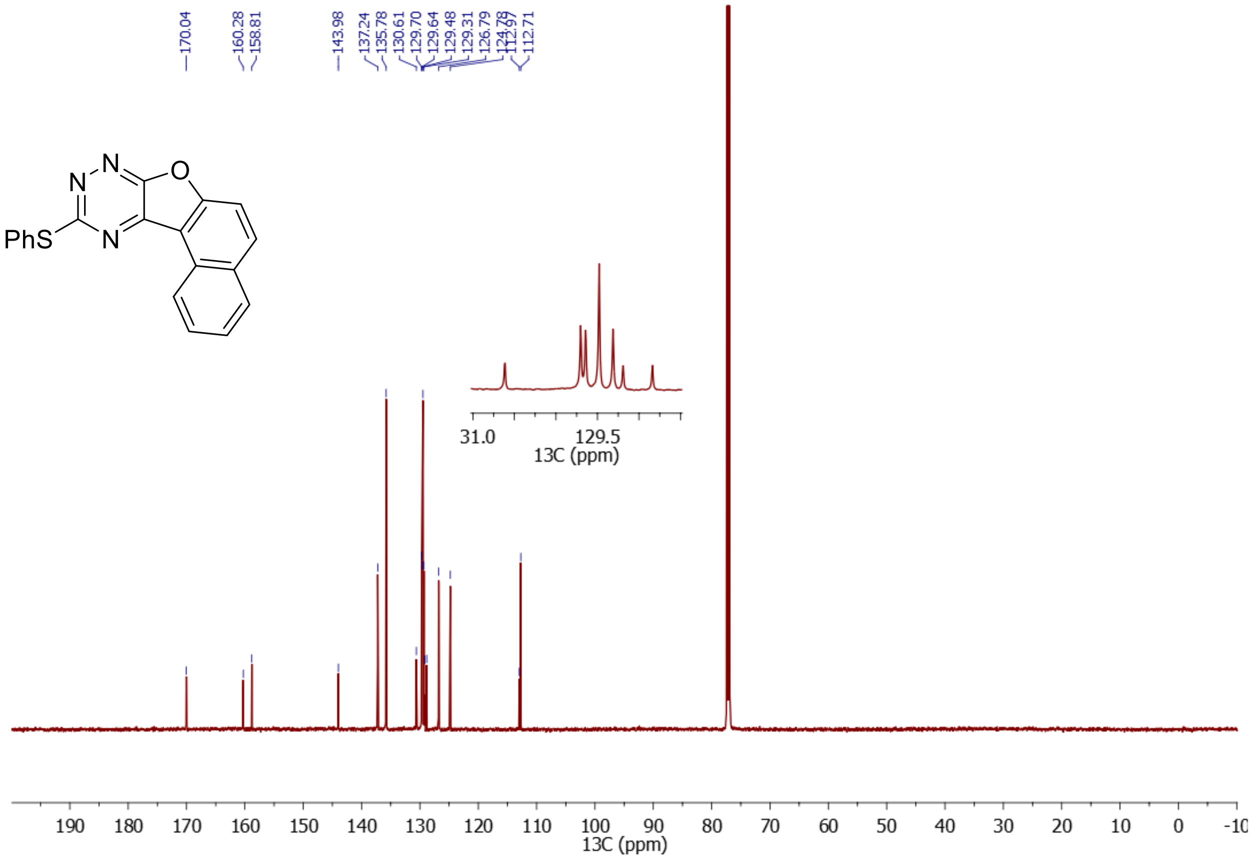

^{13}C NMR spectrum of 10-(phenylthio)naphtho[1',2':4,5]furo[3,2-*e*][1,2,4]triazine **4ha**

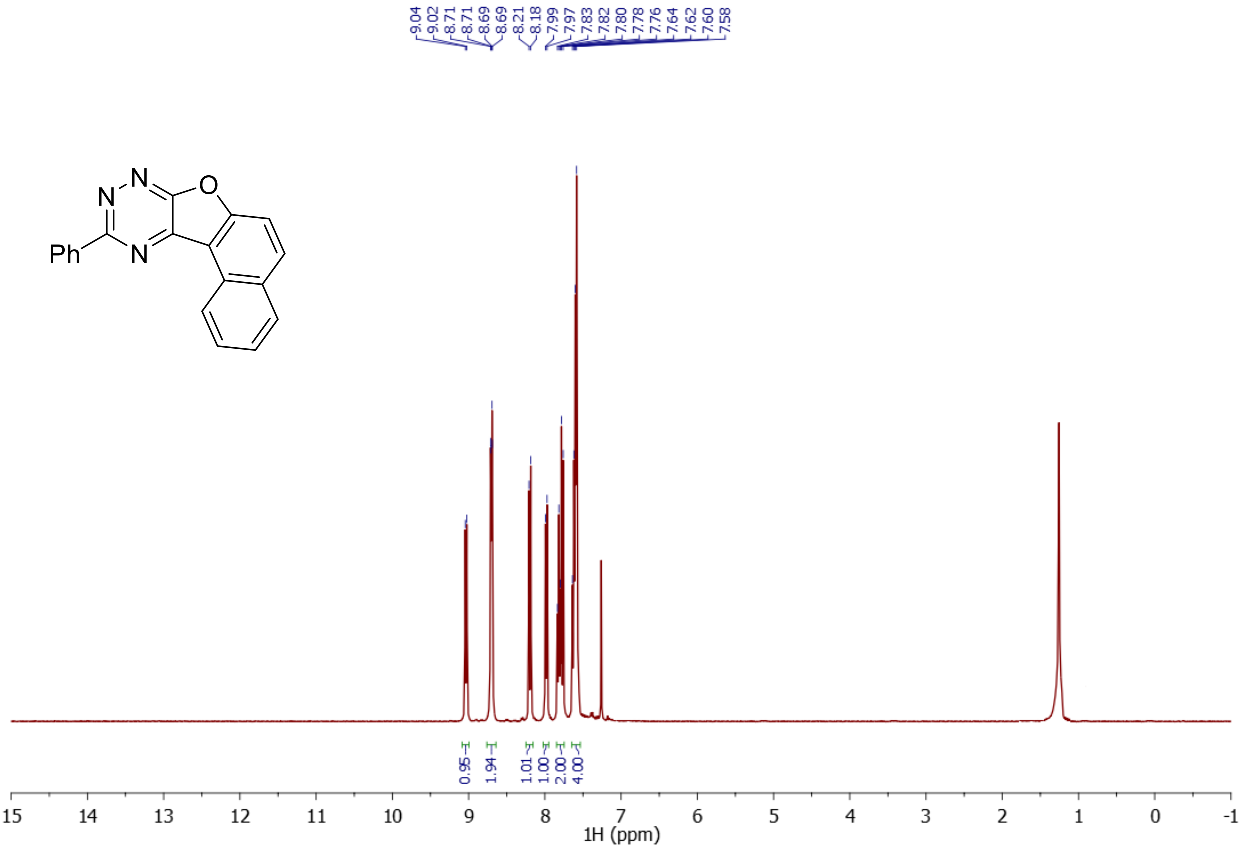

^{1}H NMR spectrum of 10-phenylnaphtho[1',2':4,5]furo[3,2-*e*][1,2,4]triazine **4ia**

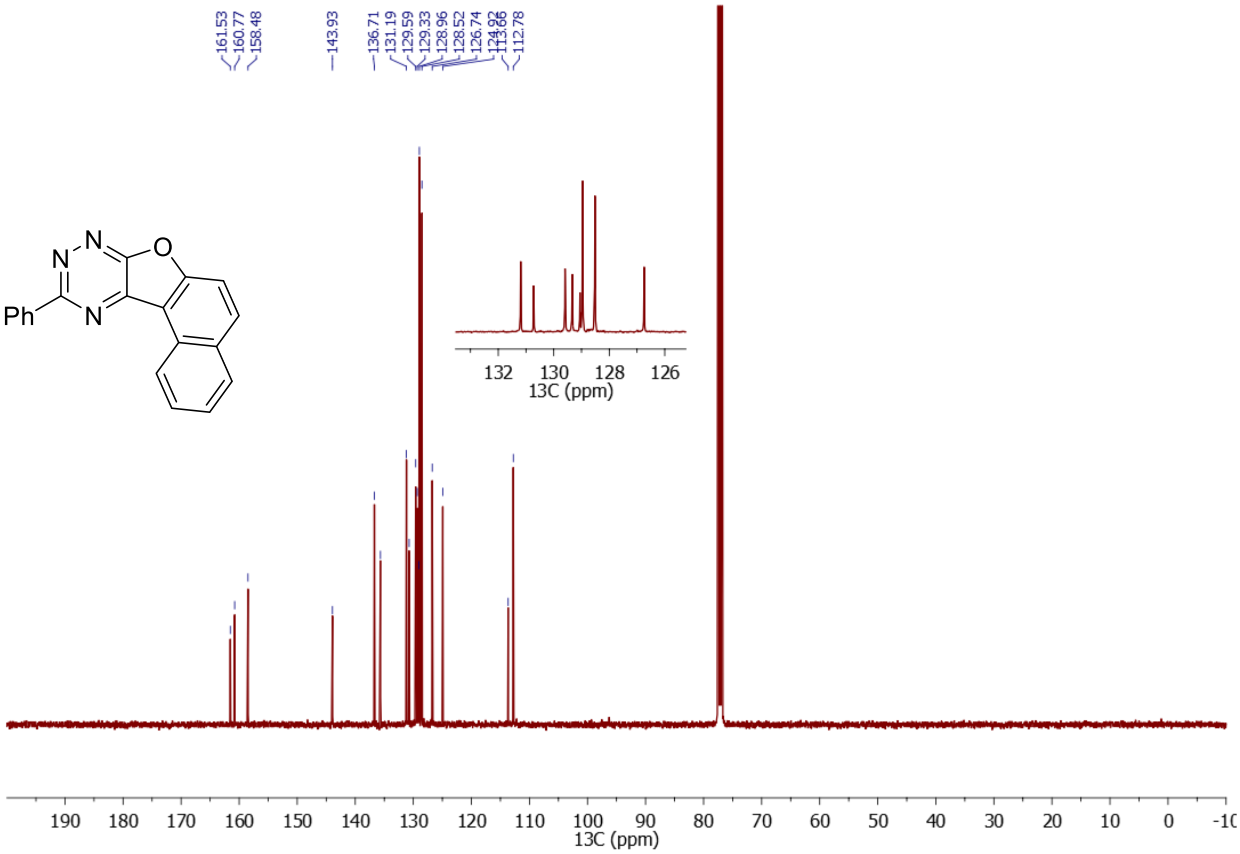

^{13}C NMR spectrum of 10-phenylnaphtho[1',2':4,5]furo[3,2-*e*][1,2,4]triazine **4ia**

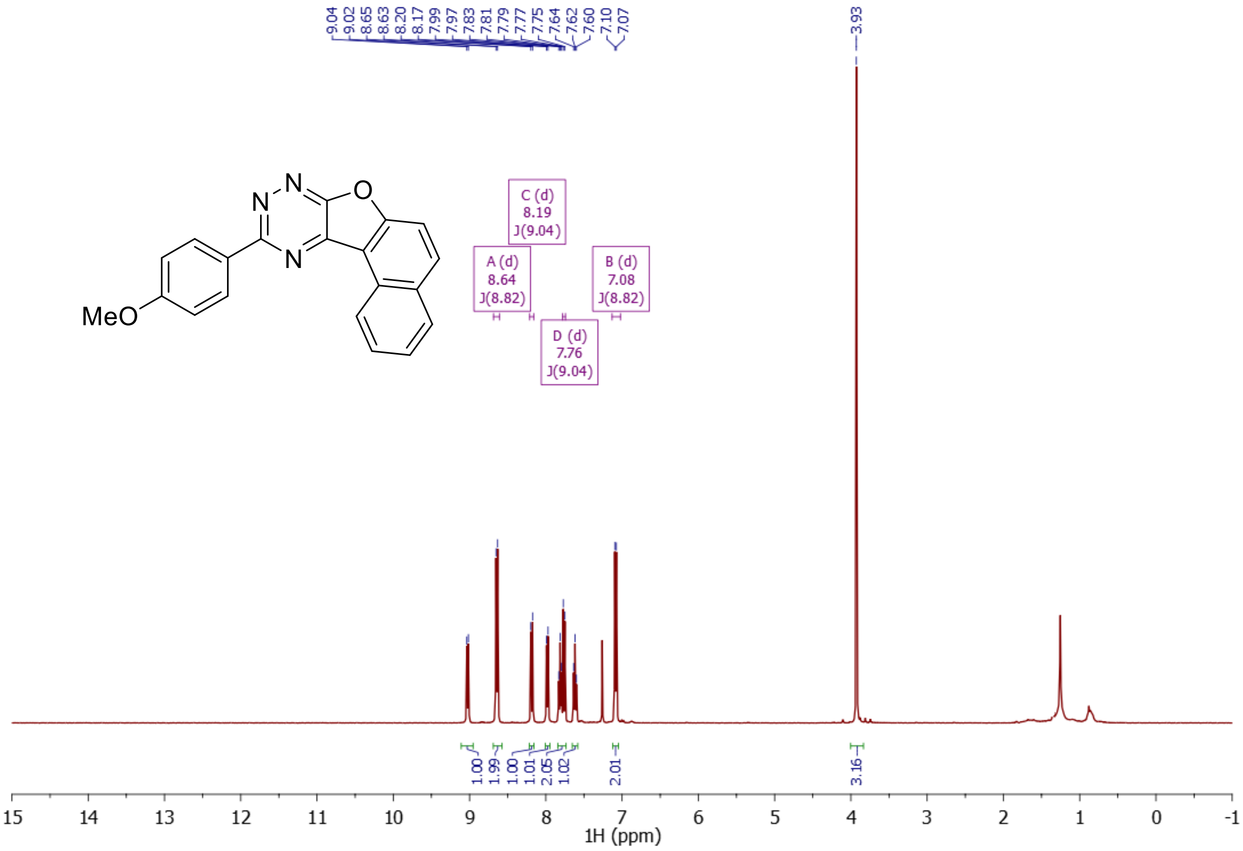

^{1}H NMR spectrum of 10-(4-methoxyphenyl)naphtho[1',2':4,5]furo[3,2-*e*][1,2,4]triazine **4ja**

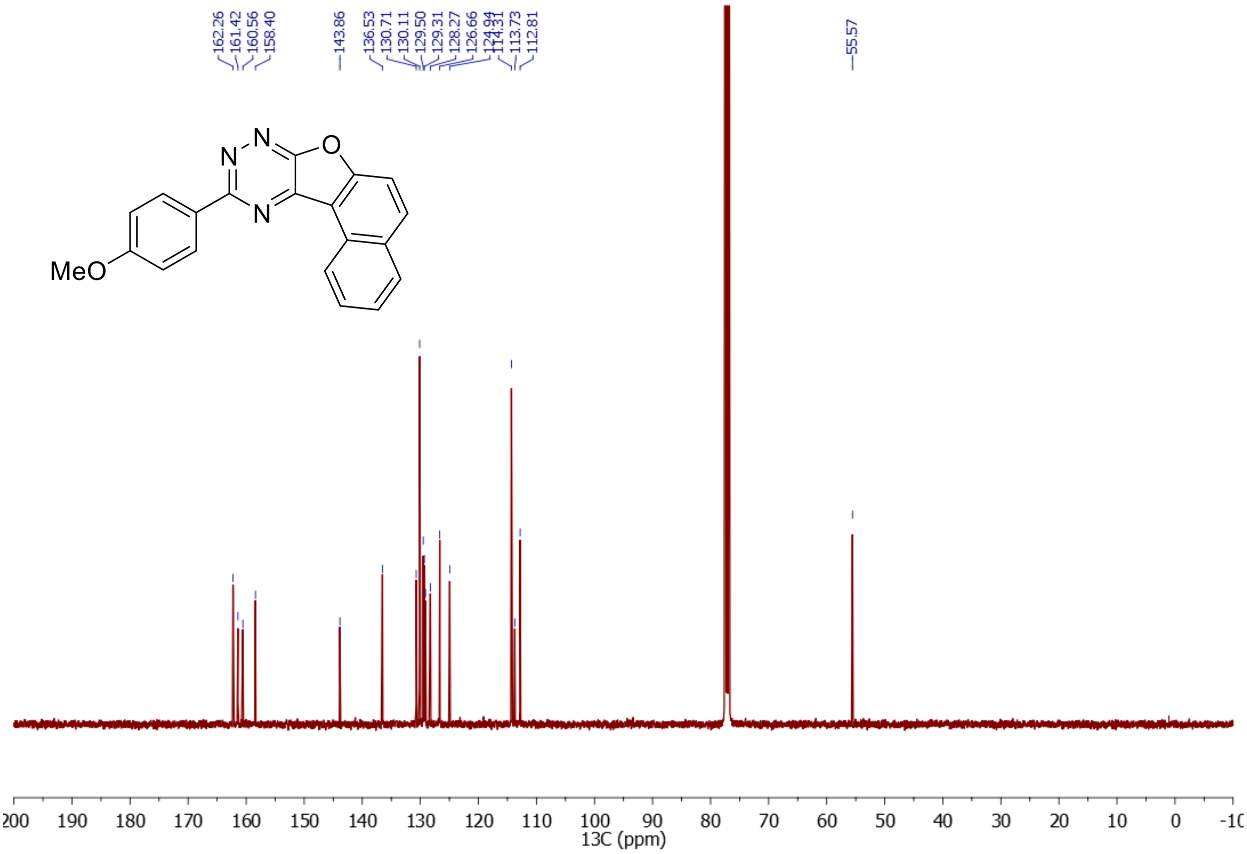

^{13}C NMR spectrum of 10-(4-methoxyphenyl)naphtho[1',2':4,5]furo[3,2-*e*][1,2,4]triazine **4ja**

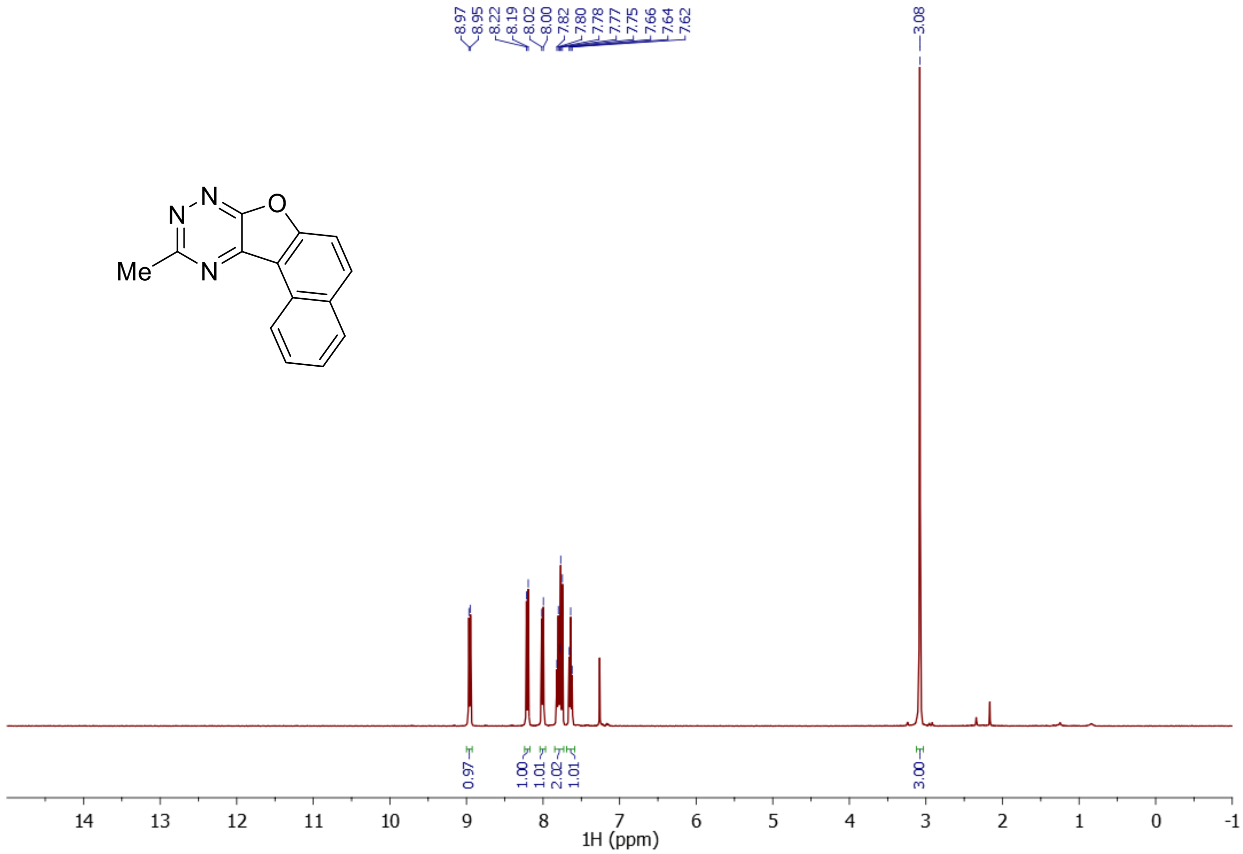

^{1}H NMR spectrum of 10-methylnaphtho[1',2':4,5]furo[3,2-*e*][1,2,4]triazine **4ka**

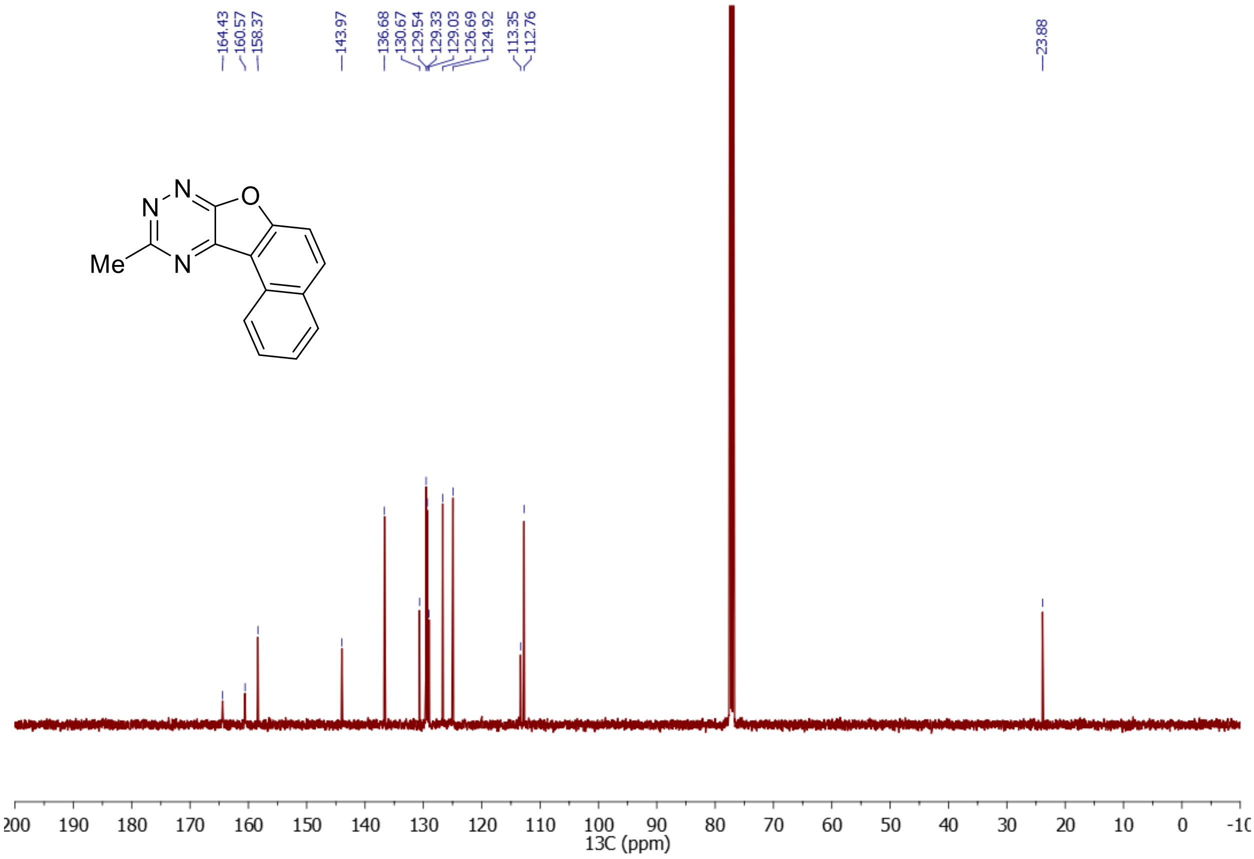

^{13}C NMR spectrum of 10-methylnaphtho[1',2':4,5]furo[3,2-*e*][1,2,4]triazine **4ka**

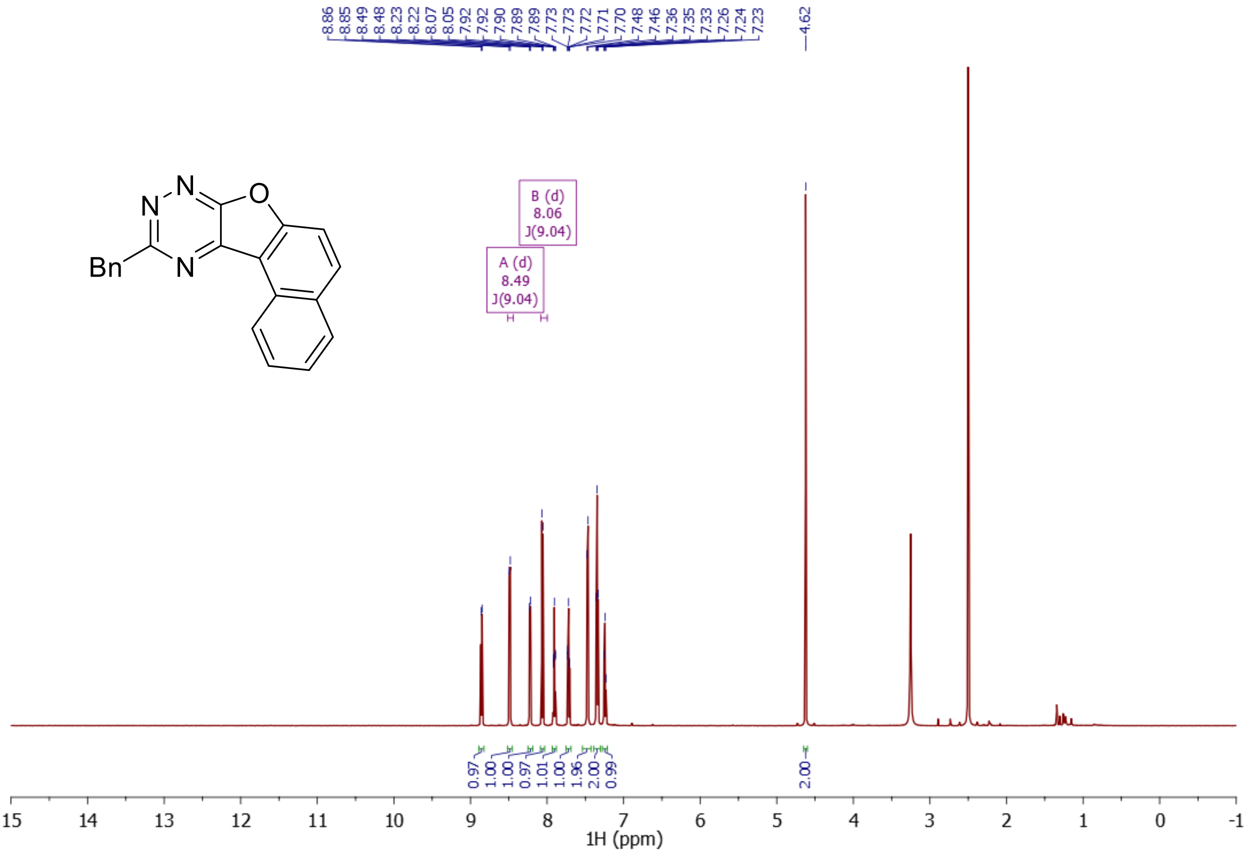

^{1}H NMR spectrum of 10-benzylnaphtho[1',2':4,5]furo[3,2-*e*][1,2,4]triazine **4la**

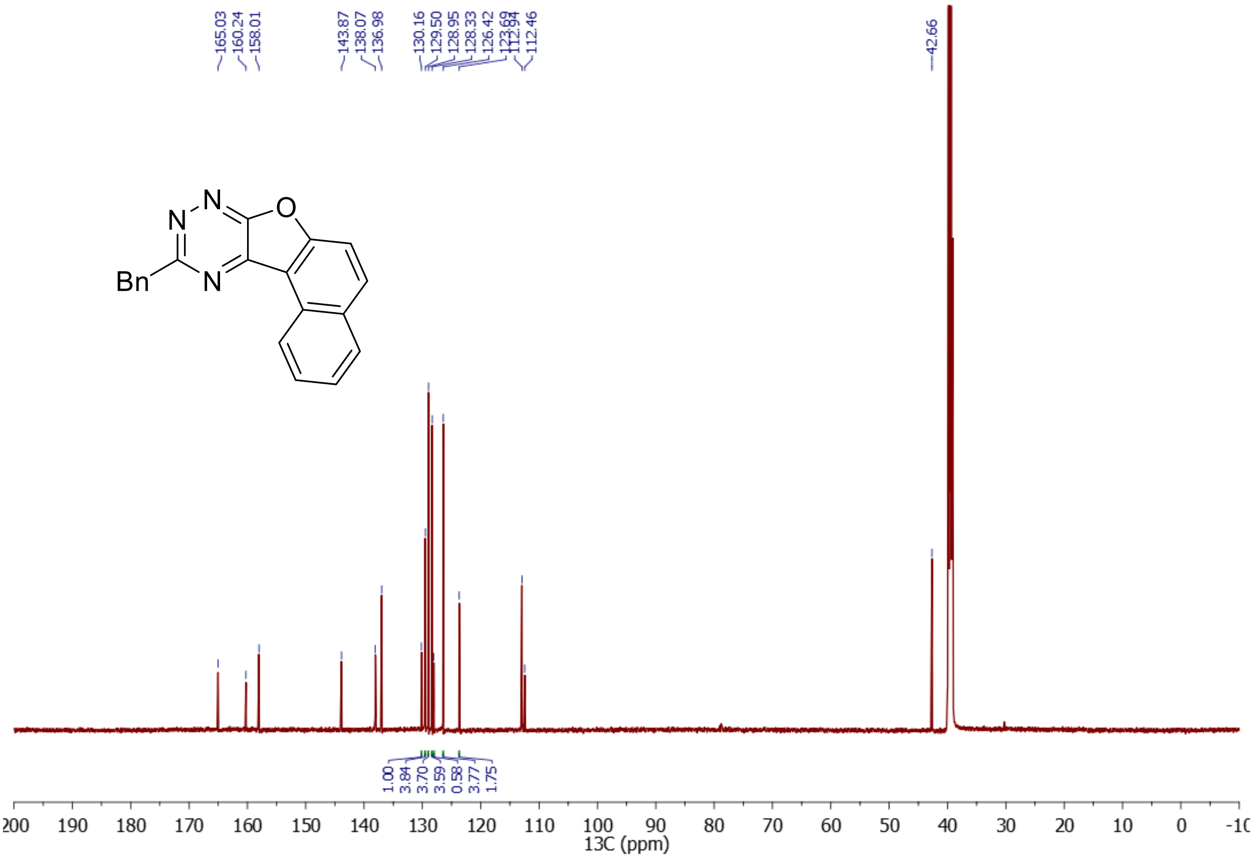

^{13}C NMR spectrum of 10-benzylnaphtho[1',2':4,5]furo[3,2-*e*][1,2,4]triazine **4la**

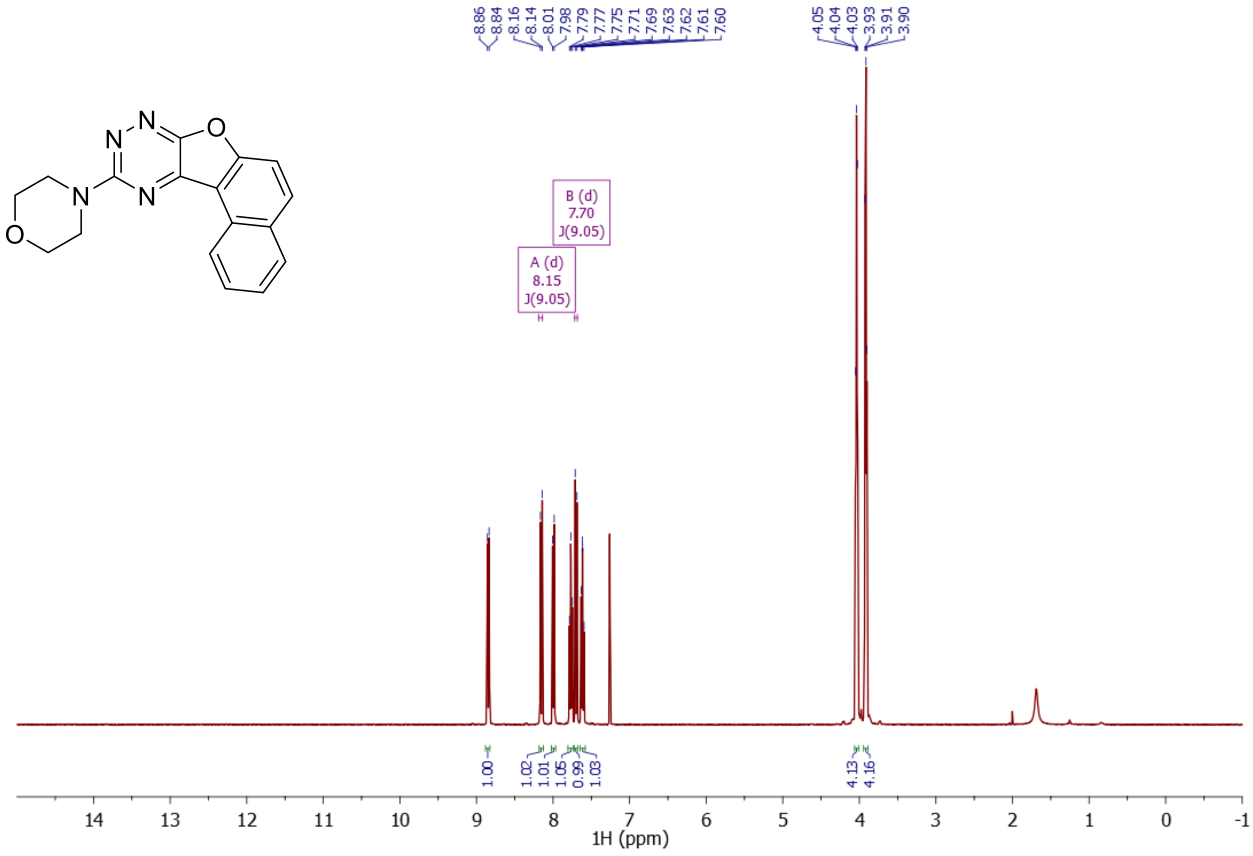

^{1}H NMR spectrum of 10-morpholinonaphtho[1',2':4,5]furo[3,2-*e*][1,2,4]triazine **4ma**

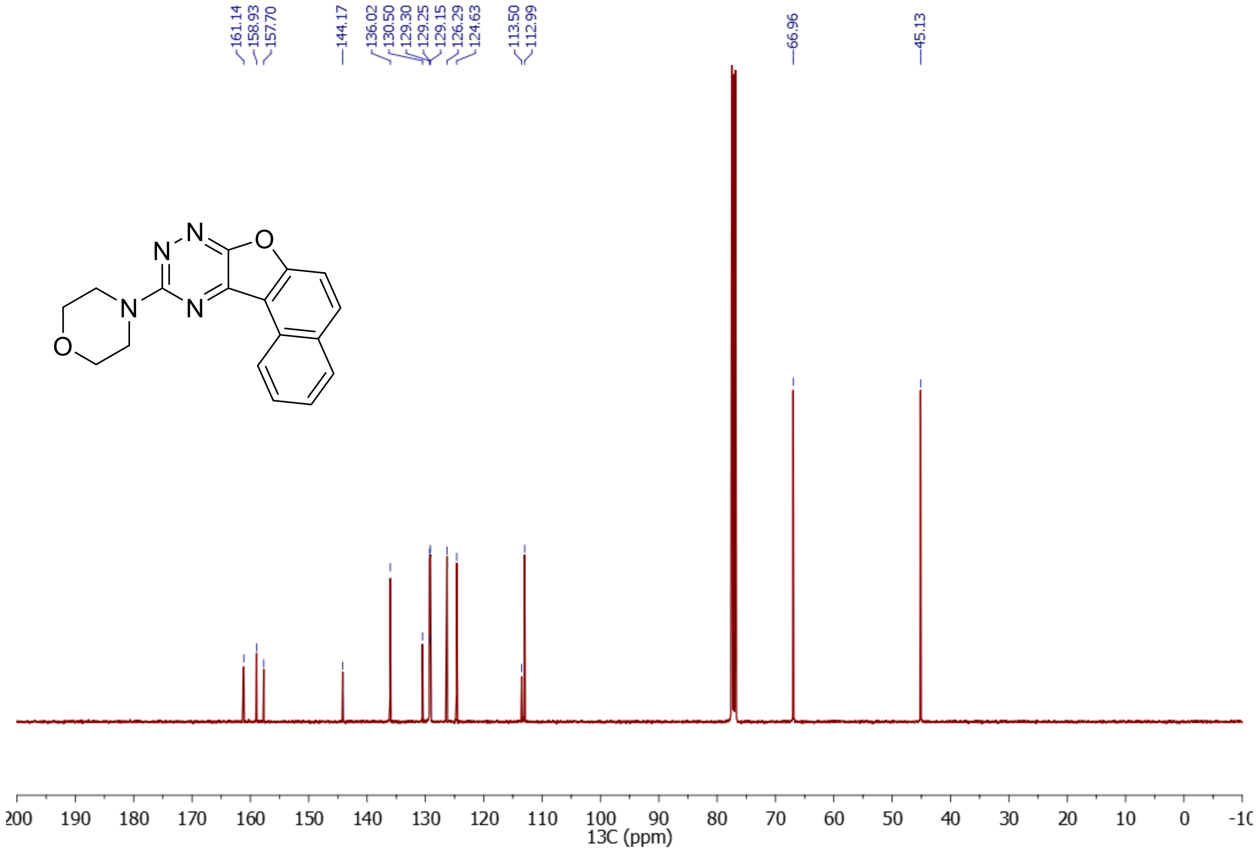

^{13}C NMR spectrum of 10-morpholinonaphtho[1',2':4,5]furo[3,2-*e*][1,2,4]triazine **4ma**

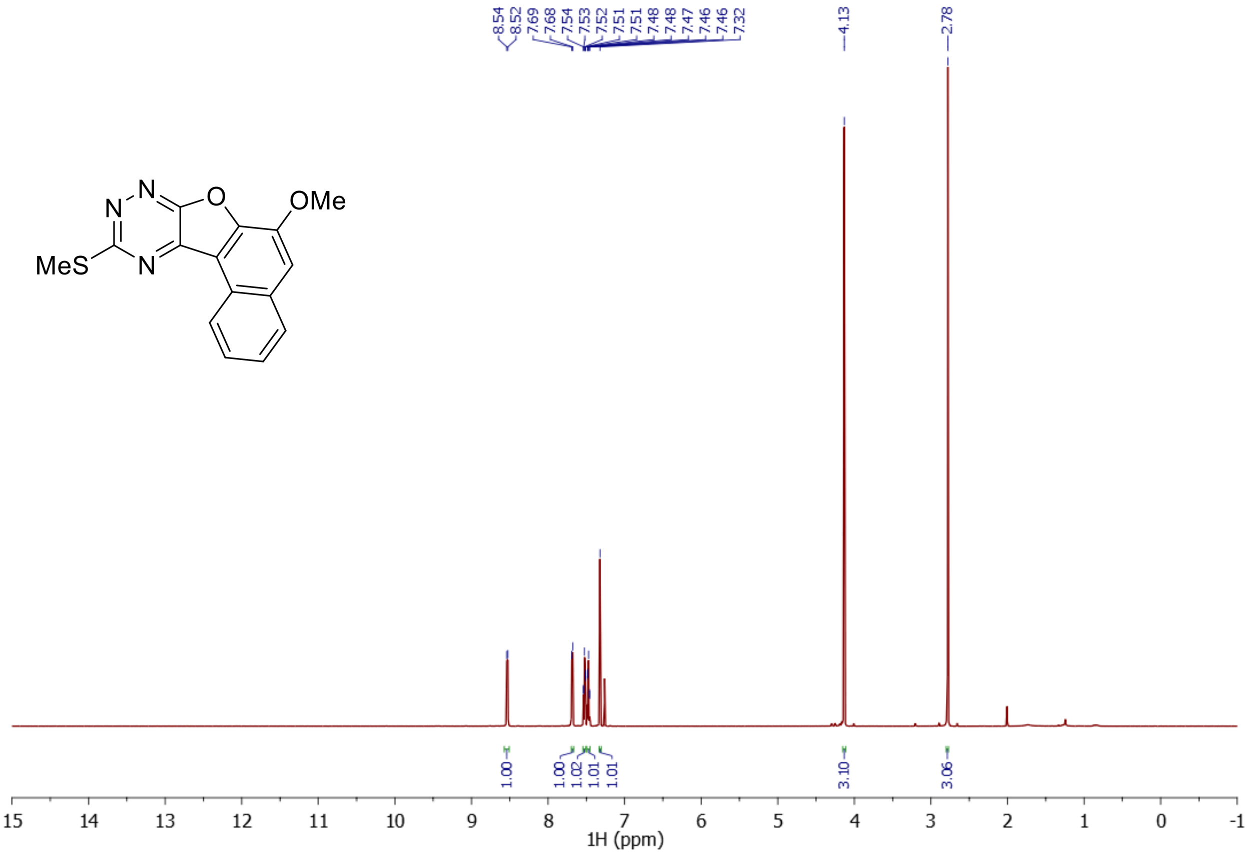

^{1}H NMR spectrum of 6-methoxy-10-(methylthio)naphtho[1',2':4,5]furo[3,2-*e*][1,2,4]triazine **4ab**

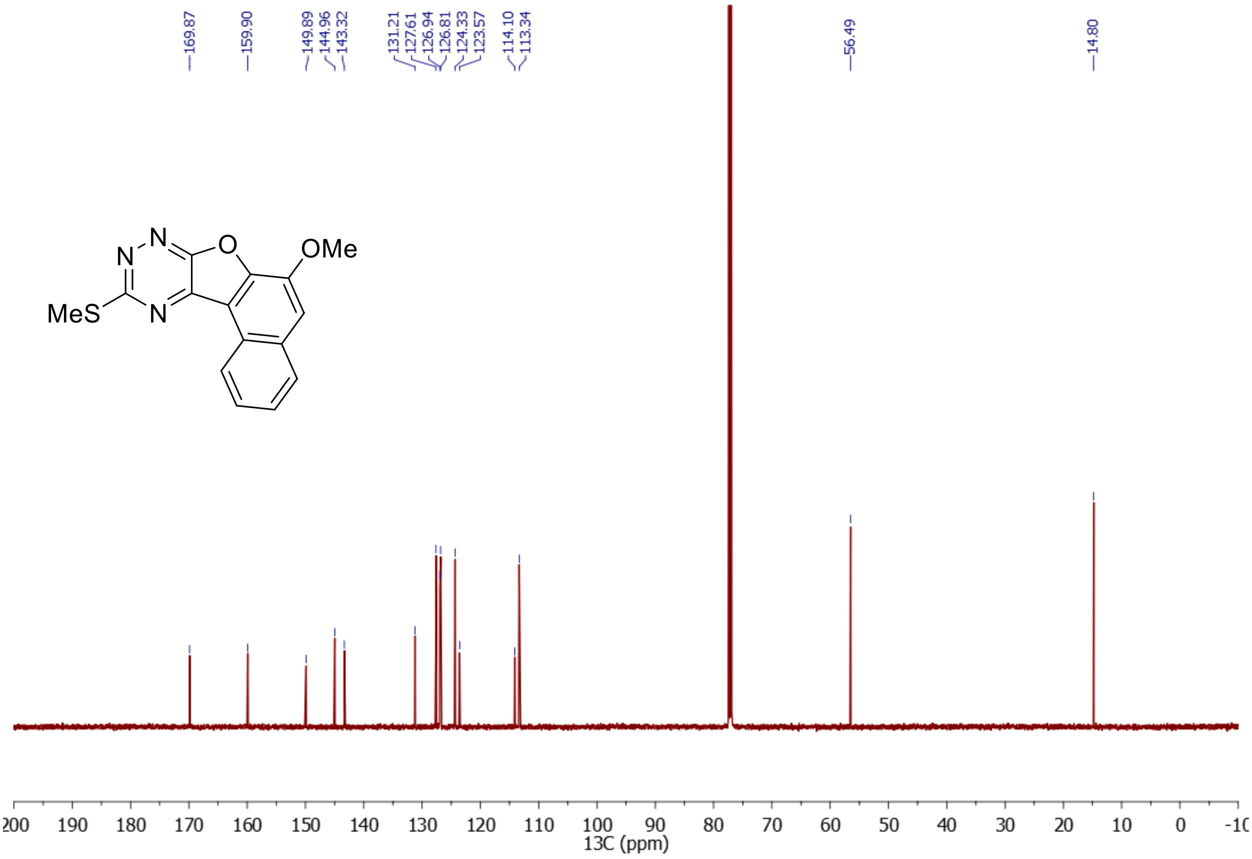

^{13}C NMR spectrum of 6-methoxy-10-(methylthio)naphtho[1',2':4,5]furo[3,2-*e*][1,2,4]triazine **4ab**

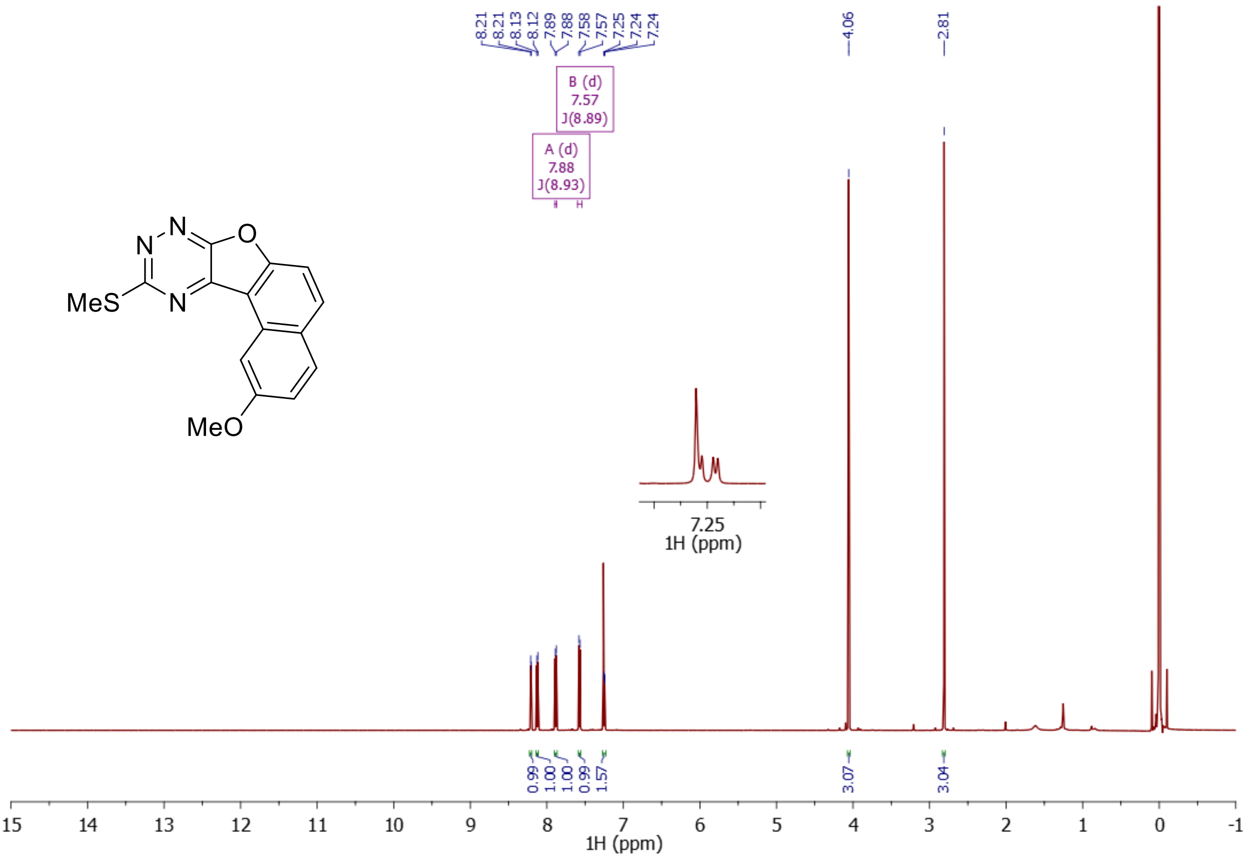

^{1}H NMR spectrum of 2-methoxy-10-(methylthio)naphtho[1',2':4,5]furo[3,2-*e*][1,2,4]triazine **4ac**

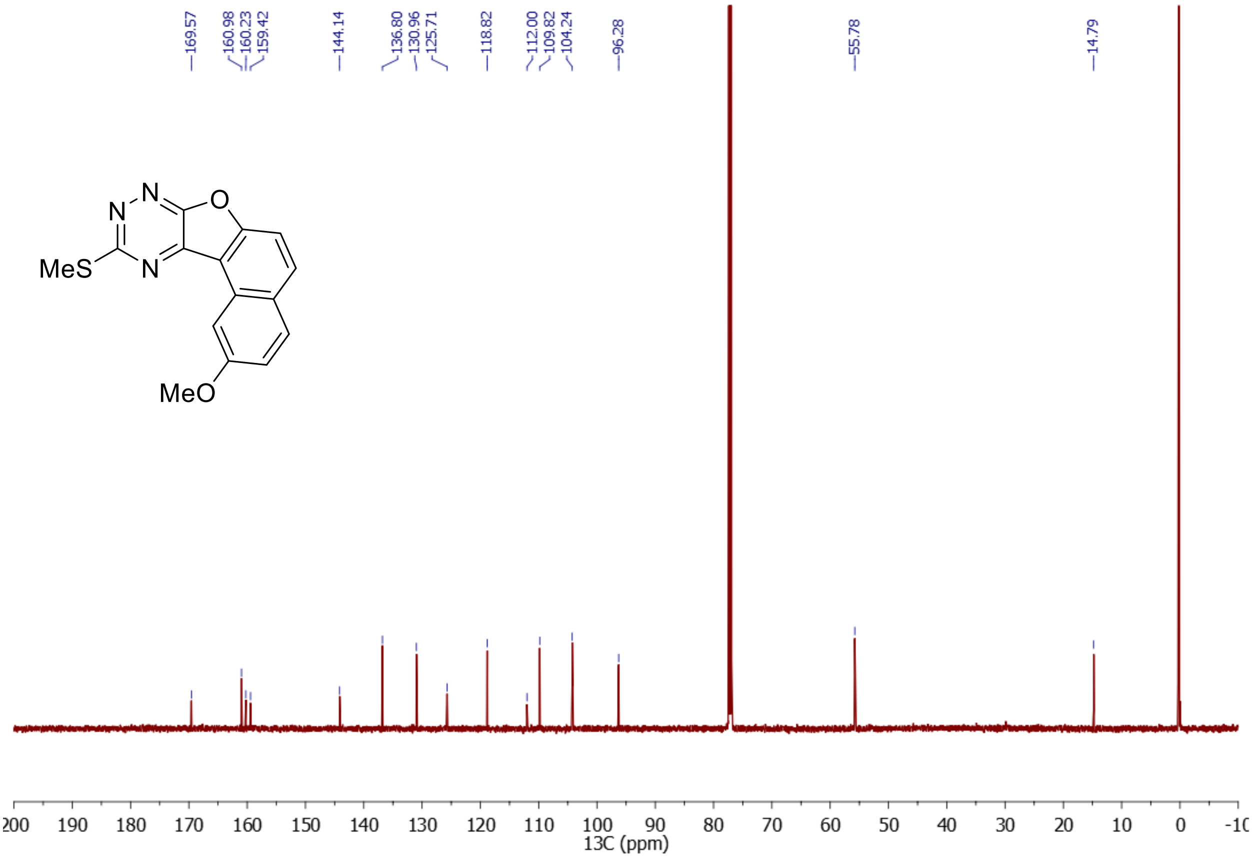

^{13}C NMR spectrum of 6-methoxy-10-(methylthio)naphtho[1',2':4,5]furo[3,2-*e*][1,2,4]triazine **4ac**

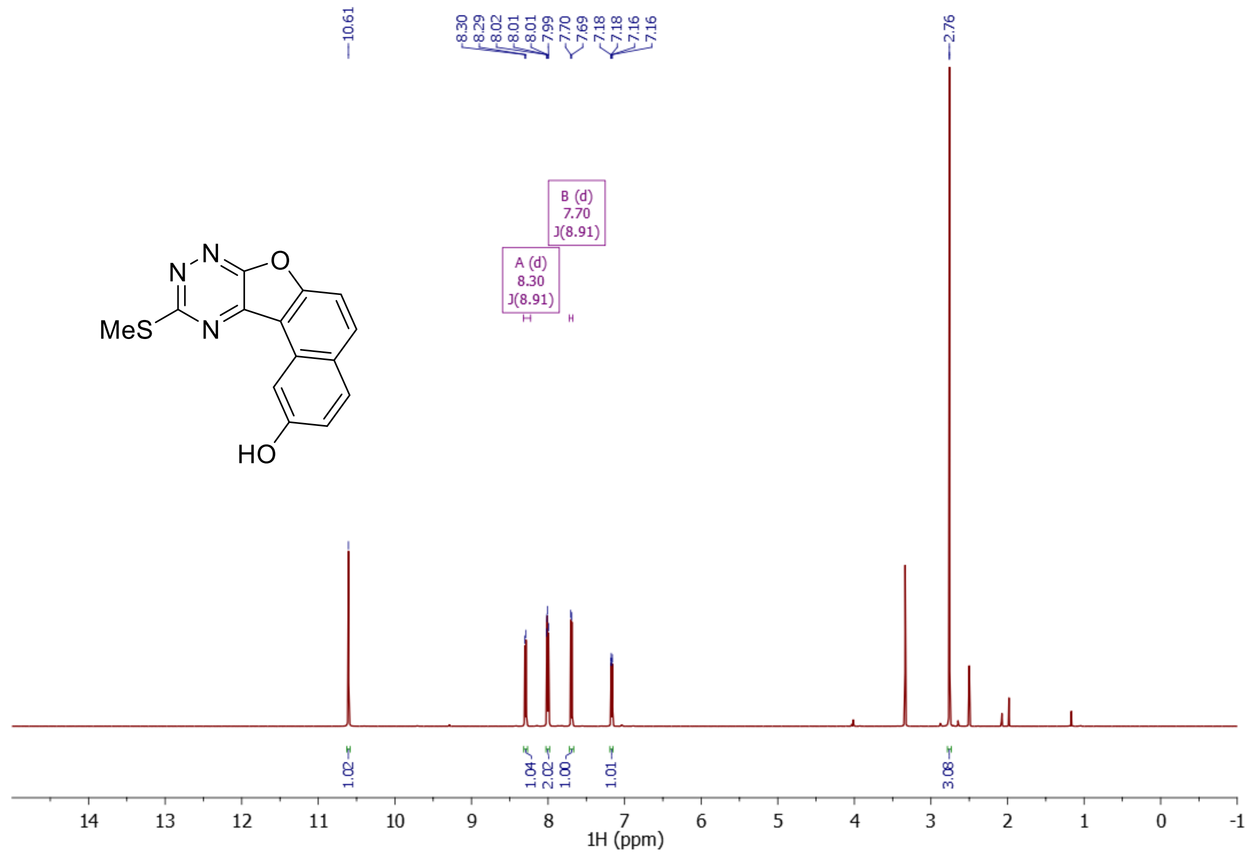

^{1}H NMR spectrum of 10-(methylthio)naphtho[1',2':4,5]furo[3,2-*e*][1,2,4]triazin-2-ol **4ad**

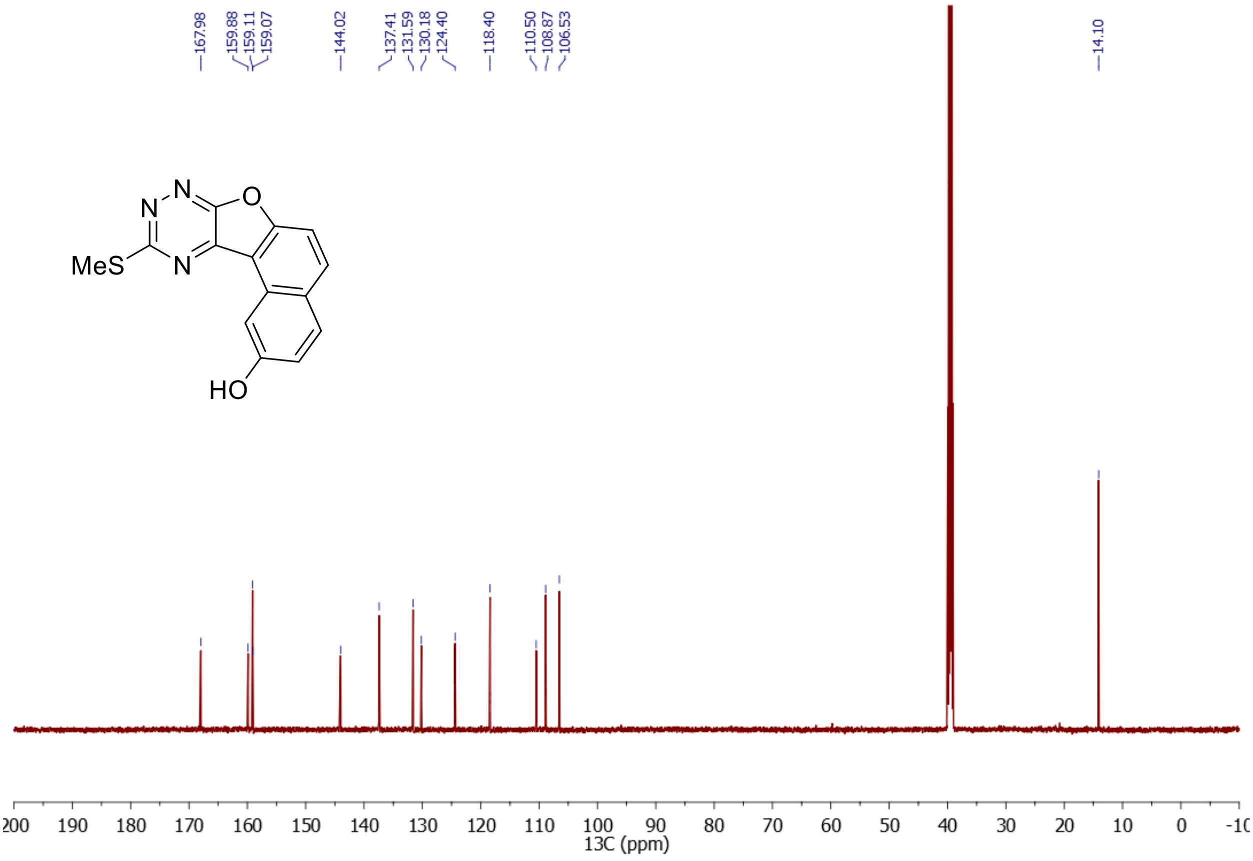

^{13}C NMR spectrum of 10-(methylthio)naphtho[1',2':4,5]furo[3,2-*e*][1,2,4]triazin-2-ol **4ad**

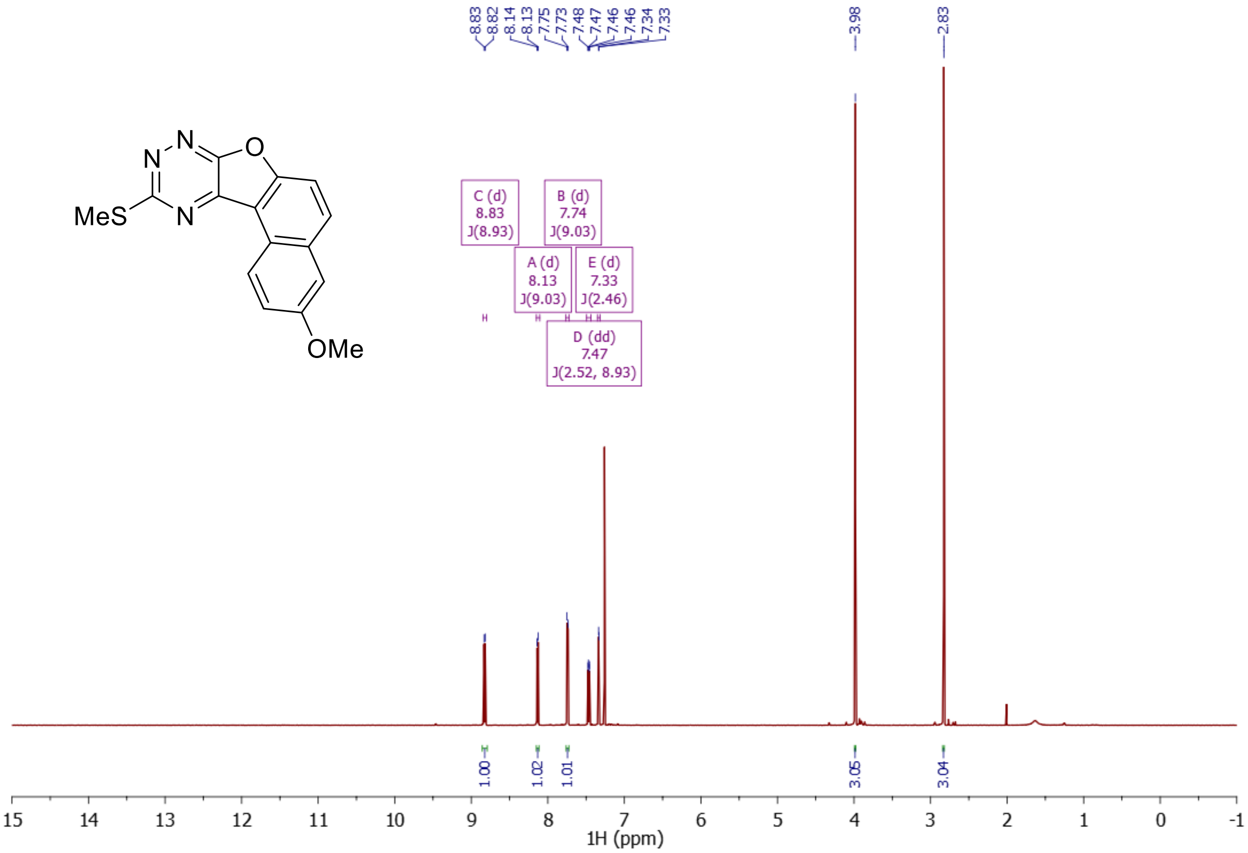

^{1}H NMR spectrum of 3-methoxy-10-(methylthio)naphtho[1',2':4,5]furo[3,2-*e*][1,2,4]triazine **4ae**

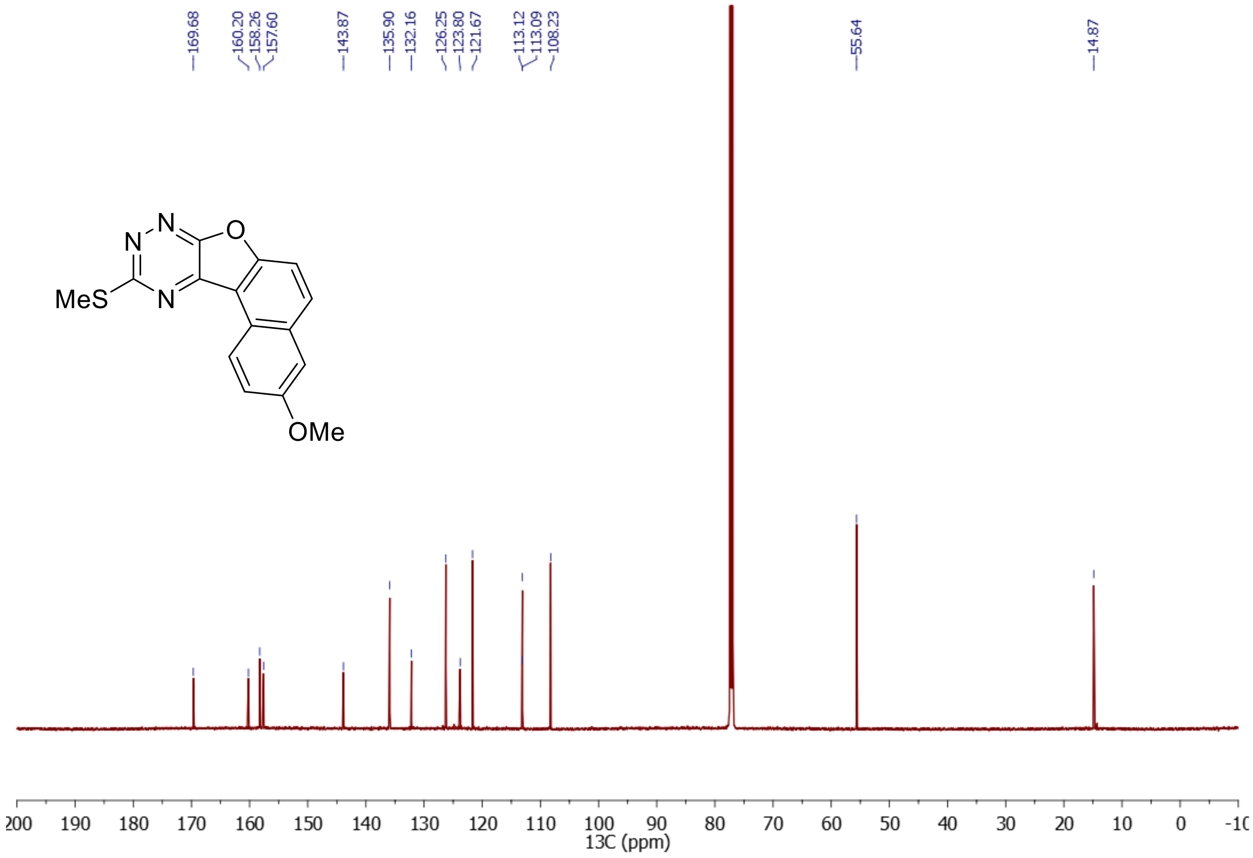

^{13}C NMR spectrum of 6-methoxy-10-(methylthio)naphtho[1',2':4,5]furo[3,2-*e*][1,2,4]triazine **4ae**

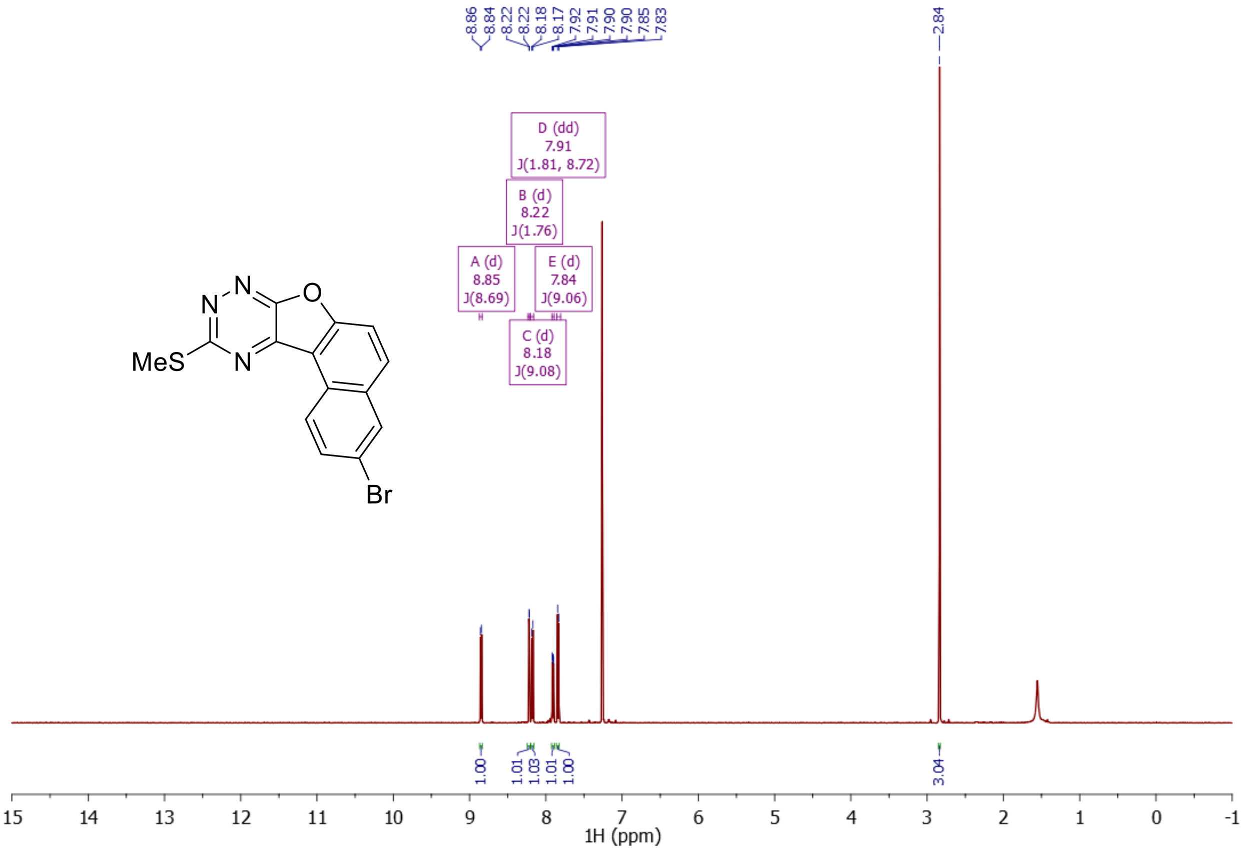

^{1}H NMR spectrum of 3-bromo-10-(methylthio)naphtho[1',2':4,5]furo[3,2-*e*][1,2,4]triazine **4af**

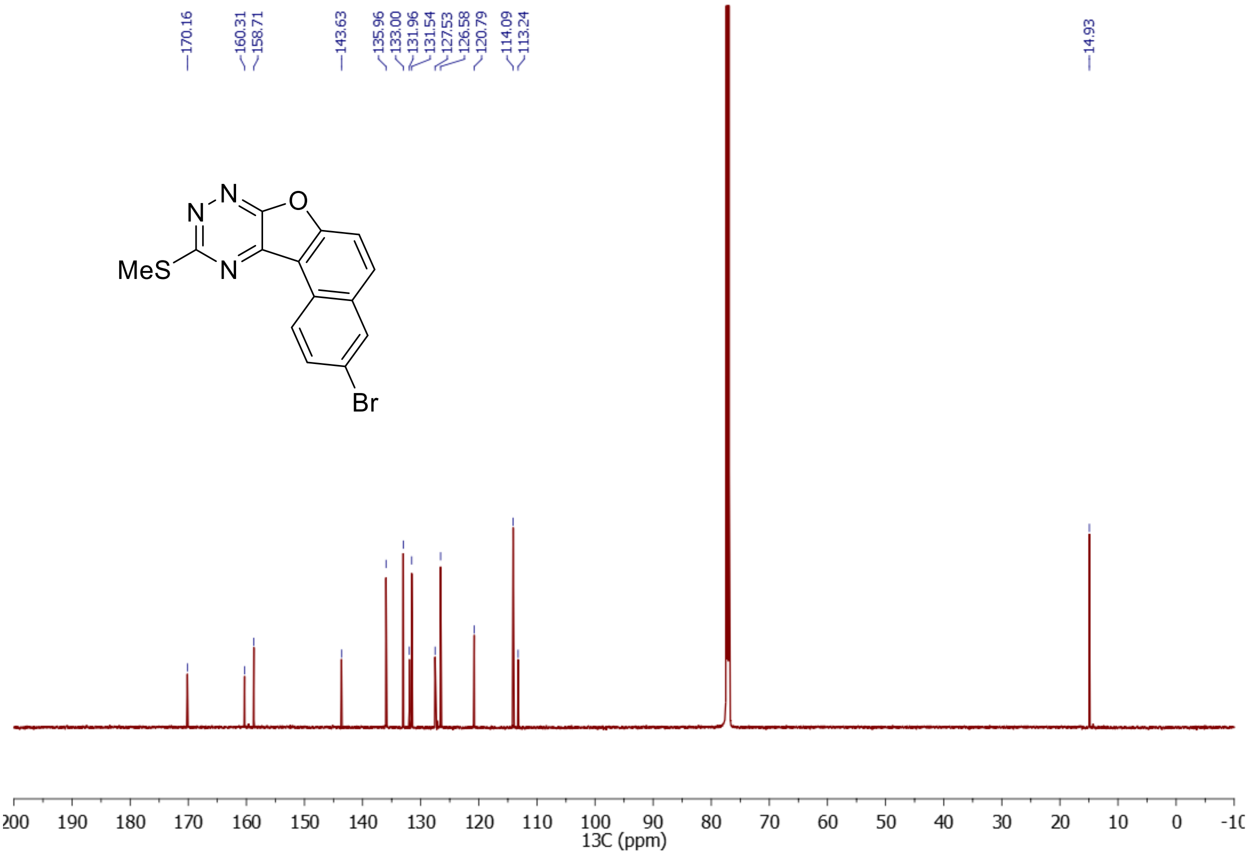

^{1}H NMR spectrum of 3-bromo-10-(methylthio)naphtho[1',2':4,5]furo[3,2-*e*][1,2,4]triazine **4af**

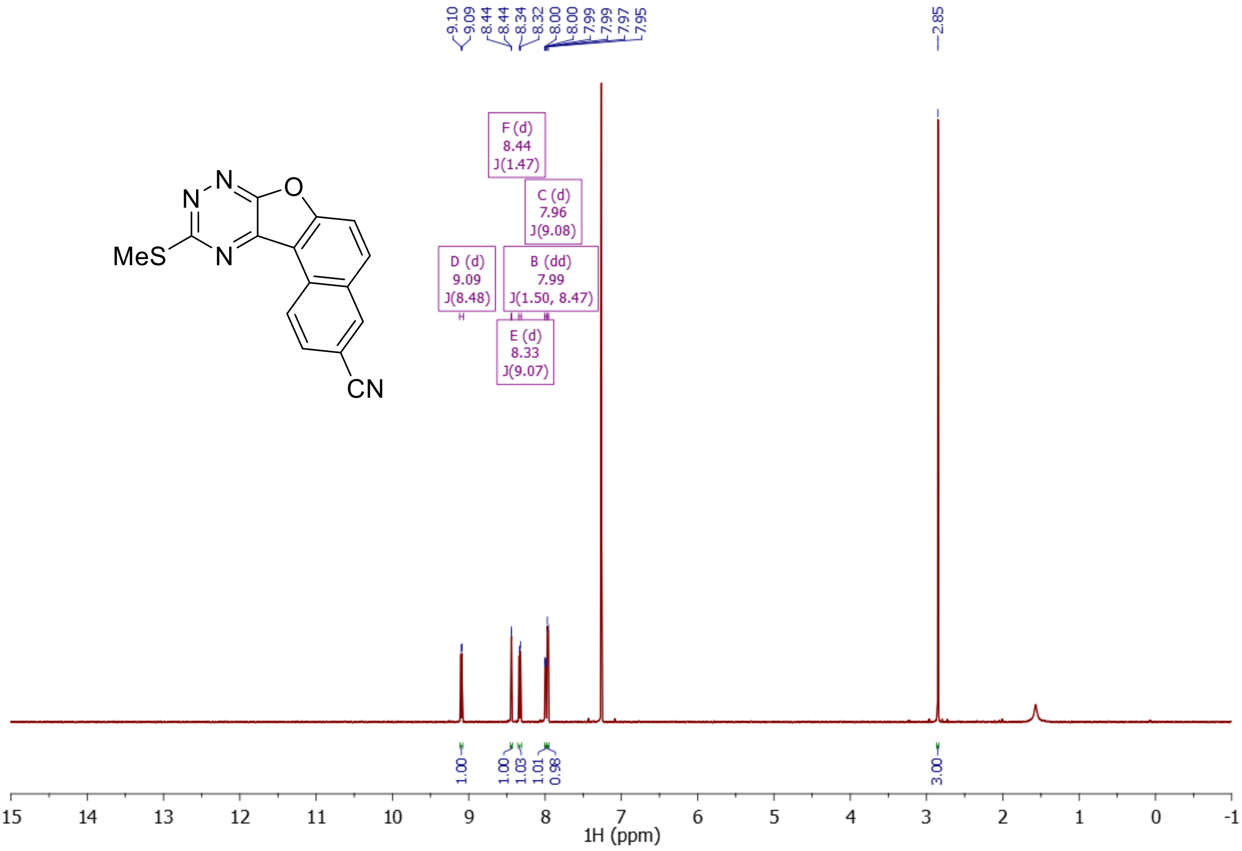

^{1}H NMR spectrum of 10-(methylthio)naphtho[1',2':4,5]furo[3,2-*e*][1,2,4]triazine-3-carbonitrile **4ag**

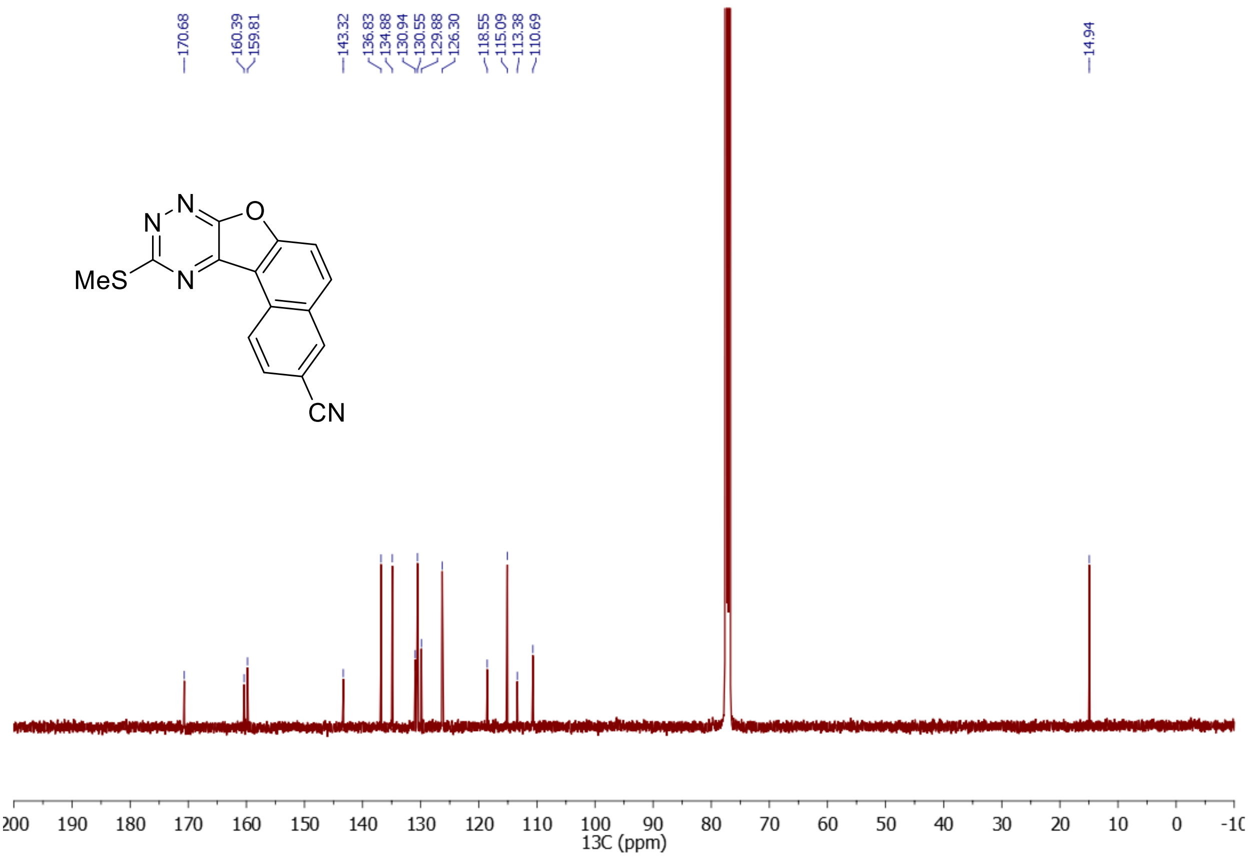

^{13}C NMR spectrum of 10-(methylthio)naphtho[1',2':4,5]furo[3,2-*e*][1,2,4]triazine-3-carbonitrile **4ag**

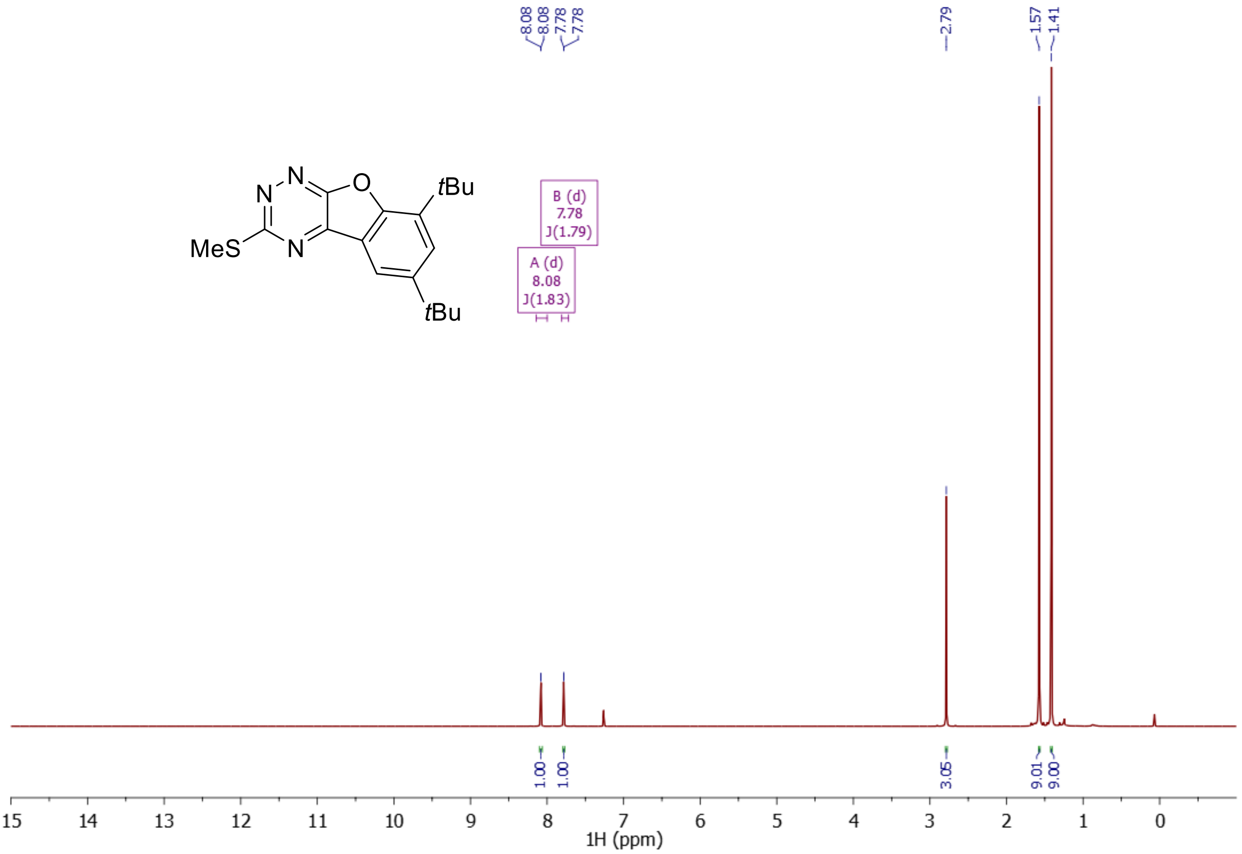

^{1}H NMR spectrum of 6,8-di-*tert*-butyl-3-(methylthio)benzofuro[3,2-*e*][1,2,4]triazine **4ah**

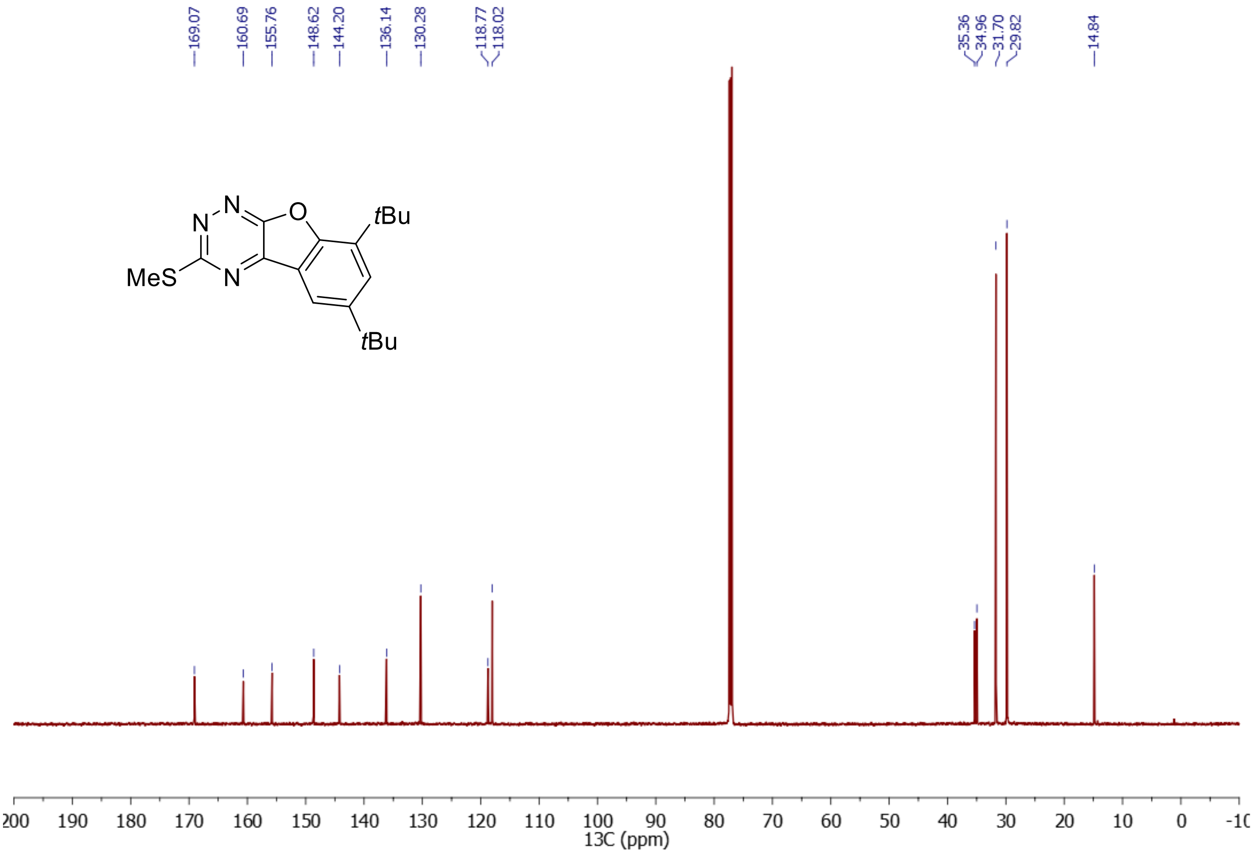

^{13}C NMR spectrum of 6,8-di-*tert*-butyl-3-(methylthio)benzofuro[3,2-*e*][1,2,4]triazine **4ah**

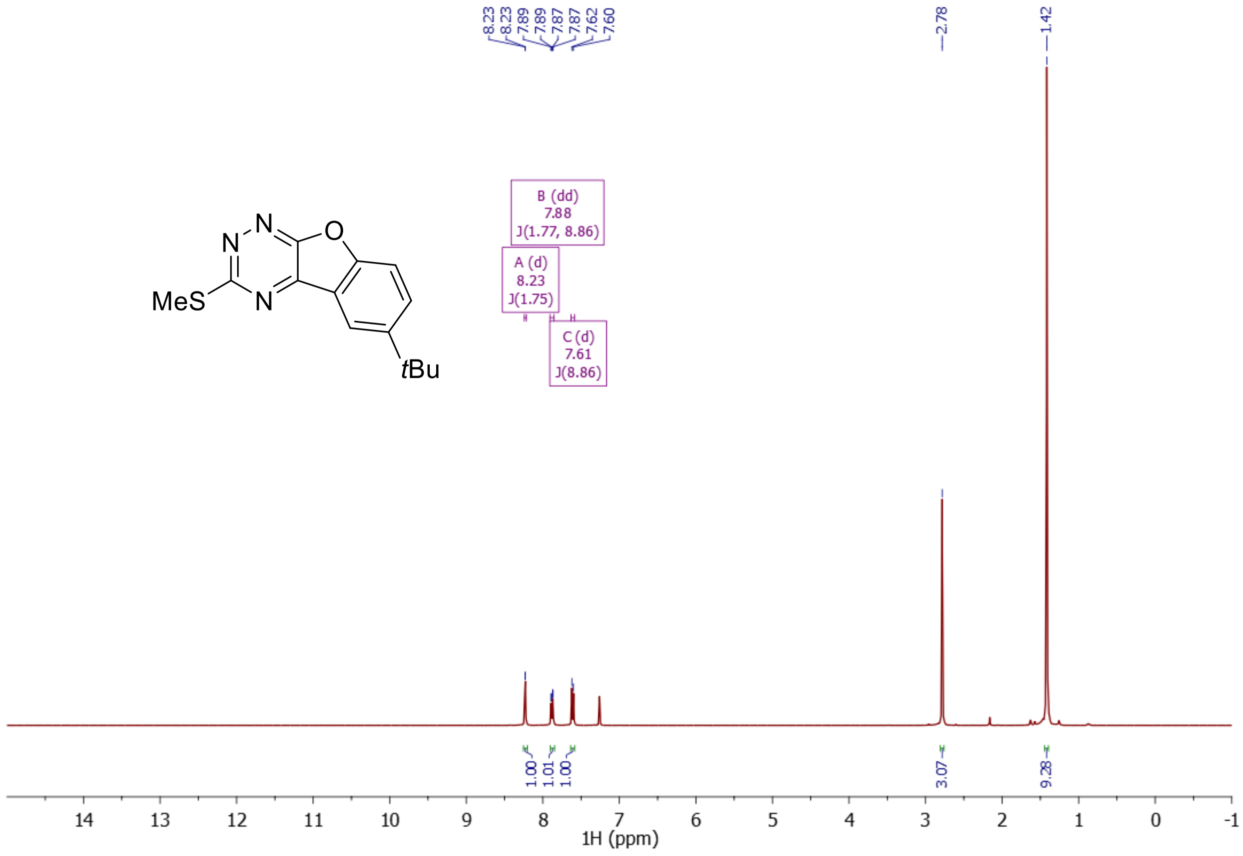

^{1}H NMR spectrum of 6-(*tert*-butyl)-3-(methylthio)benzofuro[3,2-*e*][1,2,4]triazine **4ai**

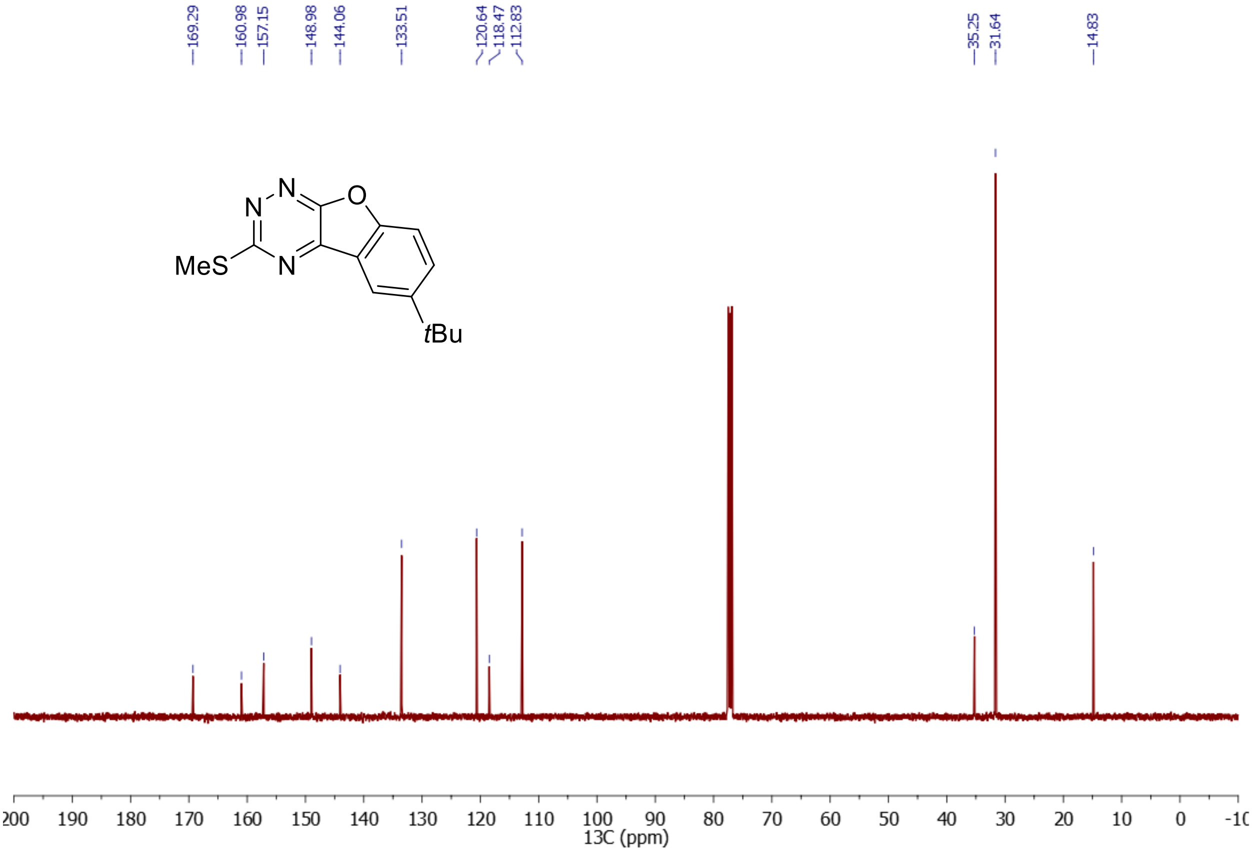

^{13}C NMR spectrum of 6-(*tert*-butyl)-3-(methylthio)benzofuro[3,2-*e*][1,2,4]triazine **4ai**

Copies of ^{1}H and ^{13}C NMR spectra for compounds **5**

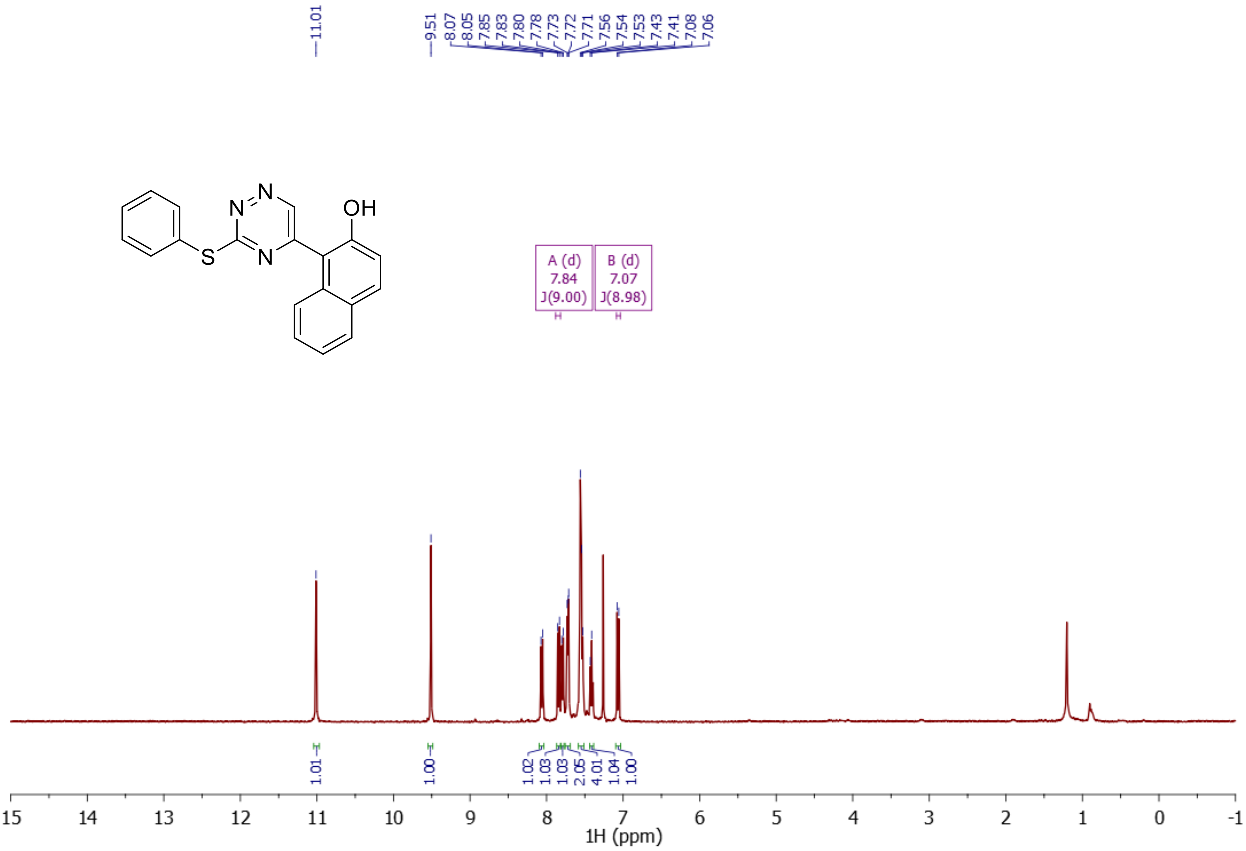

^{1}H NMR spectrum of 1-(3-(phenylthio)-1,2,4-triazin-5-yl)naphthalen-2-ol **5ha**

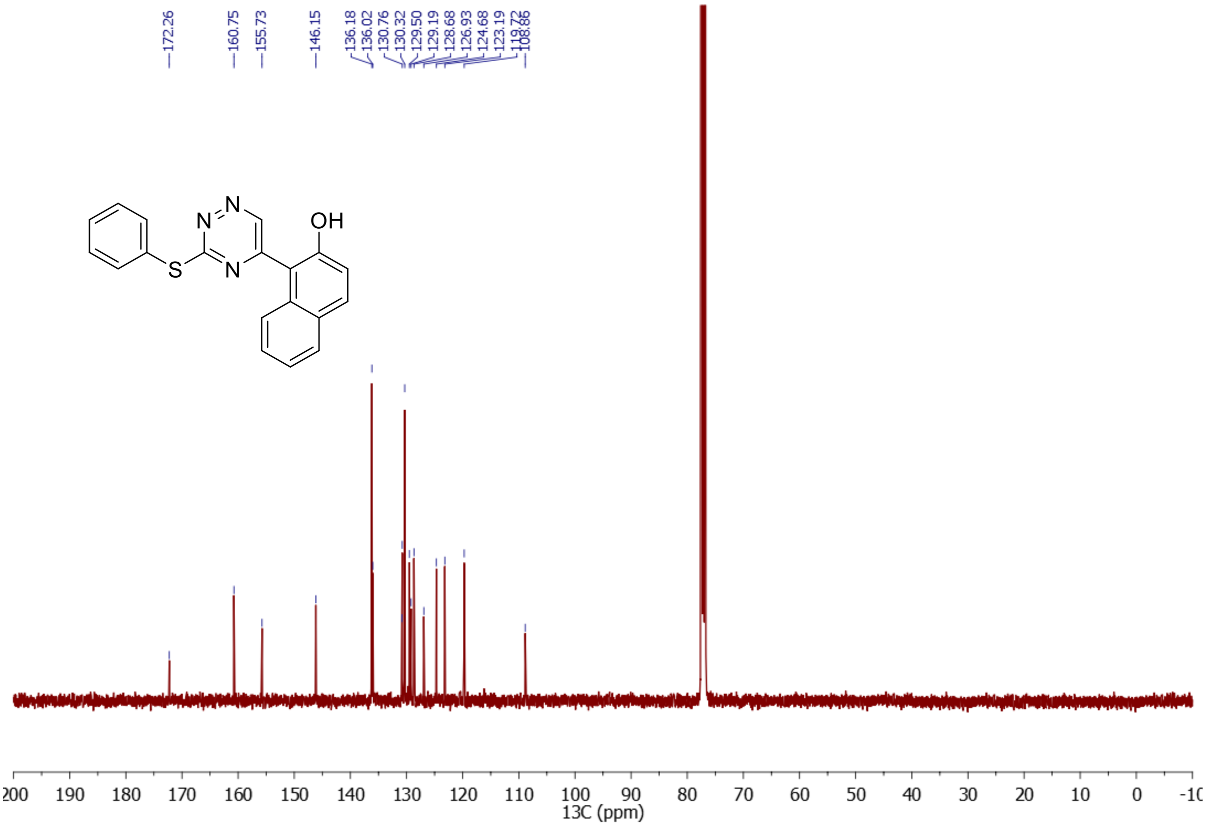

^{13}C NMR spectrum of 1-(3-(phenylthio)-1,2,4-triazin-5-yl)naphthalen-2-ol **5ha**

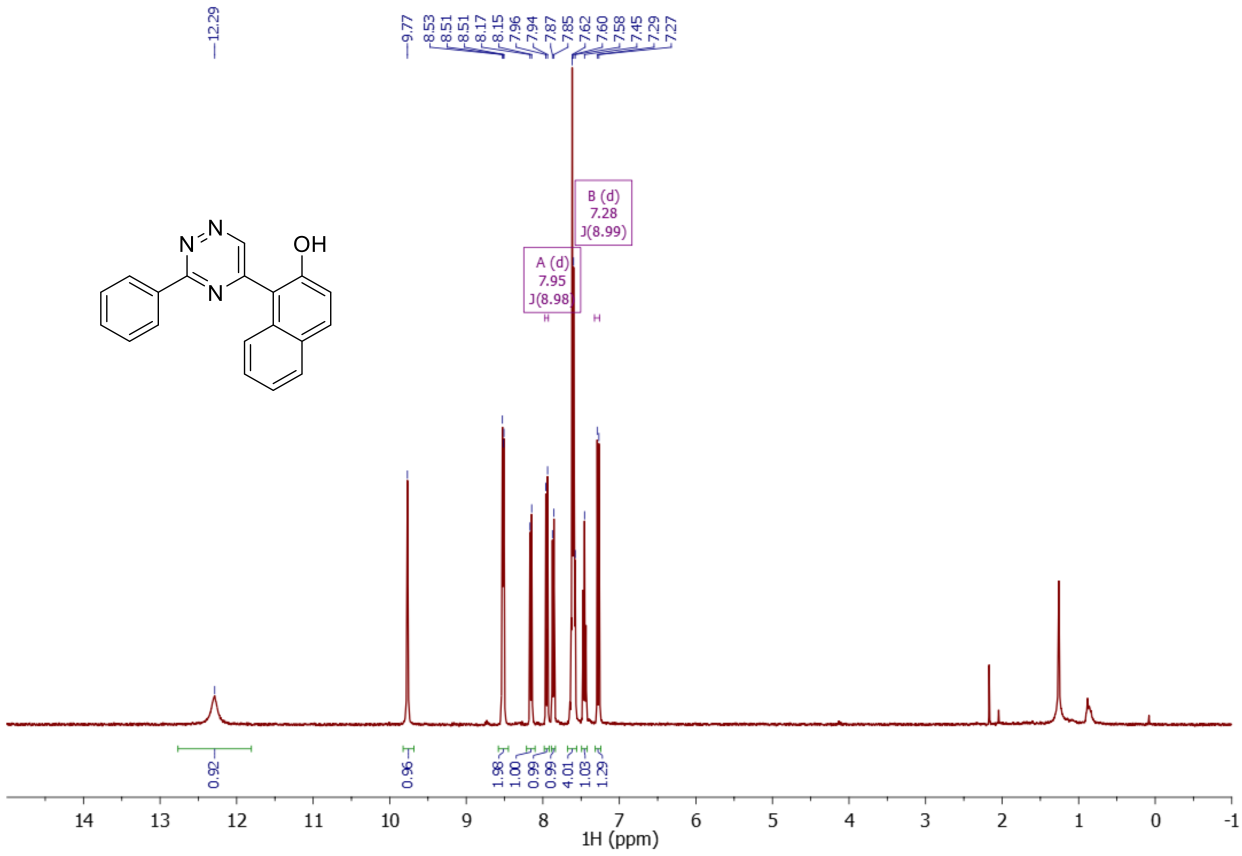

^{1}H NMR spectrum of 1-(3-phenyl-1,2,4-triazin-5-yl)naphthalen-2-ol **5ia**

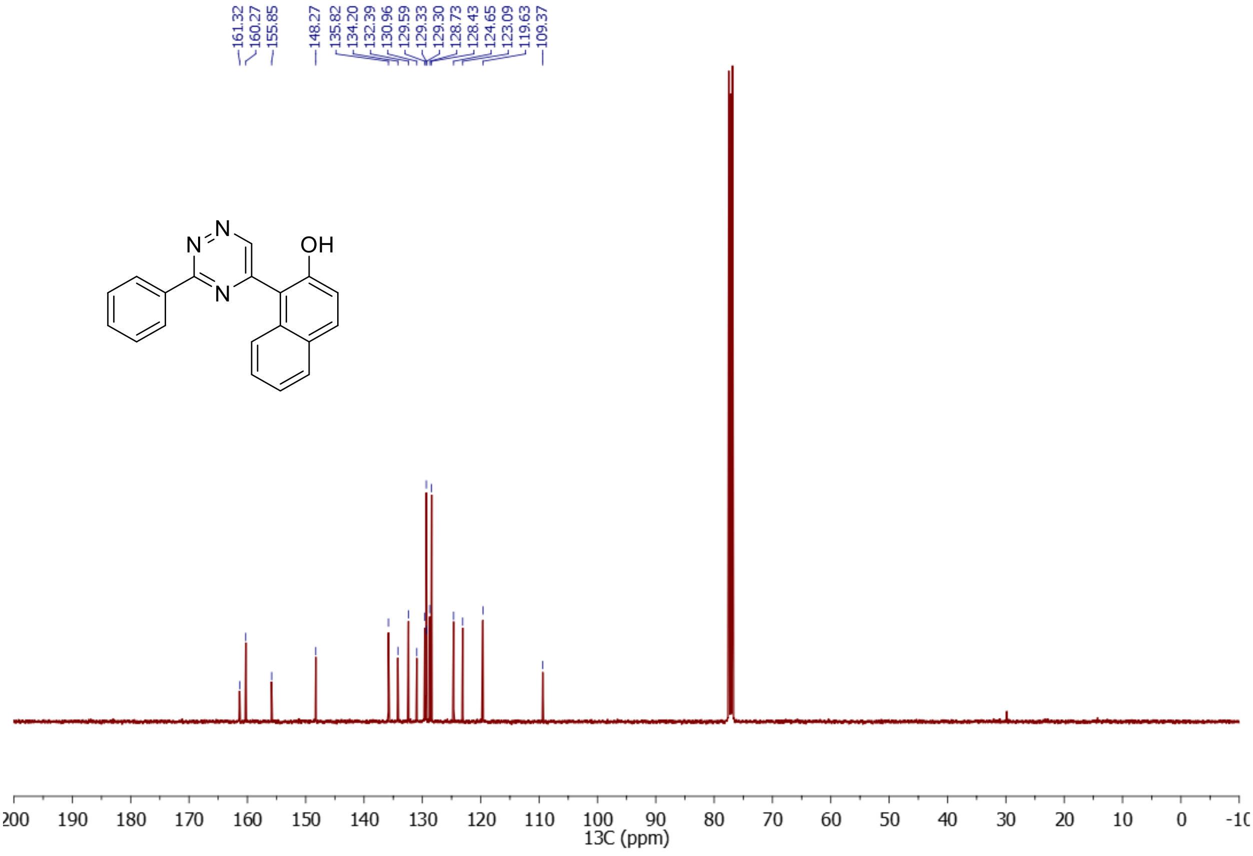

^{13}C NMR spectrum of 1-(3-phenyl-1,2,4-triazin-5-yl)naphthalen-2-ol **5ia**

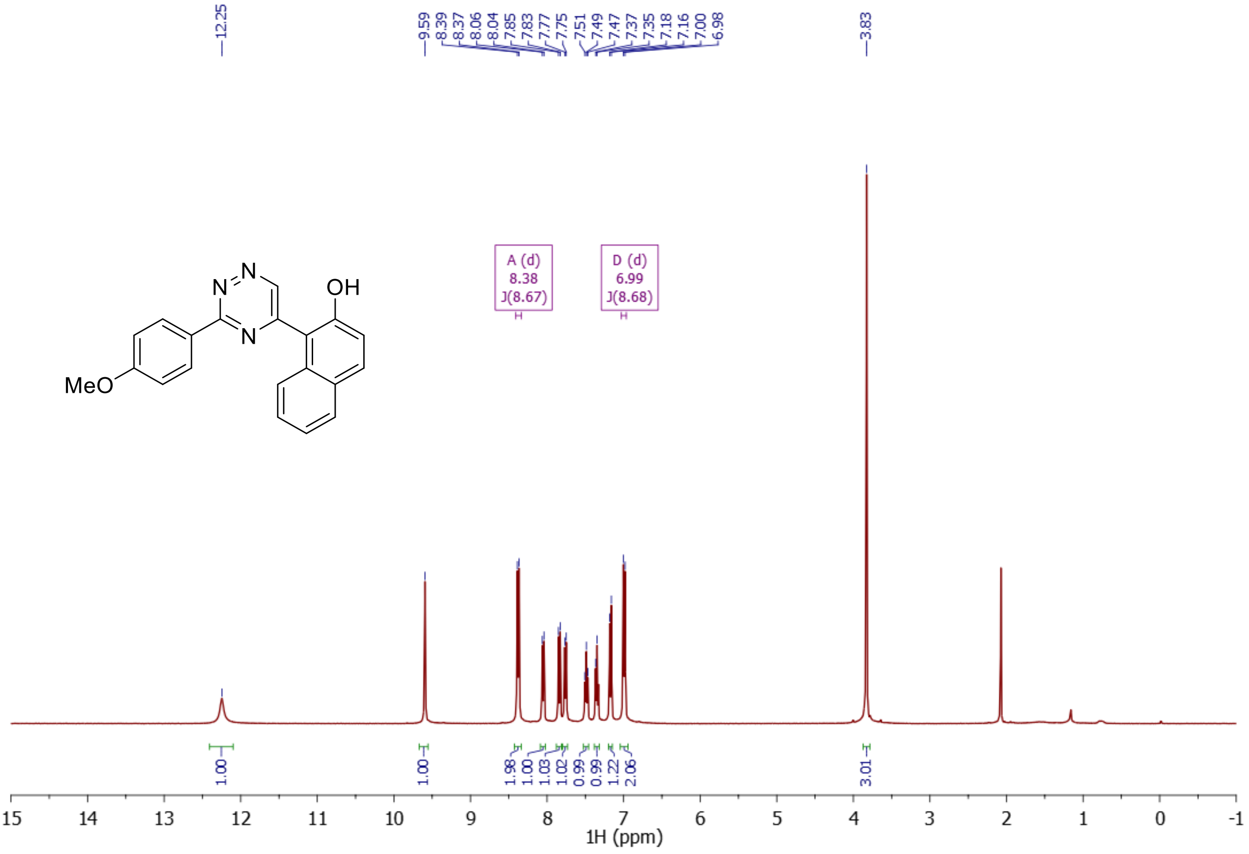

^{1}H NMR spectrum of 1-(3-(4-methoxyphenyl)-1,2,4-triazin-5-yl)naphthalen-2-ol **5ja**

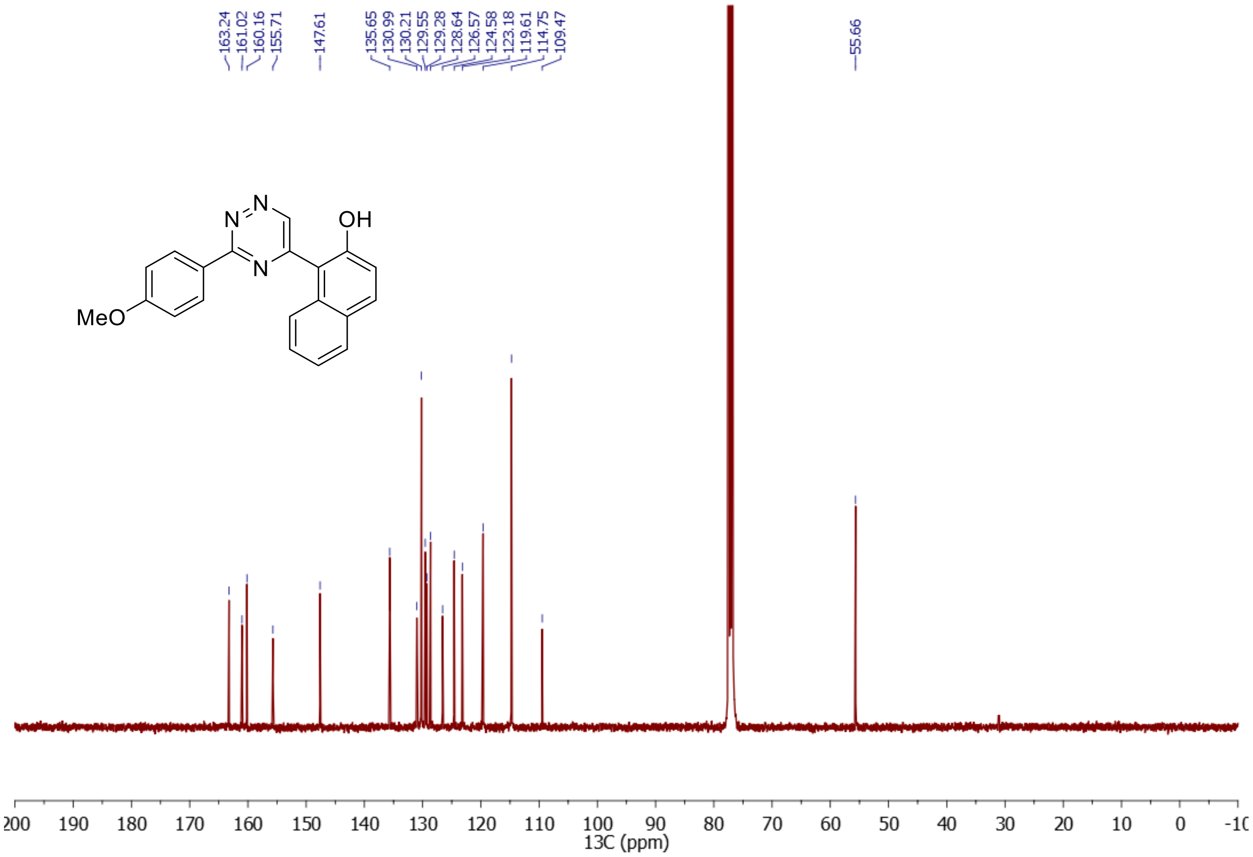

^{13}C NMR spectrum of 1-(3-(4-methoxyphenyl)-1,2,4-triazin-5-yl)naphthalen-2-ol **5ja**

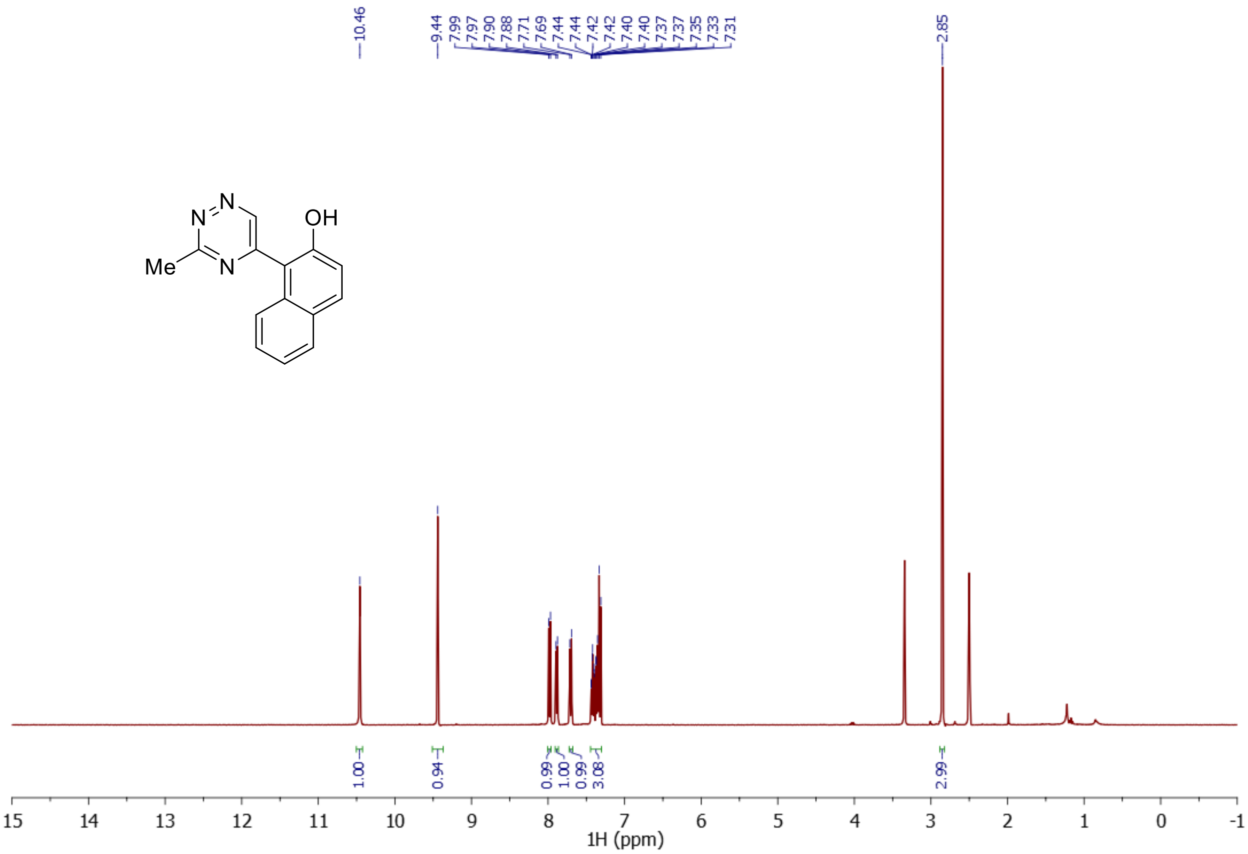

^{1}H NMR spectrum of 1-(3-methyl-1,2,4-triazin-5-yl)naphthalen-2-ol **5ka**

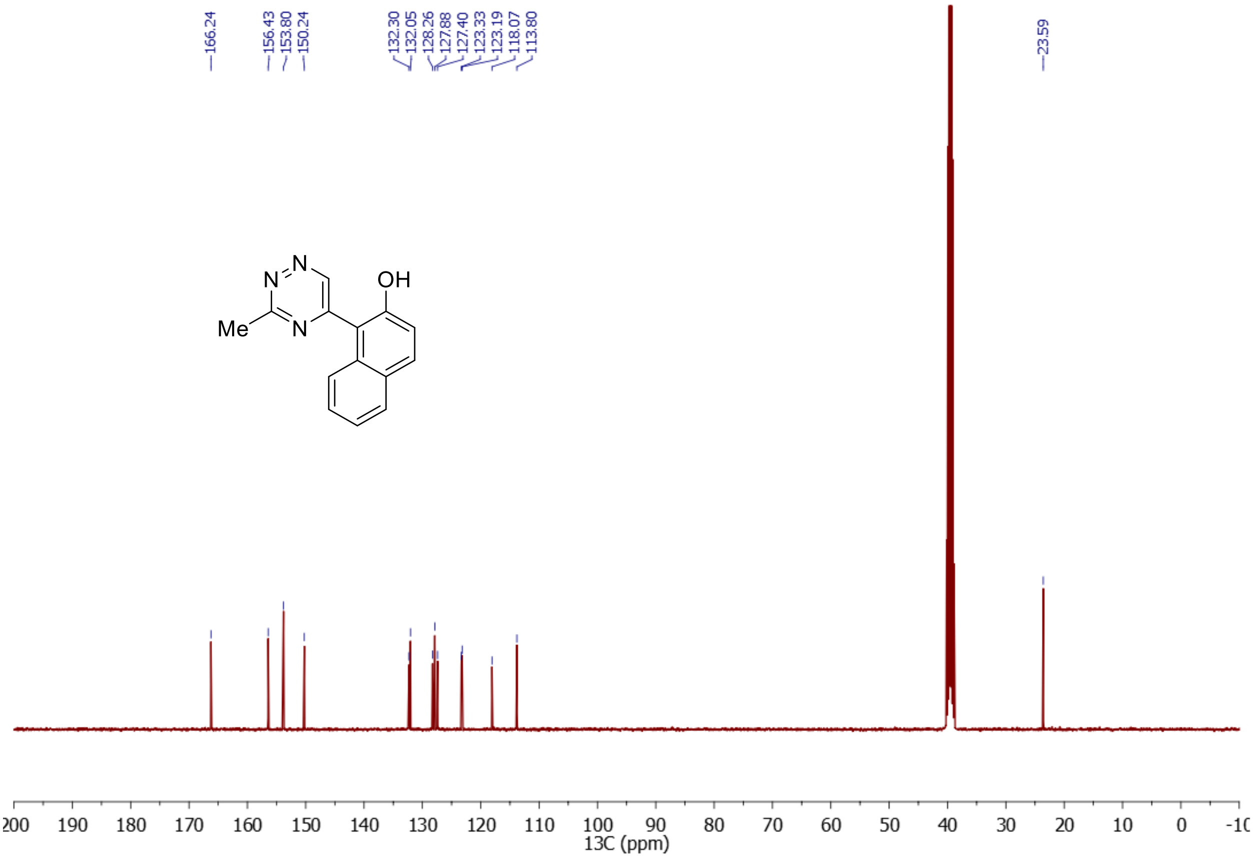

^{13}C NMR spectrum of 1-(3-methyl-1,2,4-triazin-5-yl)naphthalen-2-ol **5ka**

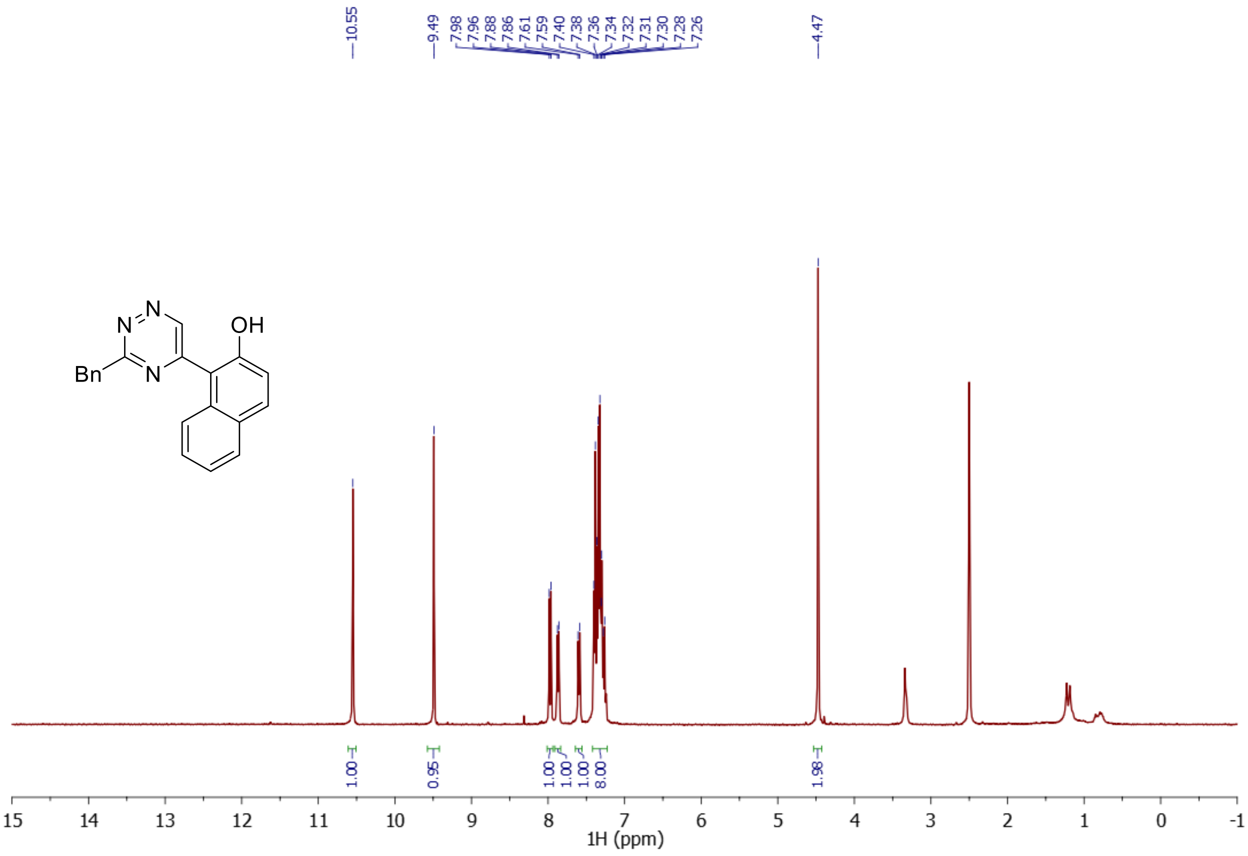

^{1}H NMR spectrum of 1-(3-benzyl-1,2,4-triazin-5-yl)naphthalen-2-ol **5la**

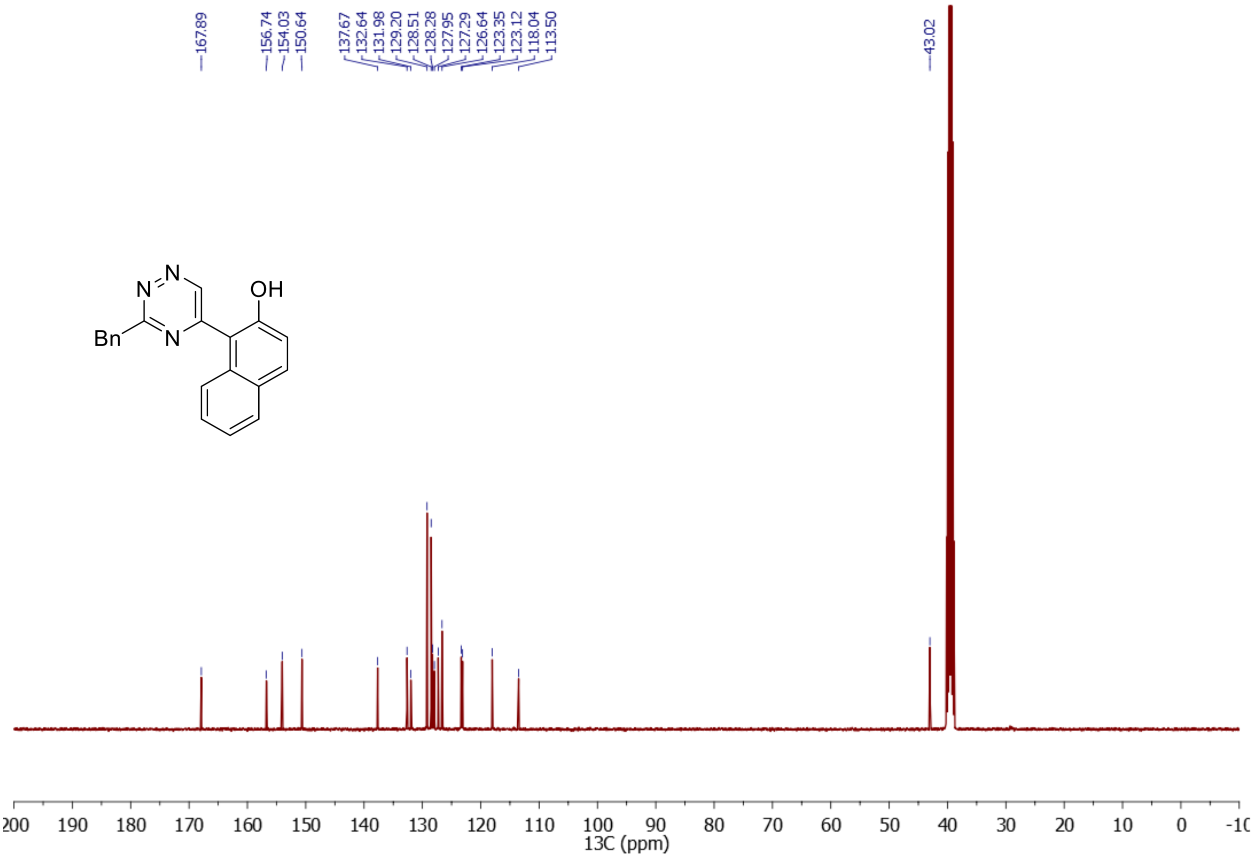

^{13}C NMR spectrum of 1-(3-benzyl-1,2,4-triazin-5-yl)naphthalen-2-ol **5la**

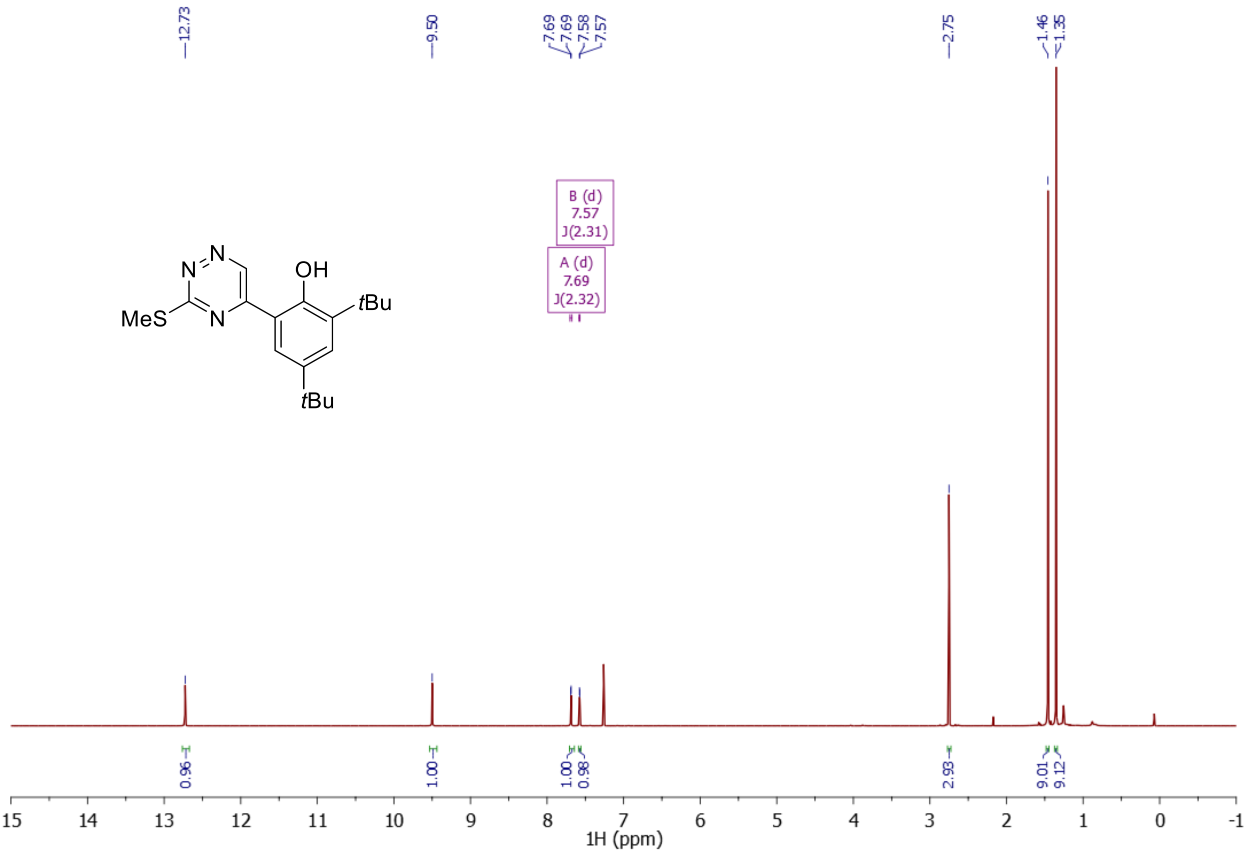

^{1}H NMR spectrum of 2,4-di-*tert*-butyl-6-(3-(methylthio)-1,2,4-triazin-5-yl)phenol **5ah**

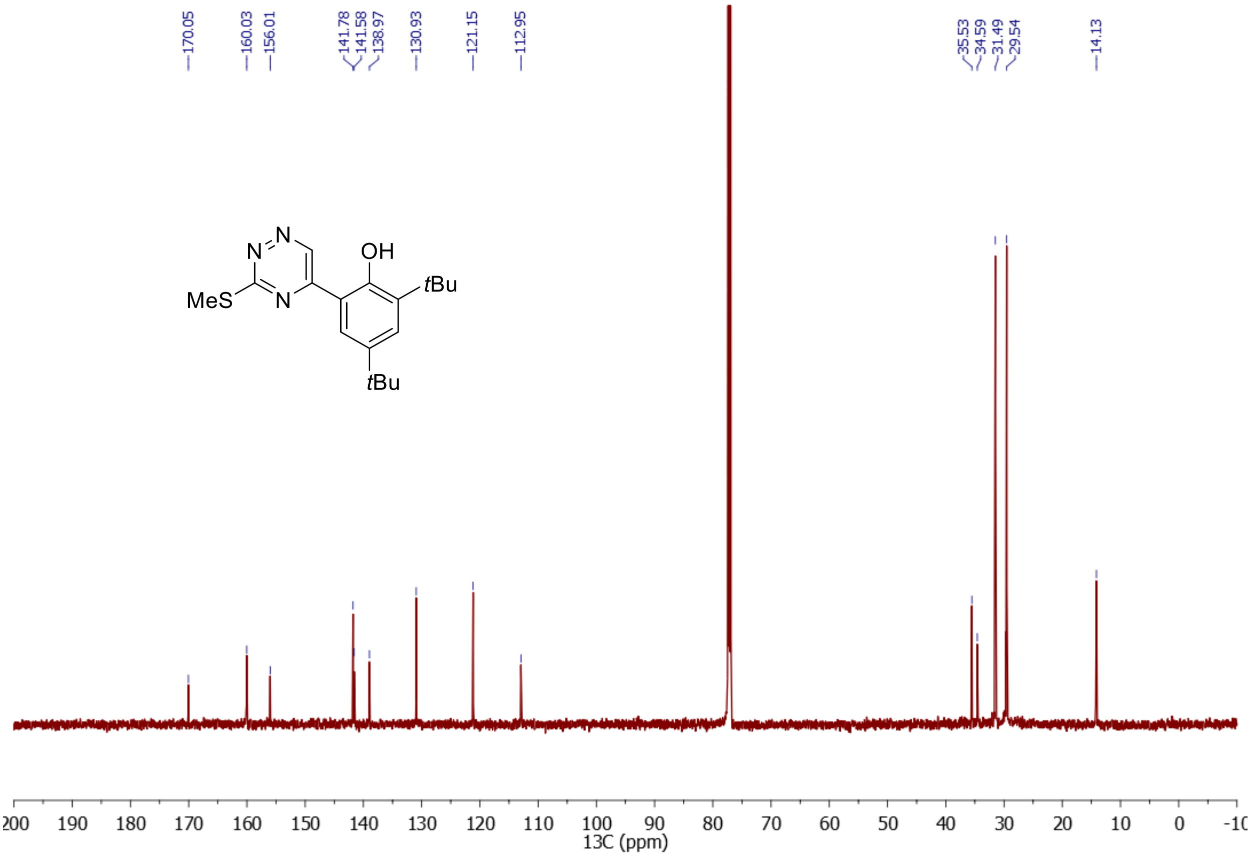

^{13}C NMR spectrum of 2,4-di-*tert*-butyl-6-(3-(methylthio)-1,2,4-triazin-5-yl)phenol **5ah**

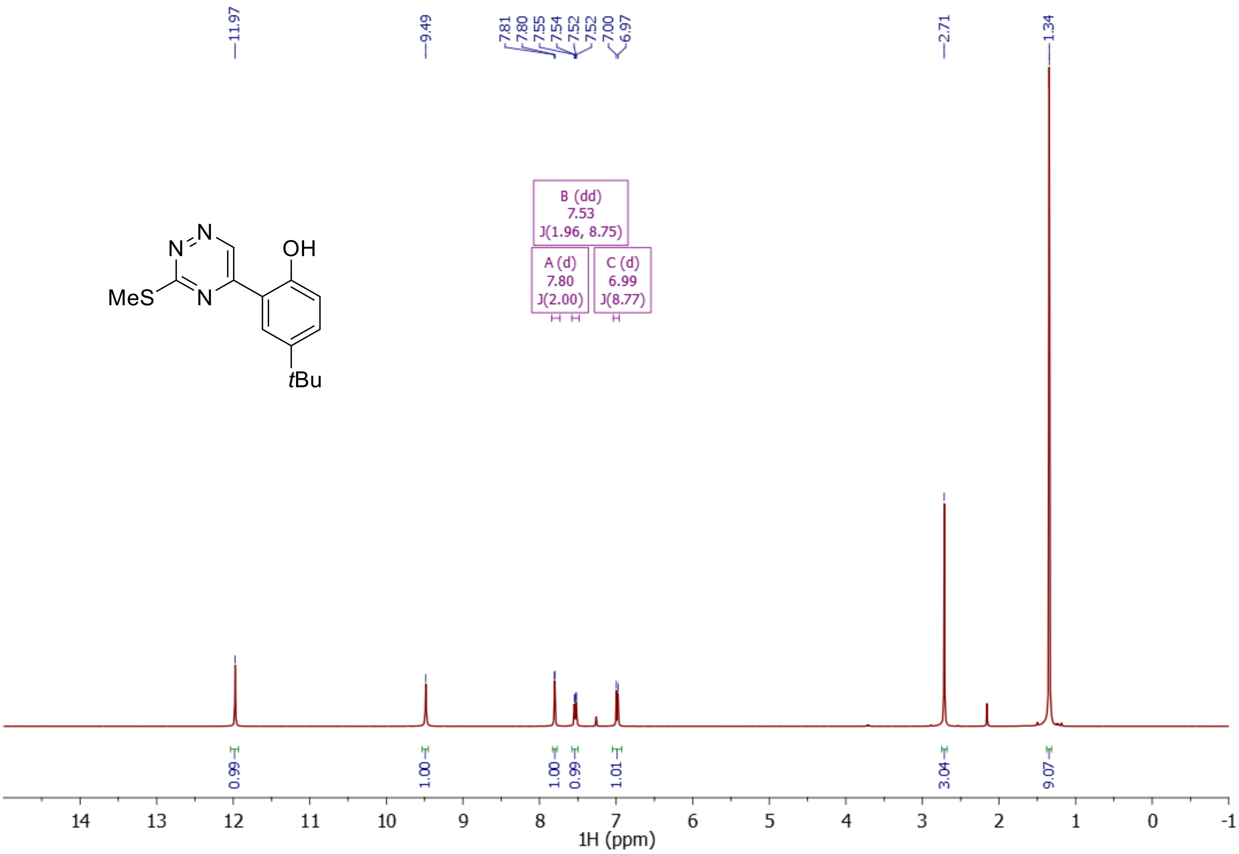

^{1}H NMR spectrum of 4-(*tert*-butyl)-2-(3-(methylthio)-1,2,4-triazin-5-yl)phenol **5ai**

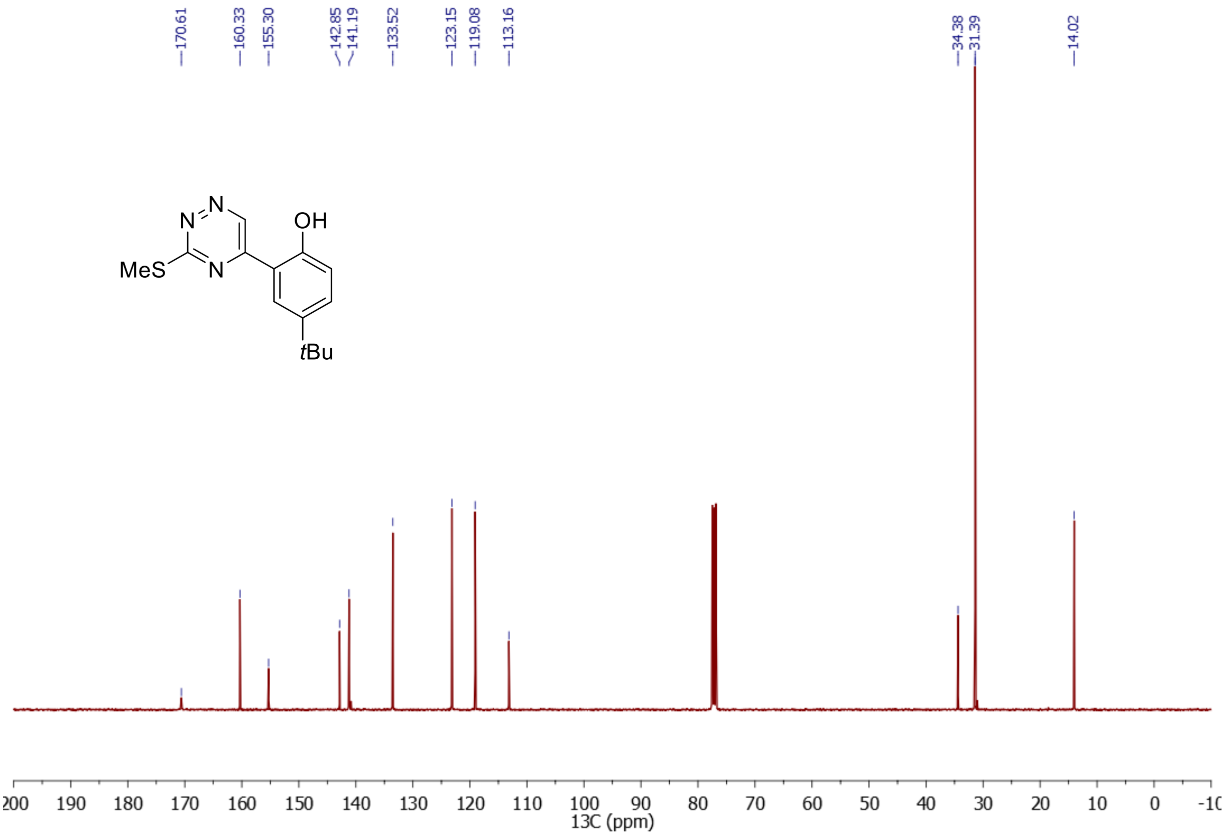

^{13}C NMR spectrum of 4-(*tert*-butyl)-2-(3-(methylthio)-1,2,4-triazin-5-yl)phenol **5ai**

Copies of ^{1}H and ^{13}C NMR spectra for compound **7**,**8**,**9**

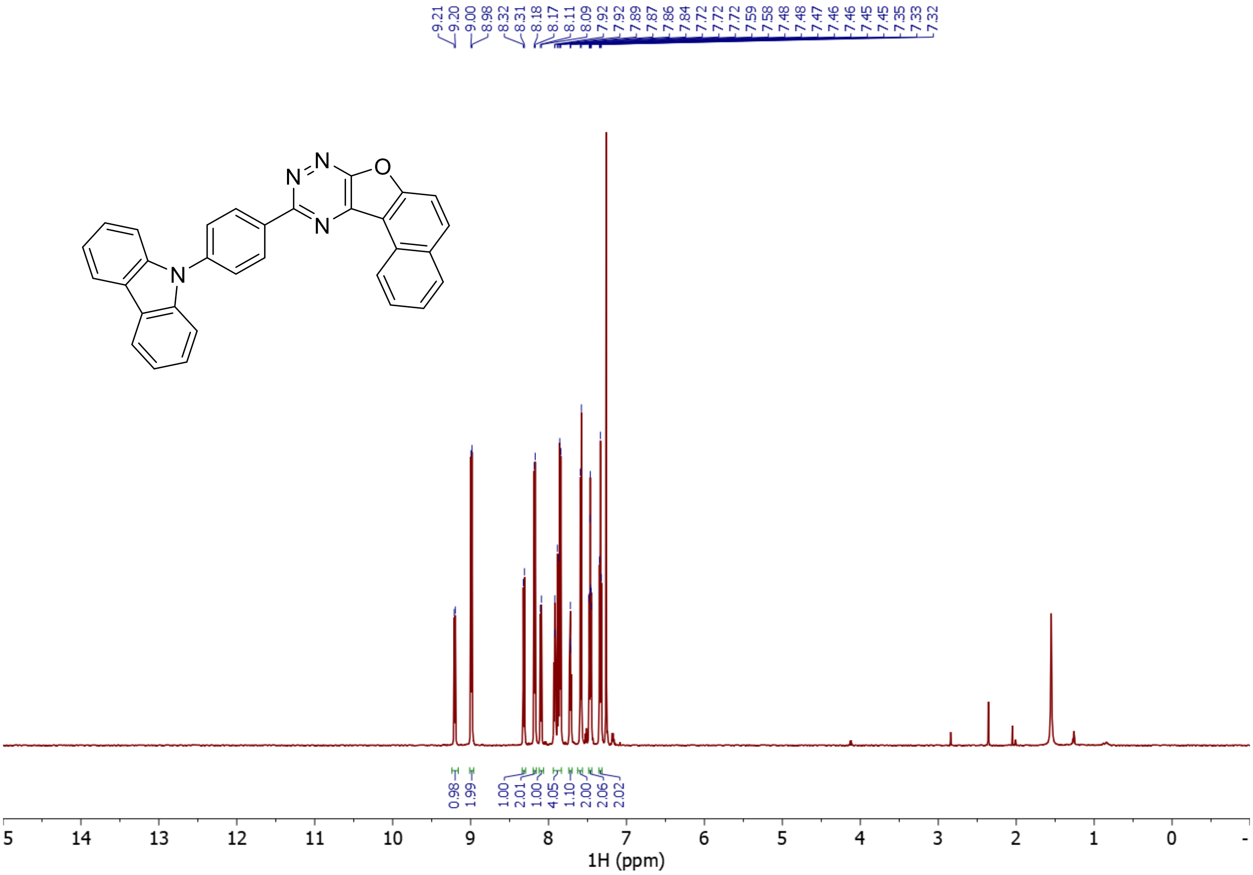

^{1}H NMR spectrum of 10-(4-(carbazol-9-yl)phenyl)naphtho[1',2':4,5]furo[3,2-*e*][1,2,4]triazine **7**

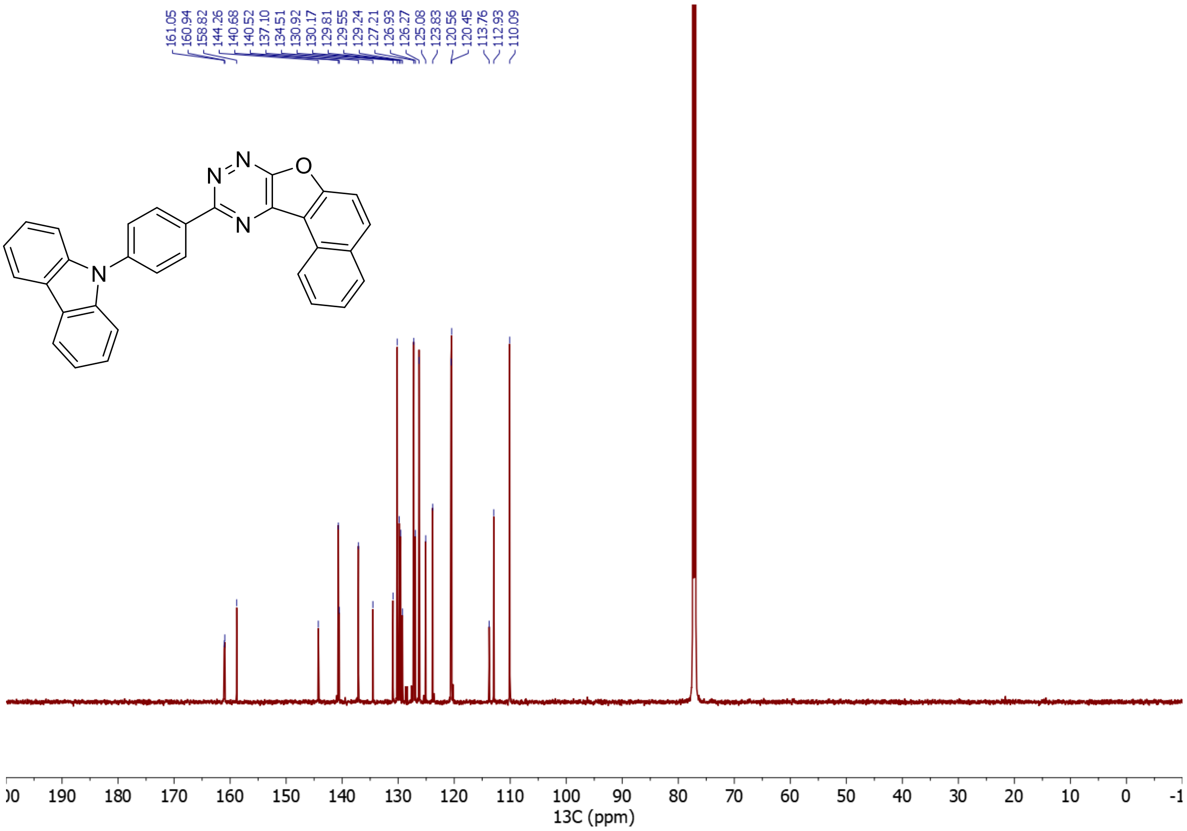

^{13}C NMR spectrum of 10-(4-(carbazol-9-yl)phenyl)naphtho[1',2':4,5]furo[3,2-*e*][1,2,4]triazine **7**

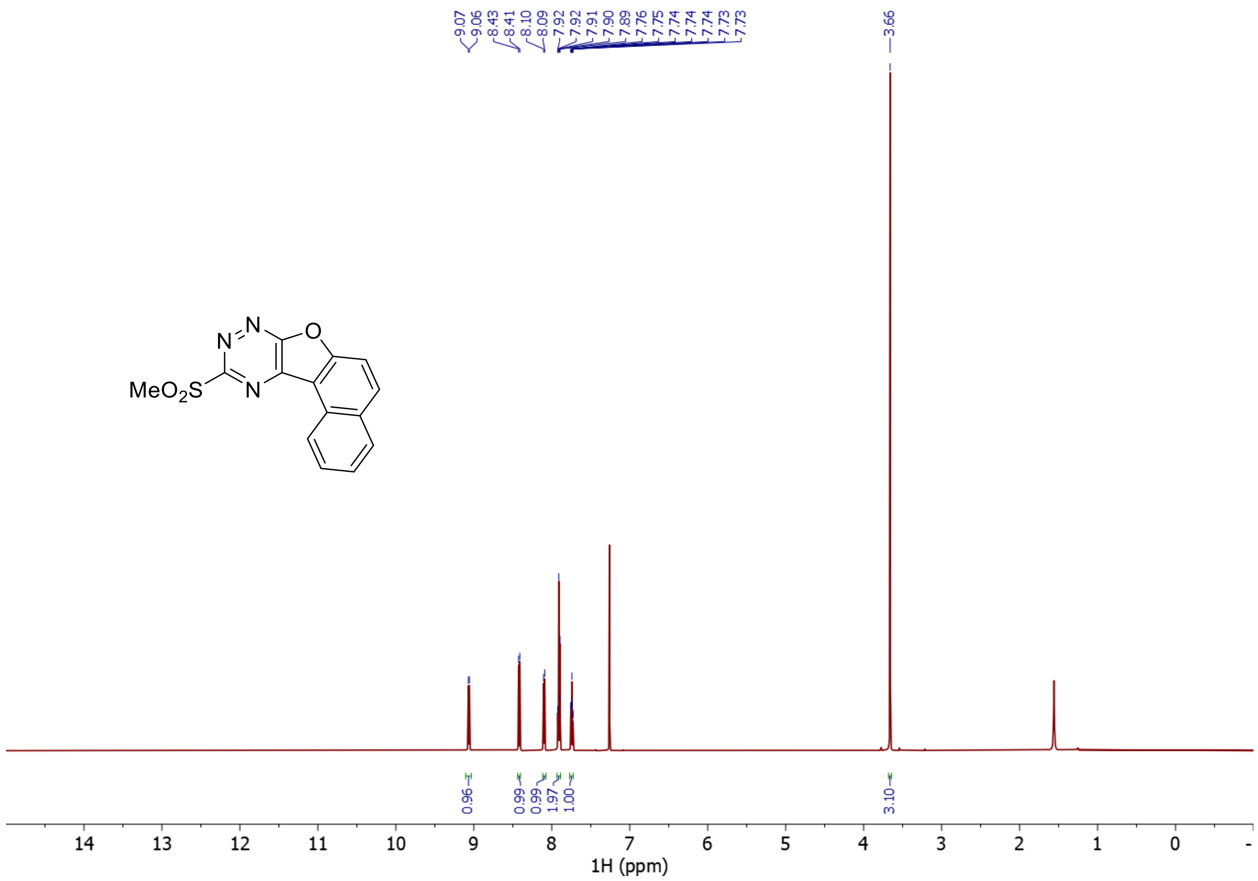

^{1}H NMR spectrum of 10-(methylsulfonyl)naphtho[1',2':4,5]furo[3,2-*e*][1,2,4]triazine **8**

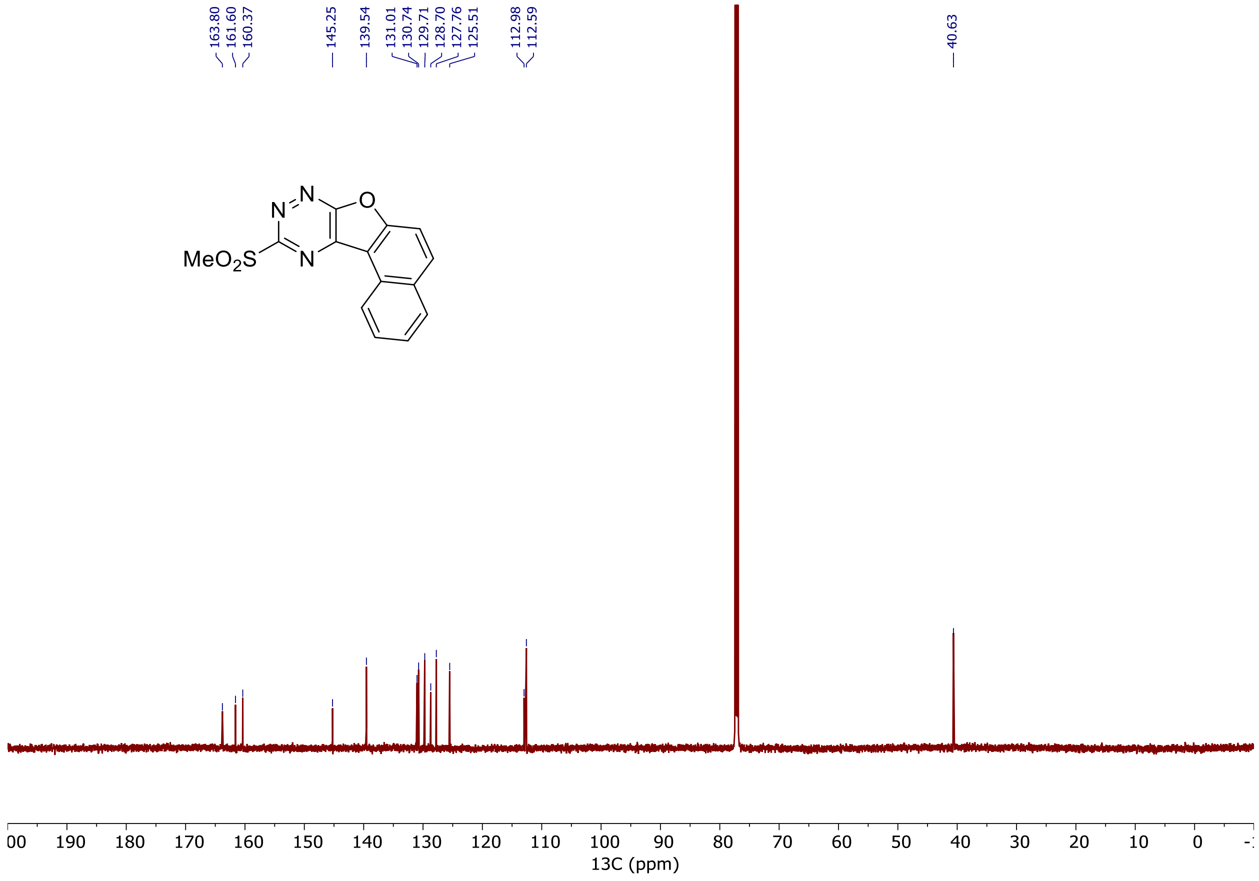

^{13}C NMR spectrum of 10-(methylsulfonyl)naphtho[1',2':4,5]furo[3,2-*e*][1,2,4]triazine **8**

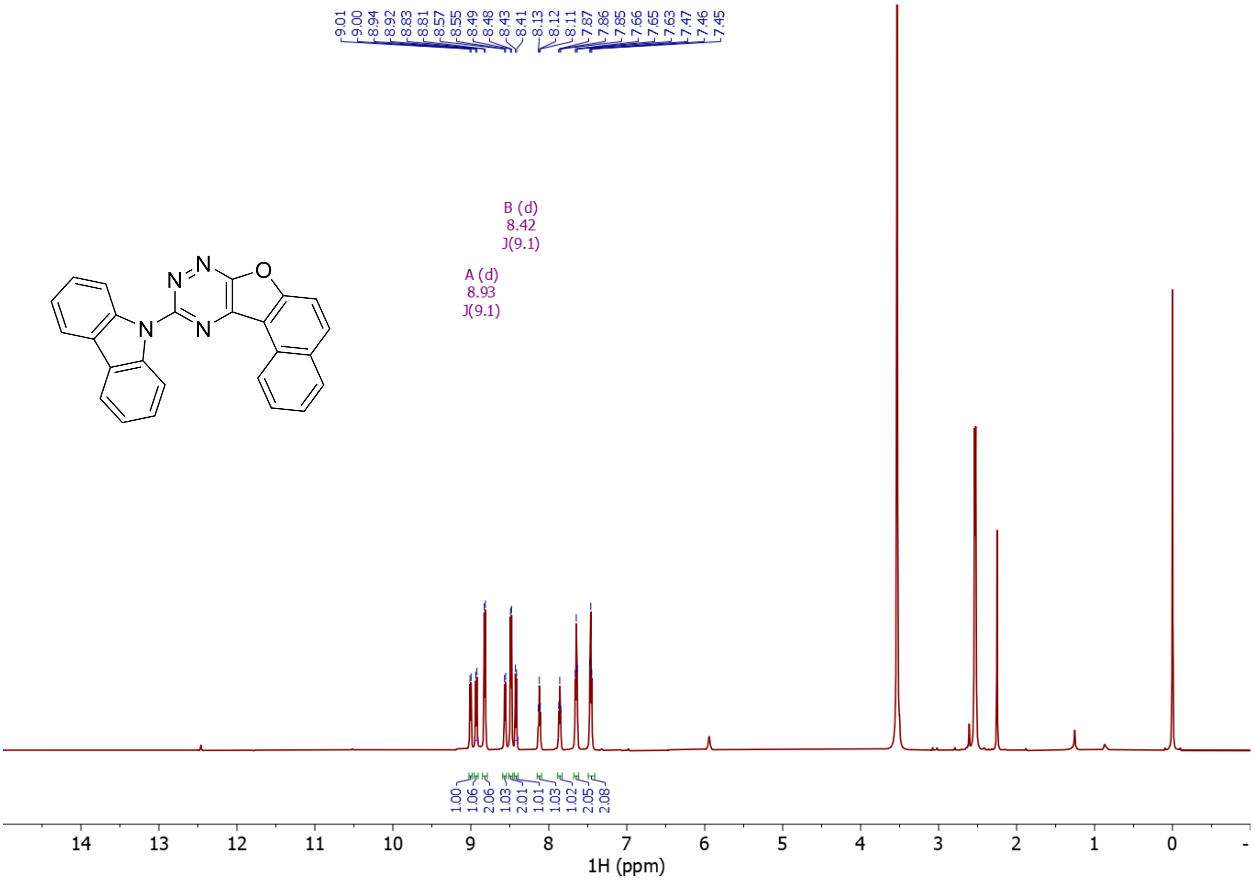

^{1}H NMR spectrum of 10-(9H-carbazol-9-yl)naphtho[1',2':4,5]furo[3,2-*e*][1,2,4]triazine **9**

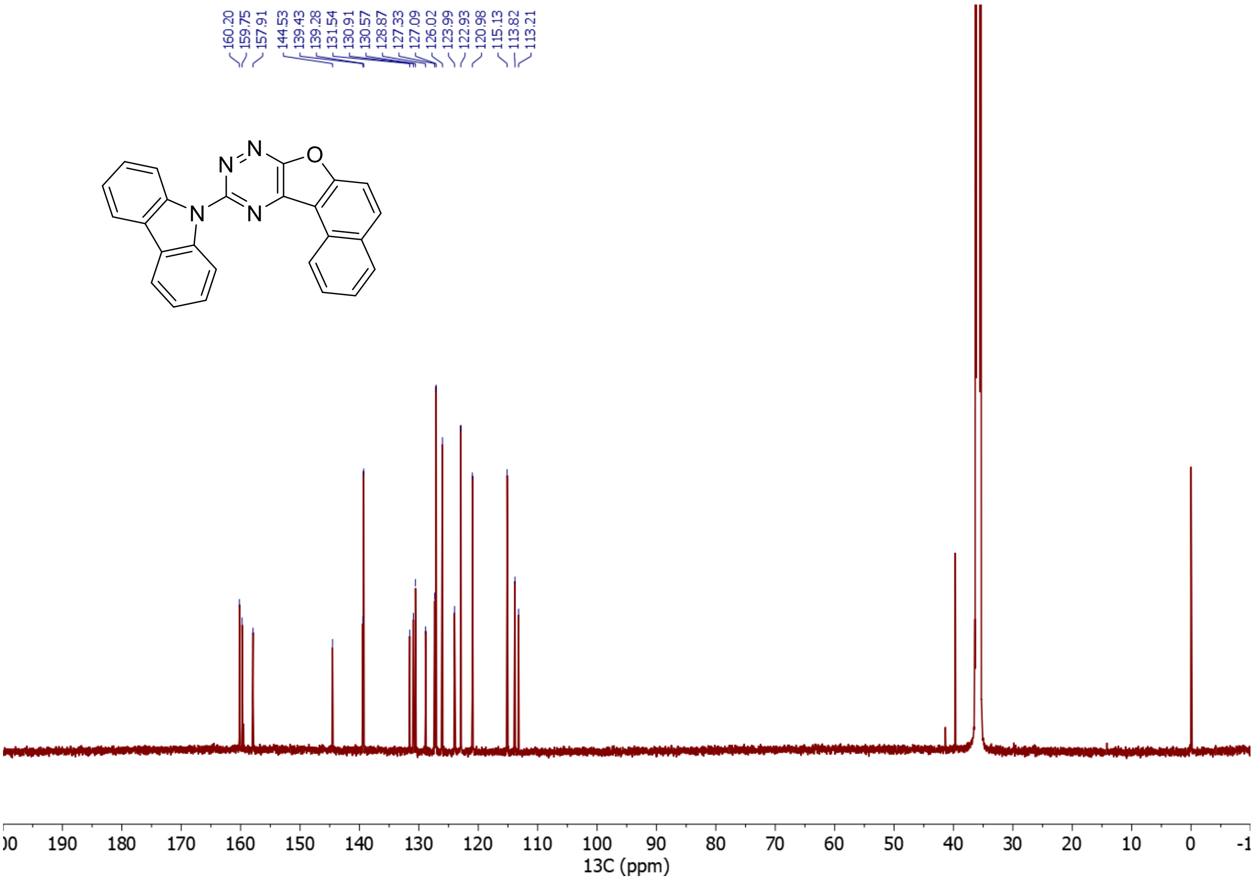

^{13}C NMR spectrum of 10-(9H-carbazol-9-yl)naphtho[1',2':4,5]furo[3,2-*e*][1,2,4]triazine **9**

Copies of ^{1}H and ^{13}C NMR spectra for compounds **4aa′**

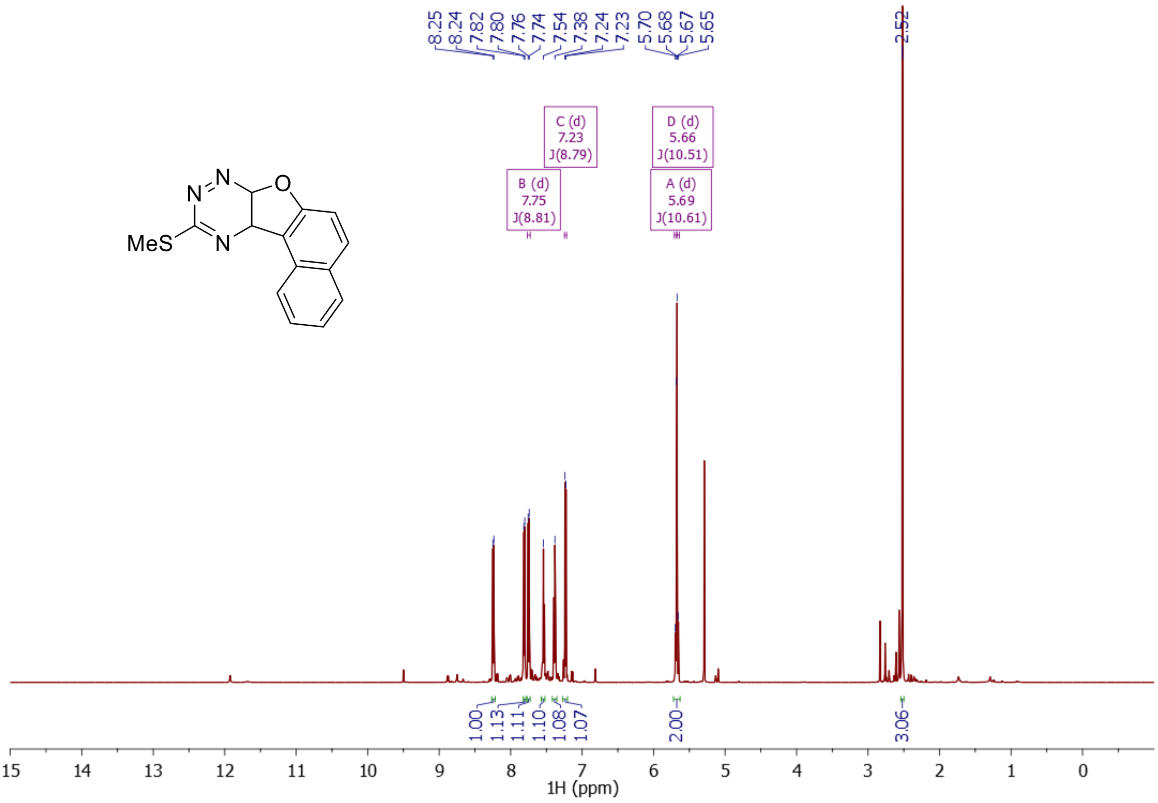

^{1}H NMR spectrum of 10-(methylthio)-7a,11a-dihydronaphtho[1',2':4,5]furo[3,2-*e*][1,2,4]triazine **4aa′**

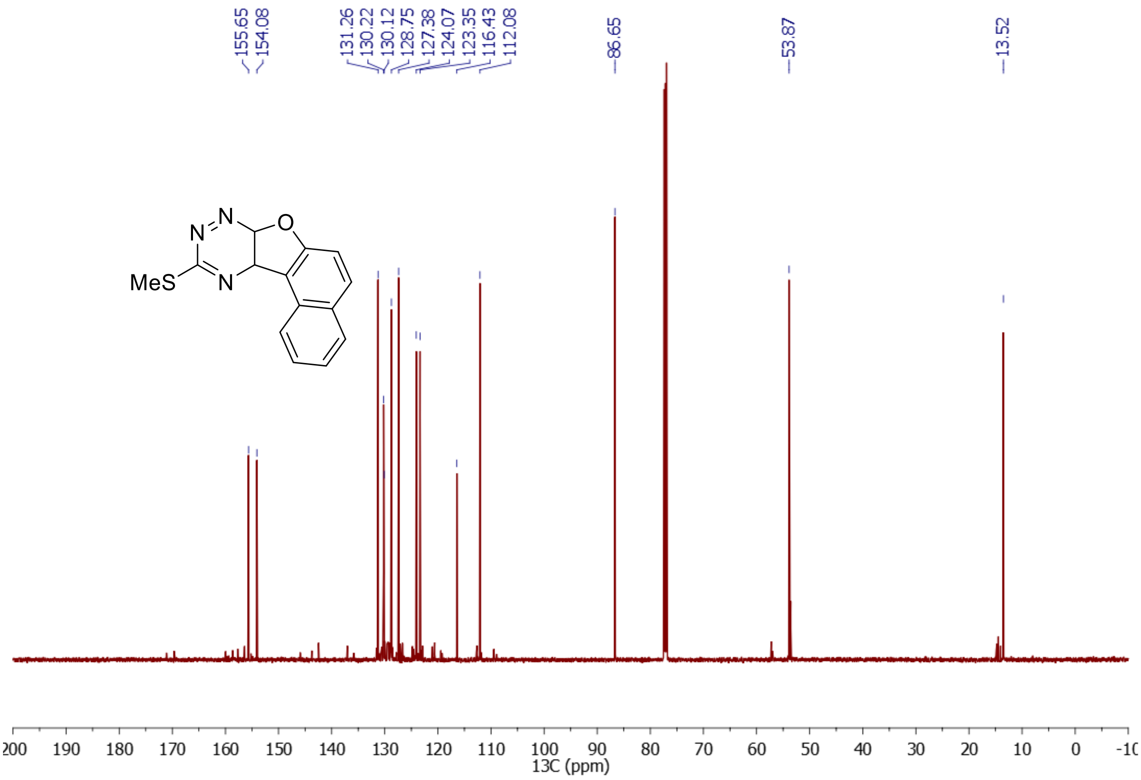

^{13}C NMR spectrum of 10-(methylthio)-7a,11a-dihydronaphtho[1',2':4,5]furo[3,2-*e*][1,2,4]triazine **4aa′**
